# Design, synthesis and biological evaluation of 4-aminoquinoline derivatives as receptor-interacting protein kinase 2 (RIPK2) inhibitors

**DOI:** 10.1080/14756366.2022.2148317

**Published:** 2022-11-21

**Authors:** Tiantian Fan, Yinchun Ji, Danqi Chen, Xia Peng, Jing Ai, Bing Xiong

**Affiliations:** a Department of Medicinal Chemistry, Shanghai Institute of Materia Medica, Chinese Academy of Sciences, Shanghai, P. R. China; b University of Chinese Academy of Sciences, Beijing, P. R. China; c Division of Antitumor Pharmacology, State Key Laboratory of Drug Research, Shanghai Institute of Materia Medica, Chinese Academy of Sciences, Shanghai, P. R. China; d Hangzhou Institute for Advanced Study (UCAS), Hangzhou, P. R. China

**Keywords:** RIPK2 inhibitor, NOD, immunity, inflammation

## Abstract

Receptor-interacting protein kinase 2 (RIPK2) is an essential protein kinase mediating signal transduction by NOD1 and NOD2, which play an important role in regulating immune signalling. In this study, we designed and synthesised a novel series of 4-aminoquinoline-based derivatives as RIPK2 inhibitors. *In vitro*, compound **14** exhibited high affinity (IC_50_ = 5.1 ± 1.6 nM) and excellent selectivity to RIPK2 showing in a dendrogram view of the human kinome phylogenetic tree. Bearing favourable lipophilicity and eligible lipophilic ligand efficiency (LipE), compound **14** was selected to investigate cellular anti-inflammatory effect and was identified as a potent inhibitor to reduce the secretion of MDP-induced TNF-α with a dose-dependent manner. Moreover, compound **14** showed moderate stability in human liver microsome. Given these promising results, compound **14** could serve as a favourable inhibitor of RIPK2 for further physiological and biochemical research so as to be used in therapeutic treatment.

## 1. Introduction

Receptor-interacting protein kinase 2 (RIPK2) belongs to RIPK family which consists of seven protein kinases that share homology in the serine-threonine kinase domain^1^. In addition to the kinase domain, each member has its unique domain structure enabling it to interact with proteins to execute specific cellular signalling processes^1^. In RIPK2, it contains a carboxy-terminal caspase activation and recruitment domain (CARD), which facilitates homotypic interactions with other CARD-containing proteins, especially the pattern recognition receptor nucleotide-binding oligomerization domain-containing proteins 1 and 2 (NOD1 and NOD2)^2^^,^^3^. These structure characteristics, together with biochemical studies, positioned RIPK2 as an essential protein kinase with the capacity to regulate immune signalling, triggering more researches and drug development on targeting RIPK2.

Based on the current knowledge, RIPK2 plays its main role in mediating signal transduction by NOD1 and NOD2^4^. Initially, NOD1 and NOD2 recognise diaminppimelic acid (iE-DAP)^5^and muramyl dipeptide (MDP) to recruit RIPK2 through the N-terminal CARD domain^6^. Then, RIPK2 undergoes ubiquitination by multiple E3 ubiquitin ligases to generate methionine 1-linked (M1) ubiquitin chains and lysine 63-linked (K63) chains on RIPK2 kinase domain^7^. In turn, these M1- and K63-Ub chains promote the recruitment and activation of the downstream kinases TAK1 and NF-κB inhibitor kinase IKKs^2^. Activation of these TAK1 and IKK complexes further leads to the activation of MAP kinases, degradation of IκB and activation of transcription factors NF-κB, and ultimately induces the production of proinflammatory cytokines and chemokines^1^^,^^2^^,^^8^.

Along with the advancement of proteomic techniques, recent studies gradually revealed the details of posttranslational regulation of RIPK2^3^^,^^4^^,^^7^^,^^9^^,^^10^. In response to NOD1/2 stimulation, RIPK2 was extensively ubiquinated by several E3 ligases, especially XIAP protein. Goncharov et al.^9^ identified that XIAP-BIR2 domain interacted with the RIPK2 and generated ubiquitin chain mainly at K538 and K410 site. Further CRISPR-Cas9 knockout cell system or RIPK2 mutant cell models confirmed that these ubiquitinations are essential for NOD2 signalling transduction. In addition to the ubiquitination of RIPK2, autophosphorylation of RIPK2 has been proposed to contribute to RIPK2 stability and form specific conformation to facilitate the XIAP binding^9^. Hrinka et al. demonstrated that some ATP-competitive RIPK2 inhibitors can interfere the XIAP binding, therefore modulating NOD signaling^4^.

**Figure 1.**
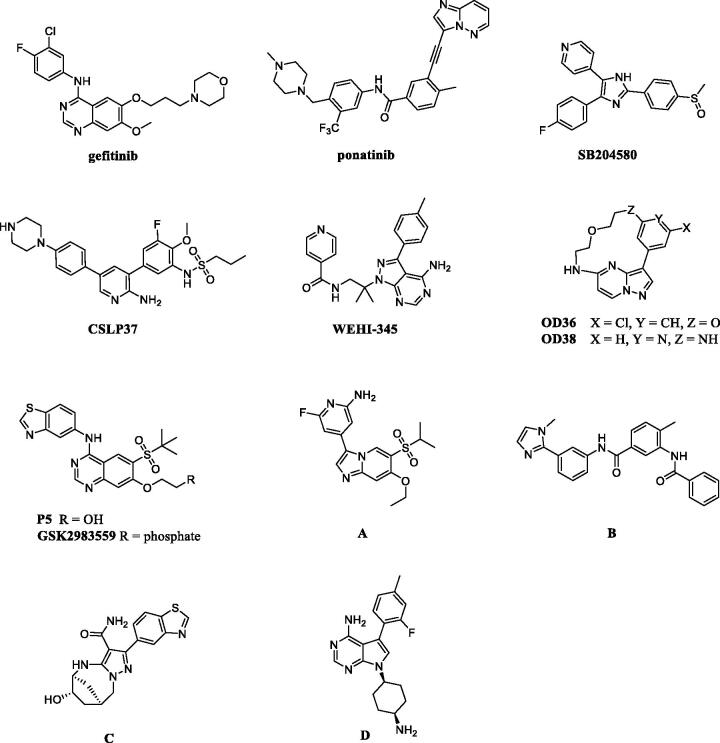
Representative inhibitors of RIPK2.

Given the importance of RIPK2 in immunity and cell death processes, RIPK2 was considered as a drug target for immunity-related disease treatment^7^^,^^11–13^. So far, a number of RIPK2 inhibitors blocked NOD2 signalling by antagonising RIPK2 have been reported in the literature (Figure 1). EGFR tyrosine kinase inhibitor Gefitinib^8^ and a pyridinyl imidazole inhibitor of p38 SB203580^14^, both as type I multi-kinase inhibitors, were previously used as a probe molecule to investigate the function of RIPK2. WEHI-345^10^, acting as type I kinase inhibitor, is more selective but less potent in cellular assays; While OD36, OD38^15^ and Ponatinib^16^ are type II kinase inhibitors, their poor overall kinome selectivity limited further development. Recently, more potent and selective RIPK2 inhibitors were discovered. These compounds were used as the chemical probes to investigate the RIPK2 kinase and NOD signalling functions. Although these compounds bear potent inhibition and good selectivity, moderate PK properties limited their further development to enter into clinical study, and more research was going on^17–21^. Notably, GSK2983559^22^, a prodrug of compound **P5**^23^, has good kinase specificity and excellent activity in vivo. It was entered into clinical study, but was halted the development due to toxicology findings and insufficient safety margins (NCT03358407). Although the detail of toxicity of GSK2983559 was unclear, we suspect it may associate with the chemical structure, and probably related to deleterious metabolites. Besides, Hrinka et al. demonstrated that some ATP-competitive RIPK2 inhibitors like Ponatinib and CSLP37 could efficiently abrogate XIAP binding to RIPK2 and block signalling in response to NOD2 stimulation^4^. Their study exemplified that binding to the ATP site of RIPK2 could interfere with the RIPK2-XIAP interaction, which could be exploited to develop RIPK2 inhibitors to investigate the potential of RIPK2 as an immunological therapeutic target. Herein, we reported our efforts on the discovery of new series of RIPK2 inhibitors based on the chemical modification of **P5**.

## 2. Results and discussion

### 2.1. Chemistry

**Scheme 1.**
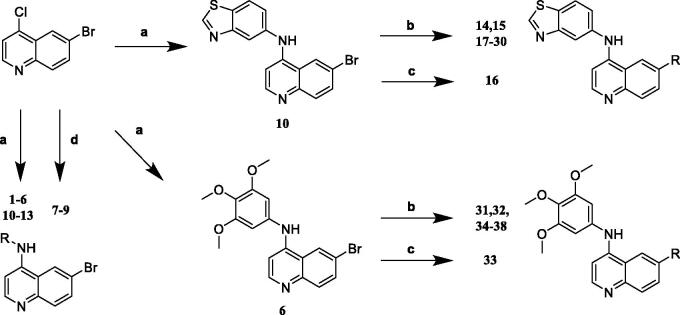
Synthesis of 4-aminoquinoline-based derivatives. Reagents and conditions: (a) 6-bromo-4-chloroquinoline, amines, tert-butanol, 80 °C, 4 h; (b) boronic acids or boronic esters, Pd(PPh_3_)_4_, Na_2_CO_3_, H_2_O, 1,4-dioxane, 80 °C, 8 h; (c) 2-pyridineboronic acid, Pd(OAc)_2_, DPPF, Cs_2_CO_3_, CuCl, DMF, 80 °C, 12 h; (d) 6-bromo-4-chloroquinoline, amines, NaH, DMF, 0–40 °C.

First of all, 6-bromo-4-chloroquinoline was reacted with a variety of amines by the substitution reaction of nucleophilic to obtain series of compounds (**1**–**13**). Later, compounds **14**–**30** were synthesised by Suzuki-Miyaura reaction utilising compound **10** with kinds of boronic acids or boronic esters. Finally, compound **6** was the precursor for compounds **31**–**38** which yielded in the same way (Scheme 1).

### 2.2. Structure activity relationships

As previously reported, the cocrystal structure of type I inhibitor **P5** bound within the ATP binding pocket of RIPK2 (PDB ID: 6RNA)^22^ addressed three momentous interactions: (1) The hinge hydrogen bonding interaction between N1 of quinazoline and backbone N–H of Met98; (2) The hydrogen bonding interaction between t-butyl sulphone group at C6 position and O–H of unique Ser25; (3) The hydrogen bond between N1 of 5-aminobenzothiazole and the catalytic residue Asp164. To retain the crucial hydrogen bond on hinge, we chose quinolinyl group as the core. The back pocket of RIPK2 was very sensitive, and small changes could affect the activity, so we chose some representative compounds with different electrical properties and sizes to replace, in order to find more appropriate groups to bind with the DFG region. As for C6 position, we thought that there was enough space for large groups, therefore, we explored more aromatic loops for this position. Based on these, chemical modification around the second and the third regions mentioned above were prepared to find selective, efficient and more stable RIPK2 inhibitors.

**Figure 2.**
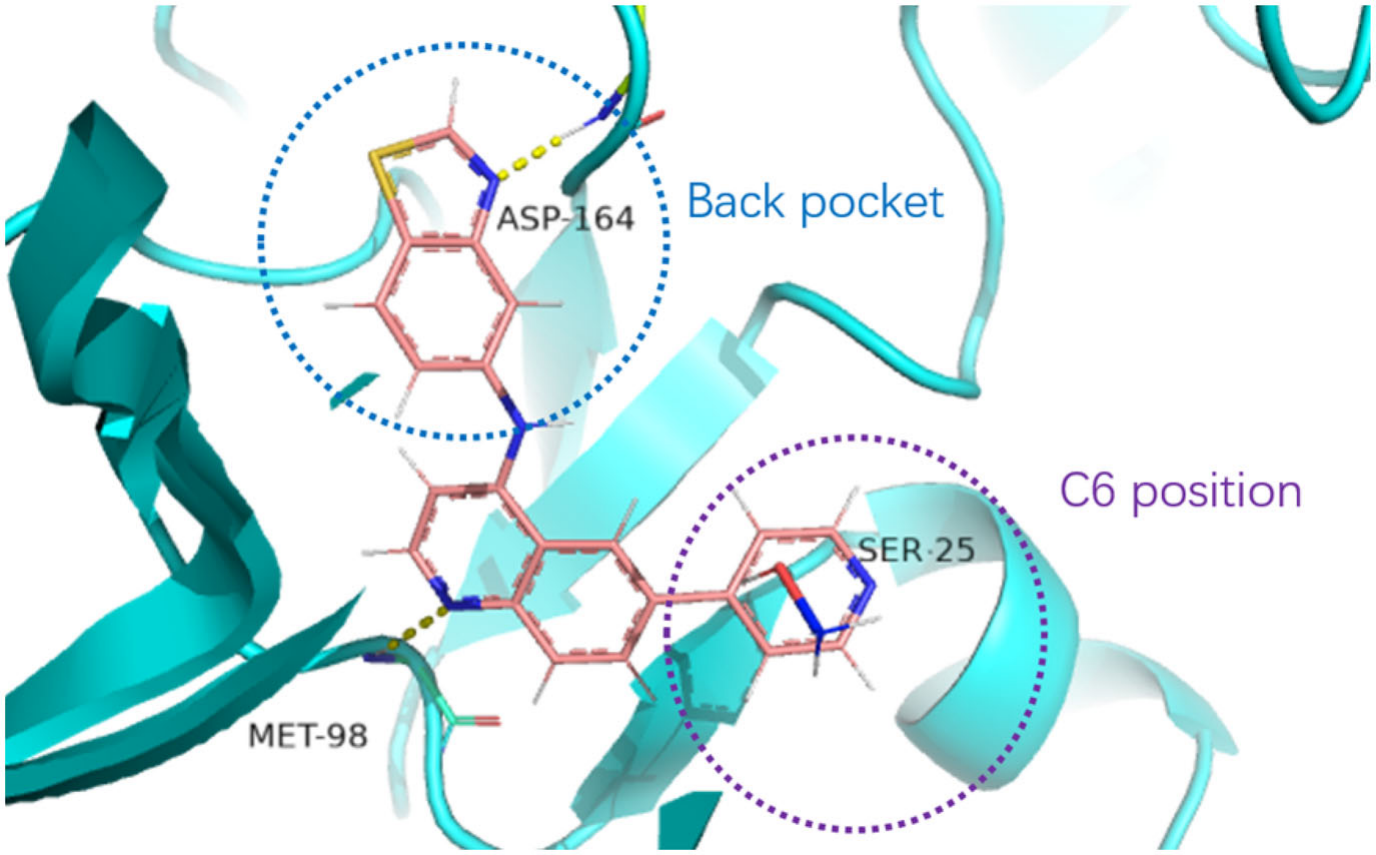
Docking study on compound **14** bound within the ATP site of RIPK2 (PDB ID: 6RNA).

Different SAR of RIPK2 inhibitors had been discovered between 4-aminoquinazoline series and 4-aminoquinoline series. Besides, RIPK2 has a high flexible back pocket in ATP-binding region. Thus, we synthesised a series of 4-aminoquinoline compounds which have different electric property and size to occupy the back pocket, hoping to explore some new fragments fitted. As for C6 position, we kept Br atom to retain the interaction with Ser25 by halogen bond^24^. Typically, the back pocket has catalytic residue Asp164 which may act either as an H-bond donor or a receptor, so we considered the substitution on the meta-position of the phenyl ring, interacted with the carboxyl group of Asp164, as well as single or bicyclic rings (Table 1). Substitutions that might form H-bonds with Asp164 were introduce to meta-position of phenyl ring (**1–6**). When a methoxy group (**2**) was adopted, the inhibition activity was about 5 times to the bare phenyl (**1**). However, the usage of an isopropoxy group (**3**) led to the loss of the activity, which suggested the isopropyl might hinder the interaction for big size. Acetyl group (**4**) and fluorine atom (**5**) could also form H-bonds and the IC_50_ of these compounds were less than 100 nM. When the phenyl ring was substituted by multiple methoxy groups (**6**), the inhibition activity increased a lot to about 10 nM. This might be due to the formation of H-bond and the appropriate size filled back pocket. Additionally, electron donating moieties seemed more favourable (**6** vs **2**, **2** vs **4**). Later, phenyl rings were replaced by pyridinyls, the meta-position (**8**) showed better activities than ortho- (**7**) and para-positions (**9**) and these compounds with pyridinyls showed decreased potency comparing to the substituted phenyls. The distance between N atom and Asp164, as well as electron deficiency could explain these distinctions. We also considered bicyclic rings to occupy the back pocket and found p-π interactions with Lys47. In addition, benzo[*d*]thiazole (**10**, **11**), indazole (**12**) and indole (**13**) rings attached in different way that the N atom of benzo[*d*]thiazole was supposed to be an H-bond receptor to interact with Asp164, while the N atom of indole ring was supposed to be an H-bond donor. Obviously, the N atoms at the meta-position (**10**, **12**) were more active and about 6 times higher than those at para-position (**11**, **13**). Ultimately, for the first round of the optimisation, we identified the trimethoxyphenyl group groups (**6**) and benzo[*d*]thiazol-5-amine (**10**) as dominant segments to occupy the back pocket of RIPK2 (Figure 2).

**Table 1.** Modifications in back pocket of RIPK2.

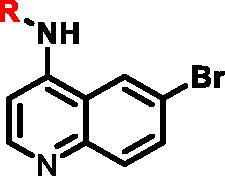		
Compd.	*R*	RIPK2 IC_50_ (nM)^a^
**1**	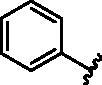	243.4 **±** 14.8
**2**	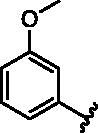	54.8 **±** 3.4
**3**	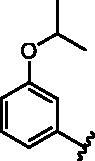	>1000
**4**	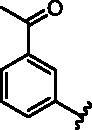	92.5 **±** 22.1
**5**	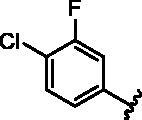	31.4 **±** 6.2
**6**	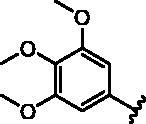	10.7 **±** 0.1
**7**	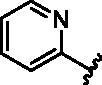	>1000
**8**	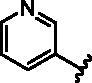	150.8 **±** 30.1
**9**	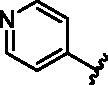	421.2 **±** 105.9
**10**	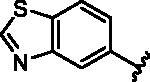	12.2 **±** 2.5
**11**	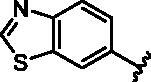	82.3 **±** 12.2
**12**	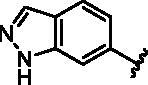	22.2 **±** 5.2
**13**	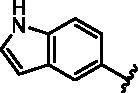	118.2 **±** 21.5

^a^The IC_50_ values are shown as the mean ± *SD* from two separate experiments. Positive control Ponatinib IC_50_ = 8.2 ± 2.9 nM.

The benzo[*d*]thiazole from **10** was kept during the exploration of substitutions on C6 position of the core quinoline. C6 position has been proved to be associated with selectivity of RIPK2 inhibitors, due to a rare polar residue Ser25 in the glycine-rich loop (Figure 2). In addition, occupying this solvent accessible space might probably interfere with the RIPK2-IAP interaction. Thus, we did a more detailed exploration of this area adopted lager moieties compared to previous studies. As there is a hydroxyl group in serine, it can be either an H-bond donor or a receptor that interacts with the small molecular inhibitors. Thus, a series of aromatic derivatives containing structures that might form H-bonds and differ from t-butyl sulphone at C6 position were synthesised (Table 2). Great potency (IC_50_ = 1.5–6 nM) were showed on the compounds with pyridinyl connecting to ortho-, meta- or para-positions (**14–16**) and pyrimidinyl (**17**), suggesting that H-bond receptors with Ser25 were more favourable when compared to phenyl ring (**18**). When R were substituted by pyrazoles, the IC_50_ values were about 4.1–19 nM. A methyl group in ortho-position improved the activities (**21** vs **20**, **23** vs **19**), this might because electron donating groups would strengthen the bonding between quinoline N atom with Met98. The destruction of the hydrogen bonding with Ser25 could be charged for the decrease in activity when both ortho-positions were occupied (**22**) or the pyrazole was replaced by another type of five-member ring thiophene (**24**). Bicyclic rings containing at least one H-bond donor or receptor, such as indole, indazole, pyrrolo[2,3-*b*]pyridine and isoquinoline were adopted in our study as well (**25**–**30**). Most of bicyclic rings (**25**–**29**) retained potency (IC_50_ < 20 nM), indicating a large space that can accommodate in this area. Besides, the decrease activity of compound **30** suggested that the orientation of extension seemed important compared to compound **29**. In this part, we identified some lager substitutions with high affinity on C6 position which could be utilised later.

**Table 2.** Optimisation on C6 position.

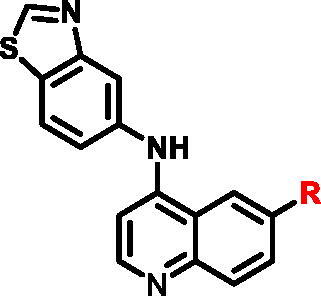		
Compd.	*R*	RIPK2 IC_50_ (nM)^a^
**14**	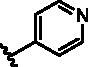	5.1 ± 1.6
**15**	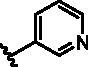	6.0 ± 1.5
**16**	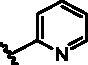	3.6 **±** 1.4
**17**	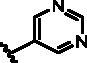	1.5 **±** 0.3
**18**	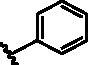	24.0 **±** 11.6
**19**	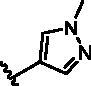	16.4 **±** 1.6
**20**	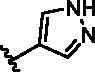	8.2 **±** 1.6
**21**	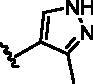	4.1 **±** 0.9
**22**	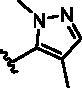	19.0 **±** 6.7
**23**	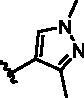	9.5 **±** 3.7
**24**	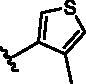	19.6 **±** 12.0
**25**	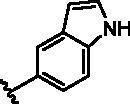	11.1 **±** 3.6
**26**	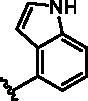	9.4 **±** 1.1
**27**	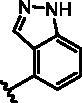	3.7 **±** 0.4
**28**	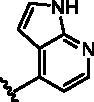	7.3 **±** 3.5
**29**	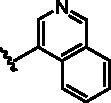	17.2 **±** 2.3
**30**	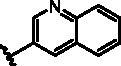	41.7 **±** 19.0

^a^The IC_50_ values are shown as the mean ± *SD* from two separate experiments. Positive control Ponatinib IC_50_ = 8.2 ± 2.9 nM.

In addition to a series of active compounds with benzo[*d*]thiazol-5-amine group, compound **6** with trimethoxyphenyl group exhibited strong inhibition on RIPK2 (Table 1). Combined with the advantageous structures obtained from Table 2, some potent compounds were designed and synthesised (Table 3). These compounds all showed strong inhibition on RIPK2 with IC_50_ value less than 20 nM. Pyridinyls (**31**–**33**) basically kept the activities while the pyrimidinyl exhibited a little decrease from 1.5 nM to 11 nM. Compounds with indazoles and pyrazoles (**35**–**38**) showed slightly difference within two to three times of inhibition activities to the corresponding compounds in Table 1. Eventually, we obtained a new group exhibited excellent activity.

**Table 3.** Inhibition of compounds **31–38** on RIPK2.

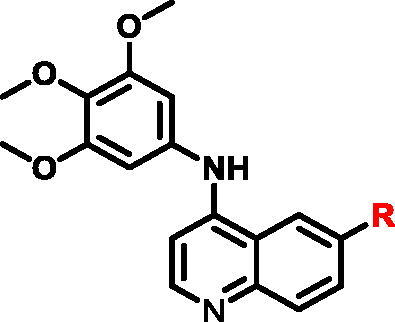		
Compd.	*R*	RIPK2 IC_50_ (nM)^a^
**31**	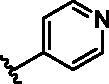	8.1 **±** 3.9
**32**	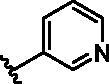	2.4 **±** 0.2
**33**	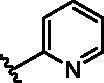	12.4 **±** 8.9
**34**	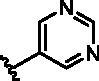	11.0 **±** 1.7
**35**	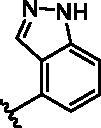	7.1 **±** 4.7
**36**	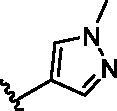	7.2 **±** 2.6
**37**	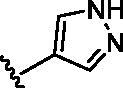	14.5 **±** 5.6
**38**	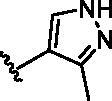	19.2 **±** 0.7

^a^The IC_50_ values are shown as the mean ± *SD* from two separate experiments. Positive control Ponatinib IC_50_ = 8.2 ± 2.9 nM.

### 2.3. Cellular anti-inflammatory effect studies

**Figure 3.**
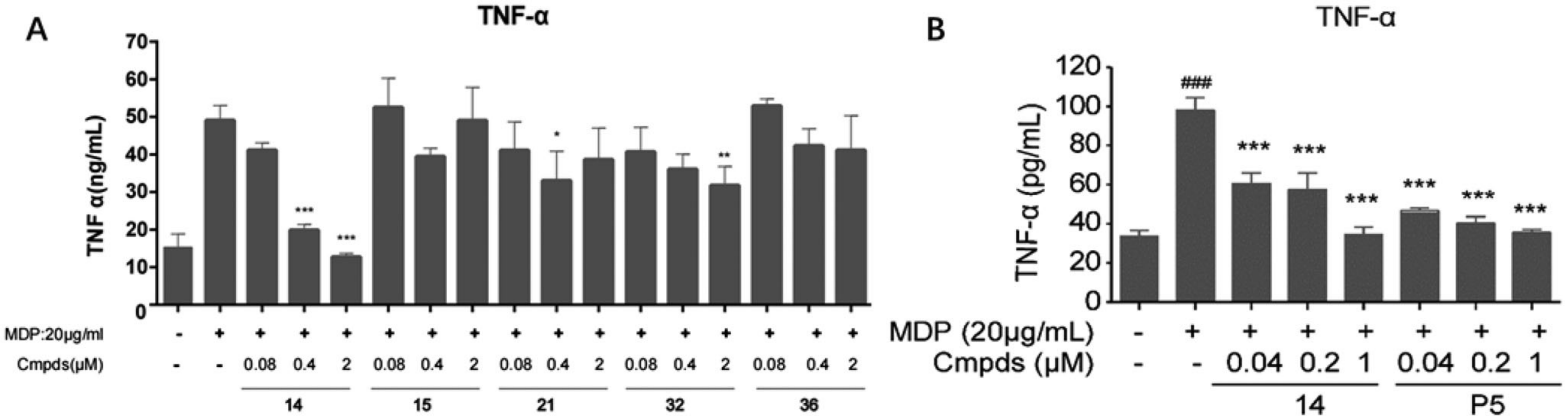
The effect of indicated compounds on the secretion of classic proinflammatory cytokine TNF-α in MDP-induced Raw264.7. TNF-α secretion in the supernatant of Raw264.7 cells from controls without stimulated, controls stimulated and compounds treated under stimulated conditions. The concentrations are presented as the mean ± *SD*. ^###^*p* < 0.001 vs non-stimulated controls group, *p < 0.05, **p < 0.01, ***p < 0.001 vs stimulated controls group, in multivariate analysis.

Compound **14**, **15**, **21**, **32** and **36** were further selected to investigate their cellular anti-inflammatory effect. These compounds had high affinity, favourable lipophilicity, eligible lipophilic ligand efficiency (LipE) and good selectivity over RIPK1 (Table 4). As shown in Table 4, all the selected compounds had similar clogP values in the range of 3.4–4.5, while compound **P5** was more hydrophilic and exhibits lower clogP value (clogP = 1.88). This was consistent with the properties of chemical structures, as we intended to incorporate more aromatic rings into the scaffold to make different physicochemical RIPK2 inhibitors, which might have differential pharmacokinetic properties. Since RIPK2 played a central role in the NOD signalling-mediated pro-inflammatory cytokine production^10^, the influence of these compounds on the secretion of classic proinflammatory cytokine TNF-α in MDP-induced Raw264.7 was tested with series concentration. As expected, upon NOD2 pathway activator MDP treatment, the production of TNF-α was dramatically increased. While such increase was inhibited with different extent by the compounds. Among them, compound **14** exhibited a strong inhibitory activity on MDP-induced TNF-α secretion with a dose-dependent manner (Figure 3(A)). However, compound **15**, which were only different in solvent region compared to compound **14**, had almost no inhibition on MDP-induced TNF-α secretion. We suspected that different groups occupied C6 position might affect the RIPK2- XIAP interaction, possibly through the intricate conformational change, which further modulated the transduction of NOD signalling. Furthermore, GSK2983559 active metabolite **P5** and compound **14** were tested in parallel (Figure 3(B)), compound **14** showed similar potence as **P5**. These results suggested that this series compounds, in particular compound **14**, potently blocked MDP-induced RIPK2-dependent inflammatory mediator production.

**Table 4.** Selectivity, ClogP and LipE of compound **14, 15, 21, 32, 36**.

Compd.	RIPK2 IC_50_ (nM)^a^	RIPK1 IR@1µM (%)	ClogP	LipE^b^
14	5.1 ± 1.6	<1	3.89	4.40
15	6.0 ± 1.5	<1	3.95	4.27
21	4.1 **±** 0.9	<1	3.40	4.99
32	2.4 **±** 0.2	19	4.32	4.30
36	7.2 **±** 2.6	<1	4.47	3.67
P5	2.6 ± 1.2	–	1.88	6.70

^a^The IC_50_ values are shown as the mean ± SD from two separate experiments. Positive control Ponatinib IC_50_ = 8.2 ± 2.9 nM. ^b^LipE = pIC_50_ − ClogP.

### 2.4. Kinase selectivity studies

**Figure 4.**
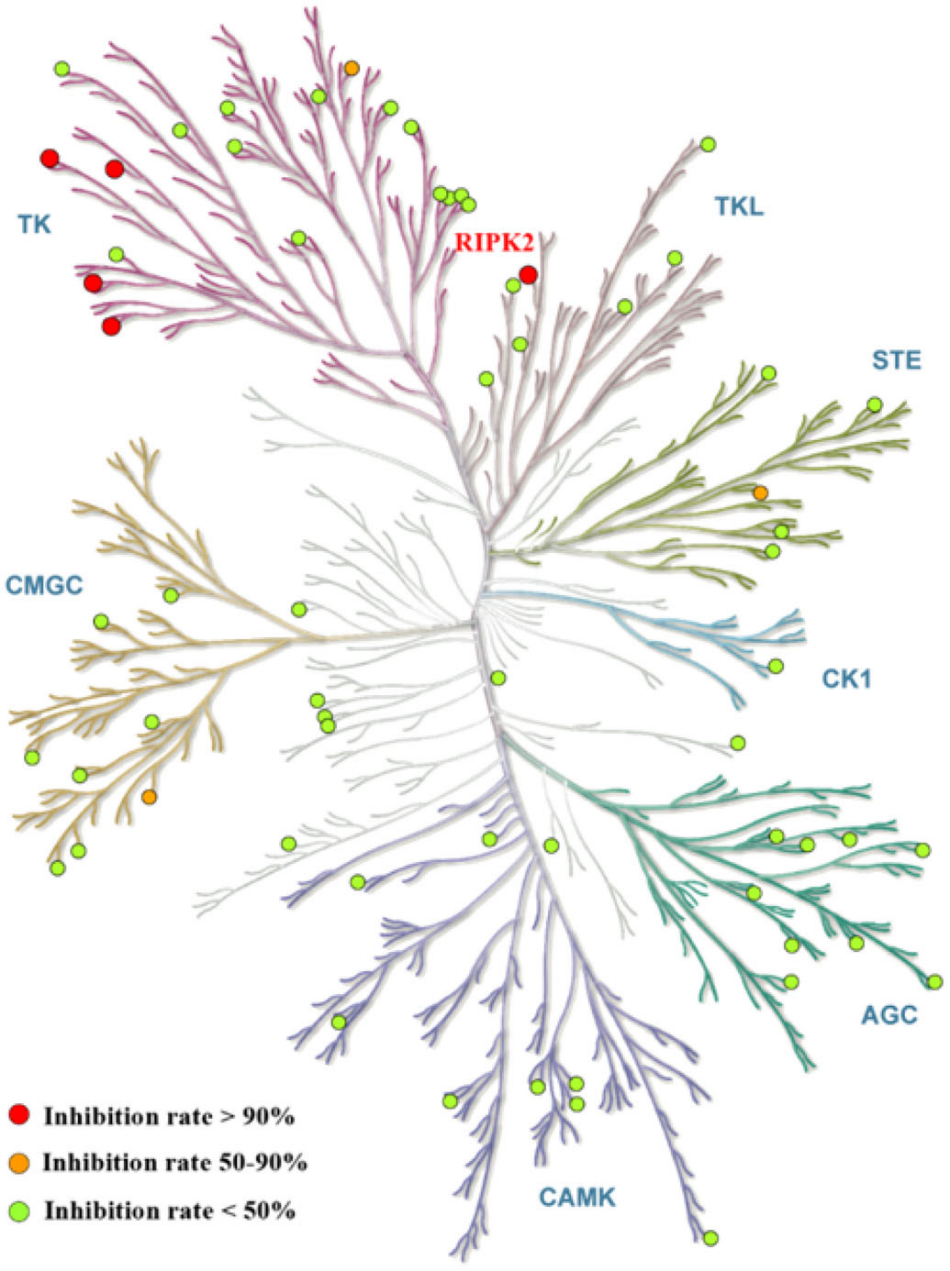
Compound **14** selectivity is represented in a dendrogram view of the human kinome phylogenetic tree^25^. Red: >90% inhibition (5 kinase). Orange: 50 − 90% inhibition (3 kinases). Green: <50% inhibition. RIP2 kinase (red) inhibited by 97%.

To confirm the anti-inflammatory effect was caused by RIPK2 inhibition, compound **14** was subjected to kinase selectivity evaluation (Figure 4). Kinase selectivity profile of compound **14** as shown by the diversity kinase Panel screen against 70 kinases assayed at 1 µM. Among all tested 70 kinases, only five were inhibited over 90% at 1 µM including Fyn, Lyn, BTK, Abl and RIPK2. Three kinases including KDR, CDK9 and LOK were inhibited 50–90% at 1 µM. Compound **14** exhibited high kinase selectivity, making it possible for future research and development.

### 2.5. Human liver microsome stability studies and AO oxidase studies

As compound **14** showed the highest inhibition of TNF-α secretion and excellent kinase selectivity, the metabolic stability of compound **14** was evaluated in human liver microsomes. The suitable metabolic stability was observed on compound **14**, *T*_1/2_ = 96.3 min, CL = 9.64 ml/min/kg, triggering for more in-depth research. However, we have done experiment on AO oxidative metabolism of compound **14** and **P5**. Both compound **14** and **P5** could undergo mono oxidative metabolism in the human liver cytosol incubation system. The addition of aldol oxidase inhibitor raloxifene and menadione significantly inhibited the production of oxidative metabolites. The result showed both compound **14** and **P5** were metabolic substrates of AO oxidase.

## 3. Conclusion

In summary, we designed and synthesised a novel series of 4-aminoquinoline derivatives as RIPK2 inhibitors. According to exploration of SAR, we identified compound **14**, with benzo[*d*]thiazol-5-amine in the back pocket of RIPK2 and para- pyridinyl on C6 position, as a potent inhibitor for further research. Compound **14** exhibited high affinity (IC_50_ = 5.1 ± 1.6 nM) and excellent selectivity to RIPK2 *in vitro*. Besides, compound **14** reduced the secretion of MDP-induced TNF-α indicating cellular anti-inflammatory effect. Meanwhile, the acceptable stability of compound **14** in human liver microsome laid the foundation for in vivo research. Given the mechanism of RIPK2 inhibitors interfering the XIAP binding is still obscure, compound **14** could be utilised as probe molecule in biochemical research. Moreover, considering RIPK2 involved in immune signalling by NOD mediating signal transduction, our findings suggested that compound **14** could serve as a potent inhibitor of RIPK2 to be used in inflammatory and immune treatment research.

## 4. Material and methods

### 4.1. Chemistry

All of the chemical solvents and reagents were analytically pure and commercially available. Thin layer chromatography (TLC) was performed on 0.20 mm silica gel 60 F254 plates (Yantai Xinnuo Chemical, China). ^1^H and ^13^C NMR were recorded on a Bruker Avance 400 spectrometer (Bruker Company, Germany) and Bruker Avance 500 spectrometer (Bruker Company, Germany). Chemical shifts were given in ppm (parts per million). Mass spectra were recorded on a Q-Tof Premier mass spectrometer (Waters Corp., Milford, MA). Waters e2695 series LC system and high-performance liquid chromatography (HPLC) were used to identified the purity of all of the compounds.

General procedure A: To a solution of 6-bromo-4-chloroquinoline (1 eq) in tert-butanol was added unspecified amines (1.1 eq) and HCl (0.01 eq). The suspension was stirred for four hours in oil bath at 80 °C. Then, the reaction mixture was allowed to cool to room temperature and partitioned between ethyl acetate and saturated sodium bicarbonate. The aqueous layer was extracted with ethyl acetate twice and the organic layer was washed with brine, dried over sodium sulphate, and filtered. The filtrate was concentrated *in vacuo* and then purified by column chromatography (0 − 5% MeOH/CH_2_Cl_2_) to provide target product.

General procedure B: A mixture of 6-bromo-4-aminoquinoline derivatives (1 eq), unspecified boronic acid or boronic ester (1.2 eq), Na_2_CO_3_ (2 eq) and Pd(PPh_3_)_4_ (0.1 eq) in solution of 1,4-dioxane and H_2_O (v/v 4:1) was deoxygenated by N_2_ gas for ten minutes, and then stirred for eight hours in oil bath at 80 °C. The reaction mixture was allowed to cool to room temperature and diluted with ethyl acetate, washed with water and brine, dried over sodium sulphate, and filtered. The filtrate was concentrated through rotary evaporator and then purified by column chromatography (0 − 10% MeOH/CH_2_Cl_2_) to obtain target product.

General procedure C: A mixture of 6-bromo-4-aminoquinoline derivatives (1 eq), 2-pyridineboronic acid (3 eq), Pd(OAc)_2_ (0.05 eq), DPPF (0.1 eq), Cs_2_CO_3_ (2 eq) and CuCl (1 eq) in DMF was purged with N_2_ gas for ten minutes and stirred for twelve hours in oil bath at 100 °C. Then the reaction mixture was allowed to cool to room temperature partitioned between ethyl acetate and brine. The organic layer was washed with brine three times, dried over sodium sulphate, and filtered. The filtrate was concentrated and then purified by column chromatography (0 − 12% MeOH/CH2Cl2) to yield target product.

General procedure D: To a solution of unspecified amines (1 eq) in dry DMF was added NaH (1.5 eq) at 0 °C. The suspension was stirred for 10 min before 6-bromo-4-chloroquinoline (1 eq) added. Then, the reaction mixture was stirred for eight hours in oil bath at 40 °C. The mixture was partitioned between ethyl acetate and brine, and then the organic layer was washed with brine three times, dried over sodium sulphate, and filtered. The filtrate was concentrated *in vacuo* and then purified by column chromatography (0 − 5% MeOH/CH_2_Cl_2_) to provide target product.

#### 4.1.1. 6-Bromo-N-phenylquinolin-4-amine (1)

6-bromo-4-chloroquinoline (50.0 mg, 0.21 mmol) and aniline (21.0 mg, 0.23 mmol) were used general procedure A to afford the title compound as a yellow solid (52.0 mg, 84.3% yield). HPLC RT = 2.657 min, 100% purity; Exact mass C_15_H_11_BrN_2_ 298.01, 300.01, found [M + H]^+^ = 299.30, 301.22; ^1^H NMR (400 MHz, DMSO-*d*_6_) *δ* 9.32 (s, 1H), 8.73 (d, *J* = 1.8 Hz, 1H), 8.48 (d, *J* = 5.5 Hz, 1H), 7.90 − 7.78 (m, 2H), 7.45 (t, *J* = 7.6 Hz, 2H), 7.38 (d, *J* = 7.9 Hz, 2H), 7.23 − 7.16 (m, 1H), 6.94 (d, *J* = 5.5 Hz, 1H); ^13^C NMR (151 MHz, DMSO) *δ* 150.7, 148.4, 146.8, 140.3, 133.3, 130.8, 130.0, 125.2, 124.9, 123.3, 121.2, 118.4, 102.3.

#### 4.1.2. 6-Bromo-N-(3-methoxyphenyl)quinolin-4-amine (2)

6-bromo-4-chloroquinoline (50.0 mg, 0.21 mmol) and 3-methoxyaniline (28.0 mg, 0.23 mmol) were used general procedure A to afford the title compound as a yellow solid (55.5 mg, 81.8% yield). HPLC RT = 2.588 min, 98% purity; Exact mass C_16_H_13_BrN_2_O 328.02, 330.02, found [M + H]^+^ = 329.20, 331.10; ^1^H NMR (400 MHz, DMSO-*d*_6_) *δ* 9.25 (s, 1H), 8.70 (d, *J* = 1.9 Hz, 1H), 8.49 (dd, *J* = 5.5, 1.2 Hz, 1H), 7.88 − 7.79 (m, 2H), 7.34 (t, *J* = 8.1 Hz, 1H), 7.02 (dd, *J* = 5.6, 1.3 Hz, 1H), 6.96 (dd, *J* = 7.9, 1.8 Hz, 1H), 6.92 (t, *J* = 2.2 Hz, 1H), 6.75 (dd, *J* = 8.3, 2.5 Hz, 1H), 3.77 (d, *J* = 1.3 Hz, 3H); ^13^C NMR (151 MHz, DMSO) *δ* 160.7, 150.5, 148.3, 146.5, 141.5, 133.4, 130.7, 130.5, 125.3, 121.2, 118.5, 115.1, 110.5, 108.7, 102.8, 55.6.

#### 4.1.3. 6-Bromo-N-(3-isopropoxyphenyl)quinolin-4-amine (3)

6-bromo-4-chloroquinoline (50.0 mg, 0.21 mmol) and 3-isopropoxyaniline (34.3 mg, 0.23 mmol) were used general procedure A to afford the title compound as a yellow solid (58.2 mg, 79.0% yield). HPLC RT = 3.054 min, 100% purity; Exact mass C_18_H_17_BrN_2_O 356.05, 358.05, found [M + H]^+^ = 357.26, 359.24; ^1^H NMR (400 MHz, DMSO-*d*_6_) *δ* 9.06 (s, 1H), 8.70 − 8.62 (m, 1H), 8.48 (d, *J* = 5.3 Hz, 1H), 7.80 (d, *J* = 1.9 Hz, 2H), 7.29 (t, *J* = 8.1 Hz, 1H), 7.01 (d, *J* = 5.3 Hz, 1H), 6.92 (dd, *J* = 7.7, 2.0 Hz, 1H), 6.87 (t, *J* = 2.2 Hz, 1H), 6.69 (dd, *J* = 8.2, 2.4 Hz, 1H), 4.58 (hept, *J* = 6.0 Hz, 1H), 1.27 (d, *J* = 6.0 Hz, 6H); ^13^C NMR (151 MHz, DMSO) *δ* 158.9, 150.0, 148.7, 144.2, 140.8, 134.3, 130.9, 128.6, 125.6, 120.7, 119.0, 115.6, 113.0, 110.9, 102.3, 69.8, 22.3.

#### 4.1.4. 1–(3-((6-Bromoquinolin-4-yl)amino)phenyl)ethan-1-one (4)

6-bromo-4-chloroquinoline (50.0 mg, 0.21 mmol) and 1–(3-aminophenyl)ethan-1-one (30.7 mg, 0.23 mmol) were used general procedure A to afford the title compound as a white solid (47.6 mg, 67.7% yield). HPLC RT = 2.469 min, 100% purity; Exact mass C_17_H_13_BrN_2_O 340.02, 342.02, found [M + H]^+^ = 343.20, 345.12; ^1^H NMR (600 MHz, DMSO-*d*_6_) *δ* 9.45 (s, 1H), 8.73 (s, 1H), 8.52 (d, *J* = 5.4 Hz, 1H), 7.91 (t, *J* = 2.0 Hz, 1H), 7.86 (d, *J* = 1.8 Hz, 2H), 7.76 (dt, *J* = 7.8, 1.3 Hz, 1H), 7.66 (ddd, *J* = 8.0, 2.3, 1.1 Hz, 1H), 7.58 (t, *J* = 7.8 Hz, 1H), 7.03 (d, *J* = 5.5 Hz, 1H), 2.60 (s, 3H); ^13^C NMR (151 MHz, DMSO) *δ* 197.6, 150.4, 147.3, 146.5, 140.5, 138.1, 132.8, 130.5, 129.9, 126.6, 124.7, 123.9, 121.4, 120.9, 118.1, 102.2, 26.8.

#### 4.1.5. 6-Bromo-N-(4-chloro-3-fluorophenyl)quinolin-4-amine (5)

6-bromo-4-chloroquinoline (50.0 mg, 0.21 mmol) and 4-chloro-3-fluoroaniline (33.0 mg, 0.23 mmol) were used general procedure A to afford the title compound as a yellow solid (61.2 mg, 84.4% yield). HPLC RT = 2.784 min, 98% purity; Exact mass C_15_H_9_BrClFN_2_ 349.96, 351.96, found [M + H]^+^ = 351.29, 353.20; ^1^H NMR (600 MHz, DMSO-*d*_6_) *δ* 9.40 (s, 1H), 8.64 (s, 1H), 8.56 (d, *J* = 5.4 Hz, 1H), 7.86 (s, 2H), 7.58 (t, *J* = 8.6 Hz, 1H), 7.40 (dd, *J* = 11.2, 2.5 Hz, 1H), 7.24 (dd, *J* = 8.7, 2.5 Hz, 1H), 7.14 (d, *J* = 5.4 Hz, 1H); ^13^C NMR (151 MHz, DMSO) *δ* 158.3, 156.7, 150.6, 146.7, 146.4, 141.3, 132.8, 131.1, 130.7, 124.7, 121.2, 118.5, 118.4, 118.3, 113.2, 113.1, 109.7, 109.5, 103.5.

#### 4.1.6. 6-Bromo-N-(3,4,5-trimethoxyphenyl)quinolin-4-amine (6)

6-bromo-4-chloroquinoline (5.0 g, 20.6 mmol) and 3,4,5-trimethoxyaniline (4.2 mg, 22.7 mmol) were used general procedure A to afford the title compound as a yellow solid (6.9 g, 86.0% yield). HPLC RT = 2.000 min, 100% purity; Exact mass C_18_H_17_BrN_2_O_3_ 388.04, 390.04, found [M + H]^+^ = 389.20, 391.10; ^1^H NMR (400 MHz, DMSO-*d*_6_) *δ* 9.10 (s, 1H), 8.67 (s, 1H), 8.47 (d, *J* = 5.4 Hz, 1H), 7.88 − 7.76 (m, 2H), 7.00 (d, *J* = 5.4 Hz, 1H), 6.67 (s, 2H), 3.78 (s, 6H), 3.68 (d, *J* = 1.1 Hz, 3H); ^13^C NMR (151 MHz, DMSO) *δ* 153.4, 150.7, 147.8, 146.8, 135.7, 134.4, 132.5, 130.7, 124.6, 120.7, 117.7, 102.1, 100.7, 60.1, 55.9.

#### 4.1.7. 6-Bromo-N-(pyridin-2-yl)quinolin-4-amine (7)

6-bromo-4-chloroquinoline (100.0 mg, 0.41 mmol) and pyridin-2-amine (39.0 mg, 0.41 mmol) were used general procedure D to afford the title compound as a white solid (59.0 mg, 47.7% yield). HPLC RT = 2.417 min, 98% purity; Exact mass C_14_H_10_BrN_3_ 299.01, 301.00, found [M + H]^+^ = 300.28, 302.22; ^1^H NMR (600 MHz, DMSO-*d*_6_) *δ* 9.70 (s, 1H), 8.91 (s, 1H), 8.68 (dd, *J* = 5.4, 1.3 Hz, 1H), 8.46 (d, *J* = 5.4 Hz, 1H), 8.34 (ddd, *J* = 4.9, 2.0, 0.8 Hz, 1H), 7.90 − 7.83 (m, 2H), 7.77 (ddd, *J* = 8.4, 7.2, 2.0 Hz, 1H), 7.50 − 7.40 (m, 1H), 7.02 (ddd, *J* = 7.2, 4.9, 1.0 Hz, 1H); ^13^C NMR (151 MHz, DMSO) *δ* 154.7, 150.7, 147.2, 146.7, 144.0, 138.0, 132.4, 131.0, 124.7, 121.3, 118.4, 117.4, 113.9, 107.1.

#### 4.1.8. 6-Bromo-N-(pyridin-3-yl)quinolin-4-amine (8)

6-bromo-4-chloroquinoline (100.0 mg, 0.41 mmol) and pyridin-3-amine (38.9 mg, 0.41 mmol) were used general procedure D to afford the title compound as a brown solid (49.5 mg, 40.0% yield). HPLC RT = 2.027 min, 98% purity; Exact mass C_14_H_10_BrN_3_ 299.01, 301.00, found [M + H]^+^ = 300.28, 302.23; ^1^H NMR (400 MHz, DMSO-*d*_6_) *δ* 9.21 (s, 1H), 8.67 (t, *J* = 1.4 Hz, 1H), 8.61 (d, *J* = 2.6 Hz, 1H), 8.51 (d, *J* = 5.3 Hz, 1H), 8.35 (dd, *J* = 4.7, 1.5 Hz, 1H), 7.84 (s, 1H), 7.84 (s, 1H), 7.80 (ddd, *J* = 8.2, 2.7, 1.5 Hz, 1H), 7.45 (dd, *J* = 8.2, 4.7 Hz, 1H), 6.98 (d, *J* = 5.4 Hz, 1H); ^13^C NMR (151 MHz, DMSO) *δ* 151.1, 147.4, 146.7, 144.6, 144.0, 137.1, 132.5, 131.3, 129.1, 124.6, 124.1, 121.2, 118.0, 102.3.

#### 4.1.9. 6-Bromo-N-(pyridin-4-yl)quinolin-4-amine (9)

6-bromo-4-chloroquinoline (100.0 mg, 0.41 mmol) and pyridin-4-amine (38.8 mg, 0.41 mmol) were used general procedure D to afford the title compound as a yellow solid (51.2 mg, 41.4% yield). HPLC RT = 1.868 min, 95% purity; Exact mass C_14_H_10_BrN_3_ 299.01, 301.00, found [M + H]^+^ = 300.28, 302.20; ^1^H NMR (400 MHz, DMSO-*d*_6_) *δ* 9.49 (s, 1H), 8.69 (d, *J* = 5.2 Hz, 1H), 8.58 (d, *J* = 1.9 Hz 1H), 8.42 (s, 1H), 8.40 (s, 1H), 7.93 − 7.85 (m, 2H), 7.43 (d, *J* = 5.2 Hz, 1H), 7.30 (s, 1H), 7.29 (s, 1H); ^13^C NMR (151 MHz, DMSO) *δ* 151.3, 150.2, 148.6, 147.7, 144.0, 132.7, 131.5, 124.8, 122.4, 118.7, 112.9, 107.1.

#### 4.1.10. N-(6-bromoquinolin-4-yl)benzo[d]thiazol-5-amine (10)

6-bromo-4-chloroquinoline (5.0 g, 20.6 mmol) and benzo[d]thiazol-5-amine (3.4 g, 22.7 mmol) were used general procedure A to afford the title compound as a yellow solid (6.2 g, 84.4% yield). HPLC RT = 2.510 min, 100% purity; Exact mass C_16_H_10_BrN_3_S 354.98, 356.98, found [M + H]^+^ = 356.25, 358.20; ^1^H NMR (400 MHz, DMSO-*d*_6_) *δ* 9.72 (s, 1H), 9.45 (s, 1H), 8.80 (d, *J* = 2.1 Hz, 1H), 8.51 (d, *J* = 5.7 Hz, 1H), 8.24 (d, *J* = 8.6 Hz, 1H), 8.07 (d, *J* = 2.1 Hz, 1H), 7.93 (dd, *J* = 9.0, 2.0 Hz, 1H), 7.87 (d, *J* = 9.0 Hz, 1H), 7.55 (dd, *J* = 8.6, 2.1 Hz, 1H), 7.01 (d, *J* = 5.7 Hz, 1H); ^13^C NMR (151 MHz, DMSO) *δ* 158.4, 154.3, 153.9, 143.0, 137.2, 136.6, 135.5, 132.6, 126.4, 123.9, 123.1, 122.3, 119.9, 119.6, 118.7, 100.6.

#### 4.1.11. N-(6-bromoquinolin-4-yl)benzo[d]thiazol-6-amine (11)

6-bromo-4-chloroquinoline (50.0 mg, 0.21 mmol) and benzo[d]thiazol-6-amine (34.3 mg, 0.23 mmol) were used general procedure A to afford the title compound as a yellow solid (45.0 mg, 61.3% yield). HPLC RT = 2.483 min, 100% purity; Exact mass C_16_H_10_BrN_3_S 354.98, 356.98, found [M + H]^+^ = 356.23, 358.18; ^1^H NMR (400 MHz, DMSO-*d*_6_) *δ* 9.59 (s, 1H), 9.35 (s, 1H), 8.77 (d, *J* = 2.1 Hz, 1H), 8.51 (d, *J* = 5.6 Hz, 1H), 8.18 (d, *J* = 2.2 Hz, 1H), 8.14 (d, *J* = 8.7 Hz, 1H), 7.90 (dd, *J* = 9.0, 2.0 Hz, 1H), 7.86 (d, *J* = 9.0 Hz, 1H), 7.56 (dd, *J* = 8.7, 2.2 Hz, 1H), 7.02 (d, *J* = 5.6 Hz, 1H); ^13^C NMR (151 MHz, DMSO) *δ* 155.6, 150.2, 150.0, 145.9, 137.5, 134.8, 133.1, 129.9, 129.6, 124.8, 123.7, 122.3, 120.7, 118.2, 115.9, 102.1.

#### 4.1.12. 6-Bromo-N-(1H-indazol-6-yl)quinolin-4-amine (12)

6-bromo-4-chloroquinoline (50.0 mg, 0.21 mmol) and 1H-indazol-6-amine (30.0 mg, 0.23 mmol) were used general procedure A to afford the title compound as a yellow solid (43.6 mg, 62.3% yield). HPLC RT = 2.481 min, 98% purity; Exact mass C_16_H_11_BrN_4_ 338.02, 340.01, found [M + H]^+^ = 339.31, 341.22; ^1^H NMR (400 MHz, DMSO-*d*_6_) *δ* 12.97 (s, 1H), 9.22 (s, 1H), 8.70 (t, *J* = 1.4 Hz, 1H), 8.49 (d, *J* = 5.3 Hz, 1H), 8.05 (t, *J* = 1.3 Hz, 1H), 7.83 (s, 2H), 7.83 (s, 1H), 7.79 (d, *J* = 8.6 Hz, 1H), 7.46 − 7.42 (m, 1H), 7.15 (dd, *J* = 8.6, 1.8 Hz, 1H), 7.03 (d, *J* = 5.3 Hz, 1H); ^13^C NMR (151 MHz, DMSO) *δ* 151.0, 147.4, 147.3, 140.7, 138.4, 133.6, 132.4, 131.2, 124.6, 121.3, 121.1, 120.0, 117.8, 117.0, 102.4, 102.3.

#### 4.1.13. 6-Bromo-N-(1H-indol-5-yl)quinolin-4-amine (13)

6-bromo-4-chloroquinoline (50.0 mg, 0.21 mmol) and 1H-indol-5-amine (30.0 mg, 0.23 mmol) were used general procedure A to afford the title compound as a yellow solid (42.0 mg, 60.2% yield). HPLC RT = 2.781 min, 98% purity; Exact mass C_17_H_12_BrN_3_ 337.02, 339.02, found [M + H]^+^ = 338.16, 340.20; ^1^H NMR (400 MHz, DMSO-*d*_6_) *δ* 11.20 (s, 1H), 9.11 (s, 1H), 8.74 (d, *J* = 2.0 Hz, 1H), 8.35 (d, *J* = 5.5 Hz, 1H), 7.80 (dd, *J* = 9.0, 1.9 Hz, 1H), 7.77 (d, *J* = 8.9 Hz, 1H), 7.51 − 7.46 (m, 2H), 7.40 (t, *J* = 2.8 Hz, 1H), 7.06 (dd, *J* = 8.5, 2.1 Hz, 1H), 6.61 (d, *J* = 5.5 Hz, 1H), 6.45 (t, *J* = 2.5 Hz, 1H); ^13^C NMR (151 MHz, DMSO) *δ* 150.5, 149.7, 146.7, 134.0, 132.4, 130.8, 130.6, 128.2, 126.4, 124.6, 120.3, 119.0, 117.3, 116.1, 112.2, 101.2, 100.6.

#### 4.1.14. N-(6-(pyridin-4-yl)quinolin-4-yl)benzo[d]thiazol-5-amine (14)

N-(6-bromoquinolin-4-yl)benzo[d]thiazol-5-amine (**10**) (50.0 mg, 0.14 mmol) and pyridin-4-ylboronic acid (20.6 mg, 0.17 mmol) were used general procedure B to afford the title compound as a white solid (31.0 mg, 62.3% yield). HPLC RT = 2.040 min, 100% purity; Exact mass C_21_H_14_N_4_S 354.09, found [M + H]^+^ = 355.42; ^1^H NMR (500 MHz, DMSO-*d*_6_) *δ* 9.44 (s, 1H), 8.92 (d, *J* = 2.1 Hz, 1H), 8.74 − 8.69 (m, 2H), 8.50 (d, *J* = 5.4 Hz, 1H), 8.23 (d, *J* = 8.5 Hz, 1H), 8.17 (dd, *J* = 8.7, 2.0 Hz, 1H), 8.08 (d, *J* = 2.1 Hz, 1H), 8.01 (d, *J* = 8.8 Hz, 1H), 7.98 − 7.93 (m, 2H), 7.58 (dd, *J* = 8.6, 2.2 Hz, 1H), 7.02 (d, *J* = 5.4 Hz, 1H); ^13^C NMR (126 MHz, DMSO) *δ* 157.4, 154.2, 151.2, 150.3, 148.7, 148.6, 146.5, 138.9, 133.2, 129.8, 129.2, 127.7, 123.2, 121.6, 121.4, 120.7, 119.7, 116.5, 101.7.

#### 4.1.15. N-(6-(pyridin-3-yl)quinolin-4-yl)benzo[d]thiazol-5-amine (15)

N-(6-bromoquinolin-4-yl)benzo[d]thiazol-5-amine (**10**) (50.0 mg, 0.14 mmol) and pyridin-3-ylboronic acid (20.5 mg, 0.17 mmol) were used general procedure B to afford the title compound as a yellow solid (35.0 mg, 70.4% yield). HPLC RT = 2.071 min, 100% purity; Exact mass C_21_H_14_N_4_S 354.09, found [M + H]^+^ = 355.42; ^1^H NMR (400 MHz, DMSO-*d*_6_) *δ* 9.45 (s, 1H), 9.17 (s, 1H), 8.85 (s, 1H), 8.63 (d, *J* = 4.7 Hz, 1H), 8.50 (d, *J* = 5.4 Hz, 1H), 8.31 (d, *J* = 8.0 Hz, 1H), 8.23 (d, *J* = 8.6 Hz, 1H), 8.13 (d, *J* = 8.8 Hz, 1H), 8.08 (s, 1H), 8.01 (d, *J* = 8.7 Hz, 1H), 7.57 (dd, *J* = 8.3, 5.8 Hz, 2H), 7.04 (d, *J* = 5.4 Hz, 1H); ^13^C NMR (126 MHz, DMSO) *δ* 157.5, 154.2, 150.8, 148.6, 148.4, 148.1, 148.1, 138.9, 135.1, 134.3, 133.4, 129.7, 129.1, 128.2, 123.9, 123.2, 121.5, 120.2, 119.7, 116.4, 101.6.

#### 4.1.16. N-(6-(pyridin-2-yl)quinolin-4-yl)benzo[d]thiazol-5-amine (16)

N-(6-bromoquinolin-4-yl)benzo[d]thiazol-5-amine (**10**) (50.0 mg, 0.14 mmol) and pyridin-2-ylboronic acid (51.8 mg, 0.42 mmol) were used general procedure C to afford the title compound as a yellow solid (21.5 mg, 43.2% yield). HPLC RT = 2.255 min, 100% purity; Exact mass C_21_H_14_N_4_S 354.09, found [M + H]^+^ = 355.42; ^1^H NMR (500 MHz, DMSO-*d*_6_) *δ* 9.45 (s, 1H), 9.18 (d, *J* = 2.0 Hz, 1H), 8.53 (dd, *J* = 8.9, 1.9 Hz, 1H), 8.50 (d, *J* = 5.5 Hz, 1H), 8.29 − 8.25 (m, 1H), 8.24 (d, *J* = 8.5 Hz, 1H), 8.10 (d, *J* = 2.1 Hz, 1H), 8.02 − 7.95 (m, 2H), 7.60 (dd, *J* = 8.6, 2.1 Hz, 1H), 7.42 (ddd, *J* = 7.5, 4.8, 1.0 Hz, 1H), 7.01 (d, *J* = 5.5 Hz, 1H); ^13^C NMR (126 MHz, DMSO) *δ* 157.5, 155.5, 154.2, 150.0, 149.6, 149.5, 147.7, 138.8, 137.3, 135.3, 129.3, 128.3, 128.3, 123.2, 122.8, 121.7, 120.8, 120.4, 119.4, 116.6, 101.7.

#### 4.1.17. N-(6-(pyrimidin-5-yl)quinolin-4-yl)benzo[d]thiazol-5-amine (17)

N-(6-bromoquinolin-4-yl)benzo[d]thiazol-5-amine (**10**) (50.0 mg, 0.14 mmol) and pyrimidin-5-ylboronic acid (20.8 mg, 0.17 mmol) were used general procedure B to afford the title compound as a yellow solid (30.2 mg, 60.5% yield). HPLC RT = 2.302 min, 100% purity; Exact mass C_20_H_13_N_5_S 355.09, found [M + H]^+^ = 356.43; ^1^H NMR (400 MHz, DMSO-*d*_6_) *δ* 9.46 (s, 1H), 9.40 (s, 2H), 9.24 (s, 1H), 8.96 (d, *J* = 2.0 Hz, 1H), 8.51 (d, *J* = 5.5 Hz, 1H), 8.28 − 8.19 (m, 2H), 8.09 (d, *J* = 2.1 Hz, 1H), 8.03 (d, *J* = 8.8 Hz, 1H), 7.58 (dd, *J* = 8.6, 2.1 Hz, 1H), 7.03 (d, *J* = 5.5 Hz, 1H); ^13^C NMR (126 MHz, DMSO) *δ* 157.6, 157.4, 154.9, 154.2, 150.6, 148.9, 147.6, 138.5, 132.7, 130.1, 129.4, 129.3, 128.0, 123.3, 121.7, 120.8, 119.5, 116.7, 101.5.

#### 4.1.18. N-(6-phenylquinolin-4-yl)benzo[d]thiazol-5-amine (18)

N-(6-bromoquinolin-4-yl)benzo[d]thiazol-5-amine (**10**) (50.0 mg, 0.14 mmol) and phenylboronic acid (20.5 mg, 0.17 mmol) were used general procedure B to afford the title compound as a yellow solid (38.1 mg, 76.8% yield). HPLC RT = 2.921 min, 100% purity; Exact mass C_22_H_15_N_3_S 353.10, found [M + H]^+^ = 354.42; ^1^H NMR (400 MHz, DMSO-*d*_6_) *δ* 9.44 (s, 1H), 8.76 (d, *J* = 2.0 Hz, 1H), 8.48 (d, *J* = 5.3 Hz, 1H), 8.22 (d, *J* = 8.6 Hz, 1H), 8.06 (dd, *J* = 6.3, 2.1 Hz, 2H), 7.98 (d, *J* = 8.7 Hz, 1H), 7.92 (d, *J* = 7.6 Hz, 2H), 7.60 − 7.50 (m, 3H), 7.42 (t, *J* = 7.4 Hz, 1H), 7.03 (d, *J* = 5.3 Hz, 1H); ^13^C NMR (126 MHz, DMSO) *δ* 157.3, 154.2, 150.4, 148.2, 147.9, 139.7, 139.1, 136.5, 129.5, 128.9, 128.8, 128.3, 127.6, 127.1, 123.1, 121.5, 119.8, 119.7, 116.2, 101.7.

#### 4.1.19. N-(6–(1-methyl-1H-pyrazol-4-yl)quinolin-4-yl)benzo[d]thiazol-5-amine (19)

N-(6-bromoquinolin-4-yl)benzo[d]thiazol-5-amine (**10**) (50.0 mg, 0.14 mmol) and 1-methyl-4–(4,4,5,5-tetramethyl-1,3,2-dioxaborolan-2-yl)-1H-pyrazole (35.0 mg, 0.17 mmol) were used general procedure B to afford the title compound as a white solid (26.2 mg, 52.2% yield). HPLC RT = 2.457 min, 100% purity; Exact mass C_20_H_15_N_5_S 357.10, found [M + H]^+^ = 358.38; ^1^H NMR (400 MHz, DMSO-*d*_6_) *δ* 9.44 (s, 1H), 9.18 (s, 1H), 8.62 (d, *J* = 1.9 Hz, 1H), 8.41 (d, *J* = 5.3 Hz, 1H), 8.28 (s, 1H), 8.22 (d, *J* = 8.6 Hz, 1H), 8.07 (d, *J* = 2.6 Hz, 2H), 7.94 (dd, *J* = 8.7, 1.8 Hz, 1H), 7.87 (d, *J* = 8.7 Hz, 1H), 7.57 (dd, *J* = 8.6, 2.1 Hz, 1H), 6.97 (d, *J* = 5.3 Hz, 1H), 3.92 (s, 3H); ^13^C NMR (126 MHz, DMSO) *δ* 157.3, 154.2, 149.7, 147.6, 147.3, 139.2, 136.4, 129.5, 129.2, 128.8, 128.2, 127.4, 123.1, 121.9, 121.5, 120.0, 116.7, 116.1, 101.6, 38.7.

#### 4.1.20. N-(6-(1H-pyrazol-4-yl)quinolin-4-yl)benzo[d]thiazol-5-amine (20)

N-(6-bromoquinolin-4-yl)benzo[d]thiazol-5-amine (**10**) (50.0 mg, 0.14 mmol) and 4–(4,4,5,5-tetramethyl-1,3,2-dioxaborolan-2-yl)-1H-pyrazole (32.7 mg, 0.17 mmol) were used general procedure B to afford the title compound as a yellow solid (24.7 mg, 51.2% yield). HPLC RT = 2.341 min, 100% purity; Exact mass C_19_H_13_N_5_S 343.09, found [M + H]^+^ = 344.33; ^1^H NMR (400 MHz, DMSO-*d*_6_) *δ* 13.08 (s, 1H), 9.44 (s, 1H), 9.21 (s, 1H), 8.66 (d, *J* = 1.9 Hz, 1H), 8.41 (d, *J* = 5.3 Hz, 1H), 8.22 (d, *J* = 8.6 Hz, 1H), 8.07 (d, *J* = 2.1 Hz, 1H), 8.01 (dd, *J* = 8.7, 1.8 Hz, 1H), 7.87 (d, *J* = 8.7 Hz, 1H), 7.57 (dd, *J* = 8.6, 2.1 Hz, 1H), 6.97 (d, *J* = 5.3 Hz, 1H); ^13^C NMR (151 MHz, DMSO) *δ* 157.4, 154.2, 149.6, 147.7, 147.2, 139.2, 136.6, 129.6, 129.3, 128.8, 127.7, 126.1, 123.1, 121.5, 121.1, 120.0, 116.8, 116.2, 101.5.

#### 4.1.21. N-(6–(3-methyl-1H-pyrazol-4-yl)quinolin-4-yl)benzo[d]thiazol-5-amine (21)

N-(6-bromoquinolin-4-yl)benzo[d]thiazol-5-amine (**10**) (50.0 mg, 0.14 mmol) and 3-methyl-4–(4,4,5,5-tetramethyl-1,3,2-dioxaborolan-2-yl)-1H-pyrazole (35.1 mg, 0.17 mmol) were used general procedure B to afford the title compound as a yellow solid (30.0 mg, 59.8% yield). HPLC RT = 2.318 min, 100% purity; Exact mass C_20_H_15_N_5_S 357.10, found [M + H]^+^ = 358.36; ^1^H NMR (400 MHz, DMSO-*d*_6_) *δ* 12.77 (s, 1H), 9.43 (s, 1H), 9.22 (s, 1H), 8.44 (d, *J* = 5.3 Hz, 2H), 8.20 (d, *J* = 8.7 Hz, 1H), 8.04 (d, *J* = 2.1 Hz, 1H), 7.91 (d, *J* = 8.7 Hz, 1H), 7.85 (dd, *J* = 8.7, 1.8 Hz, 1H), 7.55 (dd, *J* = 8.6, 2.1 Hz, 1H), 7.00 (d, *J* = 5.3 Hz, 1H), 2.47 (s, 4H); ^13^C NMR (126 MHz, DMSO) *δ* 157.3, 154.2, 149.7, 147.8, 146.9, 139.4, 138.7, 135.4, 130.6, 129.2, 129.1, 128.7, 123.1, 121.4, 120.0, 118.8, 118.2, 116.0, 101.9, 26.8.

#### 4.1.22. N-(6–(1,4-dimethyl-1H-pyrazol-5-yl)quinolin-4-yl)benzo[d]thiazol-5-amine (22)

N-(6-bromoquinolin-4-yl)benzo[d]thiazol-5-amine (**10**) (50.0 mg, 0.14 mmol) and 1,4-dimethyl-5–(4,4,5,5-tetramethyl-1,3,2-dioxaborolan-2-yl)-1H-pyrazole (37.2 mg, 0.17 mmol) were used general procedure B to afford the title compound as a white solid (32.5 mg, 62.3% yield). HPLC RT = 2.472 min, 95% purity; Exact mass C_21_H_17_N_5_S 371.12, found [M + H]^+^ = 372.40; ^1^H NMR (400 MHz, DMSO-*d*_6_) *δ* 9.43 (s, 1H), 9.27 (s, 1H), 8.52 (d, *J* = 5.4 Hz, 1H), 8.50 − 8.47 (m, 1H), 8.20 (d, *J* = 8.6 Hz, 1H), 8.05 (d, *J* = 2.1 Hz, 1H), 8.00 (d, *J* = 8.6 Hz, 1H), 7.74 (dd, *J* = 8.6, 1.8 Hz, 1H), 7.55 (dd, *J* = 8.6, 2.1 Hz, 1H), 7.40 (s, 1H), 7.04 (d, *J* = 5.4 Hz, 1H), 3.79 (s, 3H), 2.03 (s, 3H); ^13^C NMR (126 MHz, DMSO) *δ* 157.3, 154.2, 151.3, 148.2, 148.0, 139.8, 139.0, 138.3, 130.4, 129.5, 128.9, 126.3, 123.3, 123.1, 121.5, 119.5, 116.3, 114.1, 101.8, 37.3, 9.0.

#### 4.1.23. N-(6–(1,3-dimethyl-1H-pyrazol-4-yl)quinolin-4-yl)benzo[d]thiazol-5-amine (23)

N-(6-bromoquinolin-4-yl)benzo[d]thiazol-5-amine (**10**) (50.0 mg, 0.14 mmol) and 1,3-dimethyl-4–(4,4,5,5-tetramethyl-1,3,2-dioxaborolan-2-yl)-1H-pyrazole (37.0 mg, 0.17 mmol) were used general procedure B to afford the title compound as a yellow solid (28.8 mg, 55.2% yield). HPLC RT = 2.447 min, 100% purity; Exact mass C_21_H_17_N_5_S 371.12, found [M + H]^+^ = 372.38; ^1^H NMR (400 MHz, DMSO-*d*_6_) *δ* 9.43 (s, 1H), 9.20 (s, 1H), 8.48 − 8.42 (m, 1H), 8.39 (s, 1H), 8.20 (d, *J* = 8.5 Hz, 1H), 8.03 (s, 2H), 7.91 (d, *J* = 8.6 Hz, 1H), 7.80 (d, *J* = 8.7 Hz, 1H), 7.55 (d, *J* = 8.6 Hz, 1H), 7.00 (d, *J* = 5.3 Hz, 1H), 3.83 (s, 3H), 2.39 (s, 3H); ^13^C NMR (151 MHz, DMSO) *δ* 157.3, 154.2, 149.8, 147.8, 147.0, 144.4, 139.5, 130.3, 130.1, 129.2, 129.2, 128.6, 123.1, 121.3, 120.0, 119.6, 119.1, 115.9, 102.0, 38.3, 13.2.

#### 4.1.24. N-(6–(4-methylthiophen-3-yl)quinolin-4-yl)benzo[d]thiazol-5-amine (24)

N-(6-bromoquinolin-4-yl)benzo[d]thiazol-5-amine (**10**) (50.0 mg, 0.14 mmol) and (4-methylthiophen-3-yl)boronic acid (24.0 mg, 0.17 mmol) were used general procedure B to afford the title compound as a yellow solid (33.2 mg, 63.3% yield). HPLC RT = 2.971 min, 100% purity; Exact mass C_21_H_15_N_3_S_2_ 373.07, found [M + H]^+^ = 374.41; ^1^H NMR (400 MHz, DMSO-*d*_6_) *δ* 9.43 (s, 1H), 8.52 − 8.46 (m, 2H), 8.20 (d, *J* = 8.5 Hz, 1H), 8.04 (d, *J* = 2.1 Hz, 1H), 7.95 (d, *J* = 8.6 Hz, 1H), 7.81 (dd, *J* = 8.6, 1.8 Hz, 1H), 7.64 (d, *J* = 3.3 Hz, 1H), 7.55 (dd, *J* = 8.6, 2.1 Hz, 1H), 7.35 (d, *J* = 3.2 Hz, 1H), 7.03 (d, *J* = 5.4 Hz, 1H), 2.32 (s, 3H); ^13^C NMR (126 MHz, DMSO) *δ* 157.3, 154.2, 150.0, 148.3, 147.1, 142.1, 139.1, 135.7, 133.2, 130.3, 128.9, 128.5, 124.2, 123.1, 122.9, 121.5, 121.4, 119.5, 116.1, 101.7, 15.4.

#### 4.1.25. N-(6-(1H-indol-5-yl)quinolin-4-yl)benzo[d]thiazol-5-amine (25)

N-(6-bromoquinolin-4-yl)benzo[d]thiazol-5-amine (**10**) (50.0 mg, 0.14 mmol) and 5–(4,4,5,5-tetramethyl-1,3,2-dioxaborolan-2-yl)-1H-indole (40.9 mg, 0.17 mmol) were used general procedure B to afford the title compound as a yellow solid (38.0 mg, 69.0% yield). HPLC RT = 2.884 min, 100% purity; Exact mass C_24_H_16_N_4_S 392.11, found [M + H]^+^ = 393.49; ^1^H NMR (400 MHz, DMSO-*d*_6_) *δ* 11.24 (s, 1H), 9.44 (s, 1H), 8.73 (d, *J* = 2.0 Hz, 1H), 8.45 (d, *J* = 5.3 Hz, 1H), 8.21 (d, *J* = 8.6 Hz, 1H), 8.12 − 8.05 (m, 3H), 7.96 (d, *J* = 8.8 Hz, 1H), 7.67 (dd, *J* = 8.5, 1.8 Hz, 1H), 7.59 (dd, *J* = 8.6, 2.1 Hz, 1H), 7.54 (d, *J* = 8.5 Hz, 1H), 7.41 (t, *J* = 2.7 Hz, 1H), 7.03 (d, *J* = 5.3 Hz, 1H), 6.53 (t, *J* = 2.4 Hz, 1H); ^13^C NMR (126 MHz, DMSO) *δ* 157.3, 154.2, 150.0, 147.9, 147.6, 139.4, 138.3, 135.6, 130.8, 129.4, 128.8, 128.6, 128.3, 126.2, 123.0, 121.4, 120.8, 120.1, 118.9, 118.7, 116.0, 111.8, 101.7, 101.6.

#### 4.1.26. N-(6-(1H-indol-4-yl)quinolin-4-yl)benzo[d]thiazol-5-amine (26)

N-(6-bromoquinolin-4-yl)benzo[d]thiazol-5-amine (**10**) (50.0 mg, 0.14 mmol) and 4–(4,4,5,5-tetramethyl-1,3,2-dioxaborolan-2-yl)-1H-indole (40.6 mg, 0.17 mmol) were used general procedure B to afford the title compound as a yellow solid (36.8 mg, 66.8% yield). HPLC RT = 2.819 min, 100% purity; Exact mass C_24_H_16_N_4_S 392.11, found [M + H]^+^ = 393.47; ^1^H NMR (400 MHz, DMSO-*d*_6_) *δ* 11.35 (s, 1H), 9.43 (s, 1H), 8.71 (s, 1H), 8.51 (d, *J* = 5.4 Hz, 1H), 8.20 (d, *J* = 8.6 Hz, 1H), 8.10 − 8.05 (m, 2H), 8.02 (d, *J* = 8.7 Hz, 1H), 7.57 (dd, *J* = 8.5, 2.1 Hz, 1H), 7.48 (d, *J* = 7.8 Hz, 1H), 7.44 (t, *J* = 2.8 Hz, 1H), 7.30 (d, *J* = 7.1 Hz, 1H), 7.25 (t, *J* = 7.6 Hz, 1H), 7.06 (d, *J* = 5.4 Hz, 1H), 6.64 (t, *J* = 2.5 Hz, 1H); ^13^C NMR (126 MHz, DMSO) *δ* 157.3, 154.2, 149.9, 148.4, 147.2, 139.3, 137.9, 136.4, 132.6, 130.4, 128.7, 128.6, 126.0, 125.8, 123.1, 121.4, 121.3, 121.2, 120.0, 119.3, 116.0, 111.1, 101.9, 100.3.

#### 4.1.27. N-(6-(1H-indazol-4-yl)quinolin-4-yl)benzo[d]thiazol-5-amine (27)

N-(6-bromoquinolin-4-yl)benzo[d]thiazol-5-amine (**10**) (50.0 mg, 0.14 mmol) and 4–(4,4,5,5-tetramethyl-1,3,2-dioxaborolan-2-yl)-1H-indazole (41.3 mg, 0.17 mmol) were used general procedure B to afford the title compound as a yellow solid (34.5 mg, 62.5% yield). HPLC RT = 2.588 min, 100% purity; Exact mass C_23_H_15_N_5_S 393.10, found [M + H]^+^ = 394.39; ^1^H NMR (400 MHz, DMSO-*d*_6_) *δ* 13.32 (s, 1H), 9.44 (s, 1H), 8.79 (s, 1H), 8.52 (d, *J* = 5.4 Hz, 1H), 8.30 (s, 1H), 8.21 (d, *J* = 8.6 Hz, 1H), 8.16 − 8.10 (m, 1H), 8.08 − 8.02 (m, 2H), 7.62 (d, *J* = 8.2 Hz, 1H), 7.57 (dd, *J* = 8.6, 2.0 Hz, 1H), 7.52 (t, *J* = 7.6 Hz, 1H), 7.44 (d, *J* = 7.0 Hz, 1H), 7.06 (d, *J* = 5.4 Hz, 1H); ^13^C NMR (126 MHz, DMSO) *δ* 157.3, 154.2, 150.5, 148.5, 147.8, 140.5, 139.2, 136.1, 133.7, 132.9, 130.0, 129.3, 128.9, 126.3, 123.1, 121.5, 121.4, 121.2, 120.2, 120.0, 116.2, 109.6, 101.9.

#### 4.1.28. N-(6-(1H-pyrrolo[2,3-b]pyridin-4-yl)quinolin-4-yl)benzo[d]thiazol-5-amine (28)

N-(6-bromoquinolin-4-yl)benzo[d]thiazol-5-amine (**10**) (50.0 mg, 0.14 mmol) and 4–(4,4,5,5-tetramethyl-1,3,2-dioxaborolan-2-yl)-1H-pyrrolo[2,3-b]pyridine (41.0 mg, 0.17 mmol) were used general procedure B to afford the title compound as a yellow solid (29.0 mg, 52.5% yield). HPLC RT = 2.200 min, 100% purity; Exact mass C_23_H_15_N_5_S 393.10, found [M + H]^+^ = 394.30; ^1^H NMR (400 MHz, DMSO-*d*_6_) *δ* 11.88 (s, 1H), 9.43 (s, 1H), 9.38 (s, 1H), 8.82 (d, *J* = 1.9 Hz, 1H), 8.53 (d, *J* = 5.3 Hz, 1H), 8.36 (d, *J* = 4.9 Hz, 1H), 8.20 (d, *J* = 8.7 Hz, 1H), 8.13 (dd, *J* = 8.7, 1.8 Hz, 1H), 8.06 (dd, *J* = 5.4, 3.4 Hz, 2H), 7.62 − 7.53 (m, 2H), 7.40 (d, *J* = 5.0 Hz, 1H), 7.08 (d, *J* = 5.3 Hz, 1H), 6.72 (dd, *J* = 3.6, 1.8 Hz, 1H); ^13^C NMR (151 MHz, DMSO) *δ* 157.3, 154.2, 151.3, 149.2, 148.7, 148.2, 142.9, 140.1, 139.3, 135.0, 129.8, 129.5, 128.7, 126.8, 123.1, 121.9, 121.3, 120.1, 117.5, 116.0, 114.8, 102.1, 99.2.

#### 4.1.29. N-(6-(isoquinolin-4-yl)quinolin-4-yl)benzo[d]thiazol-5-amine (29)

N-(6-bromoquinolin-4-yl)benzo[d]thiazol-5-amine (**10**) (50.0 mg, 0.14 mmol) and isoquinolin-4-ylboronic acid (29.0 mg, 0.17 mmol) were used general procedure B to afford the title compound as a yellow solid (39.9 mg, 70.3% yield). HPLC RT = 2.213 min, 100% purity; Exact mass C_25_H_16_N_4_S 404.11, found [M + H]^+^ = 405.30; ^1^H NMR (400 MHz, DMSO-*d*_6_) *δ* 9.43 (s, 1H), 9.41 (s, 1H), 8.69 (d, *J* = 1.9 Hz, 1H), 8.63 (s, 1H), 8.56 (d, *J* = 5.4 Hz, 1H), 8.26 (d, *J* = 8.1 Hz, 1H), 8.19 (d, *J* = 8.6 Hz, 1H), 8.08 (d, *J* = 8.6 Hz, 1H), 8.05 (d, *J* = 2.1 Hz, 1H), 7.97 − 7.88 (m, 2H), 7.85 − 7.79 (m, 1H), 7.76 (t, *J* = 7.4 Hz, 1H), 7.55 (dd, *J* = 8.6, 2.1 Hz, 1H), 7.09 (d, *J* = 5.4 Hz, 1H); ^13^C NMR (126 MHz, DMSO) *δ* 157.3, 154.1, 152.2, 150.6, 148.4, 147.6, 143.0, 138.9, 133.3, 133.1, 132.2, 131.5, 131.3, 129.0, 128.8, 128.2, 128.0, 127.6, 124.2, 123.5, 123.1, 121.4, 119.7, 116.1, 101.8.

#### 4.1.30. N-([3,6'-biquinolin]-4'-yl)benzo[d]thiazol-5-amine (30)

N-(6-bromoquinolin-4-yl)benzo[d]thiazol-5-amine (**10**) (50.0 mg, 0.14 mmol) and quinolin-3-ylboronic acid (29.3 mg, 0.17 mmol) were used general procedure B to afford the title compound as a yellow solid (37.5 mg, 66.1% yield). HPLC RT = 2.387 min, 100% purity; Exact mass C_25_H_16_N_4_S 404.11, found [M + H]^+^ = 405.30; ^1^H NMR (400 MHz, DMSO-*d*_6_) *δ* 9.55 (d, *J* = 2.4 Hz, 1H), 9.45 (s, 1H), 9.00 (d, *J* = 2.1 Hz, 1H), 8.86 (d, *J* = 2.4 Hz, 1H), 8.51 (d, *J* = 5.4 Hz, 1H), 8.28 (dd, *J* = 8.8, 1.9 Hz, 1H), 8.24 (d, *J* = 8.6 Hz, 1H), 8.10 (dd, *J* = 5.7, 3.2 Hz, 3H), 8.05 (d, *J* = 8.8 Hz, 1H), 7.82 − 7.76 (m, 1H), 7.67 (t, *J* = 7.5 Hz, 1H), 7.60 (dd, *J* = 8.6, 2.1 Hz, 1H), 7.05 (d, *J* = 5.4 Hz, 1H); ^13^C NMR (126 MHz, DMSO) *δ* 157.5, 154.2, 150.9, 149.8, 148.5, 148.2, 146.9, 139.0, 133.3, 133.1, 132.3, 129.9, 129.7, 129.1, 128.8, 128.4, 127.7, 127.2, 123.2, 121.6, 120.5, 119.9, 116.5, 101.7.

#### 4.1.31. 6-(Pyridin-4-yl)-N-(3,4,5-trimethoxyphenyl)quinolin-4-amine (31)

6-bromo-N-(3,4,5-trimethoxyphenyl)quinolin-4-amine (**6**) (50.0 mg, 0.13 mmol) and pyridin-4-ylboronic acid (19.0 mg, 0.15 mmol) were used general procedure B to afford the title compound as a yellow solid (36.2 mg, 72.4% yield). HPLC RT = 2.132 min, 99% purity; Exact mass C_23_H_21_N_3_O_3_ 387.16, found [M + H]^+^ = 388.25; ^1^H NMR (400 MHz, DMSO-*d*_6_) *δ* 9.21 (s, 1H), 8.88 (d, *J* = 2.0 Hz, 1H), 8.72 (d, *J* = 1.7 Hz, 1H), 8.71 (d, *J* = 1.8 Hz, 1H), 8.48 (d, *J* = 5.4 Hz, 1H), 8.15 (dd, *J* = 8.8, 1.9 Hz, 1H), 8.00 − 7.94 (m, 2H), 7.00 (d, *J* = 5.4 Hz, 1H), 6.71 (s, 2H), 3.80 (s, 5H), 3.69 (s, 3H); ^13^C NMR (126 MHz, DMSO) *δ* 153.4, 151.3, 150.3, 148.8, 148.7, 146.5, 135.9, 134.4, 133.0, 129.8, 127.5, 121.3, 120.6, 119.5, 101.9, 101.0, 60.2, 55.9.

#### 4.1.32. 6-(Pyridin-3-yl)-N-(3,4,5-trimethoxyphenyl)quinolin-4-amine (32)

6-bromo-N-(3,4,5-trimethoxyphenyl)quinolin-4-amine (**6**) (50.0 mg, 0.13 mmol) and pyridin-3-ylboronic acid (19.2 mg, 0.15 mmol) were used general procedure B to afford the title compound as a yellow solid (34.9 mg, 70.1% yield). HPLC RT = 2.150 min, 100% purity; Exact mass C_23_H_21_N_3_O_3_ 387.16, found [M + H]^+^ = 388.26; ^1^H NMR (400 MHz, DMSO-*d*_6_) *δ* 9.17 (d, *J* = 2.4 Hz, 1H), 8.79 (d, *J* = 1.9 Hz, 1H), 8.62 (dd, *J* = 4.8, 1.6 Hz, 1H), 8.47 (d, *J* = 5.4 Hz, 1H), 8.30 (dt, *J* = 8.1, 2.0 Hz, 1H), 8.10 (dd, *J* = 8.8, 1.9 Hz, 1H), 7.97 (d, *J* = 8.7 Hz, 1H), 7.56 (dd, *J* = 8.0, 4.8 Hz, 1H), 7.01 (d, *J* = 5.4 Hz, 1H), 6.71 (s, 2H), 3.80 (s, 5H), 3.69 (s, 2H); ^13^C NMR (126 MHz, DMSO) *δ* 153.4, 150.9, 148.6, 148.4, 148.2, 148.0, 136.0, 135.1, 134.3, 134.2, 133.1, 129.8, 127.9, 123.9, 120.1, 119.6, 101.8, 100.8, 60.2, 55.9.

#### 4.1.33. 6-(Pyridin-2-yl)-N-(3,4,5-trimethoxyphenyl)quinolin-4-amine (33)

6-bromo-N-(3,4,5-trimethoxyphenyl)quinolin-4-amine (**6**) (50.0 mg, 0.13 mmol) and pyridin-2-ylboronic acid (47.4 mg, 0.39 mmol) were used general procedure C to afford the title compound as a yellow solid (18.0 mg, 36.2% yield). HPLC RT = 2.321 min, 94% purity; Exact mass C_23_H_21_N_3_O_3_ 387.16, found [M + H]^+^ = 388.28; ^1^H NMR (400 MHz, DMSO-*d*_6_) *δ* 9.21 (s, 1H), 9.07 (d, *J* = 1.9 Hz, 1H), 8.74 (d, *J* = 4.8 Hz, 1H), 8.52 − 8.43 (m, 2H), 8.25 (d, *J* = 8.1 Hz, 1H), 8.01 − 7.93 (m, 2H), 7.41 (dd, *J* = 7.4, 4.8 Hz, 1H), 7.00 (d, *J* = 5.4 Hz, 1H), 6.72 (s, 2H), 3.80 (s, 5H), 3.69 (s, 2H); ^13^C NMR (126 MHz, DMSO) *δ* 155.7, 153.4, 151.0, 149.6, 148.9, 148.7, 137.2, 136.1, 134.8, 134.2, 129.2, 127.6, 122.6, 120.7, 120.2, 119.4, 102.0, 100.7, 60.2, 55.9.

#### 4.1.34. 6-(Pyrimidin-5-yl)-N-(3,4,5-trimethoxyphenyl)quinolin-4-amine (34)

6-bromo-N-(3,4,5-trimethoxyphenyl)quinolin-4-amine (**6**) (50.0 mg, 0.13 mmol) and pyrimidin-5-ylboronic acid (19.1 mg, 0.15 mmol) were used general procedure B to afford the title compound as a yellow solid (31.4 mg, 62.9% yield). HPLC RT = 2.373 min, 100% purity; Exact mass C_22_H_20_N_4_O_3_ 388.15, found [M + H]^+^ = 389.25; ^1^H NMR (400 MHz, DMSO-*d*_6_) *δ* 9.38 (s, 1H), 9.23 (s, 0H), 9.14 − 9.03 (m, 0H), 8.86 (d, *J* = 2.0 Hz, 1H), 8.48 (d, *J* = 5.3 Hz, 1H), 8.16 (dd, *J* = 8.7, 1.9 Hz, 1H), 7.98 (d, *J* = 8.7 Hz, 1H), 7.02 (d, *J* = 5.3 Hz, 1H), 6.71 (s, 1H), 3.80 (s, 3H), 3.69 (s, 2H); ^13^C NMR (126 MHz, DMSO) *δ* 157.3, 154.9, 153.4, 151.5, 148.7, 148.3, 135.9, 134.4, 132.8, 130.2, 129.6, 127.4, 120.5, 119.6, 101.8, 100.8, 60.2, 56.0.

#### 4.1.35. 6–(1 h-indazol-4-yl)-N-(3,4,5-trimethoxyphenyl)quinolin-4-amine (35)

6-bromo-N-(3,4,5-trimethoxyphenyl)quinolin-4-amine (**6**) (50.0 mg, 0.13 mmol) and 4–(4,4,5,5-tetramethyl-1,3,2-dioxaborolan-2-yl)-1H-indazole (37.6 mg, 0.15 mmol) were used general procedure B to afford the title compound as a yellow solid (34.0 mg, 62.1% yield). HPLC RT = 2.674 min, 100% purity; Exact mass C_25_H_22_N_4_O_3_ 426.17, found [M + H]^+^ = 427.29; ^1^H NMR (400 MHz, DMSO-*d*_6_) *δ* 13.31 (s, 1H), 9.07 (s, 1H), 8.72 (d, *J* = 1.9 Hz, 1H), 8.49 (d, *J* = 5.3 Hz, 1H), 8.29 (d, *J* = 1.3 Hz, 1H), 8.07 (dd, *J* = 8.7, 1.8 Hz, 1H), 8.01 (d, *J* = 8.7 Hz, 1H), 7.61 (d, *J* = 8.3 Hz, 1H), 7.51 (dd, *J* = 8.3, 7.1 Hz, 1H), 7.44 − 7.40 (m, 1H), 7.05 (d, *J* = 5.3 Hz, 1H), 6.70 (s, 2H), 3.78 (s, 6H), 3.68 (s, 3H); ^13^C NMR (126 MHz, DMSO) *δ* 153.3, 150.9, 148.3, 148.3, 140.5, 136.4, 135.7, 134.1, 133.8, 133.0, 129.7, 129.6, 126.3, 121.2, 121.2, 120.1, 119.9, 109.5, 102.1, 100.5, 60.2, 55.9.

#### 4.1.36. 6–(1-Methyl-1H-pyrazol-4-yl)-N-(3,4,5-trimethoxyphenyl)quinolin-4-amine (36)

6-bromo-N-(3,4,5-trimethoxyphenyl)quinolin-4-amine (**6**) (50.0 mg, 0.13 mmol) and 1-methyl-4–(4,4,5,5-tetramethyl-1,3,2-dioxaborolan-2-yl)-1H-pyrazole (32.1 mg, 0.15 mmol) were used general procedure B to afford the title compound as a yellow solid (32.8 mg, 65.4% yield). HPLC RT = 2.527 min, 100% purity; Exact mass C_22_H_22_N_4_O_3_ 390.17, found [M + H]^+^ = 391.25; ^1^H NMR (400 MHz, DMSO-*d*_6_) *δ* 8.90 (s, 1H), 8.57 (d, *J* = 1.9 Hz, 1H), 8.38 (d, *J* = 5.3 Hz, 1H), 8.26 (s, 1H), 8.06 (s, 1H), 7.91 (dd, *J* = 8.6, 1.8 Hz, 1H), 7.84 (d, *J* = 8.7 Hz, 1H), 6.94 (d, *J* = 5.3 Hz, 1H), 6.70 (s, 2H), 3.91 (s, 3H), 3.80 (s, 6H), 3.69 (s, 3H); ^13^C NMR (126 MHz, DMSO) *δ* 153.4, 149.7, 147.8, 147.3, 136.4, 136.2, 134.2, 129.4, 129.0, 128.1, 127.2, 121.9, 119.8, 116.6, 101.7, 100.8, 60.2, 55.9, 38.7.

#### 4.1.37. 6–(1 h-pyrazol-4-yl)-N-(3,4,5-trimethoxyphenyl)quinolin-4-amine (37)

6-bromo-N-(3,4,5-trimethoxyphenyl)quinolin-4-amine (**6**) (50.0 mg, 0.13 mmol) and 4–(4,4,5,5-tetramethyl-1,3,2-dioxaborolan-2-yl)-1H-pyrazole (30.0 mg, 0.15 mmol) were used general procedure B to afford the title compound as a yellow solid (26.9 mg, 55.6% yield). HPLC RT = 2.425 min, 100% purity; Exact mass C_21_H_20_N_4_O_3_ 376.15, found [M + H]^+^ = 377.26; ^1^H NMR (400 MHz, DMSO-*d*_6_) *δ* 13.05 (s, 1H), 8.89 (s, 1H), 8.59 (d, *J* = 1.9 Hz, 1H), 8.37 (d, *J* = 5.3 Hz, 1H), 8.34 (s, 1H), 8.13 (s, 1H), 7.97 (dd, *J* = 8.7, 1.8 Hz, 1H), 7.83 (d, *J* = 8.7 Hz, 1H), 6.92 (d, *J* = 5.3 Hz, 1H), 6.70 (s, 2H), 3.79 (s, 5H), 3.69 (s, 3H); ^13^C NMR (151 MHz, DMSO) *δ* 153.4, 149.7, 147.8, 147.2, 136.5, 136.2, 134.2, 129.4, 129.3, 127.5, 125.8, 121.1, 119.8, 116.7, 101.6, 100.9, 60.2, 55.9.

#### 4.1.38. 6–(3-Methyl-1H-pyrazol-4-yl)-N-(3,4,5-trimethoxyphenyl)quinolin-4-amine (38)

6-bromo-N-(3,4,5-trimethoxyphenyl)quinolin-4-amine (**6**) (50.0 mg, 0.13 mmol) and 3-methyl-4–(4,4,5,5-tetramethyl-1,3,2-dioxaborolan-2-yl)-1H-pyrazole (32.1 mg, 0.15 mmol) were used general procedure B to afford the title compound as a yellow solid (28.8 mg, 57.4% yield). HPLC RT = 2.402 min, 100% purity; Exact mass C_22_H_22_N_4_O_3_ 390.17, found [M + H]^+^ = 391.27; ^1^H NMR (400 MHz, DMSO-*d*_6_) *δ* 12.77 (s, 1H), 8.89 (s, 1H), 8.41 (d, *J* = 5.3 Hz, 1H), 8.37 (d, *J* = 1.9 Hz, 1H), 7.88 (d, *J* = 8.7 Hz, 1H), 7.81 (dd, *J* = 8.7, 1.8 Hz, 1H), 6.96 (d, *J* = 5.3 Hz, 1H), 6.68 (s, 2H), 3.78 (s, 6H), 3.68 (s, 3H), 2.48 (s, 4H); ^13^C NMR (126 MHz, DMSO) *δ* 153.3, 149.9, 147.8, 147.1, 138.7, 136.5, 135.1, 134.1, 130.3, 129.2, 128.8, 119.8, 118.7, 118.0, 101.9, 100.6, 60.2, 55.9, 26.8.

### 4.2. RIPK2 kinase inhibition assay

RIPK2 kinase activity was analysed using the ADP-Glo™ Assay Kit (RIPK2 Kinase Enzyme System, Promega, USA). Reactions were performed according to the instructions. Specific signal was calculated by subtracting values in the wells without protein and inhibitor from the values in the test wells. Inhibition, % =(((specific signal (DMSO control) – specific signal (inhibitor))/(specific signal (DMSOcontrol))) × 100%. IC_50_ values were calculated by dose-response curve fitting using Prism (Graph Pad).

### 4.3. Cell culture and stimulation

Raw 264.7 was cultured in DMEM containing 10% (w/v) foetal bovine serum (FBS). Cell were seeded at a density of 1 × 105 cells/well in a 48 well plate overnight, then cells were treated with different concentrations of compounds for1h, following by stimulated with L18-MDP (20 µg/mL, Invivogen) for 24 h. The Cell supernatants were harvested for enzyme-linked immunosorbent assay (ELISA).

### 4.4. Elisa for mouse TNFα

TNFα commercial ELISA kits (DAKEWE) were used to detect TNFα in cell culture supernatants according to the manufacturer’s instructions. The concentration of cytokines was determined using multiwell spectrophotometer (SpetraMAX PLUS, Molecular Devices).

## Supplementary Material

Supplemental Material
