# Supplementary material for: Design, synthesis and biological evaluation of 4-aminoquinoline derivatives as receptor-interacting protein kinase 2 (RIPK2) inhibitors
Source: J Enzyme Inhib Med Chem. 2022 Nov 21;38(1):282–93. doi: 10.1080/14756366.2022.2148317 (PMC9683047; doi:10.1080/14756366.2022.2148317)
Supplement: Supplemental Material [file IENZ_A_2148317_SM4803.pdf]

Design, Synthesis and Biological Evaluation of 4-aminoquinoline Derivatives as Receptor-Interacting Protein Kinase 2 (RIPK2) Inhibitors

Tiantian Fan,[1,3,#] Yinchun Ji [2,#], Danqi Chen,[1] Xia Peng [2], Jing Ai,[2,3,4,*] Bing Xiong[1,3,*]

[1] Department of Medicinal Chemistry, Shanghai Institute of Materia Medica, Chinese Academy of Sciences, 555 Zuchongzhi Road, Shanghai 201203, P. R. China

[2] Division of Antitumor Pharmacology, State Key Laboratory of Drug Research, Shanghai Institute of Materia Medica, Chinese Academy of Sciences, 555 Zuchongzhi Road, Shanghai 201203, P. R. China

[3] University of Chinese Academy of Sciences, NO.19A Yuquan Road, Beijing 100049, P. R. China

[4] Hangzhou Institute for Advanced Study (UCAS), Hangzhou 310024, P. R. China

content

1. NMR, MS, HPLC for all synthesized compounds are given below.......3
    1.1 Compound 1.......3
    1.2 Compound 2.......5
    1.3 Compound 3.......8
    1.4 Compound 4.......10
    1.5 Compound 5.......12
    1.6 Compound 6.......14
    1.7 Compound 7.......17
    1.8 Compound 8.......19
    1.9 Compound 9.......21
    1.2 Compound 10.......23
    1.11 Compound 11.......26
    1.12 Compound 12.......29
    1.13 Compound 13.......31
    1.3 Compound 14.......34
    1.15 Compound 15.......36
    1.16 Compound 16.......38
    1.17 Compound 17.......40
    1.18 Compound 18.......43
    1.19 Compound 19.......45
    1.20 Compound 20.......48
    1.21 Compound 21.......50
    1.22 Compound 22.......53
    1.23 Compound 23.......55
    1.24 Compound 24.......57
    1.25 Compound 25.......60
    1.26 Compound 26.......62
    1.27 Compound 27.......65
    1.28 Compound 28.......67
    1.29 Compound 29.......69
    1.30 Compound 30.......71
    1.31 Compound 31.......73
    1.32 Compound 32.......76
    1.33 Compound 33.......78
    1.34 Compound 34.......81
    1.35 Compound 35.......83
    1.36 Compound 36.......86
    1.37 Compound 37.......88
    1.38 Compound 38.......91
2. Kinase selectivity of compound 14.......94
3. ClogP and LipE.......95

1. NMR, MS, HPLC for all synthesized compounds are given below

1.1 Compound 1

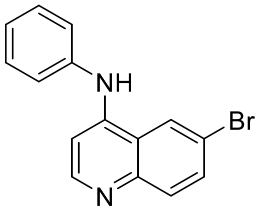

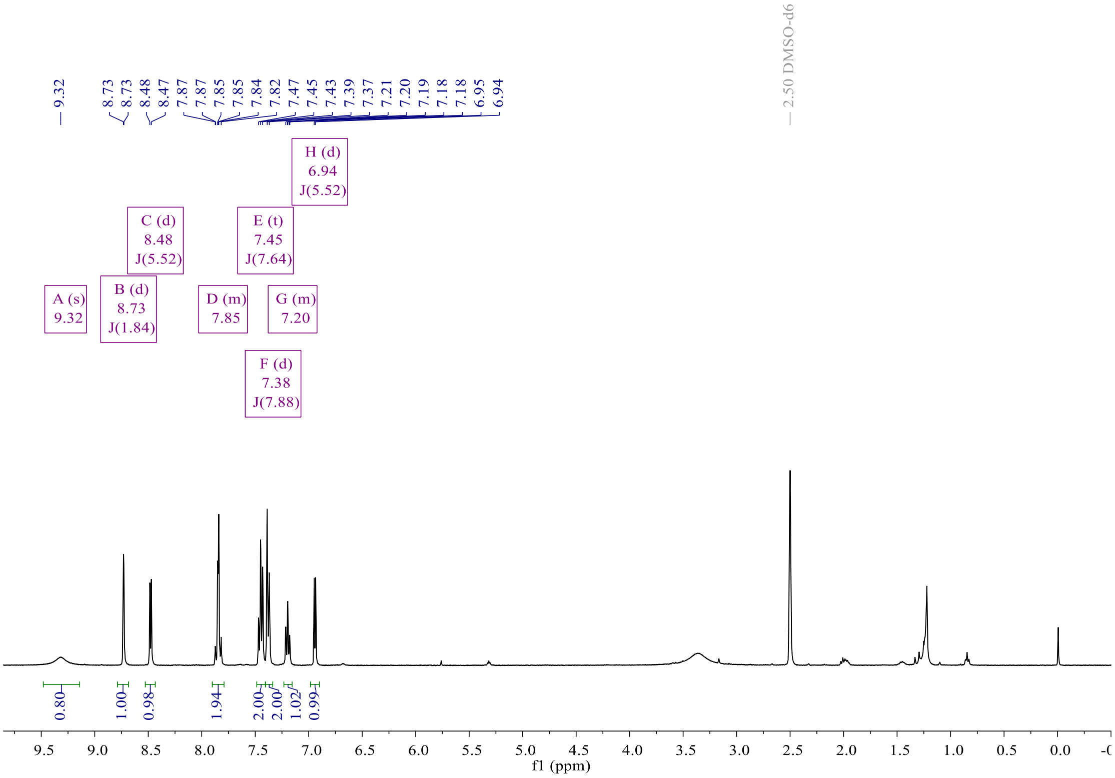

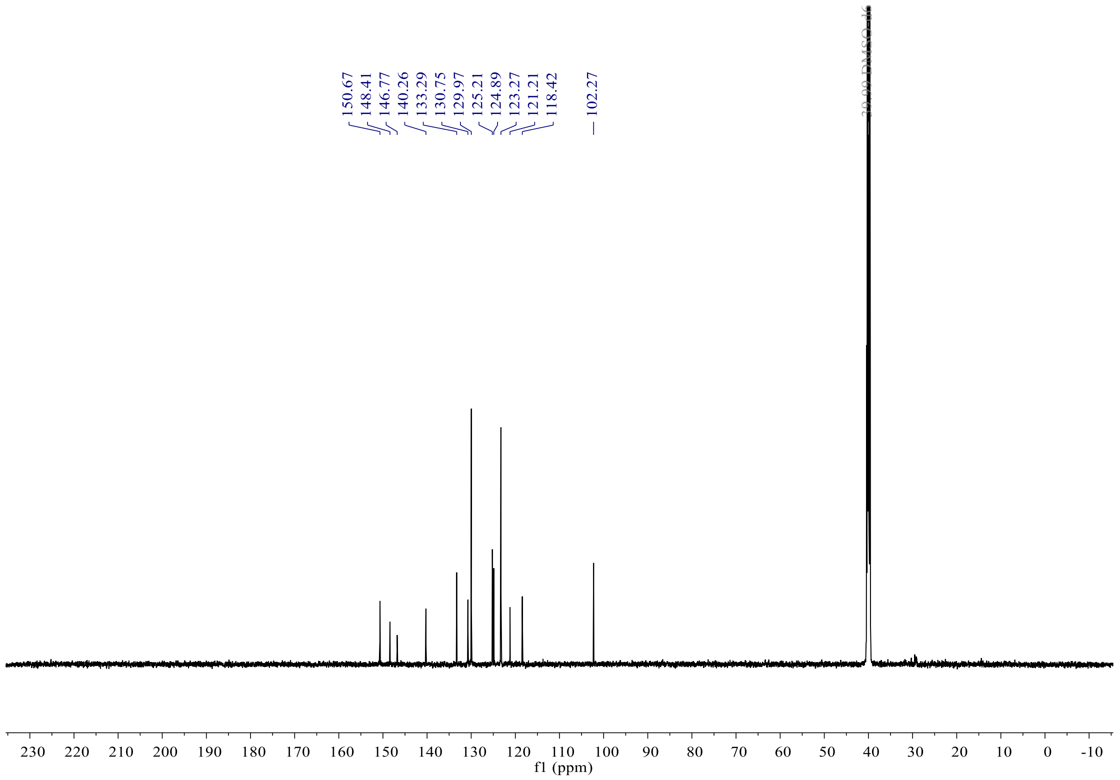

Analysis Report

Graph

**Sample Name**	ftt-7862-146
**Application Name**	Admin App (Administrator)
**Method Name**	User2
**Configuration Name**	Configuration 1
**Version**	23
**Data Instrument Name**	Detector
**Data Channel Name**	156 Channel 1
**Notes**	
**Injection Number**	3

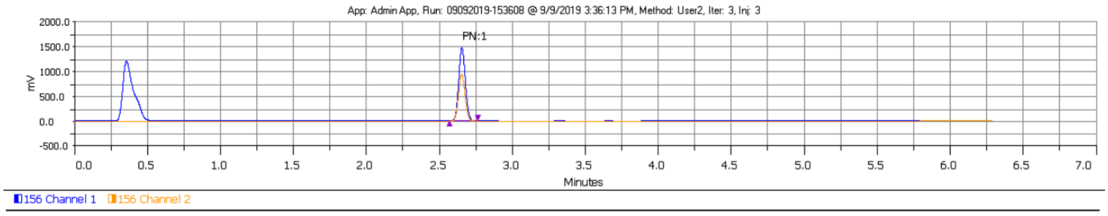

Sample Table

Injection Number	Sample Name	Sample Location	Peak Name	Retention Time (min)	Area (uVmin x100)	Area %	Height (mV)	Plate Number	
3	ftt-7862-146	Sample Zone->14	1	2.657	7822353.75	100	1477.328	15965.109	

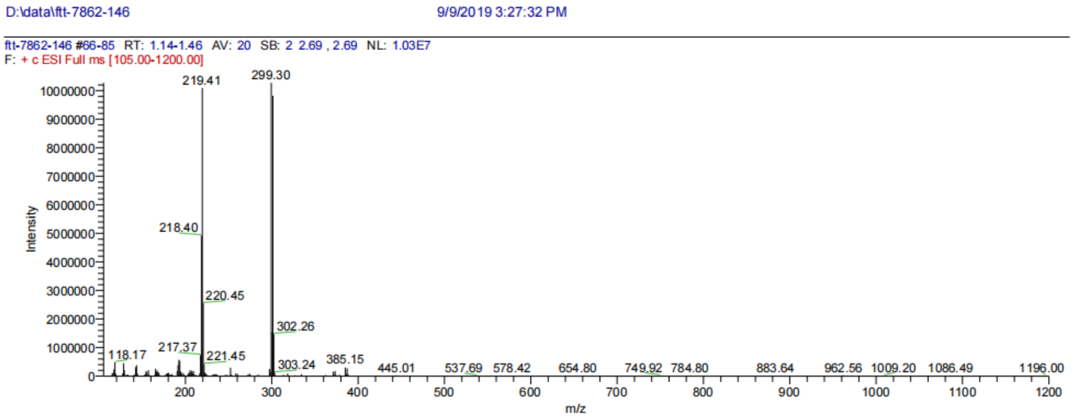

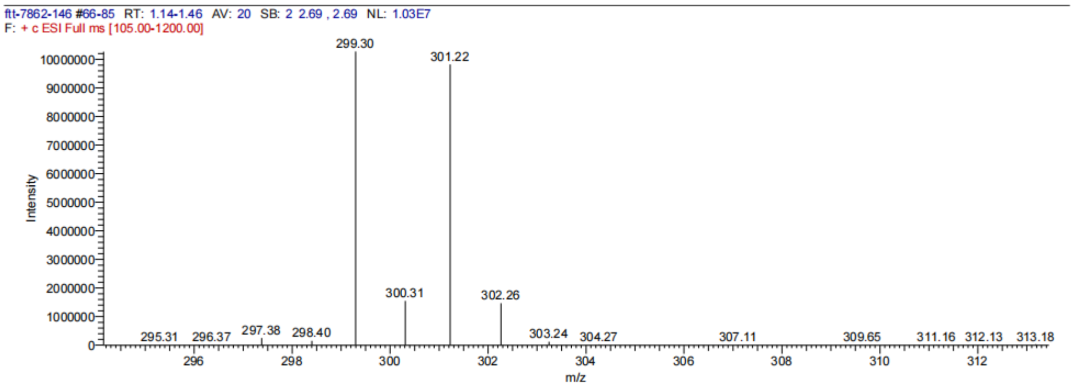

1.2 Compound 2

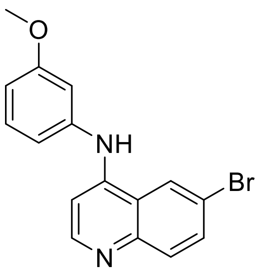

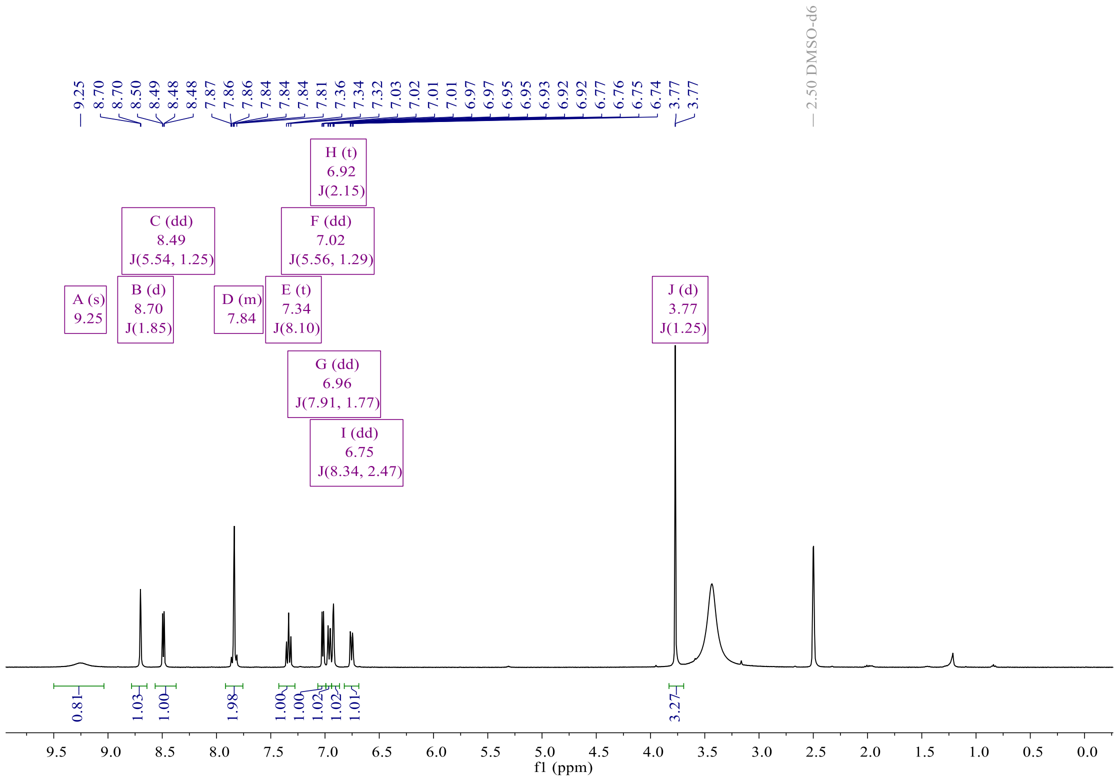
9.25 8.70 8.70 8.50 8.49 8.48 8.48 7.87 7.86 7.86 7.84 7.84 7.84 7.81 7.36 7.34 7.32 7.03 7.02 7.01 7.01 6.97 6.97 6.95 6.95 6.93 6.92 6.92 6.77 6.76 6.75 6.74 3.77 3.77
2.50 DMSO-d6
A (s) 9.25
B (d) 8.70 J(1.85)
C (dd) 8.49 J(5.54, 1.25)
D (m) 7.84
E (t) 7.34 J(8.10)
F (dd) 7.02 J(5.56, 1.29)
G (dd) 6.96 J(7.91, 1.77)
H (t) 6.92 J(2.15)
I (dd) 6.75 J(8.34, 2.47)
J (d) 3.77 J(1.25)
0.81 1.03 1.00 1.98 1.00 1.00 1.02 1.02 1.01 3.27
9.5 9.0 8.5 8.0 7.5 7.0 6.5 6.0 5.5 5.0 4.5 4.0 3.5 3.0 2.5 2.0 1.5 1.0 0.5 0.0
f1 (ppm)

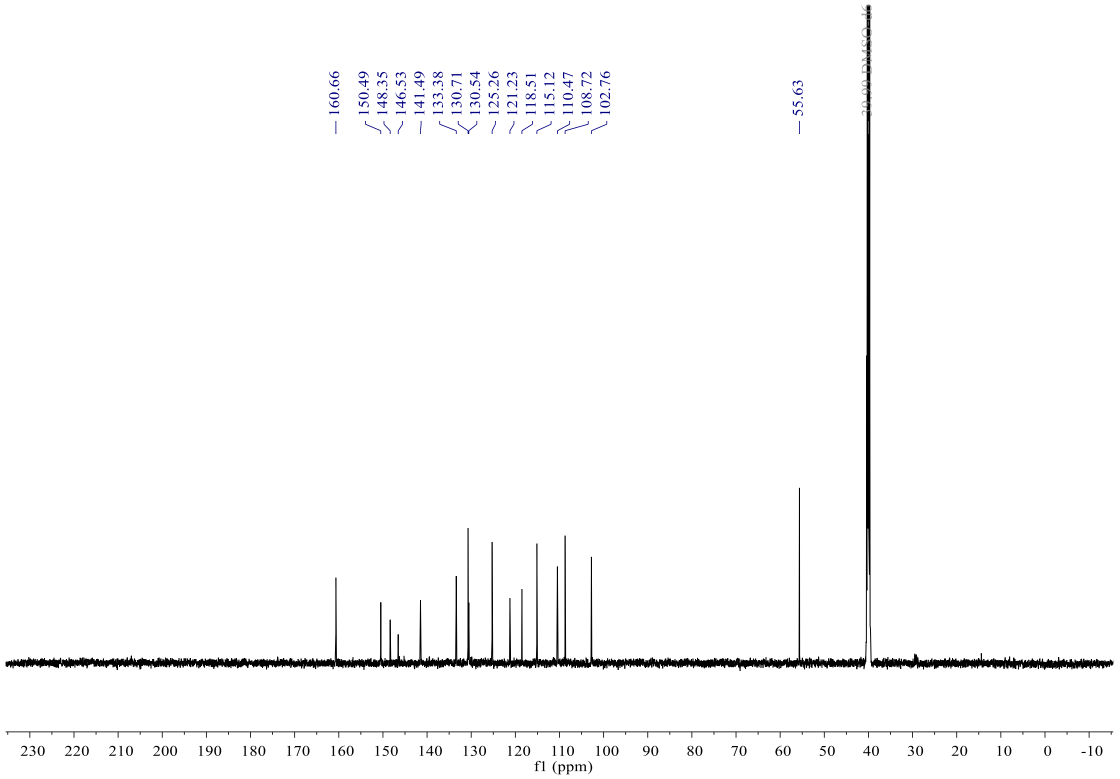
160.66 150.49 148.35 146.53 141.49 133.38 130.71 130.54 125.26 121.23 118.51 115.12 110.47 108.72 102.76
55.63
230 220 210 200 190 180 170 160 150 140 130 120 110 100 90 80 70 60 50 40 30 20 10 0 -10
f1 (ppm)

D:\data\ftt-7862-121 7/25/2019 12:01:39 PM

ftt-7862-121 #52-72 RT: 0.92-1.24 AV: 21 SB: 53 0.42-1.02 , 1.22-1.52 NL: 7.96E7
F: + c ESI Full ms [105.00-1200.00]

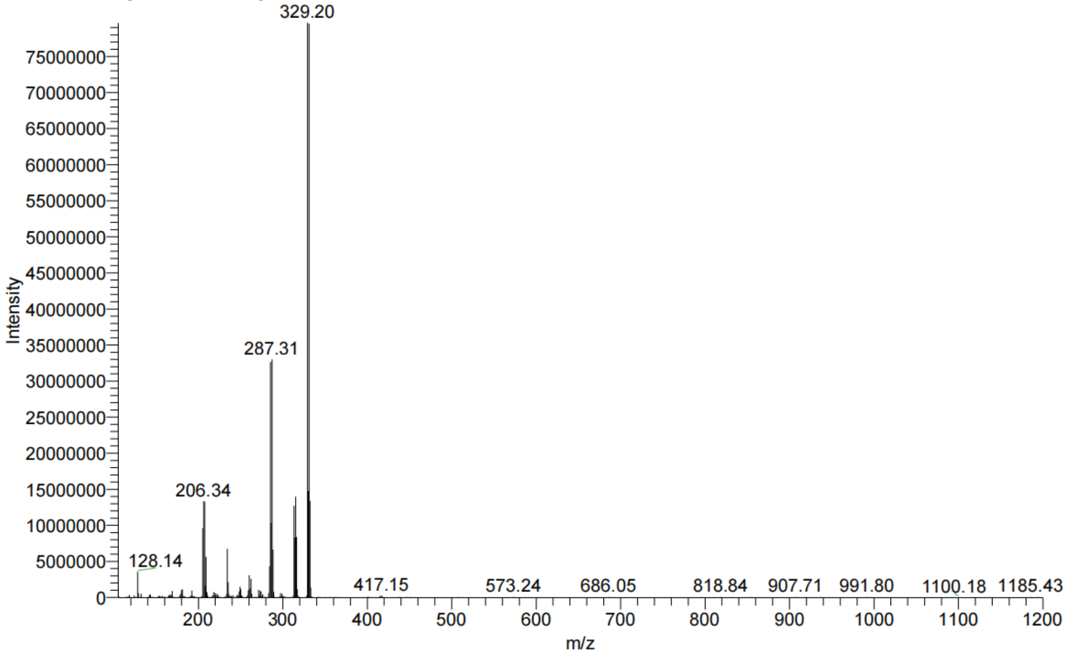

Analysis Report

Graph

**Sample Name**	ftt-7862-121
**Application Name**	Admin App (Administrator)
**Method Name**	User2
**Configuration Name**	Configuration 1
**Version**	18
**Data Instrument Name**	Detector
**Data Channel Name**	156 Channel 1
**Notes**	
**Injection Number**	9

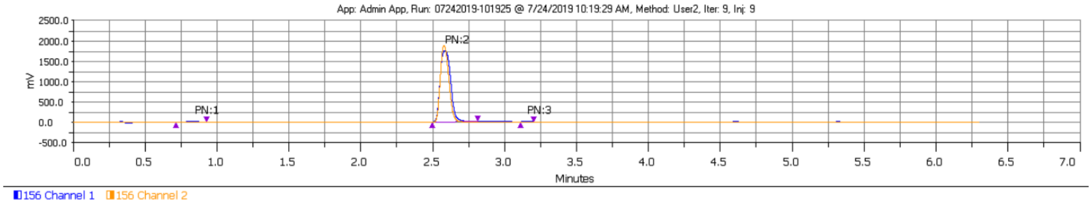

Sample Table

Injection Number	Sample Name	Sample Location	Peak Name	Retention Time (min)	Area (uVmin x100)	Area %	Height (mV)	Plate Number	
9	ftt-7862-121	Sample Zone->20	1	0.841	224782.5	1.504	22.282	398.788	
9	ftt-7862-121	Sample Zone->20	2	2.588	14693864.5833	98.31	1741.46	5641.035	
9	ftt-7862-121	Sample Zone->20	3	3.156	27745.8333	0.186	6.274	29873.149	

1.3 Compound 3

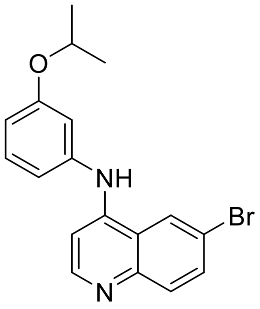

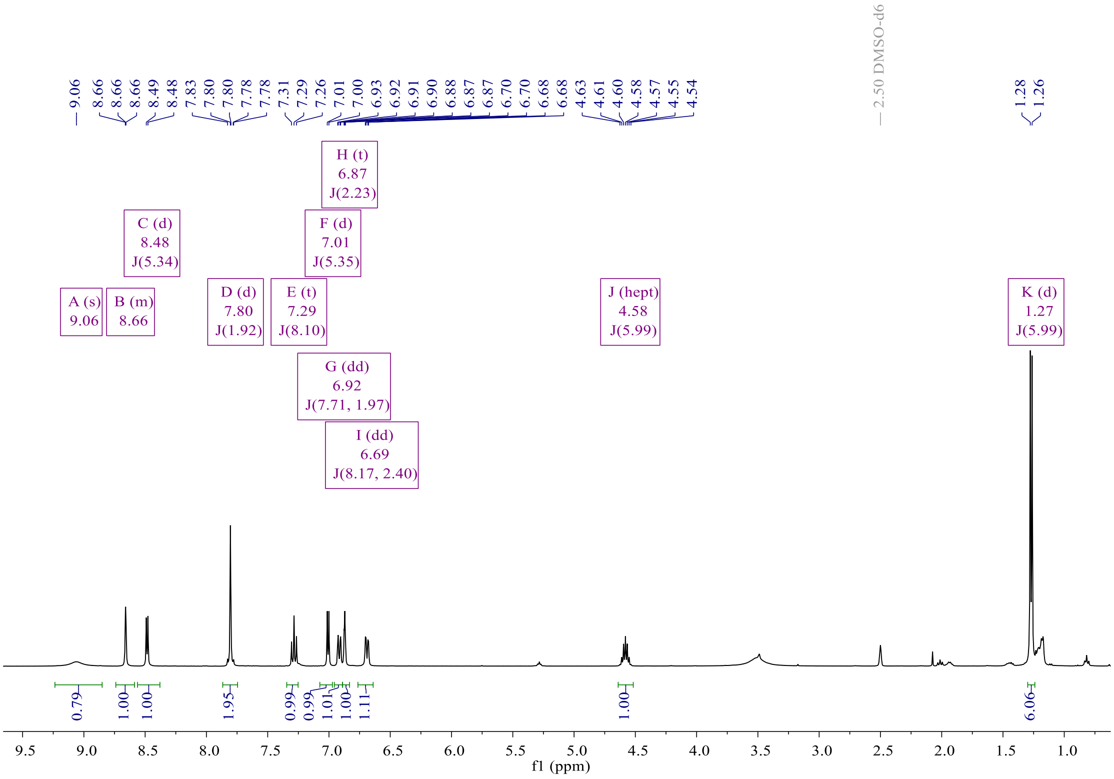

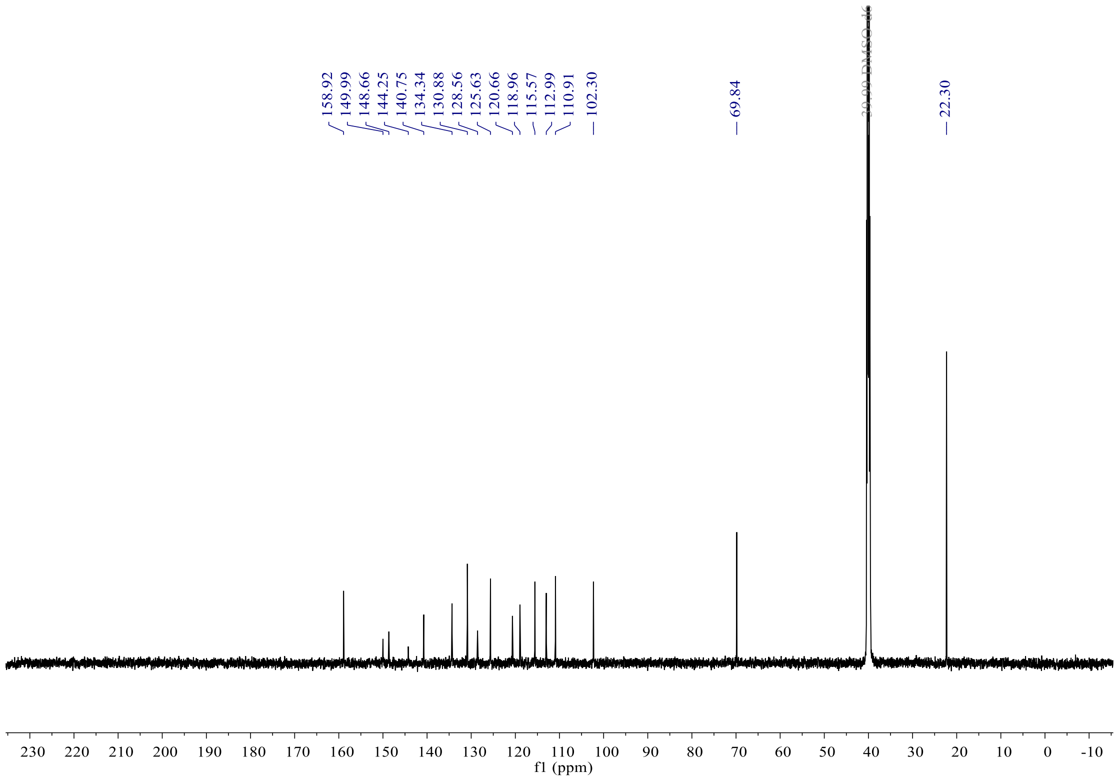

Analysis Report

Graph

**Sample Name**	ftt-11812-050
**Application Name**	Admin App (Administrator)
**Method Name**	User2
**Configuration Name**	Configuration 1
**Version**	23
**Data Instrument Name**	Detector
**Data Channel Name**	156 Channel 1
**Notes**	
**Injection Number**	8

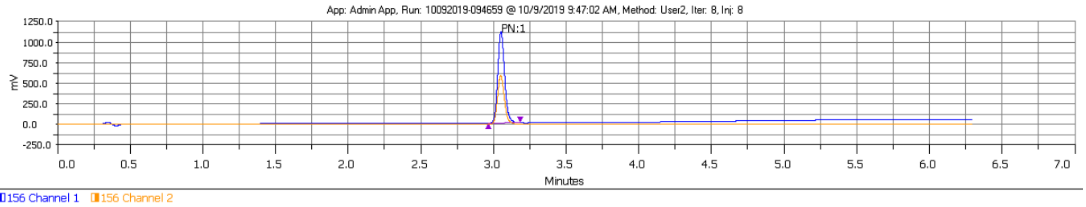

Sample Table

Injection Number	Sample Name	Sample Location	Peak Name	Retention Time (min)	Area (uVmin x100)	Area %	Height (mV)	Plate Number	
8	ftt-11812-050	Sample Zone->10	1	3.054	6086935.8333	100	1112.247	20593.973	

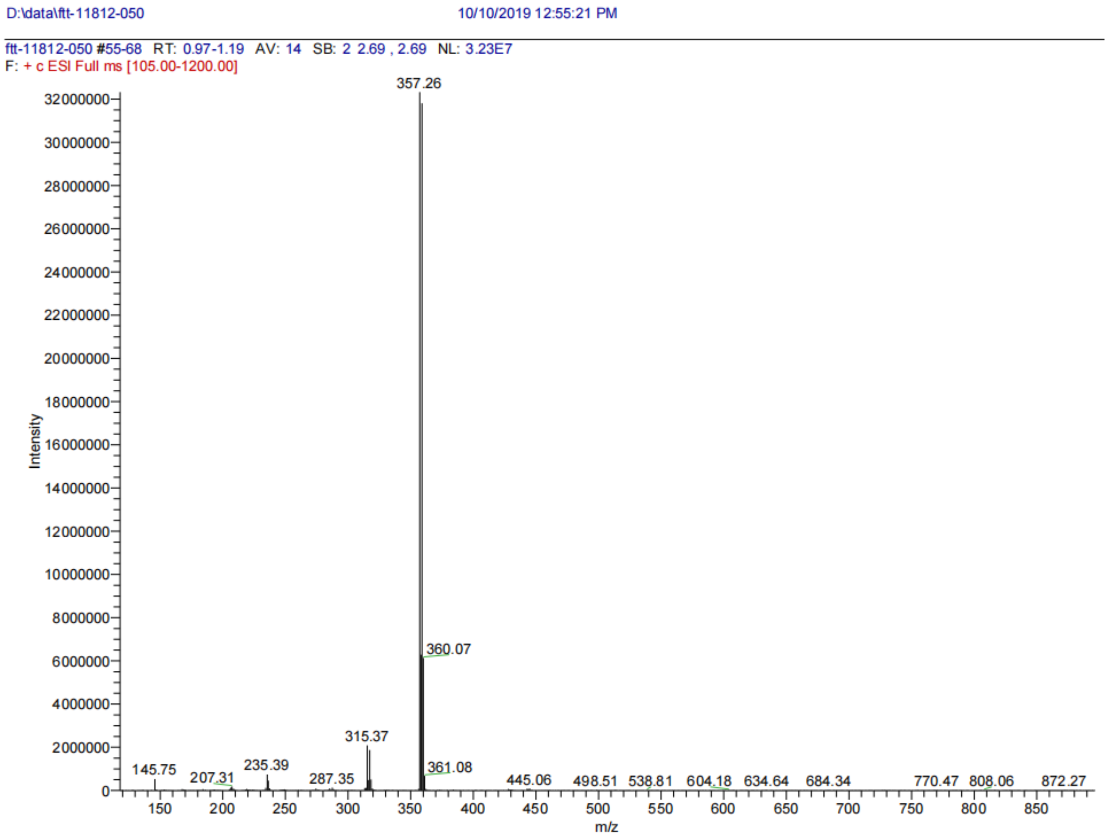

1.4 Compound 4

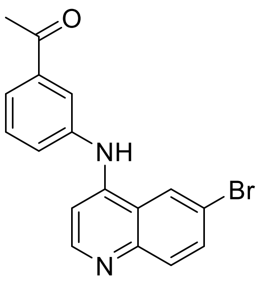

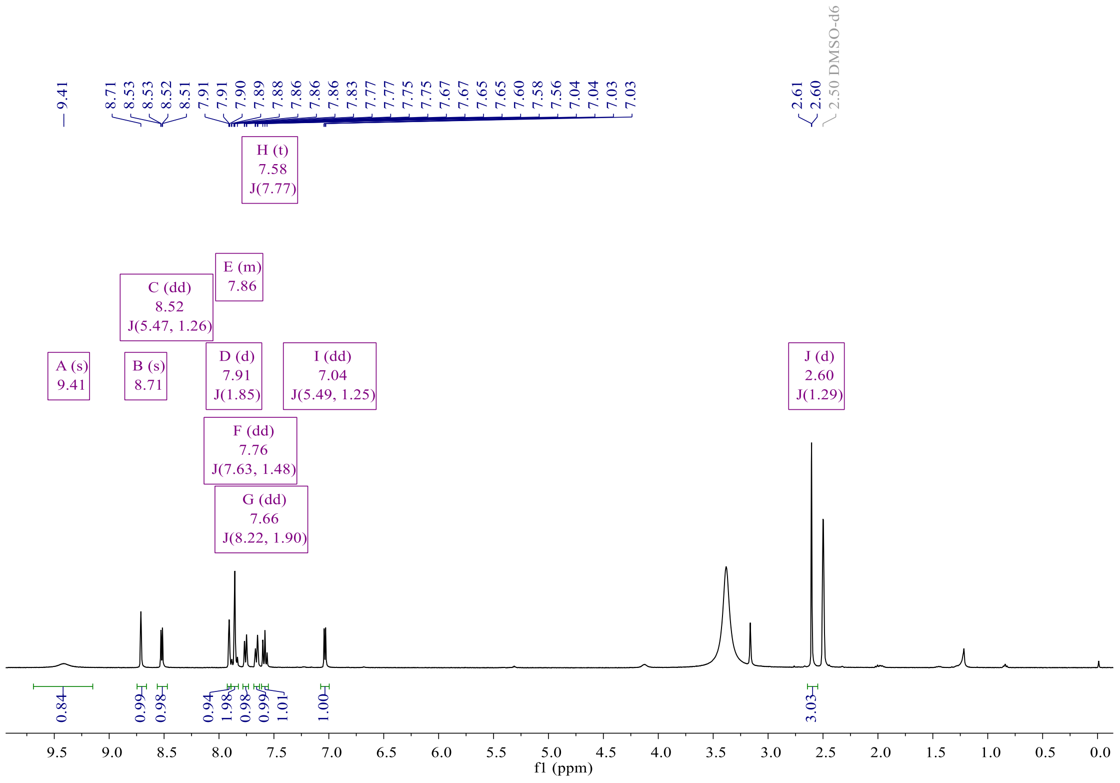

A (s) 9.41
B (s) 8.71
C (dd) 8.52 J(5.47, 1.26)
D (d) 7.91 J(1.85)
E (m) 7.86
F (dd) 7.76 J(7.63, 1.48)
G (dd) 7.66 J(8.22, 1.90)
H (t) 7.58 J(7.77)
I (dd) 7.04 J(5.49, 1.25)
J (d) 2.60 J(1.29)
2.50 DMSO-d6
f1 (ppm)

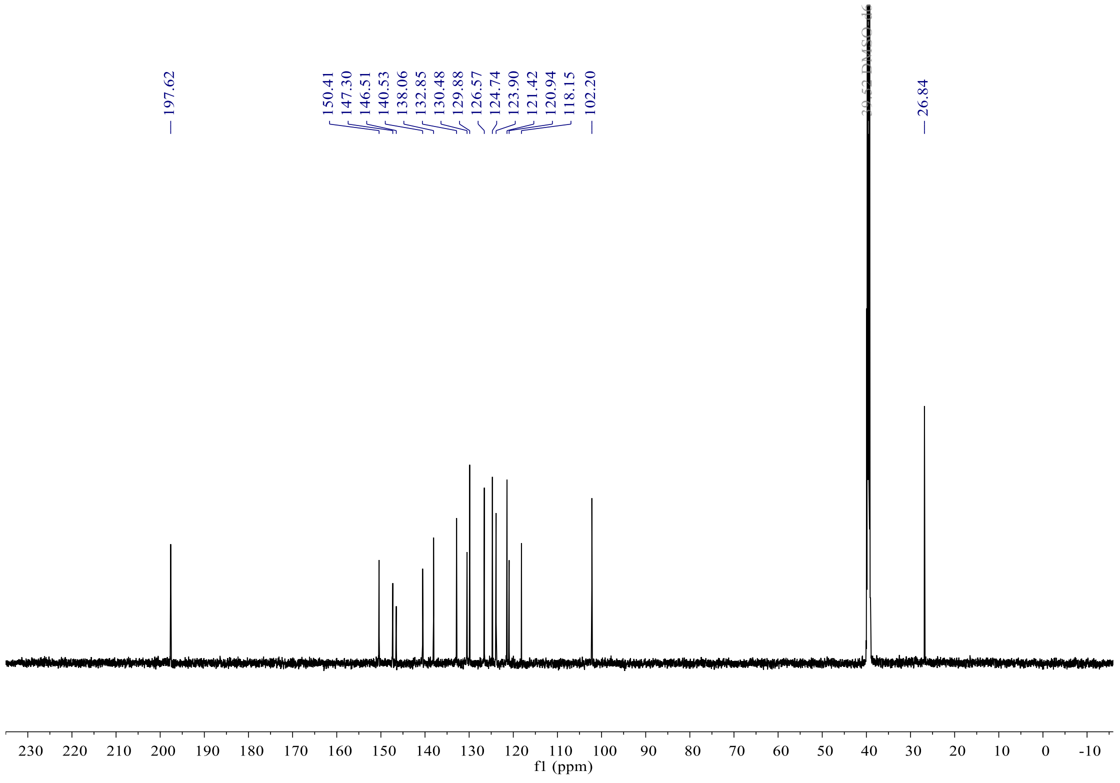

197.62
150.41
147.30
146.51
140.53
138.06
132.85
130.48
129.88
126.57
124.74
123.90
121.42
120.94
118.15
102.20
26.84
f1 (ppm)

Analysis Report

Graph

**Sample Name**	ftt-7862-128
**Application Name**	Admin App (Administrator)
**Method Name**	User2
**Configuration Name**	Configuration 1
**Version**	18
**Data Instrument Name**	Detector
**Data Channel Name**	156 Channel 1
**Notes**	
**Injection Number**	1

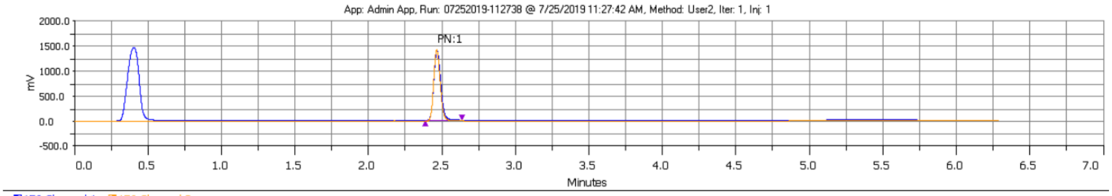

Sample Table

Injection Number	Sample Name	Sample Location	Peak Name	Retention Time (min)	Area (uVmin x100)	Area %	Height (mV)	Plate Number
1	ftt-7862-128	Sample Zone->34	1	2.469	7696070.4167	100	1389.634	12677.365

ftt-7862-128 #51-66 RT: 0.93-1.18 AV: 16 SB: 27 0.73-0.95 , 1.13-1.36 NL: 8.47E7
F: + c ESI Full ms [105.00-1200.00]

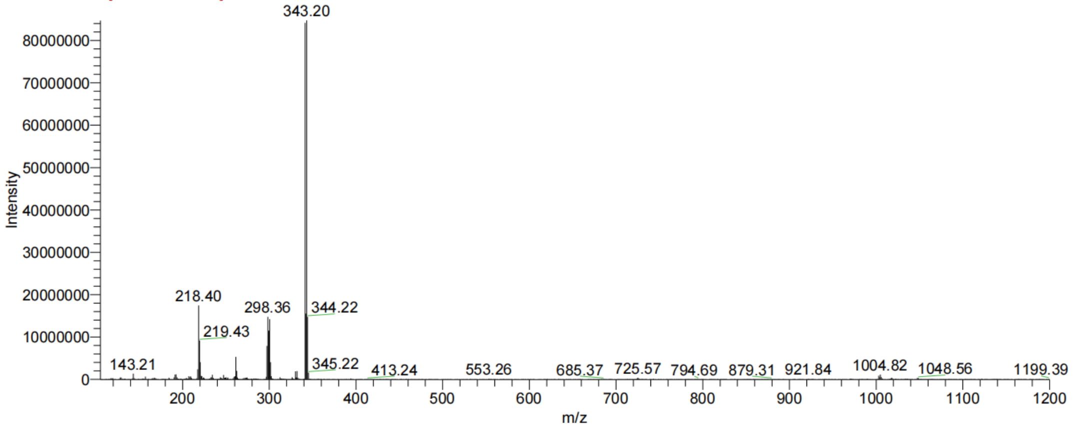

1.5 Compound 5

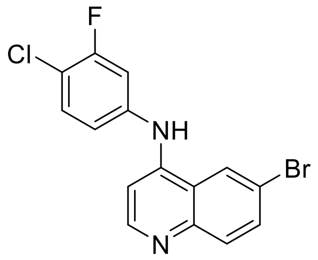

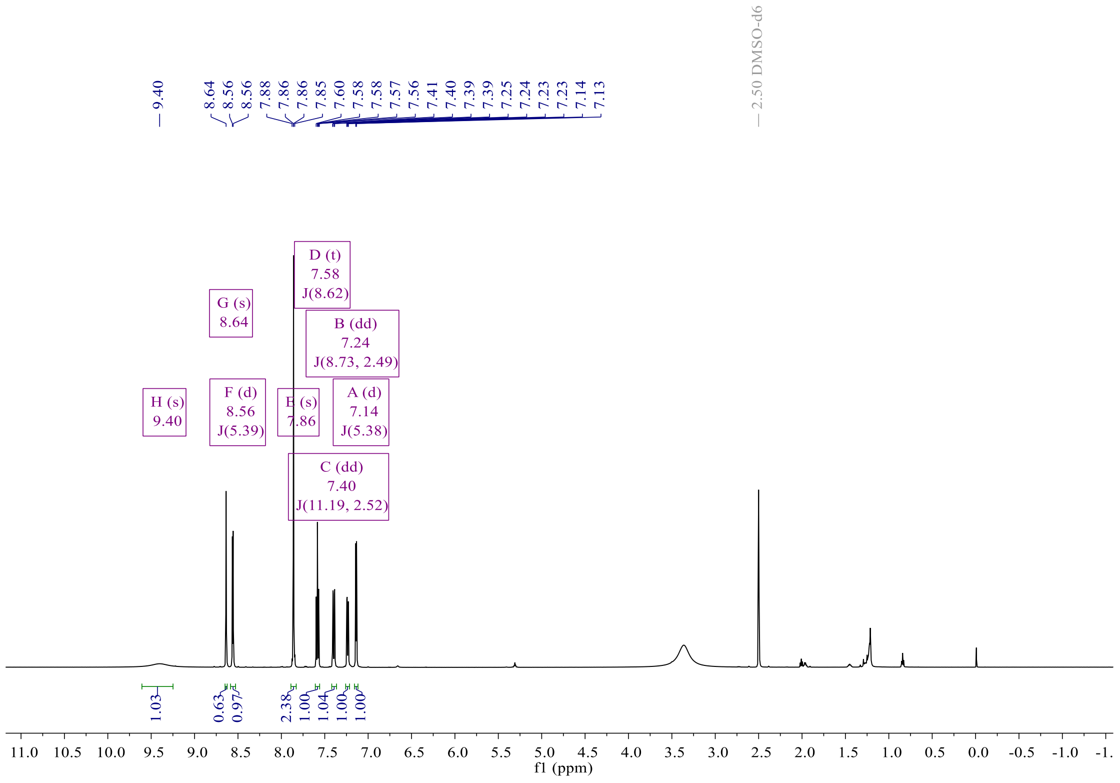

9.40
8.64 8.56 8.56 7.88 7.86 7.86 7.85 7.60 7.58 7.58 7.57 7.56 7.41 7.40 7.39 7.39 7.25 7.24 7.23 7.23 7.14 7.13
2.50 DMSO-d6
H (s) 9.40
G (s) 8.64
F (d) 8.56 J(5.39)
E (s) 7.86
D (t) 7.58 J(8.62)
C (dd) 7.40 J(11.19, 2.52)
B (dd) 7.24 J(8.73, 2.49)
A (d) 7.14 J(5.38)
1.03 0.63 0.97 2.38 1.00 1.04 1.00 1.00
f1 (ppm)

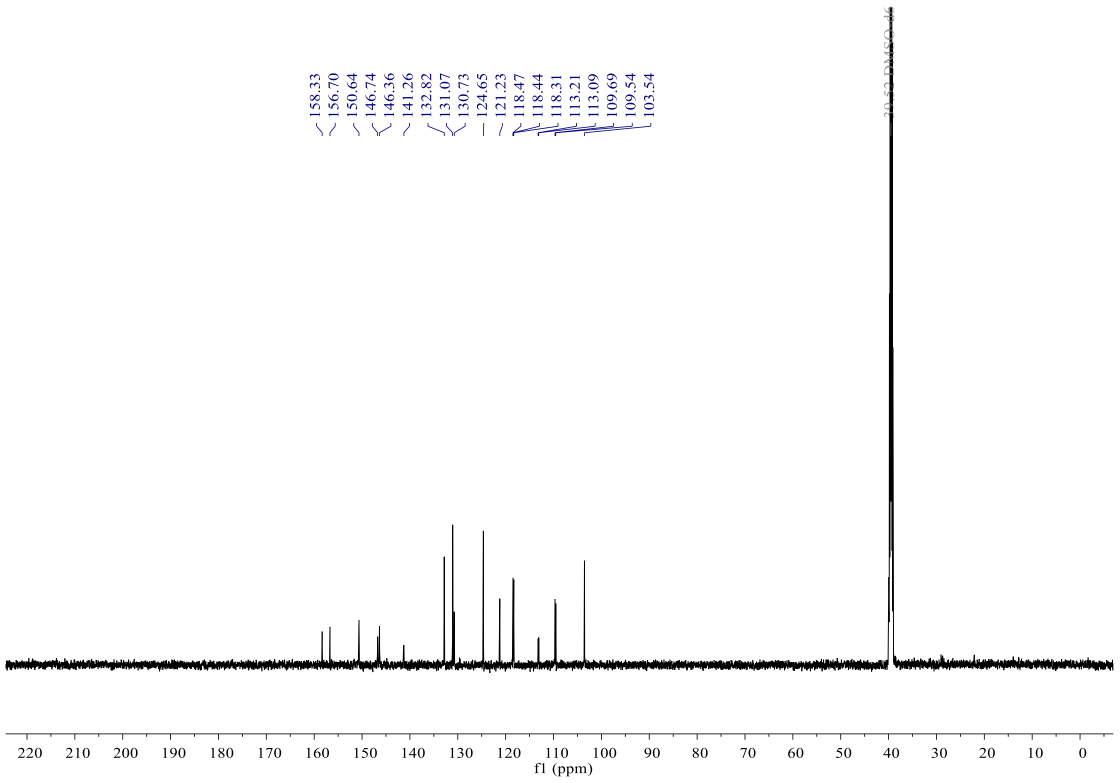

158.33 156.70 150.64 146.74 146.36 141.26 132.82 131.07 130.73 124.65 121.23 118.47 118.44 118.31 113.21 113.09 109.69 109.54 103.54
f1 (ppm)

Analysis Report

Graph

**Sample Name**	ftt-7862-130
**Application Name**	Admin App (Administrator)
**Method Name**	User2
**Configuration Name**	Configuration 1
**Version**	18
**Data Instrument Name**	Detector
**Data Channel Name**	156 Channel 1
**Notes**	
**Injection Number**	3

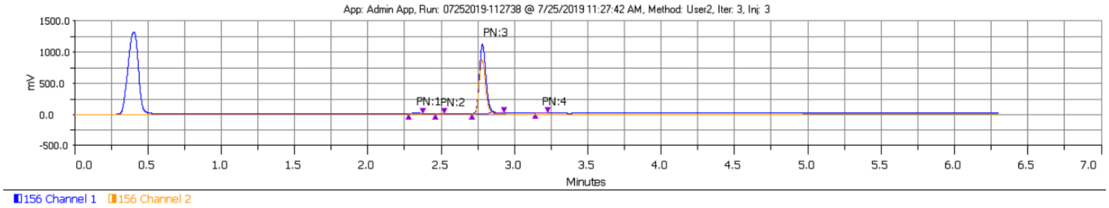

Sample Table

Injection Number	Sample Name	Sample Location	Peak Name	Retention Time (min)	Area (uVmin x100)	Area %	Height (mV)	Plate Number
3	ftt-7862-130	Sample Zone->36	1	2.328	60425.8333	1.047	14.633	19698.545
3	ftt-7862-130	Sample Zone->36	2	2.495	9316.6667	0.162	2.744	29731.396
3	ftt-7862-130	Sample Zone->36	3	2.784	5674390	98.365	1111.133	19559.248
3	ftt-7862-130	Sample Zone->36	4	3.186	24567.9167	0.426	5.693	31767.166

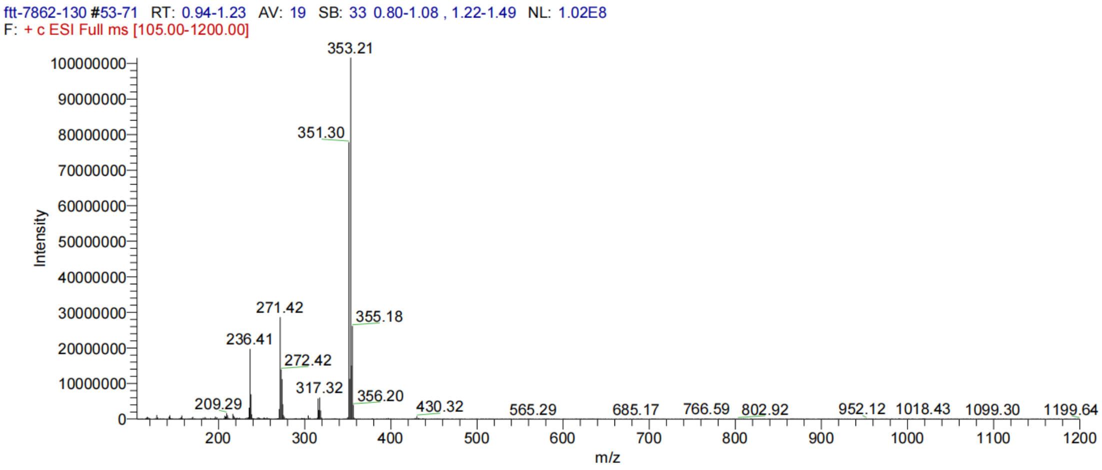

1.6 Compound 6

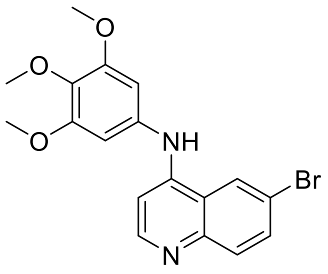

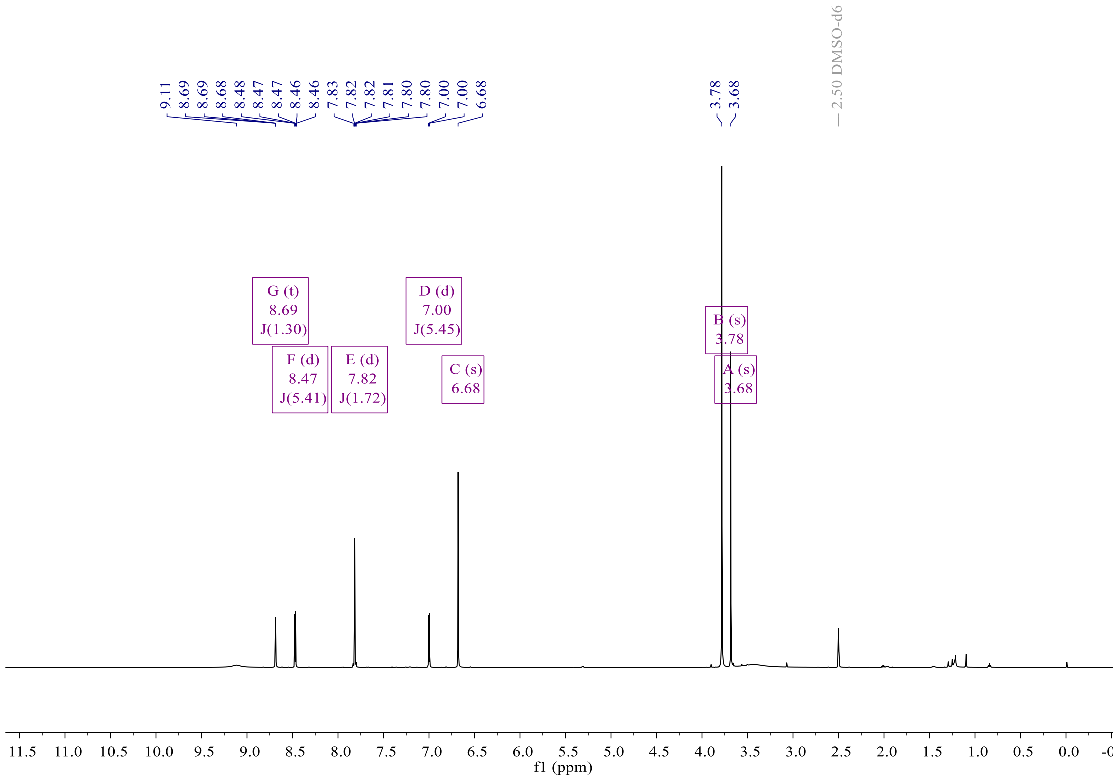
9.11
8.69
8.69
8.68
8.48
8.47
8.47
8.46
8.46
7.83
7.82
7.82
7.81
7.80
7.80
7.00
7.00
6.68
3.78
3.68
2.50 DMSO-d6
G (t) 8.69 J(1.30)
F (d) 8.47 J(5.41)
E (d) 7.82 J(1.72)
D (d) 7.00 J(5.45)
C (s) 6.68
B (s) 3.78
A (s) 3.68
11.5 11.0 10.5 10.0 9.5 9.0 8.5 8.0 7.5 7.0 6.5 6.0 5.5 5.0 4.5 4.0 3.5 3.0 2.5 2.0 1.5 1.0 0.5 0.0
f1 (ppm)

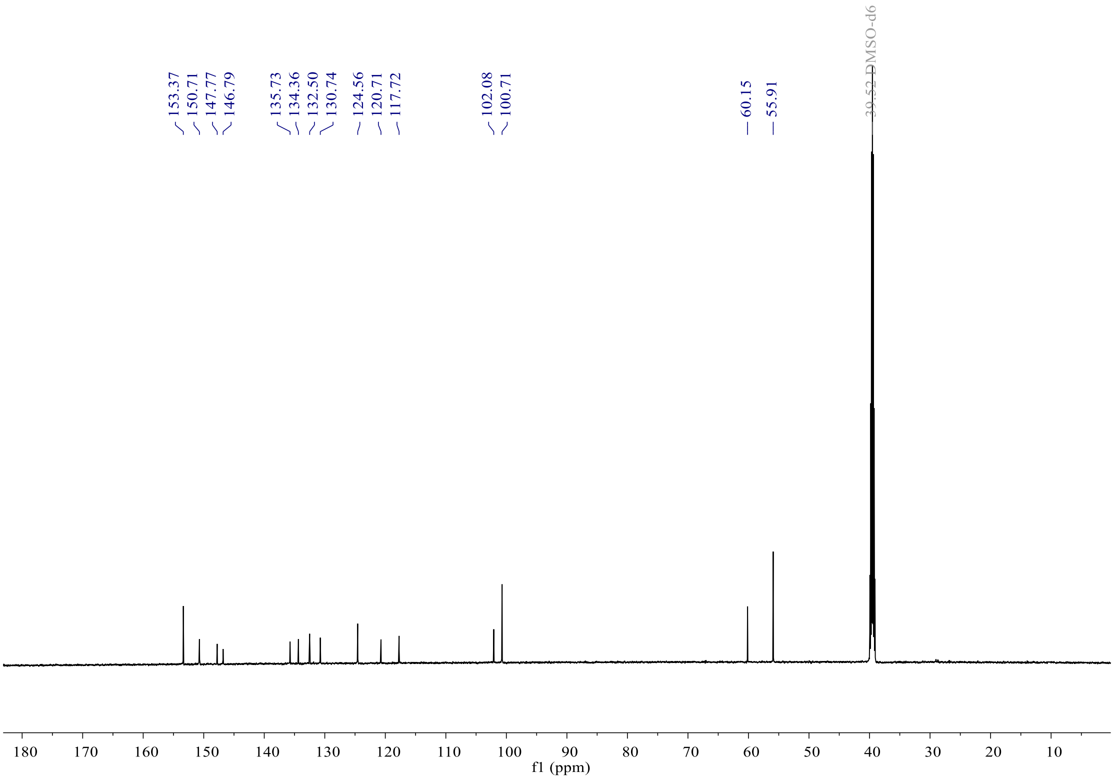

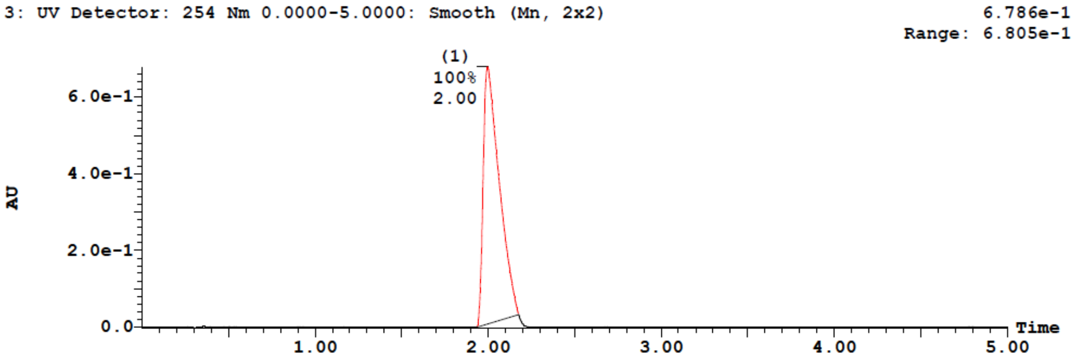

Peak Number	Compound	Time	AreaAbs	Area %Total	Width	Height	Mass Found
1		2.00	7e+004	100.00	0	7e+005	Not Found

Peak ID	Compound	Time	Mass Found	Target
1		2.00	Not Found	No Target

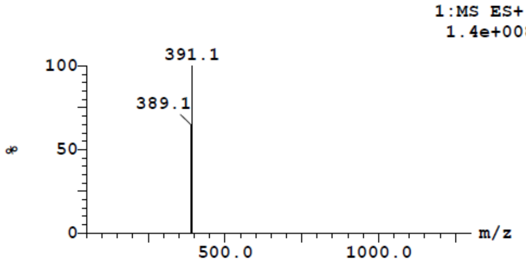

Peak ID	Compound	Time	Mass Found	Target
1		2.00	Not Found	No Target

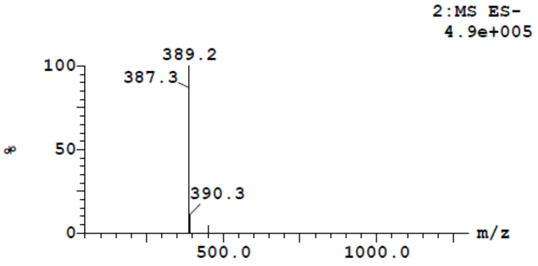

Figure 1. HPLC and MS spectrum of compound **6**

1.7 Compound 7

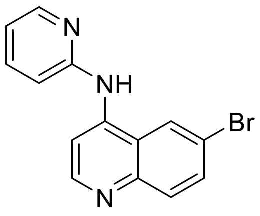

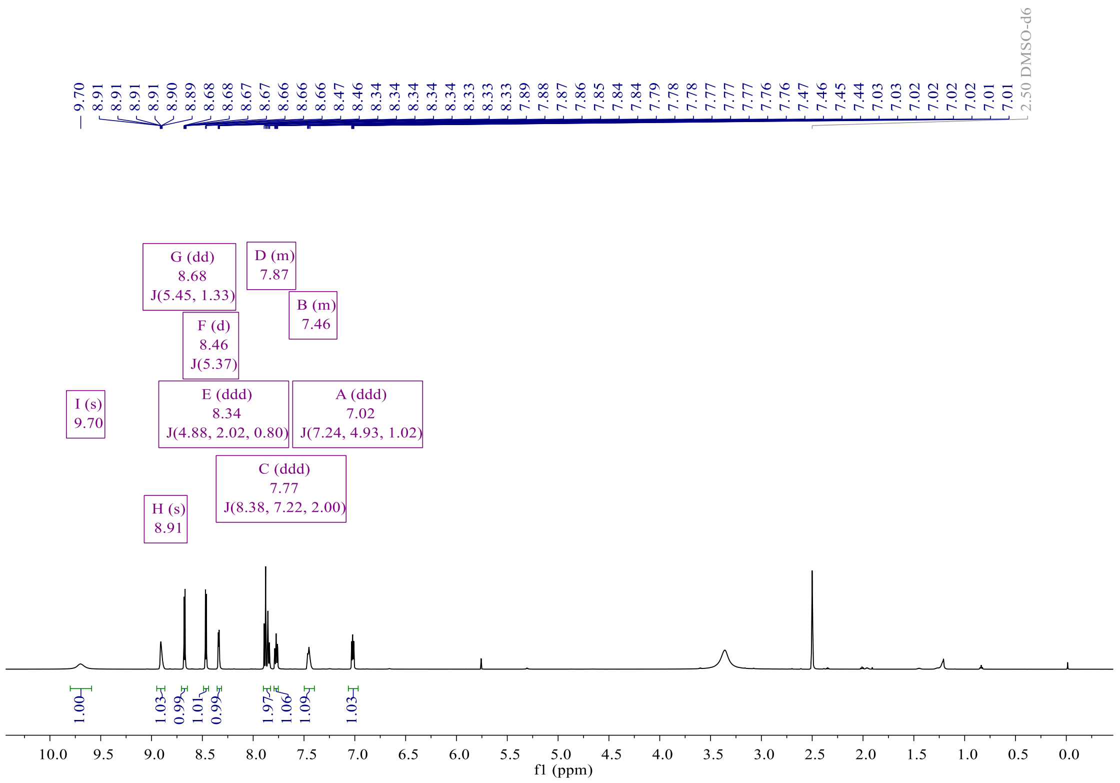

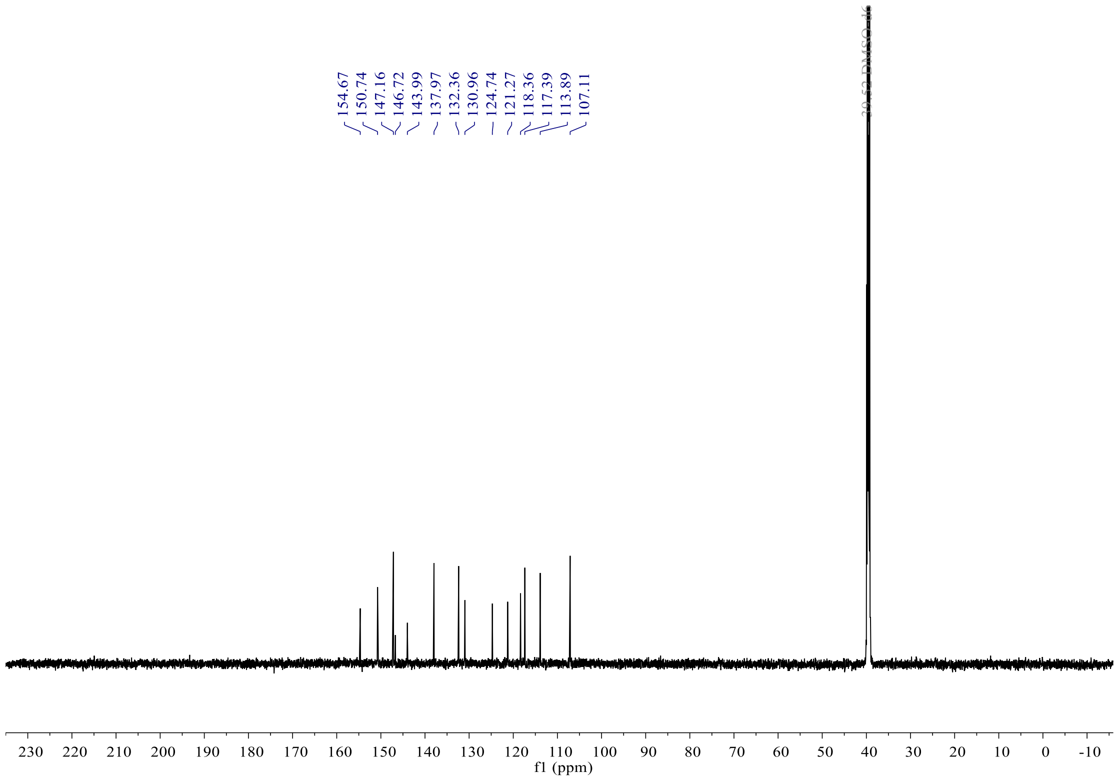

Analysis Report

Graph

**Sample Name**	ftt-11812-012r
**Application Name**	Admin App (Administrator)
**Method Name**	User2
**Configuration Name**	Configuration 1
**Version**	23
**Data Instrument Name**	Detector
**Data Channel Name**	156 Channel 1
**Notes**	
**Injection Number**	5

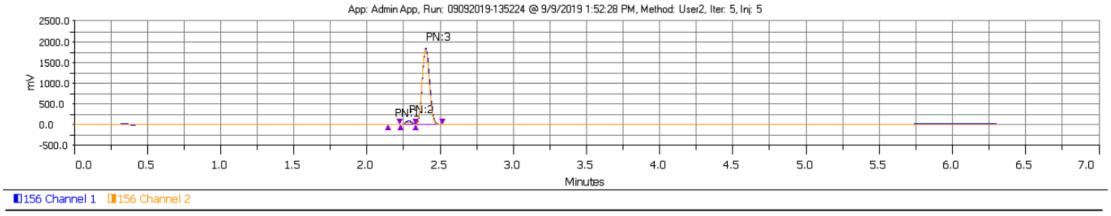

Sample Table

Injection Number	Sample Name	Sample Location	Peak Name	Retention Time (min)	Area (uVmin x100)	Area %	Height (mV)	Plate Number	
5	ftt-11812-012r	Sample Zone->16	1	2.188	24962.0833	0.223	6.135	16183.597	
5	ftt-11812-012r	Sample Zone->16	2	2.288	405808.2572	3.621	84.654	13826.055	
5	ftt-11812-012r	Sample Zone->16	3	2.404	10777405.0761	96.157	1828.953	10224.992	

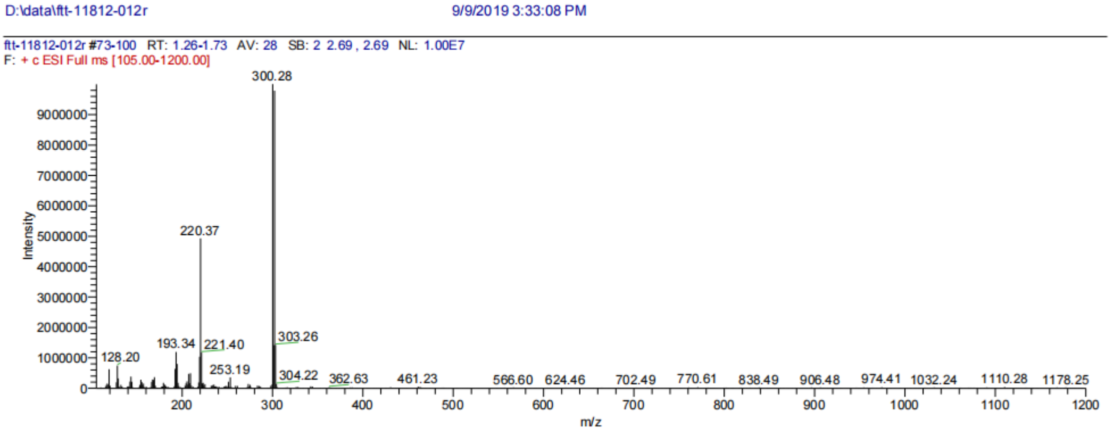

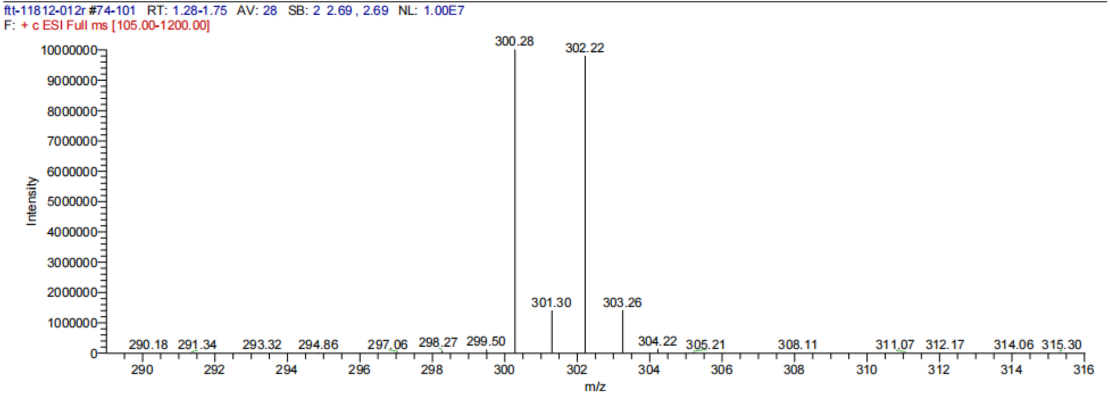

1.8 Compound 8

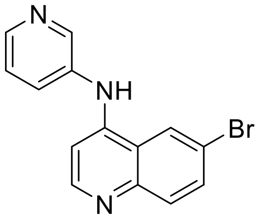

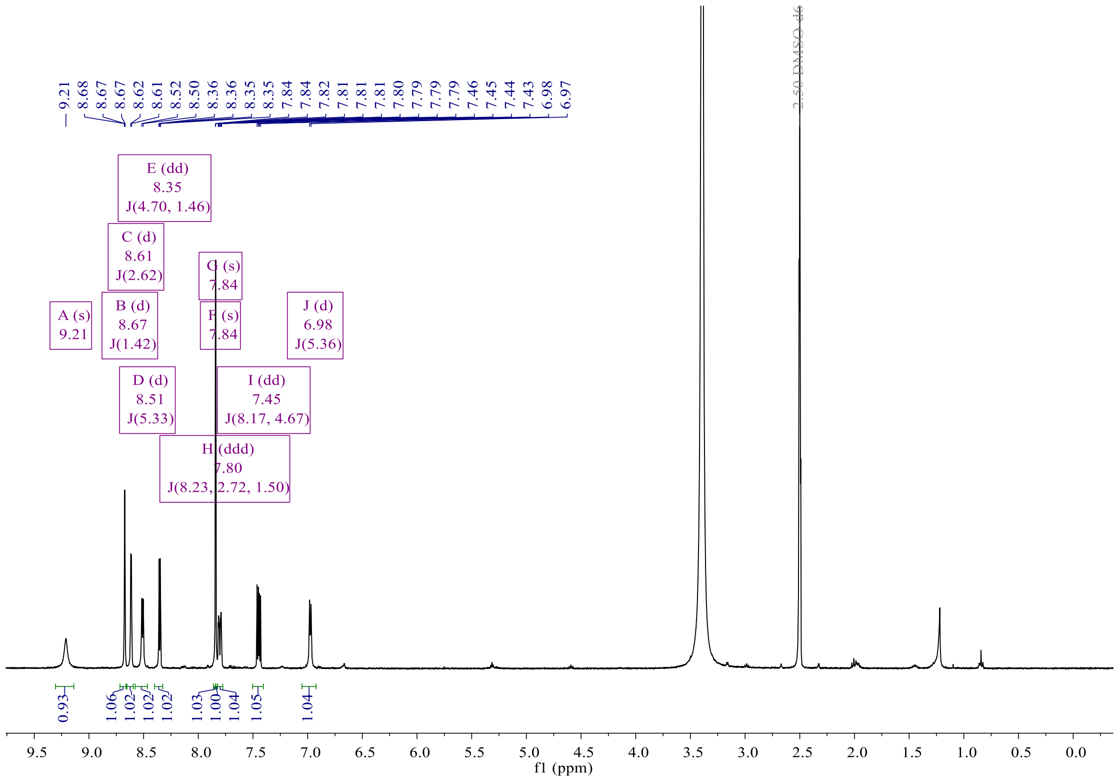
9.21
8.68
8.67
8.67
8.62
8.61
8.52
8.50
8.36
8.36
8.35
8.35
7.84
7.84
7.82
7.81
7.81
7.81
7.80
7.79
7.79
7.79
7.46
7.45
7.44
7.43
6.98
6.97
2.50 DMSO-d6
E (dd) 8.35 J(4.70, 1.46)
C (d) 8.61 J(2.62)
G (s) 7.84
A (s) 9.21
B (d) 8.67 J(1.42)
F (s) 7.84
J (d) 6.98 J(5.36)
D (d) 8.51 J(5.33)
I (dd) 7.45 J(8.17, 4.67)
H (ddd) 7.80 J(8.23, 2.72, 1.50)
0.93
1.06
1.02
1.02
1.02
1.03
1.00
1.04
1.05
1.04
f1 (ppm)

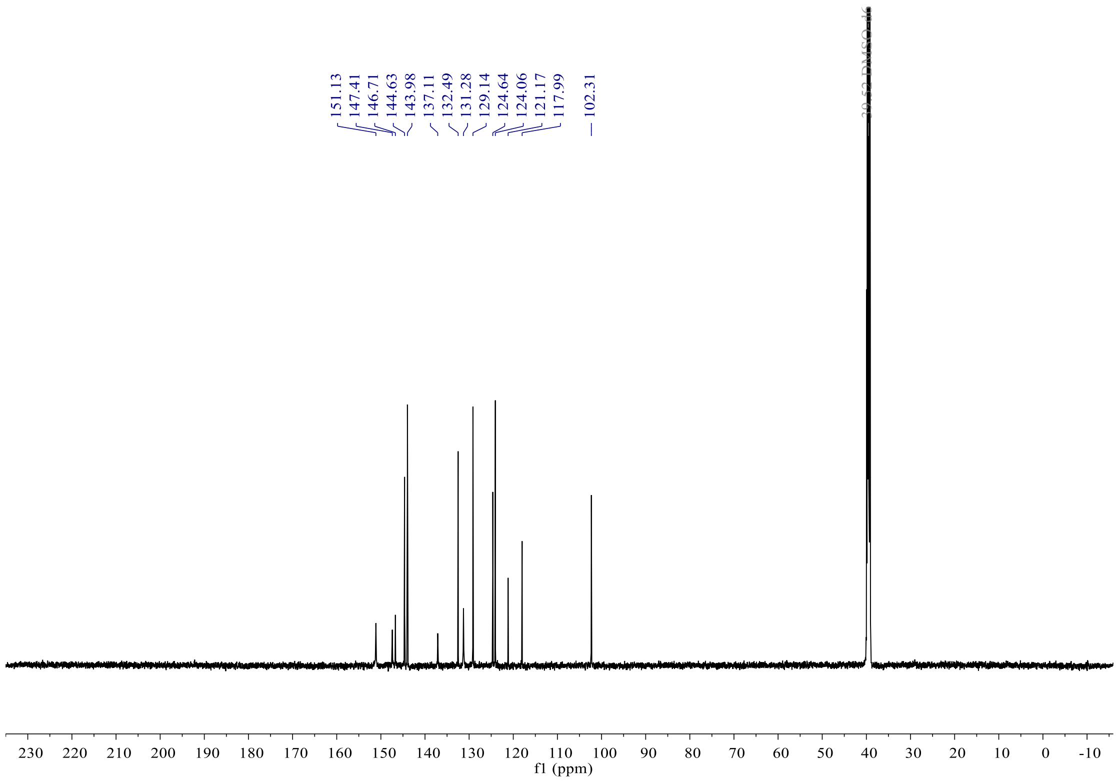
151.13
147.41
146.71
144.63
143.98
137.11
132.49
131.28
129.14
124.64
124.06
121.17
117.99
102.31
f1 (ppm)

Analysis Report

Graph

**Sample Name**	ftt-11812-013
**Application Name**	Admin App (Administrator)
**Method Name**	User1
**Configuration Name**	Configuration 1
**Version**	26
**Data Instrument Name**	Detector
**Data Channel Name**	156 Channel 1
**Notes**	
**Injection Number**	4

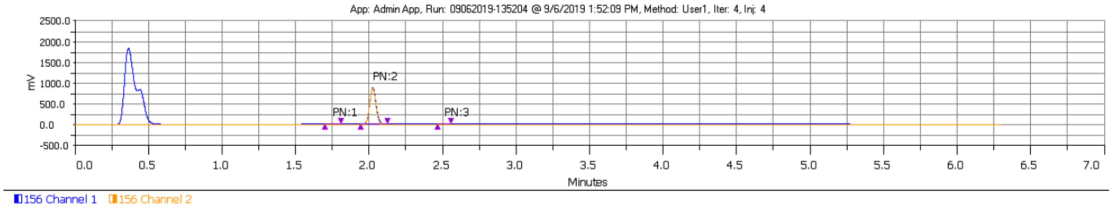

Sample Table

Injection Number	Sample Name	Sample Location	Peak Name	Retention Time (min)	Area (uVmin x100)	Area %	Height (mV)	Plate Number	
4	ftt-11812-013	Sample Zone->45	1	1.753	50196.6667	1.138	10.022	7396.13	
4	ftt-11812-013	Sample Zone->45	2	2.027	4328040	98.159	864.376	10440.749	
4	ftt-11812-013	Sample Zone->45	3	2.512	30972.5	0.702	7.041	18877.663	

D:\data\ftt-11812-013 9/9/2019 3:19:08 PM

ftt-11812-013 #72-87 RT: 1.25-1.51 AV: 16 SB: 32 1.20-1.40 , 1.69-2.00 NL: 5.41E6
F: + c ESI Full ms [105.00-1200.00]

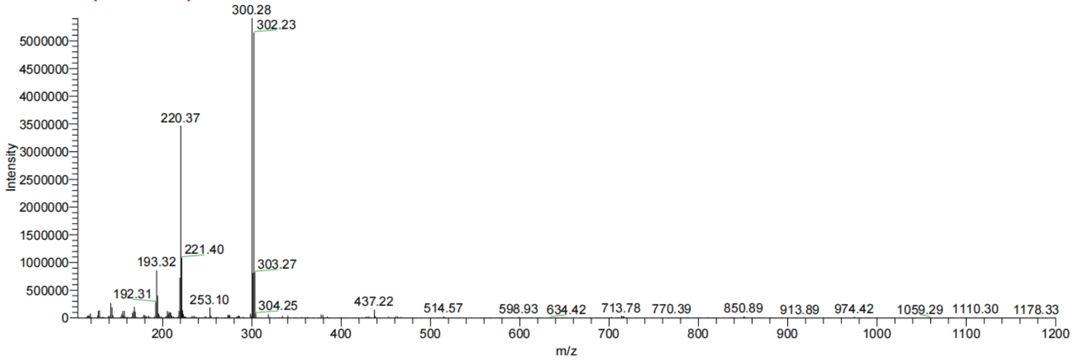

1.9 Compound 9

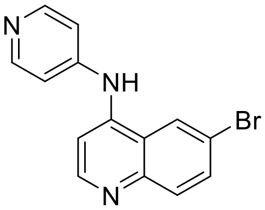

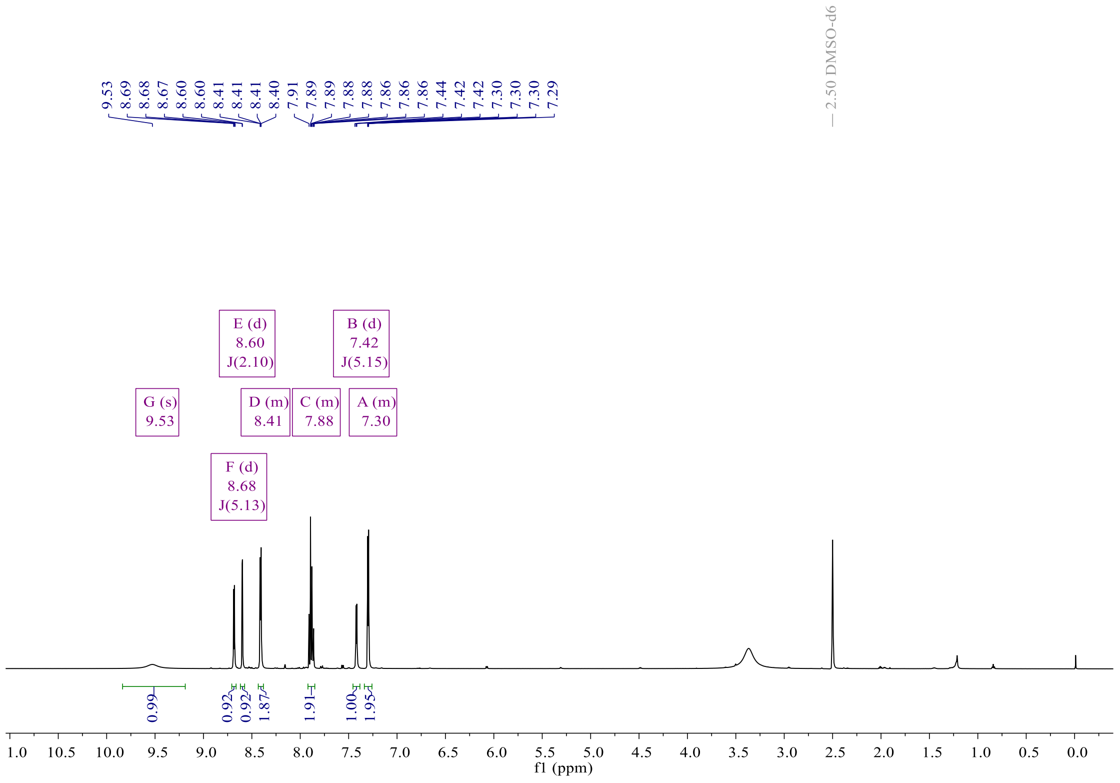

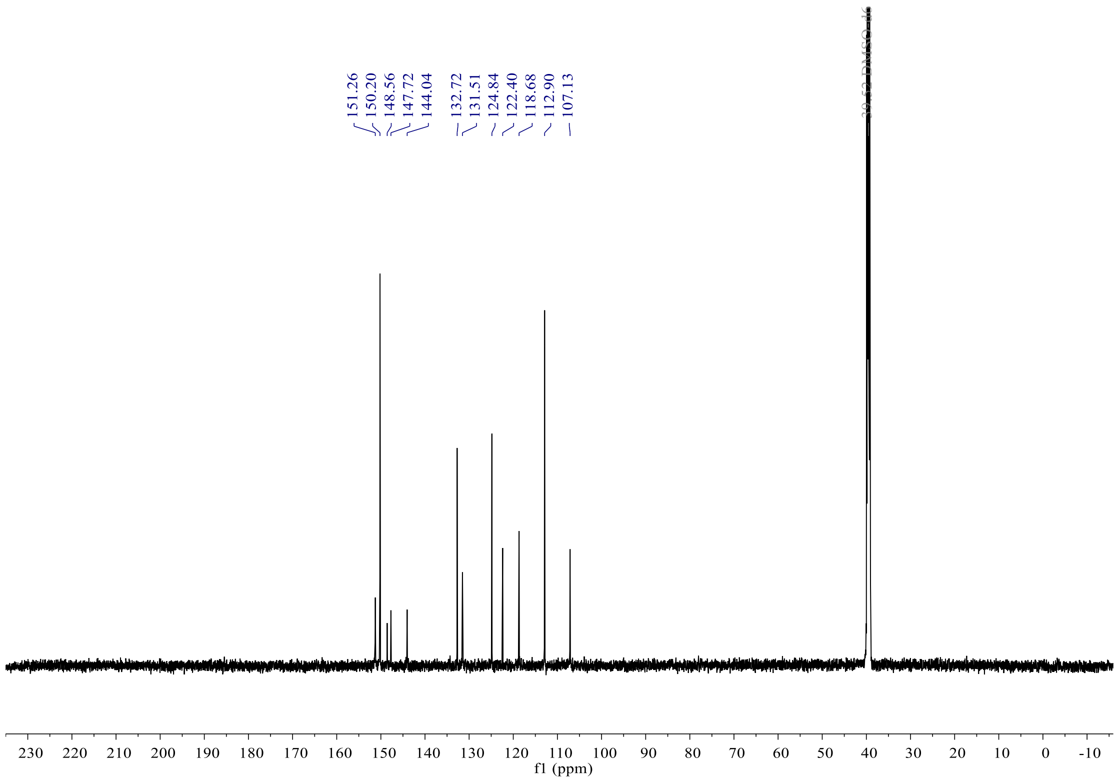

Analysis Report

Graph

**Sample Name**	ftt-11812-014r
**Application Name**	Admin App (Administrator)
**Method Name**	User1
**Configuration Name**	Configuration 1
**Version**	26
**Data Instrument Name**	Detector
**Data Channel Name**	156 Channel 1
**Notes**	
**Injection Number**	3

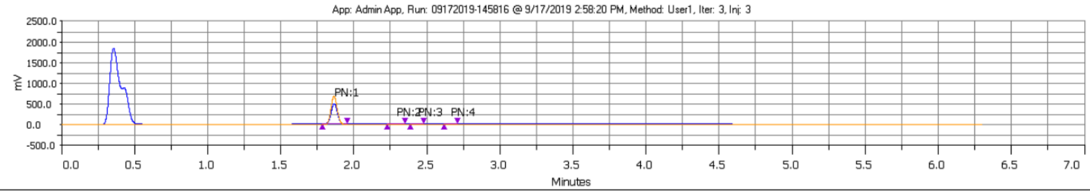

156 Channel 1 156 Channel 2

Sample Table

Injection Number	Sample Name	Sample Location	Peak Name	Retention Time (min)	Area (uVmin x100)	Area %	Height (mV)	Plate Number
3	ftt-11812-014r	Sample Zone->3	1	1.868	2454015.4167	94.595	489.796	8826.427
3	ftt-11812-014r	Sample Zone->3	2	2.292	94725.8333	3.651	18.089	11651.632
3	ftt-11812-014r	Sample Zone->3	3	2.436	27223.3333	1.049	6.037	16929.412
3	ftt-11812-014r	Sample Zone->3	4	2.661	18266.6667	0.704	3.487	13750.912

D:\data\ftt-11812-014 9/9/2019 3:21:56 PM

ftt-11812-014 #71-88 RT: 1.23-1.53 AV: 18 SB: 2 2.69 , 2.69 NL: 4.45E6
F: + c ESI Full ms [105.00-1200.00]

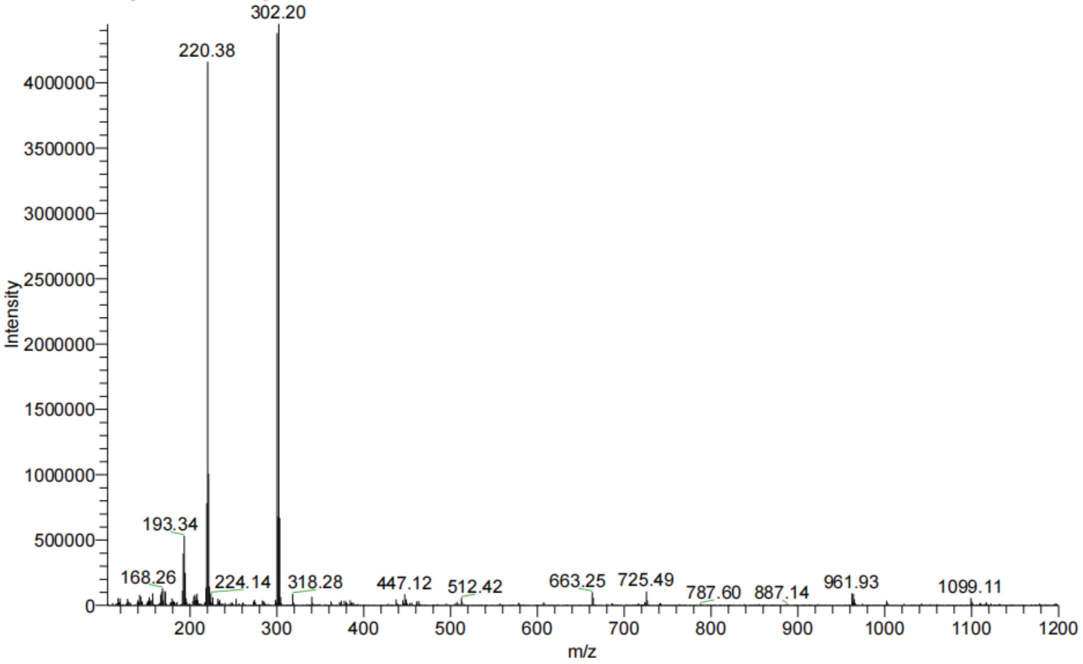

1.2 Compound 10

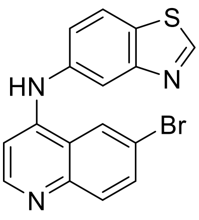

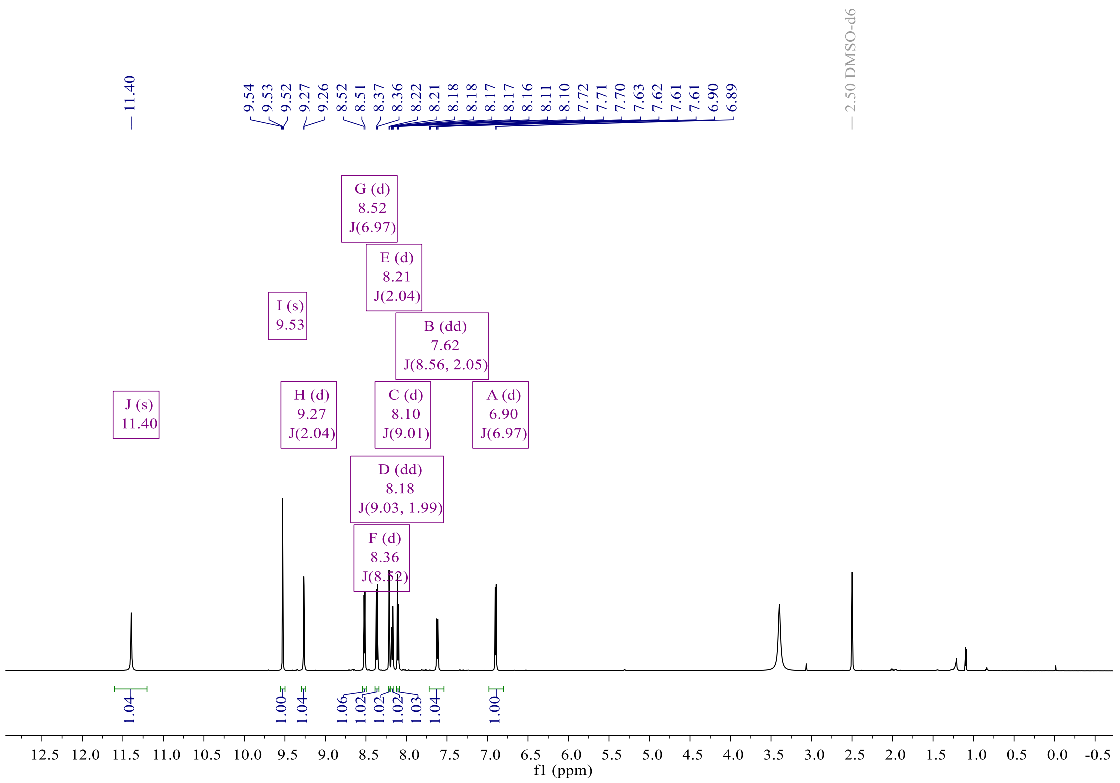

11.40
9.54
9.53
9.52
9.27
9.26
8.52
8.51
8.37
8.36
8.22
8.21
8.18
8.18
8.17
8.17
8.16
8.11
8.10
7.72
7.71
7.70
7.63
7.62
7.61
7.61
6.90
6.89
2.50 DMSO-d6
G (d) 8.52 J(6.97)
E (d) 8.21 J(2.04)
I (s) 9.53
B (dd) 7.62 J(8.56, 2.05)
J (s) 11.40
H (d) 9.27 J(2.04)
C (d) 8.10 J(9.01)
A (d) 6.90 J(6.97)
D (dd) 8.18 J(9.03, 1.99)
F (d) 8.36 J(8.52)
1.04
1.00
1.04
1.06
1.02
1.02
1.02
1.03
1.04
1.00
12.5 12.0 11.5 11.0 10.5 10.0 9.5 9.0 8.5 8.0 7.5 7.0 6.5 6.0 5.5 5.0 4.5 4.0 3.5 3.0 2.5 2.0 1.5 1.0 0.5 0.0 -0.5
f1 (ppm)

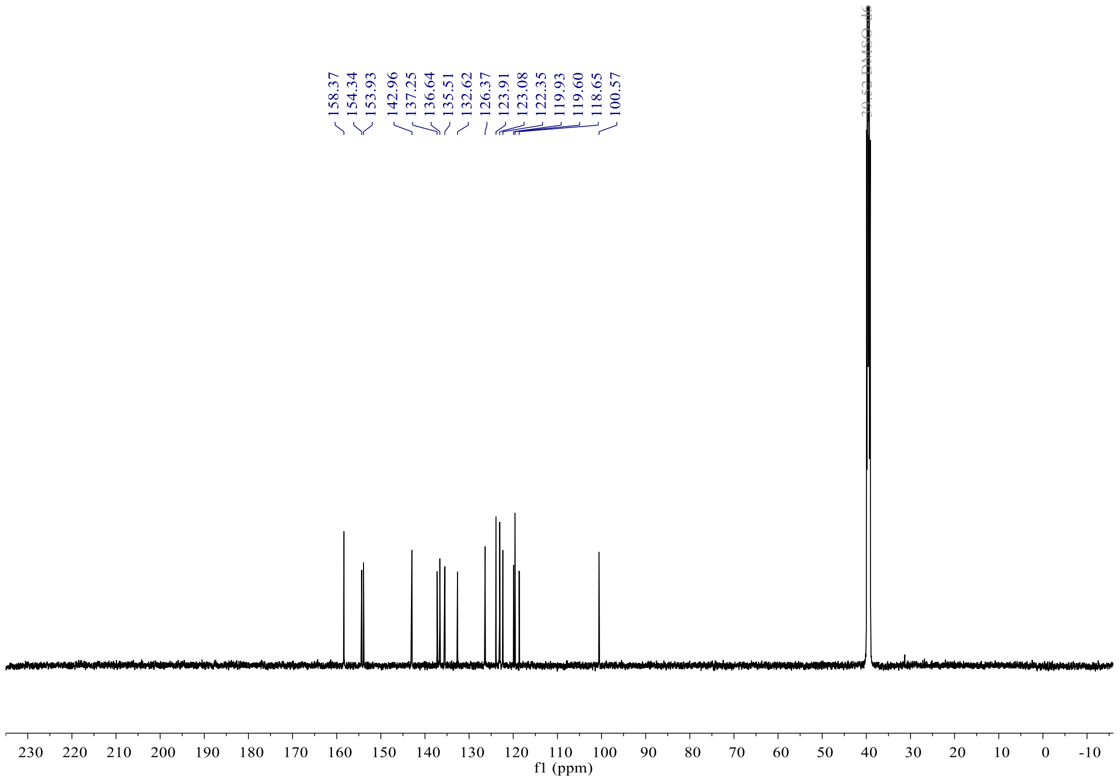

Analysis Report

Graph

**Sample Name**	ftt-11812-027
**Application Name**	Admin App (Administrator)
**Method Name**	User2
**Configuration Name**	Configuration 1
**Version**	23
**Data Instrument Name**	Detector
**Data Channel Name**	156 Channel 1
**Notes**	
**Injection Number**	8

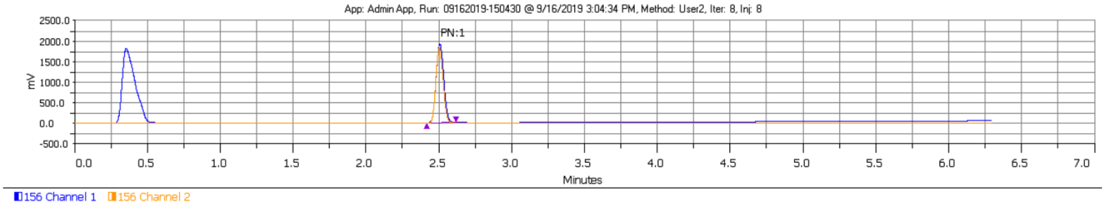

Sample Table

Injection Number	Sample Name	Sample Location	Peak Name	Retention Time (min)	Area (uVmin x100)	Area %	Height (mV)	Plate Number	
8	ftt-11812-027	Sample Zone->40	1	2.51	11764700.8333	100	1912.344	10097.586	

Figure 2. HPLC spectrum of compound **10**

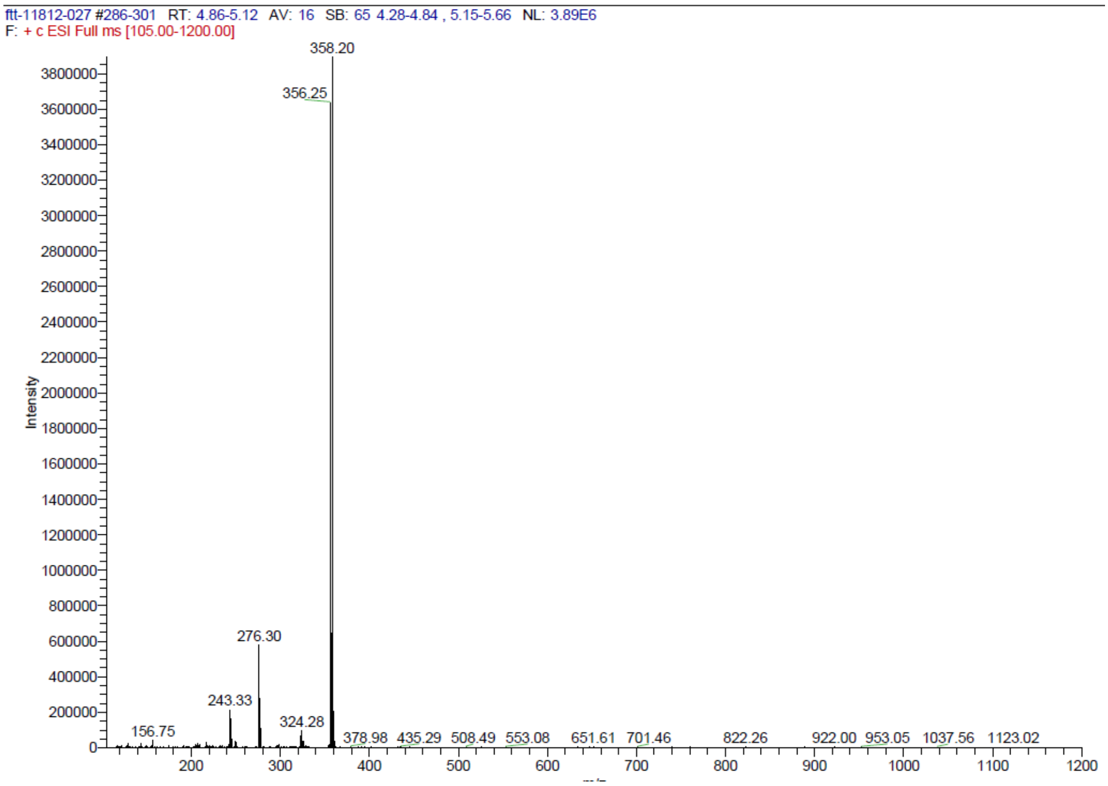

Figure 3. MS spectrum of compound **10**

1.11 Compound 11

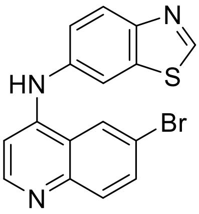

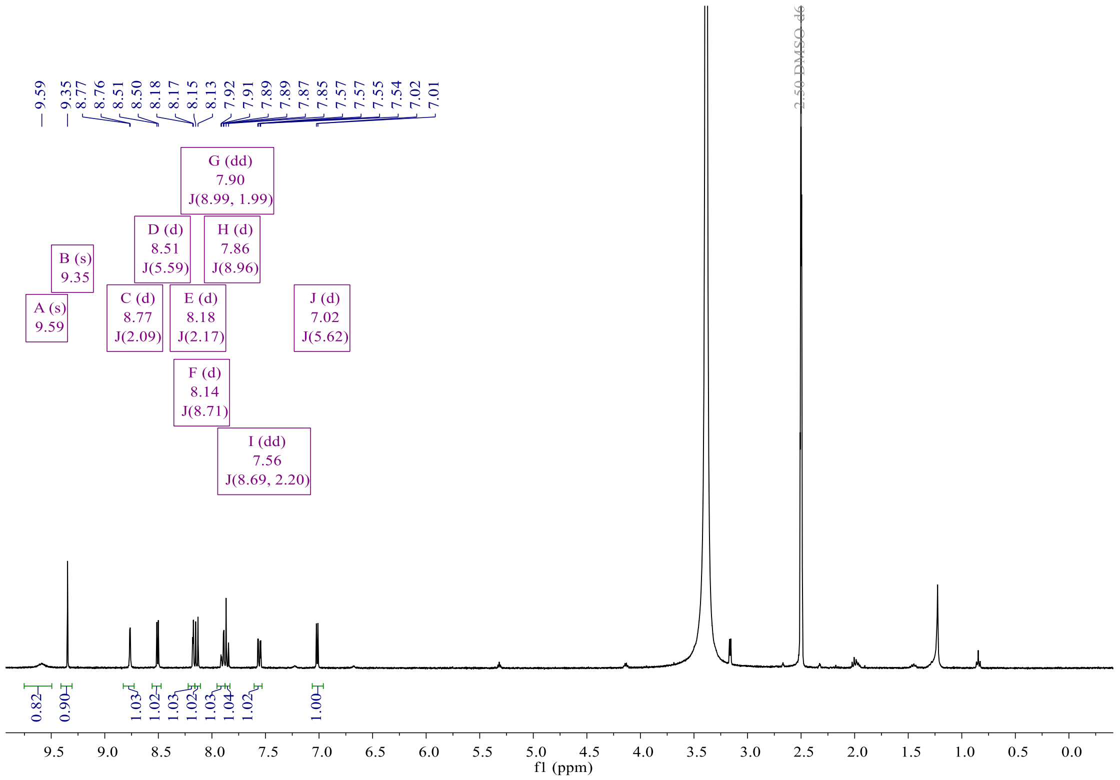
9.59
9.35
8.77
8.76
8.51
8.50
8.18
8.17
8.15
8.13
7.92
7.91
7.89
7.89
7.87
7.85
7.57
7.55
7.54
7.02
7.01
2.50 DMSO-d6
G (dd) 7.90 J(8.99, 1.99)
D (d) 8.51 J(5.59)
H (d) 7.86 J(8.96)
B (s) 9.35
A (s) 9.59
C (d) 8.77 J(2.09)
E (d) 8.18 J(2.17)
J (d) 7.02 J(5.62)
F (d) 8.14 J(8.71)
I (dd) 7.56 J(8.69, 2.20)
0.82
0.90
1.03
1.02
1.03
1.02
1.03
1.04
1.02
1.00
f1 (ppm)

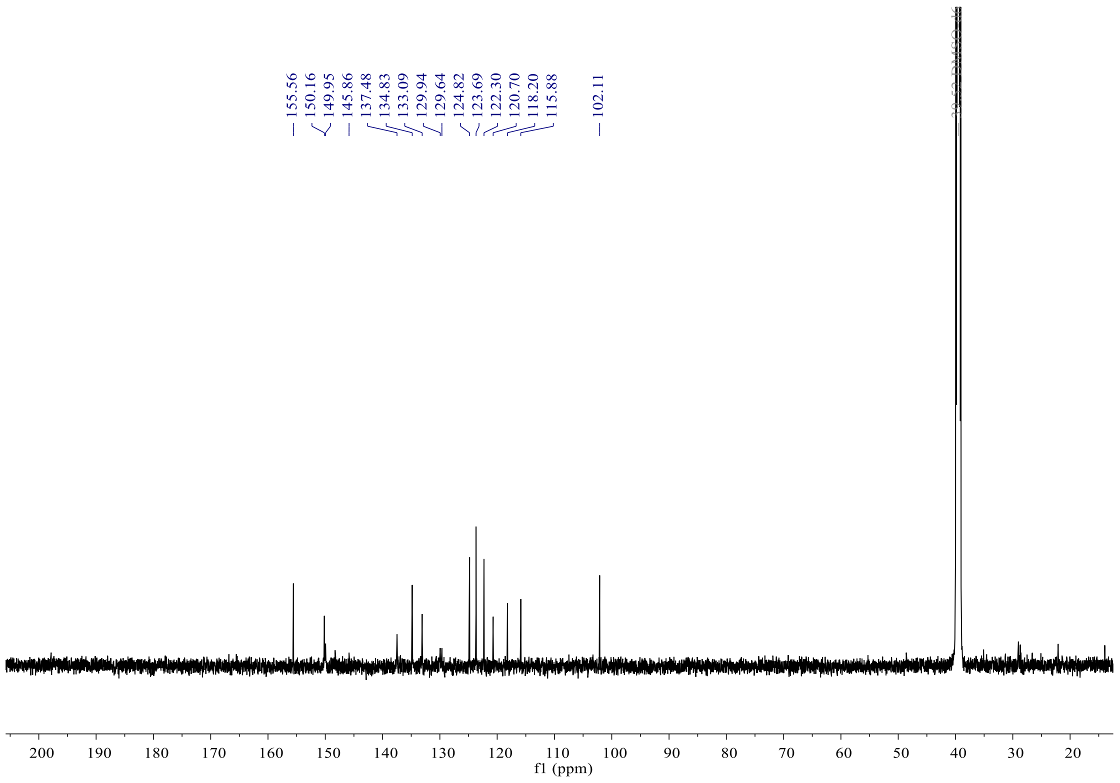
155.56
150.16
149.95
145.86
137.48
134.83
133.09
129.94
129.64
124.82
123.69
122.30
120.70
118.20
115.88
102.11
f1 (ppm)

Analysis Report

Graph

**Sample Name**	ftt-11812-028
**Application Name**	Admin App (Administrator)
**Method Name**	User1
**Configuration Name**	Configuration 1
**Version**	26
**Data Instrument Name**	Detector
**Data Channel Name**	156 Channel 1
**Notes**	
**Injection Number**	9

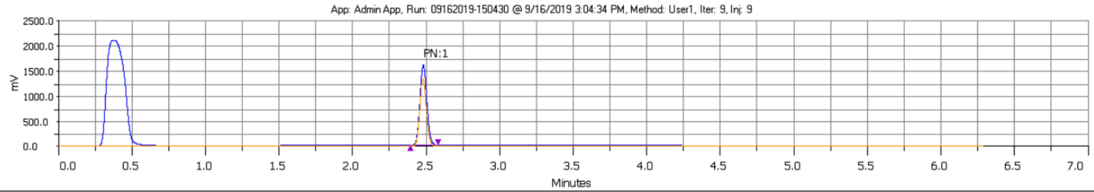

Sample Table

Injection Number	Sample Name	Sample Location	Peak Name	Retention Time (min)	Area (uVmin x100)	Area %	Height (mV)	Plate Number	
9	ftt-11812-028	Sample Zone->41	1	2.483	8775019.5833	100	1600.42	12883.104	

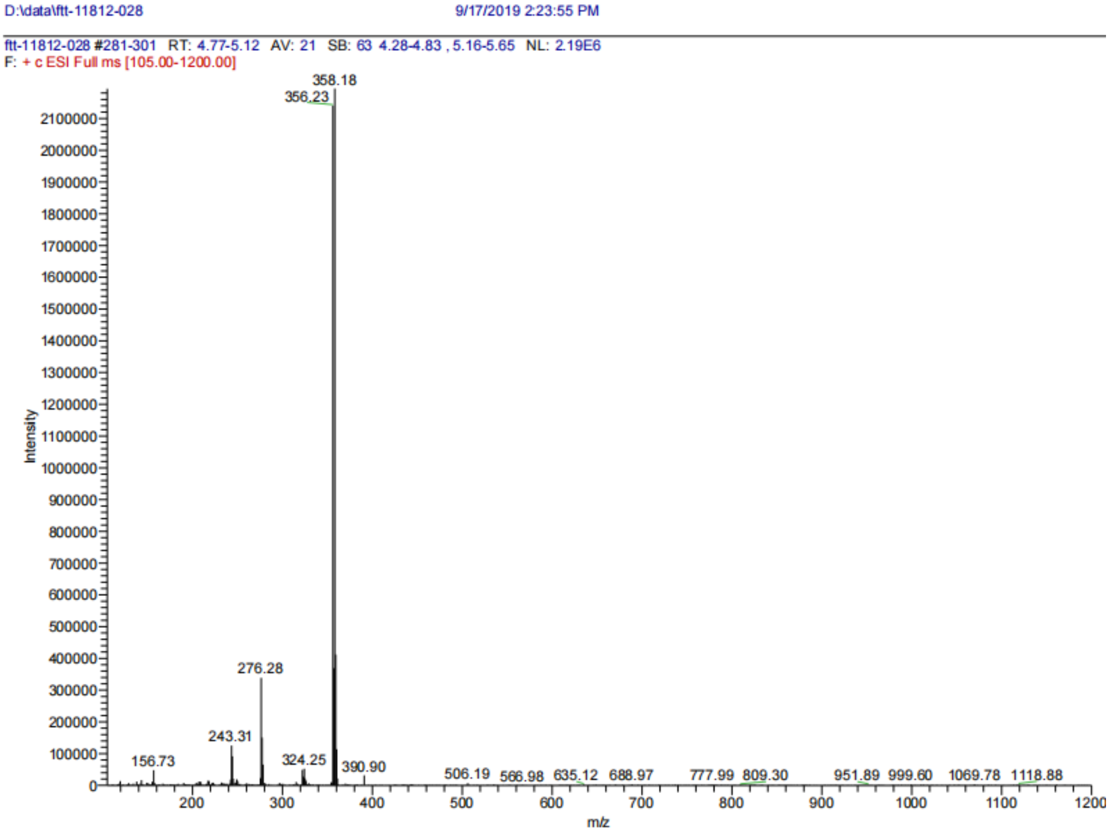

1.12 Compound 12

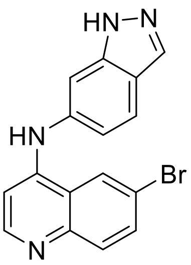

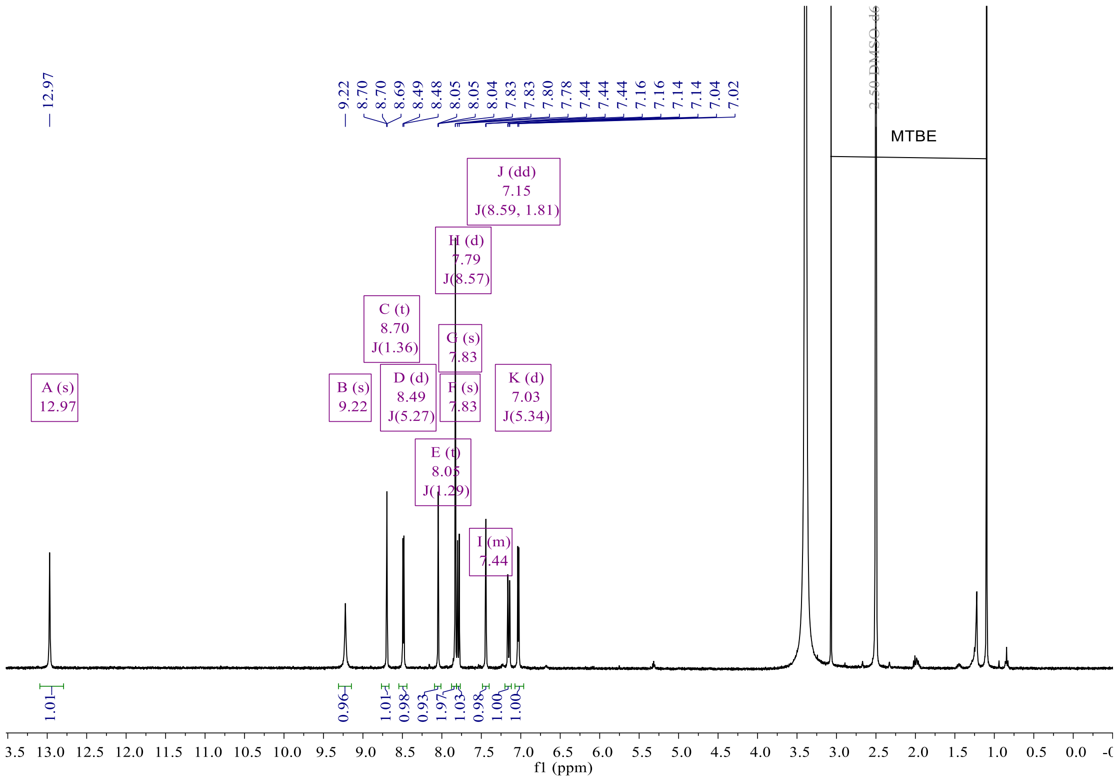

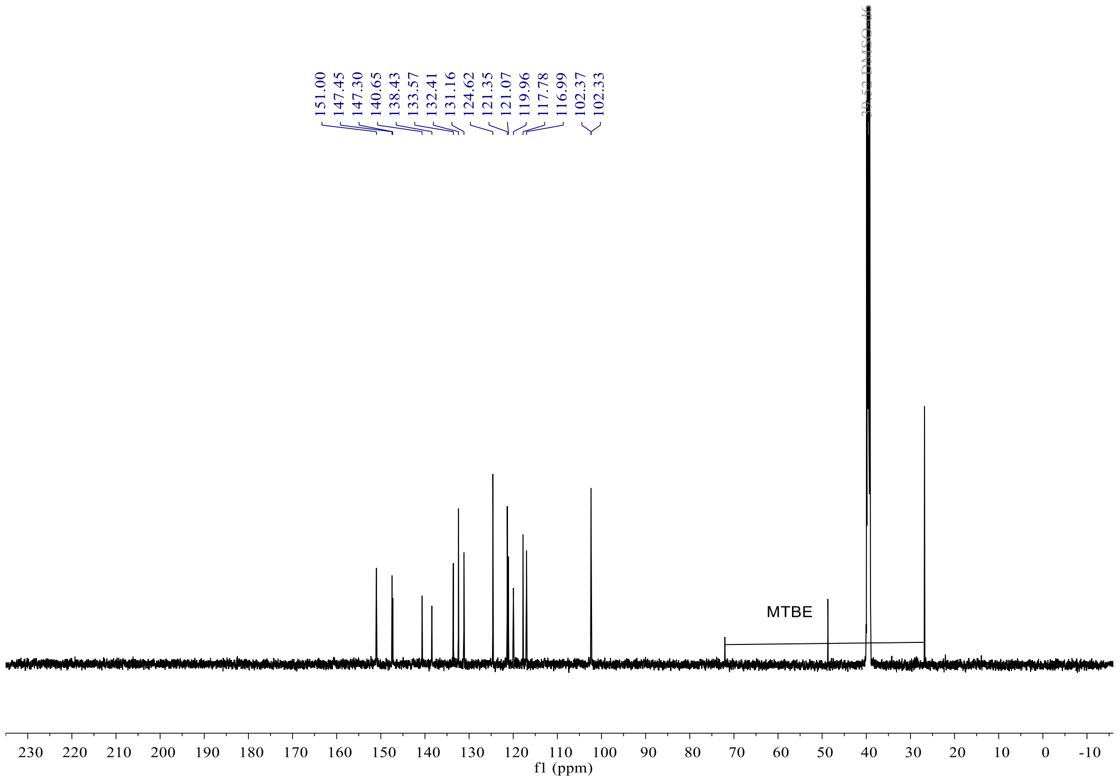

Analysis Report

Graph

Sample Name ftt-11812-032
Application Name Admin App (Administrator)
Method Name User1
Configuration Name Configuration 1
Version 26
Data Instrument Name Detector
Data Channel Name 156 Channel 1
Notes
Injection Number 13

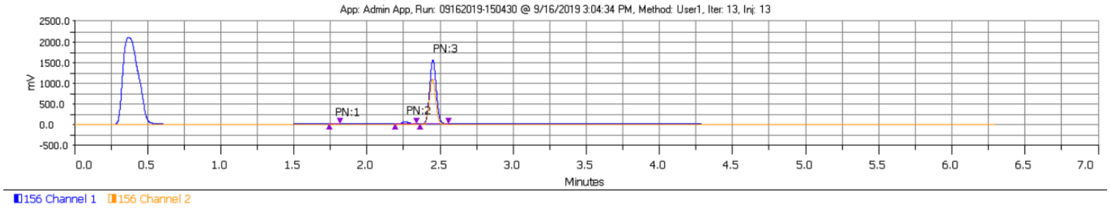

Sample Table

Injection Number	Sample Name	Sample Location	Peak Name	Retention Time (min)	Area (uVmin x100)	Area %	Height (mV)	Plate Number	
13	ftt-11812-032	Sample Zone->45	1	1.781	13430.4167	0.16	3.215	9974.375	
13	ftt-11812-032	Sample Zone->45	2	2.265	298366.6667	3.549	57.34	12197.749	
13	ftt-11812-032	Sample Zone->45	3	2.453	8094119.1667	96.291	1534.297	13561.418	

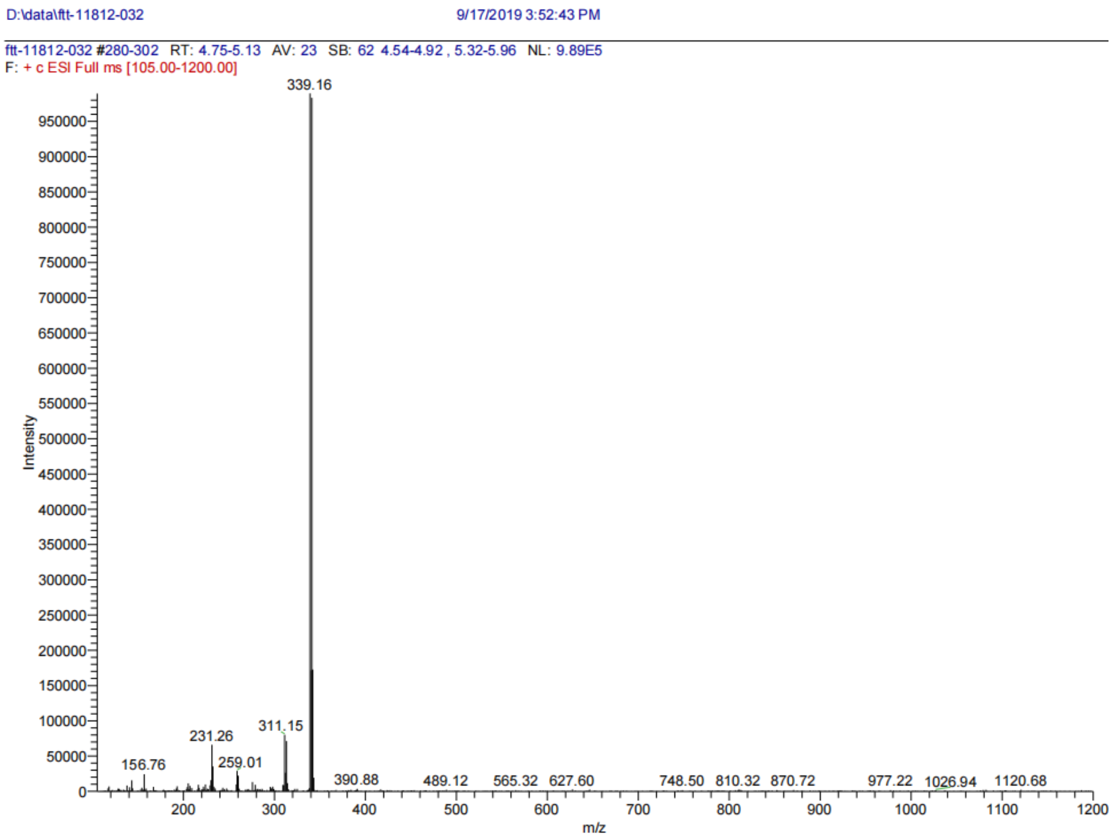

1.13 Compound 13

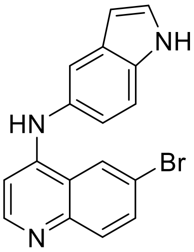

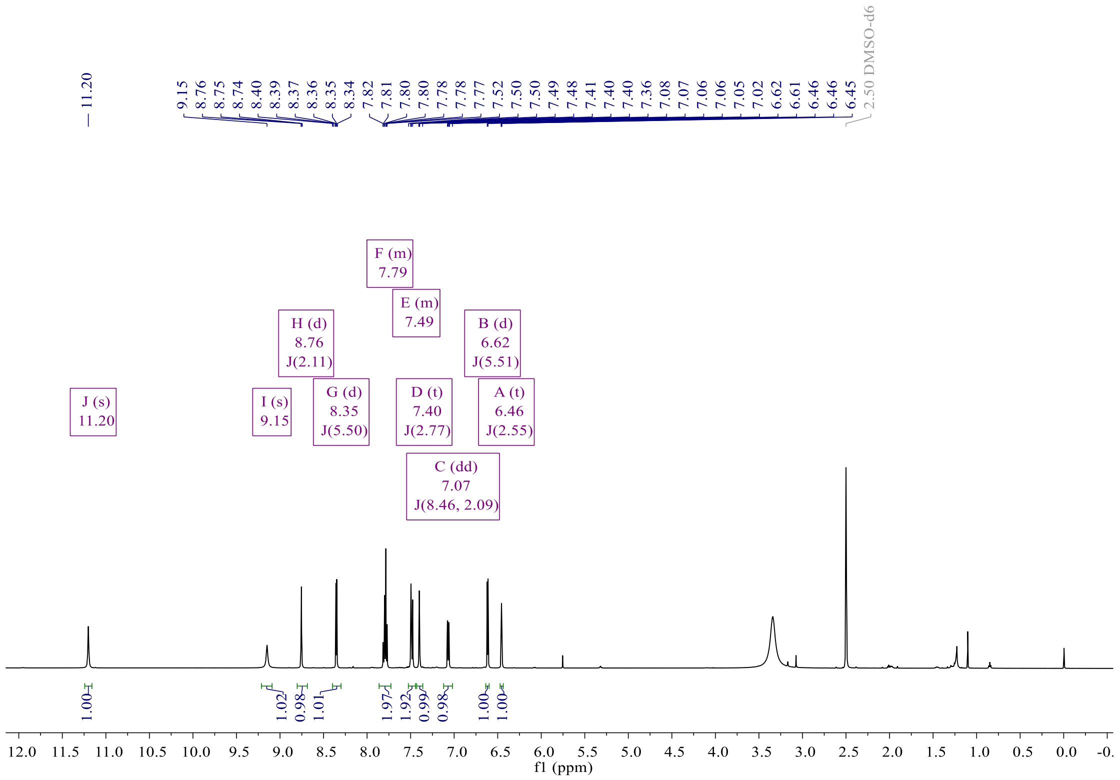
11.20
9.15 8.76 8.75 8.74 8.40 8.39 8.37 8.36 8.35 8.34 7.82 7.81 7.80 7.80 7.78 7.78 7.77 7.52 7.50 7.50 7.49 7.48 7.41 7.40 7.36 7.08 7.07 7.06 7.06 7.05 7.02 6.62 6.61 6.46 6.46 6.45
2.50 DMSO-d6
F (m) 7.79
E (m) 7.49
H (d) 8.76 J(2.11)
B (d) 6.62 J(5.51)
J (s) 11.20
I (s) 9.15
G (d) 8.35 J(5.50)
D (t) 7.40 J(2.77)
A (t) 6.46 J(2.55)
C (dd) 7.07 J(8.46, 2.09)
1.00 1.02 0.98 1.01 1.97 1.92 0.99 0.98 1.00 1.00
f1 (ppm)

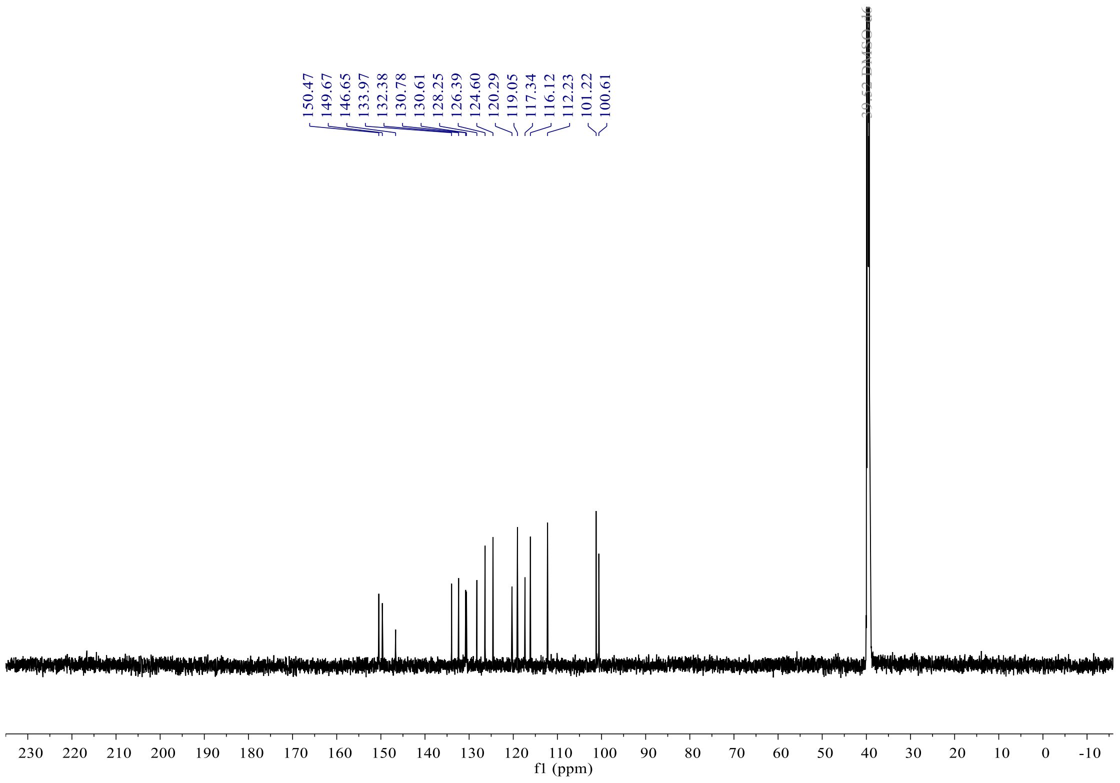
150.47 149.67 146.65 133.97 132.38 130.78 130.61 128.25 126.39 124.60 120.29 119.05 117.34 116.12 112.23 101.22 100.61
f1 (ppm)

Analysis Report

Graph

**Sample Name**	ftt-11812-033
**Application Name**	Admin App (Administrator)
**Method Name**	User2
**Configuration Name**	Configuration 1
**Version**	23
**Data Instrument Name**	Detector
**Data Channel Name**	156 Channel 1
**Notes**	
**Injection Number**	14

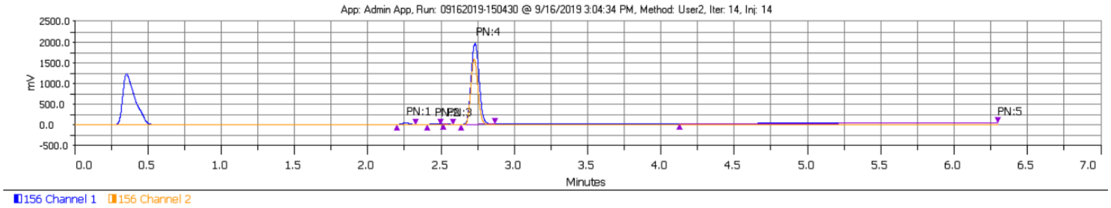

Sample Table

Injection Number	Sample Name	Sample Location	Peak Name	Retention Time (min)	Area (uVmin x100)	Area %	Height (mV)	Plate Number	
14	ftt-11812-033	Sample Zone->46	1	2.26	191196.6667	1.424	38.255	12771.246	
14	ftt-11812-033	Sample Zone->46	2	2.457	40040	0.298	9.314	19031.365	
14	ftt-11812-033	Sample Zone->46	3	2.542	10289.1667	0.077	2.683	23429.113	
14	ftt-11812-033	Sample Zone->46	4	2.734	12395008.75	92.319	1943.029	11278.372	
14	ftt-11812-033	Sample Zone->46	5	5.045	789688.3333	5.882	7.147	34.697	

D:\data\ftt-11812-033 9/17/2019 4:01:28 PM

ftt-11812-033 #290-306 RT: 4.92-5.20 AV: 17 SB: 63 4.46-5.01 , 5.11-5.61 NL: 1.02E6
F: + c ESI Full ms [105.00-1200.00]

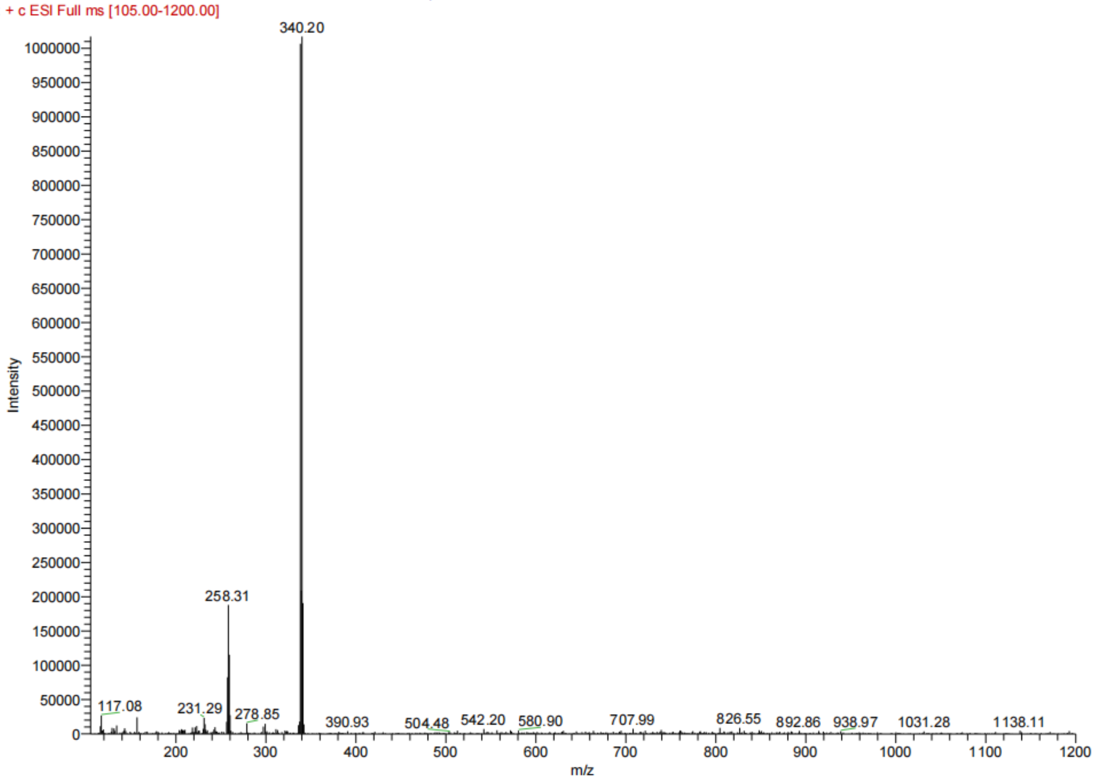

1.3 Compound 14

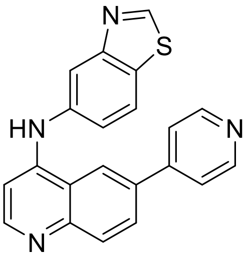

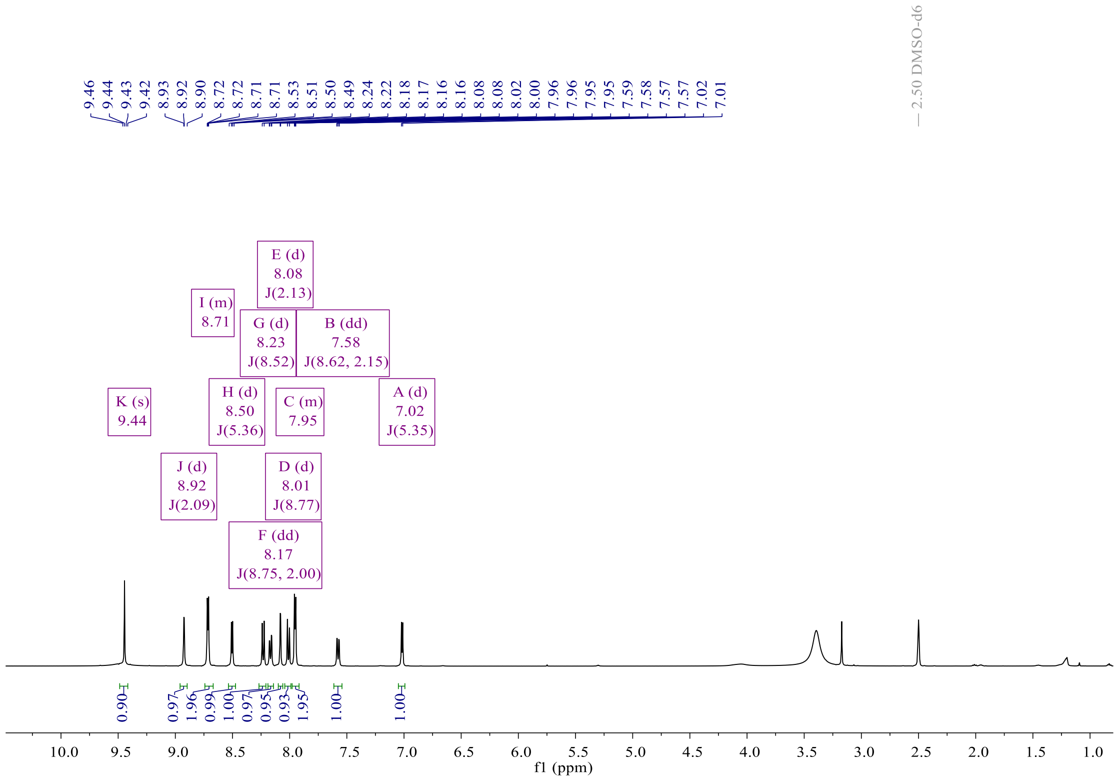

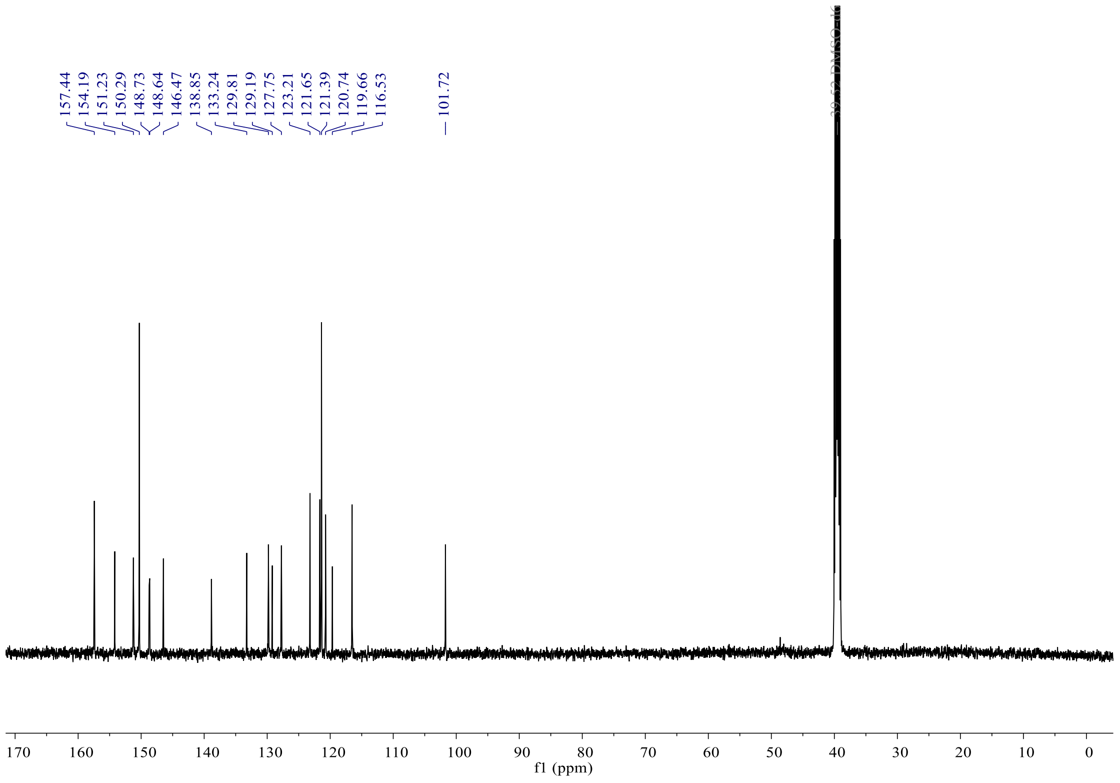

Analysis Report

Graph

**Sample Name**	ftt-10708-063
**Application Name**	Admin App (Administrator)
**Method Name**	User1
**Configuration Name**	Configuration 1
**Version**	48
**Data Instrument Name**	Detector
**Data Channel Name**	156 Channel 1
**Notes**	
**Injection Number**	3

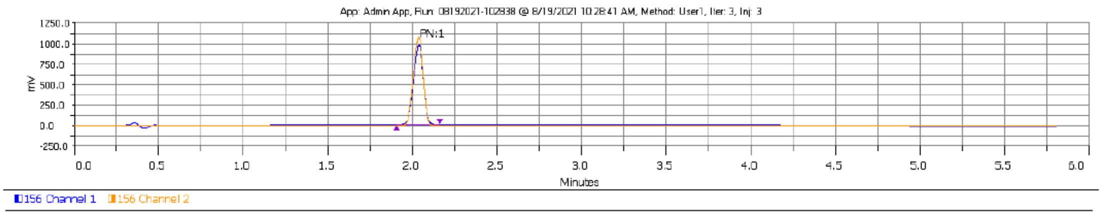

Sample Table

Injection Number	Sample Name	Sample Location	Peak Name	Retention Time (min)	Area (uVmin x100)	Area %	Height (mV)	Plate Number	
3	ftt-10708-063	Sample Zone->11	1	2.04	6562520	100	965.043	5367.193	

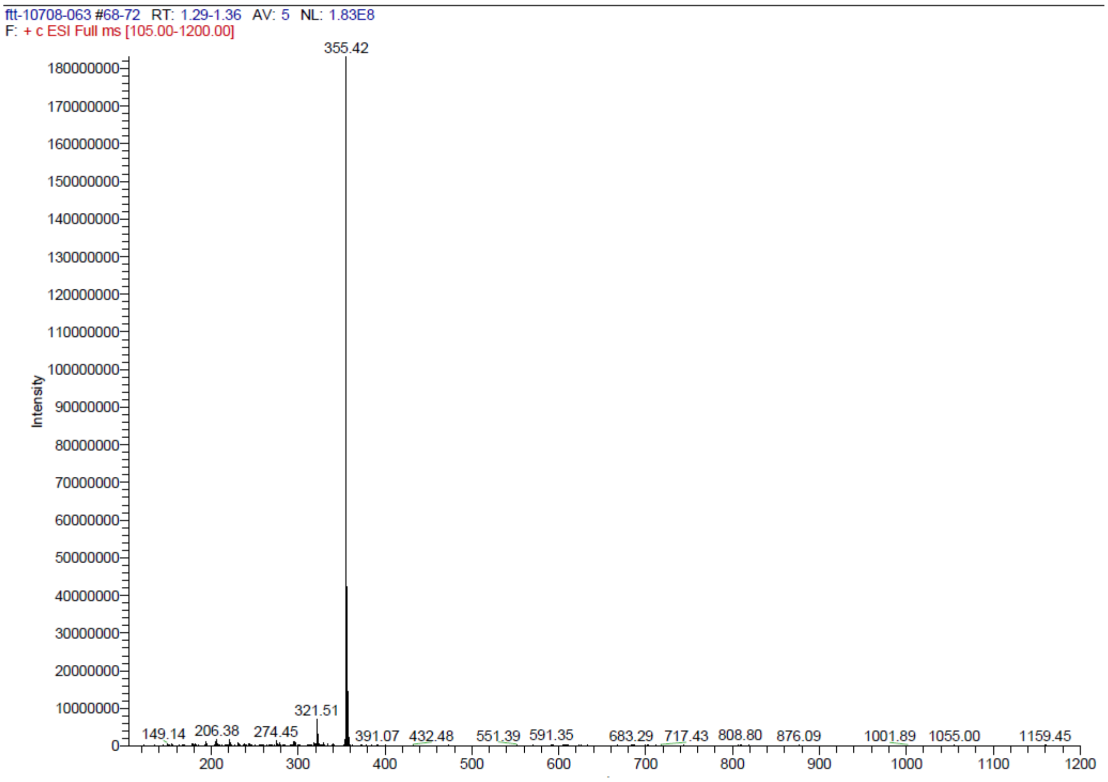

1.15 Compound 15

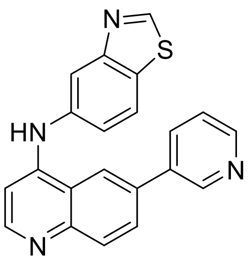

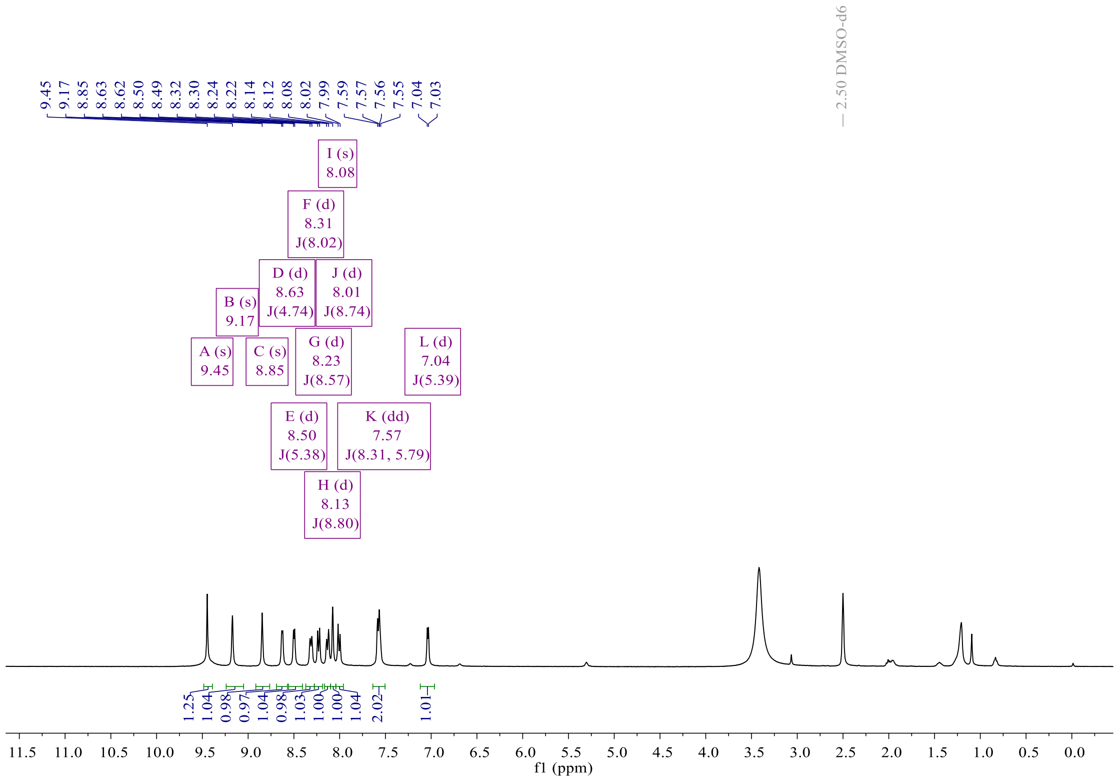

9.45 9.17 8.85 8.63 8.62 8.50 8.49 8.32 8.30 8.24 8.22 8.14 8.12 8.08 8.02 7.99 7.59 7.57 7.56 7.55 7.04 7.03
2.50 DMSO-d6
I (s) 8.08
F (d) 8.31 J(8.02)
D (d) 8.63 J(4.74)
J (d) 8.01 J(8.74)
B (s) 9.17
A (s) 9.45
C (s) 8.85
G (d) 8.23 J(8.57)
L (d) 7.04 J(5.39)
E (d) 8.50 J(5.38)
K (dd) 7.57 J(8.31, 5.79)
H (d) 8.13 J(8.80)
1.25 1.04 0.98 0.97 1.04 0.98 1.03 1.00 1.00 1.04 2.02 1.01
f1 (ppm)

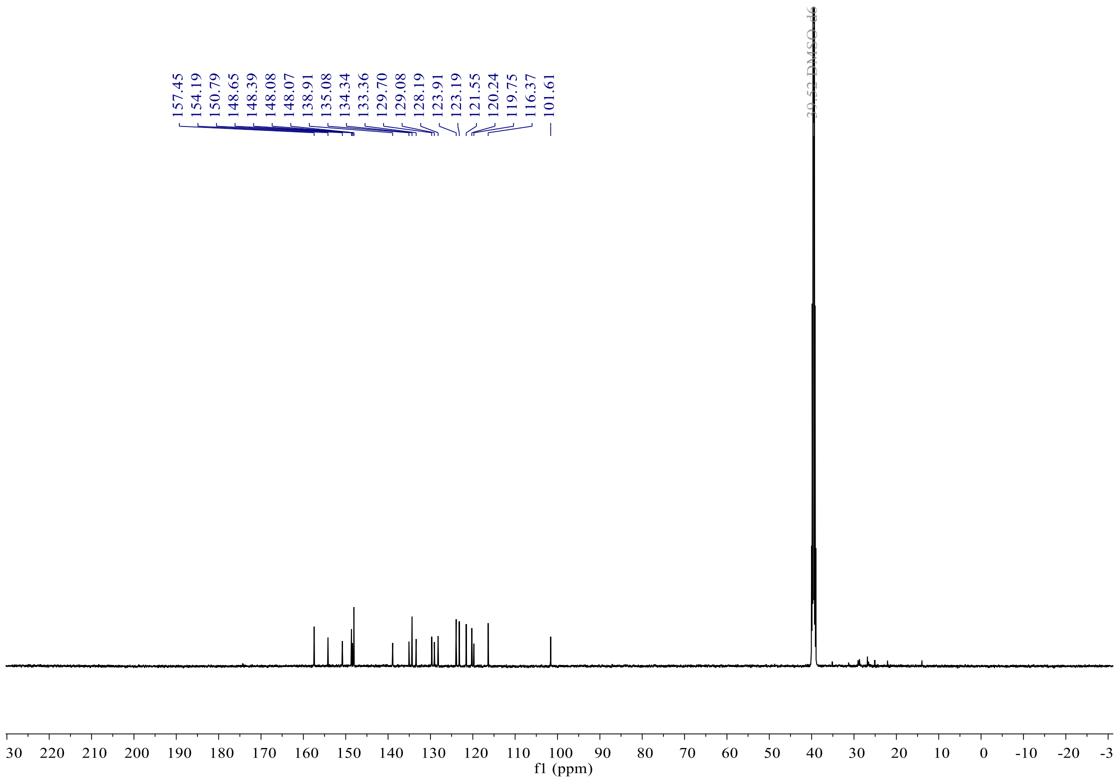

157.45 154.19 150.79 148.65 148.39 148.08 148.07 138.91 135.08 134.34 133.36 129.70 129.08 128.19 123.91 123.19 121.55 120.24 119.75 116.37 101.61
f1 (ppm)

Analysis Report

Graph

**Sample Name**	ftt-10708-064
**Application Name**	Admin App (Administrator)
**Method Name**	User2
**Configuration Name**	Configuration 1
**Version**	42
**Data Instrument Name**	Detector
**Data Channel Name**	156 Channel 1
**Notes**	
**Injection Number**	5

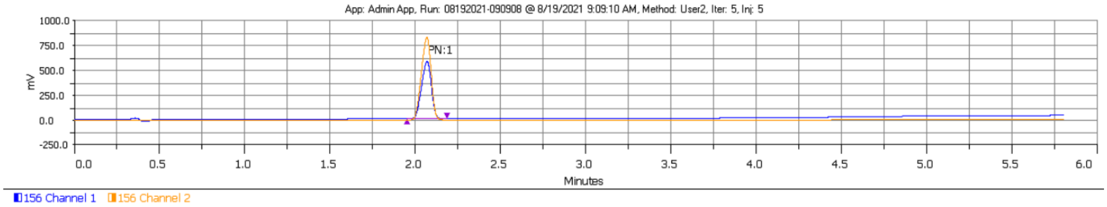

Sample Table

Injection Number	Sample Name	Sample Location	Peak Name	Retention Time (min)	Area (uVmin x100)	Area %	Height (mV)	Plate Number	
5	ftt-10708-064	Sample Zone->12	1	2.071	3889182.9167	100	577.697	5638.343	

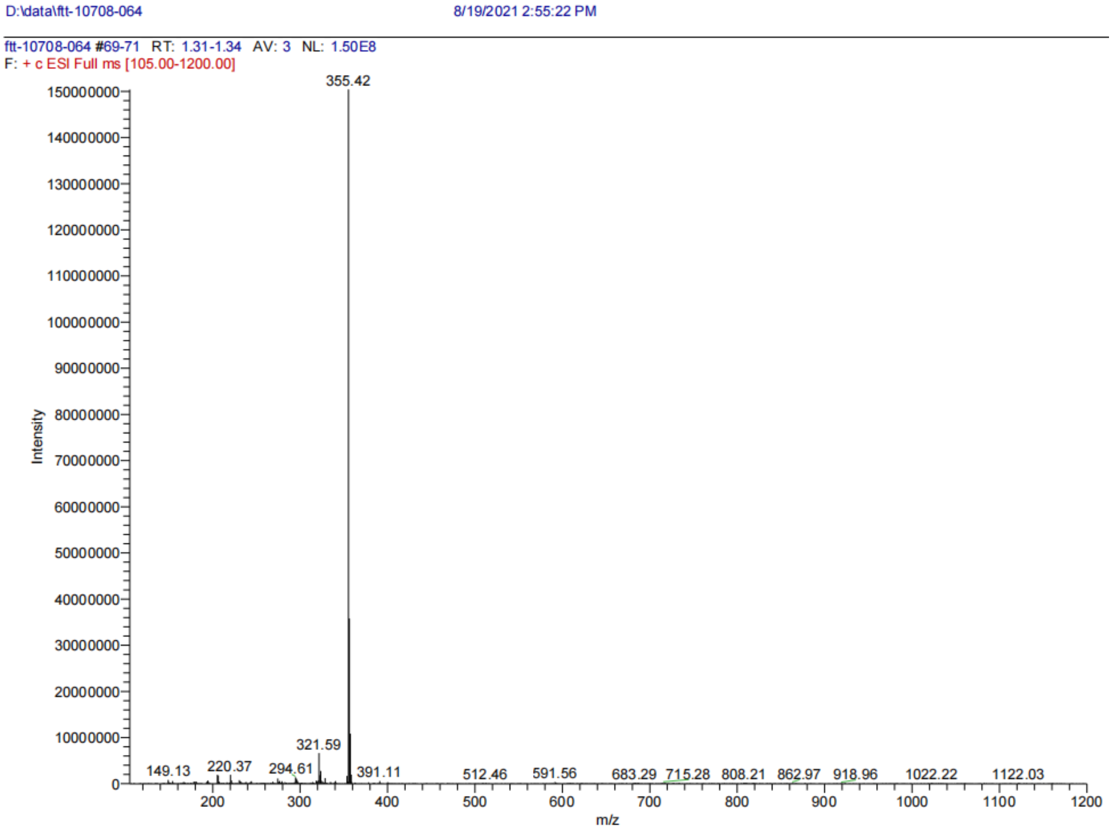

1.16 Compound 16

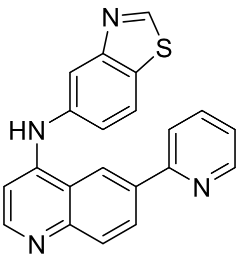

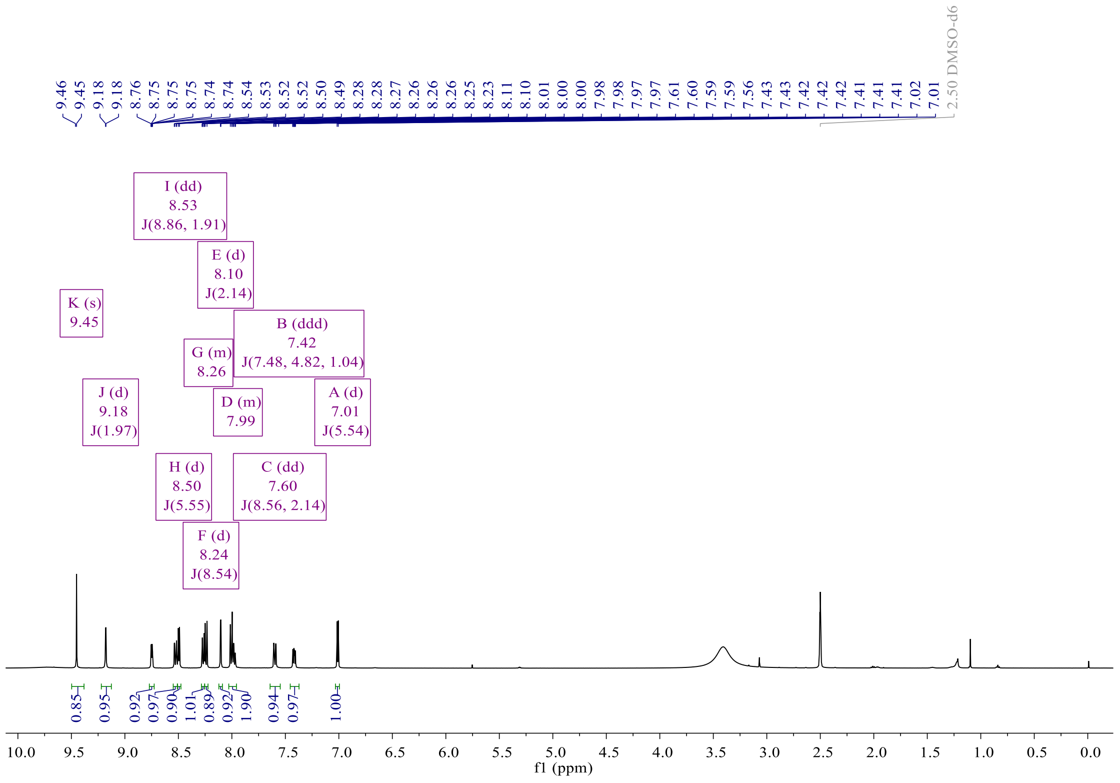

9.46
9.45
9.18
9.18
8.76
8.75
8.75
8.75
8.74
8.74
8.54
8.53
8.52
8.52
8.50
8.49
8.28
8.28
8.27
8.26
8.26
8.26
8.25
8.23
8.11
8.10
8.01
8.00
8.00
7.98
7.98
7.97
7.97
7.61
7.60
7.59
7.59
7.56
7.43
7.43
7.42
7.42
7.42
7.41
7.41
7.41
7.02
7.01
2.50 DMSO-d6
I (dd) 8.53 J(8.86, 1.91)
E (d) 8.10 J(2.14)
K (s) 9.45
B (ddd) 7.42 J(7.48, 4.82, 1.04)
G (m) 8.26
J (d) 9.18 J(1.97)
D (m) 7.99
A (d) 7.01 J(5.54)
H (d) 8.50 J(5.55)
C (dd) 7.60 J(8.56, 2.14)
F (d) 8.24 J(8.54)
0.85
0.95
0.92
0.97
0.90
1.01
0.89
0.92
1.90
0.94
0.97
1.00
10.0
9.5
9.0
8.5
8.0
7.5
7.0
6.5
6.0
5.5
5.0
4.5
4.0
3.5
3.0
2.5
2.0
1.5
1.0
0.5
0.0
f1 (ppm)

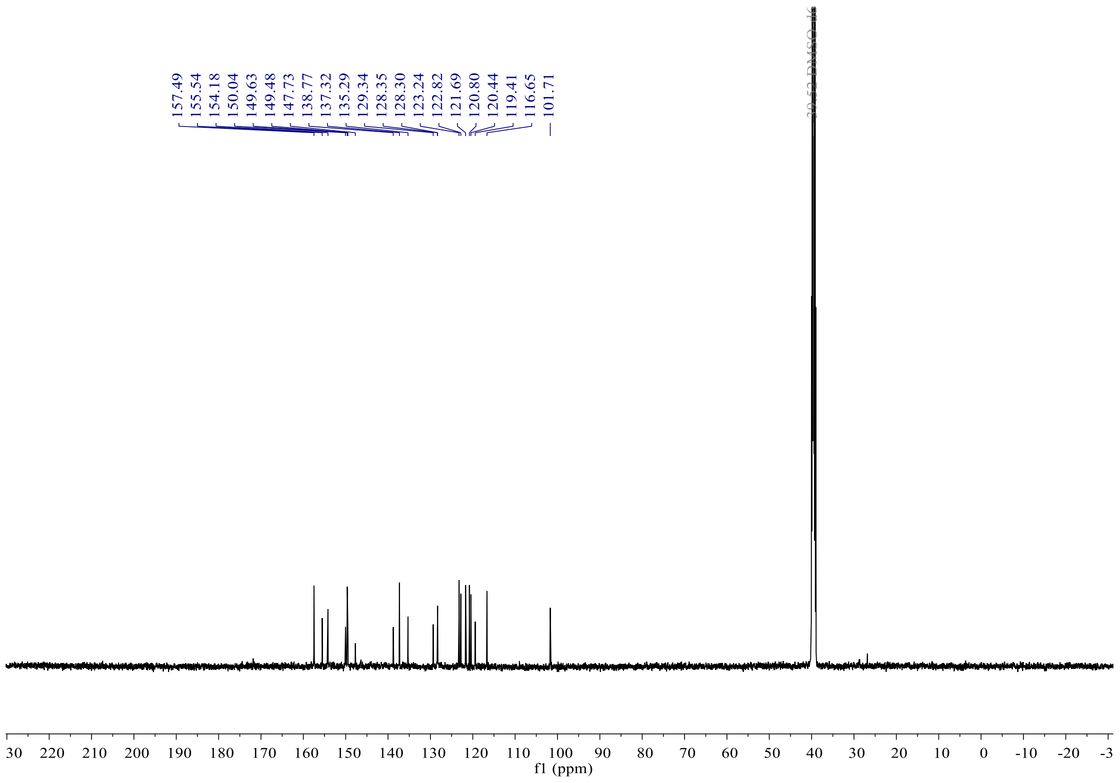

157.49
155.54
154.18
150.04
149.63
149.48
147.73
138.77
137.32
135.29
129.34
128.35
128.30
123.24
122.82
121.69
120.80
120.44
119.41
116.65
101.71
220
210
200
190
180
170
160
150
140
130
120
110
100
90
80
70
60
50
40
30
20
10
0
-10
-20
f1 (ppm)

Analysis Report

Graph

**Sample Name**	ftt-10708-065
**Application Name**	Admin App (Administrator)
**Method Name**	User1
**Configuration Name**	Configuration 1
**Version**	48
**Data Instrument Name**	Detector
**Data Channel Name**	156 Channel 1
**Notes**	
**Injection Number**	2

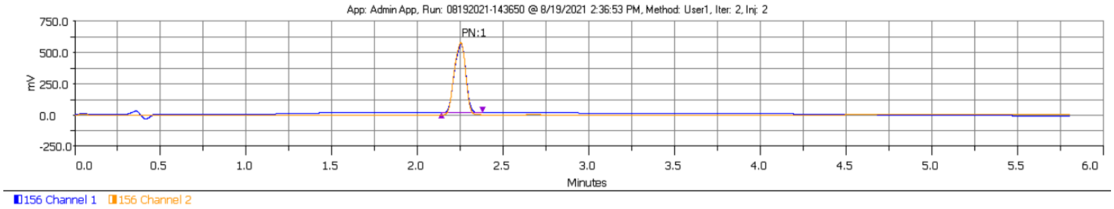

Sample Table

Injection Number	Sample Name	Sample Location	Peak Name	Retention Time (min)	Area (uVmin x100)	Area %	Height (mV)	Plate Number	
2	ftt-10708-065	Sample Zone->21	1	2.255	4238786.6667	100	547.178	4856.74	

D:\data\ftt-10708-065 8/19/2021 2:58:15 PM

ftt-10708-065 #71-72 RT: 1.33-1.35 AV: 2 NL: 2.23E8
F: + c ESI Full ms [105.00-1200.00]

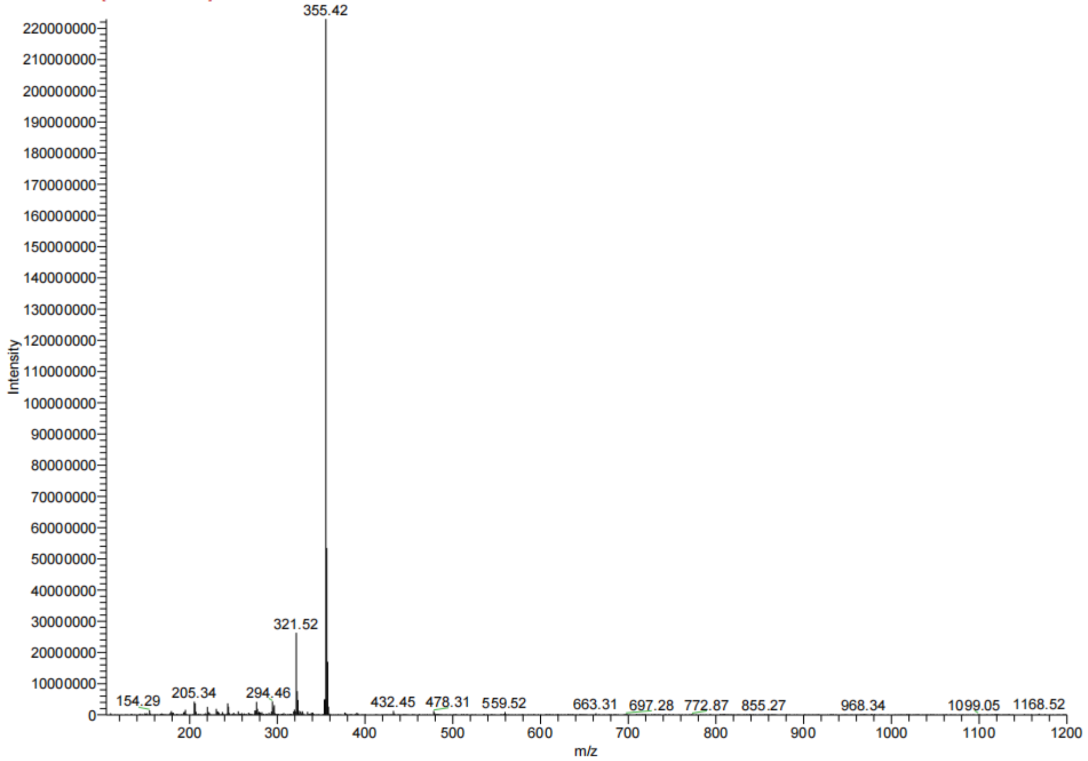

1.17 Compound 17

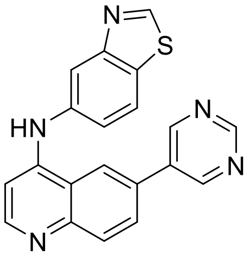

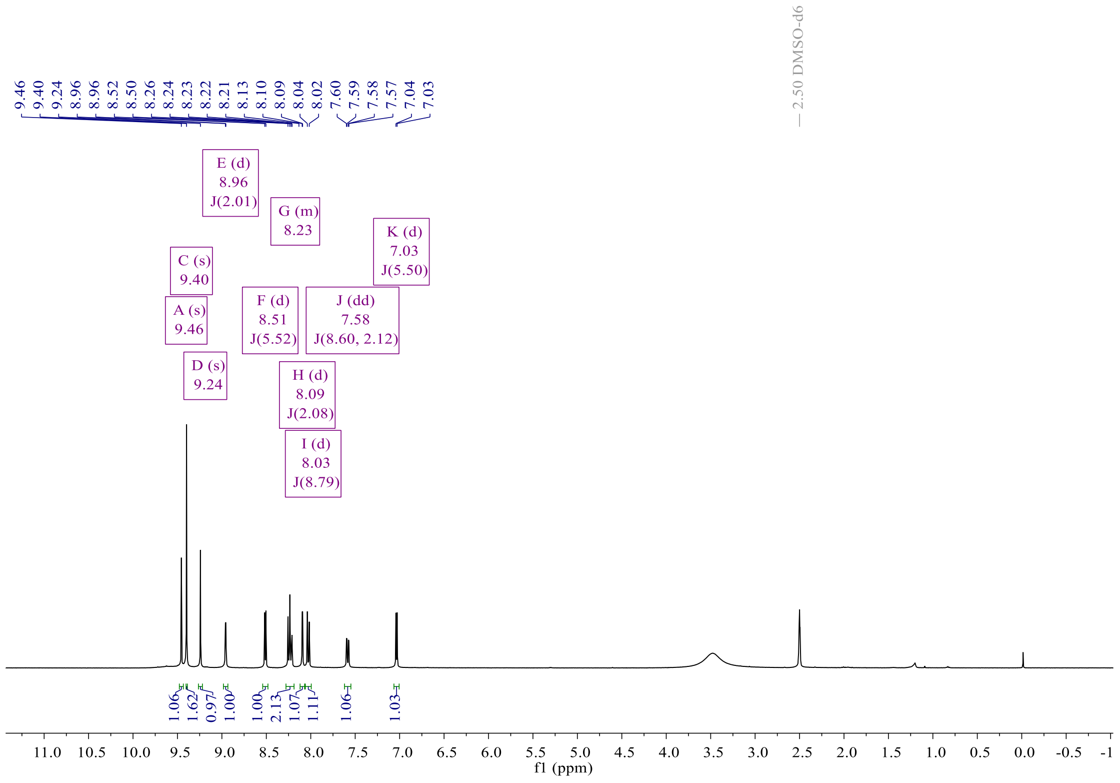
9.46
9.40
9.24
8.96
8.96
8.52
8.50
8.26
8.24
8.23
8.22
8.21
8.13
8.10
8.09
8.04
8.02
7.60
7.59
7.58
7.57
7.04
7.03
2.50 DMSO-d6
E (d) 8.96 J(2.01)
G (m) 8.23
K (d) 7.03 J(5.50)
C (s) 9.40
A (s) 9.46
F (d) 8.51 J(5.52)
J (dd) 7.58 J(8.60, 2.12)
D (s) 9.24
H (d) 8.09 J(2.08)
I (d) 8.03 J(8.79)
1.06
1.62
0.97
1.00
1.00
2.13
1.07
1.11
1.06
1.03
11.0 10.5 10.0 9.5 9.0 8.5 8.0 7.5 7.0 6.5 6.0 5.5 5.0 4.5 4.0 3.5 3.0 2.5 2.0 1.5 1.0 0.5 0.0 -0.5 -1
f1 (ppm)

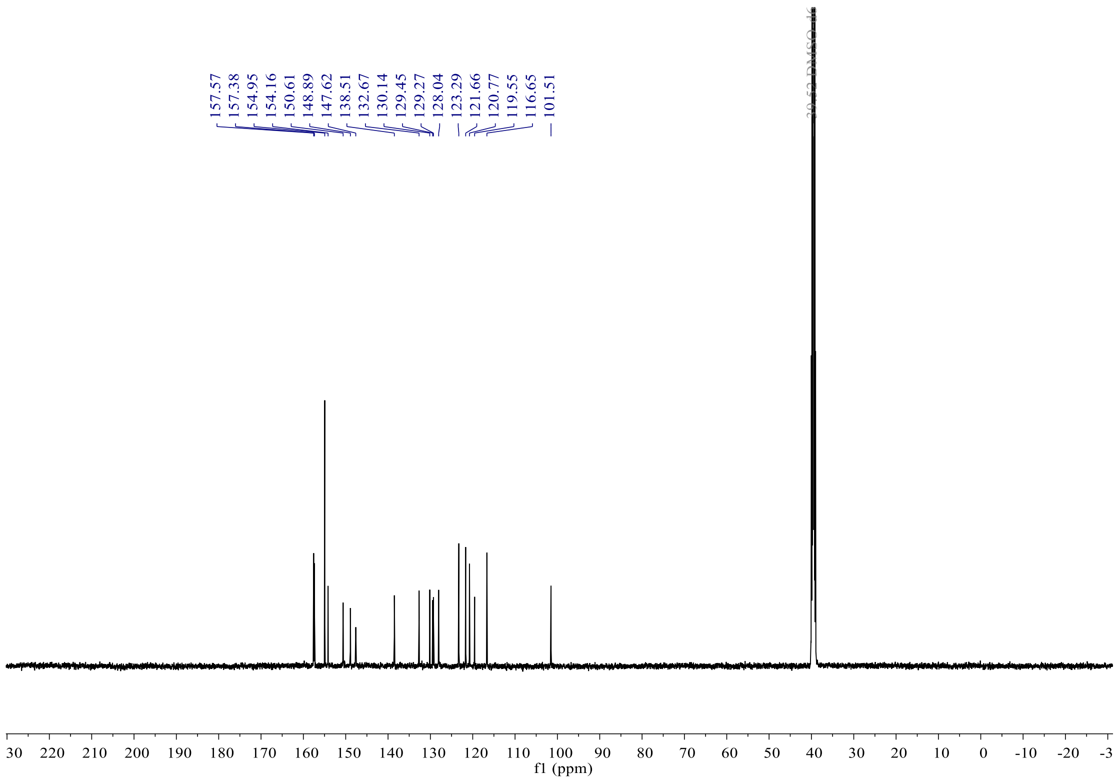
157.57
157.38
154.95
154.16
150.61
148.89
147.62
138.51
132.67
130.14
129.45
129.27
128.04
123.29
121.66
120.77
119.55
116.65
101.51
30 220 210 200 190 180 170 160 150 140 130 120 110 100 90 80 70 60 50 40 30 20 10 0 -10 -20 -3
f1 (ppm)

Analysis Report

Graph

**Sample Name**	ftt-10708-067
**Application Name**	Admin App (Administrator)
**Method Name**	User2
**Configuration Name**	Configuration 1
**Version**	42
**Data Instrument Name**	Detector
**Data Channel Name**	156 Channel 1
**Notes**	
**Injection Number**	2

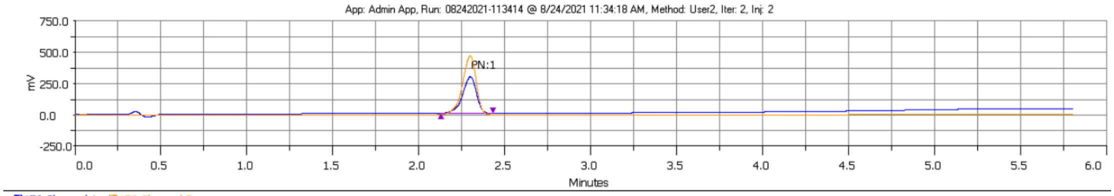

Sample Table

Injection Number	Sample Name	Sample Location	Peak Name	Retention Time (min)	Area (uVmin x100)	Area %	Height (mV)	Plate Number	
2	ftt-10708-067	Sample Zone->19	1	2.302	2688636.6667	100	291.21	4108.527	

D:\data\ftt-10708-067 8/24/2021 3:25:36 PM

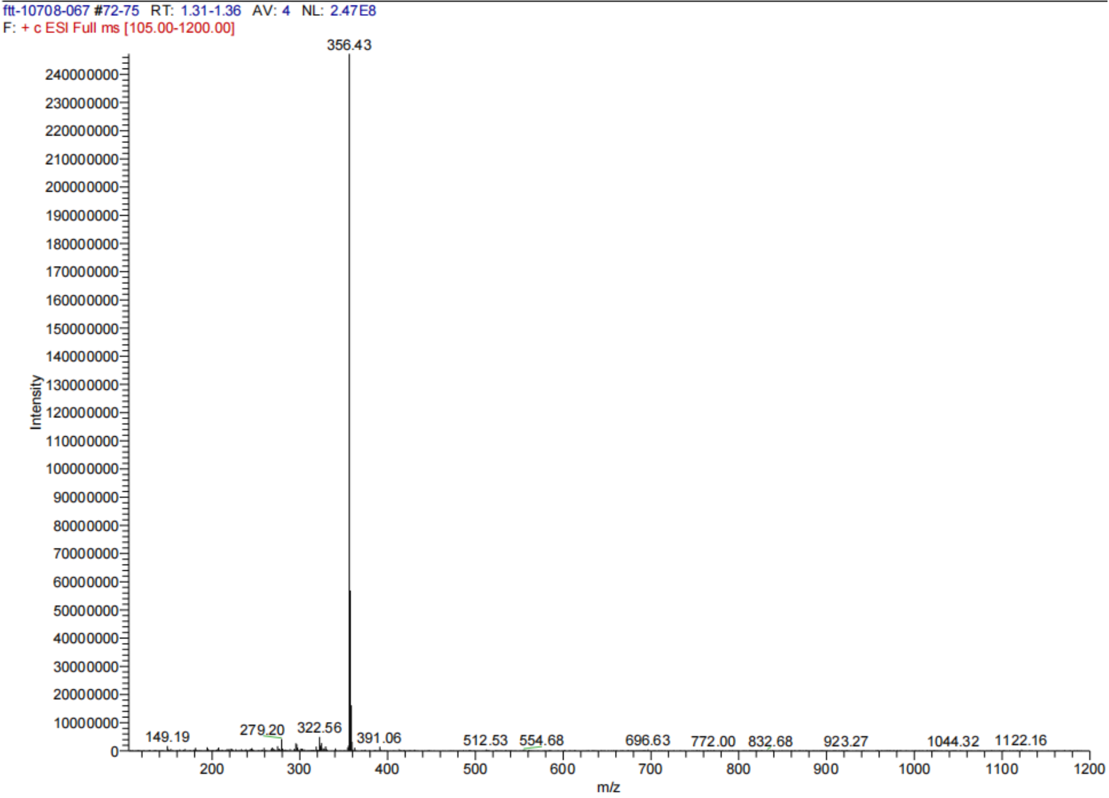

1.18 Compound 18

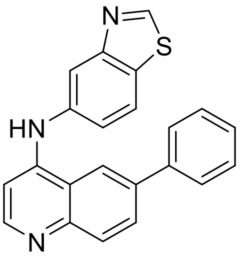

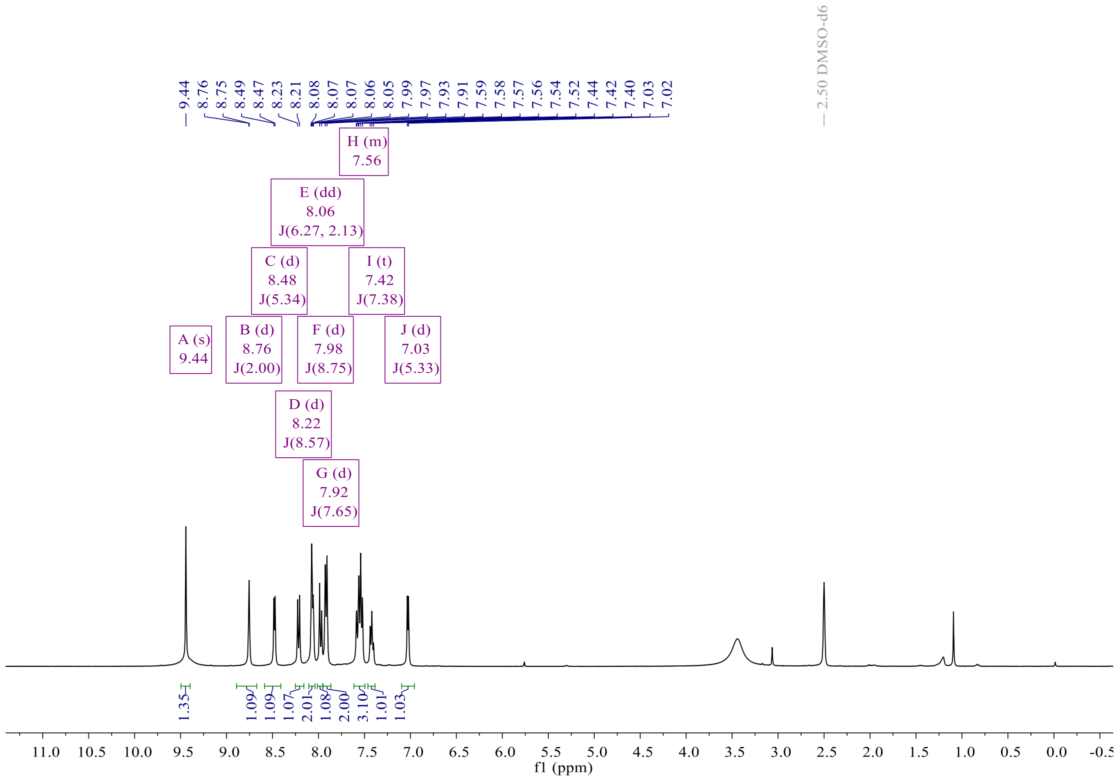

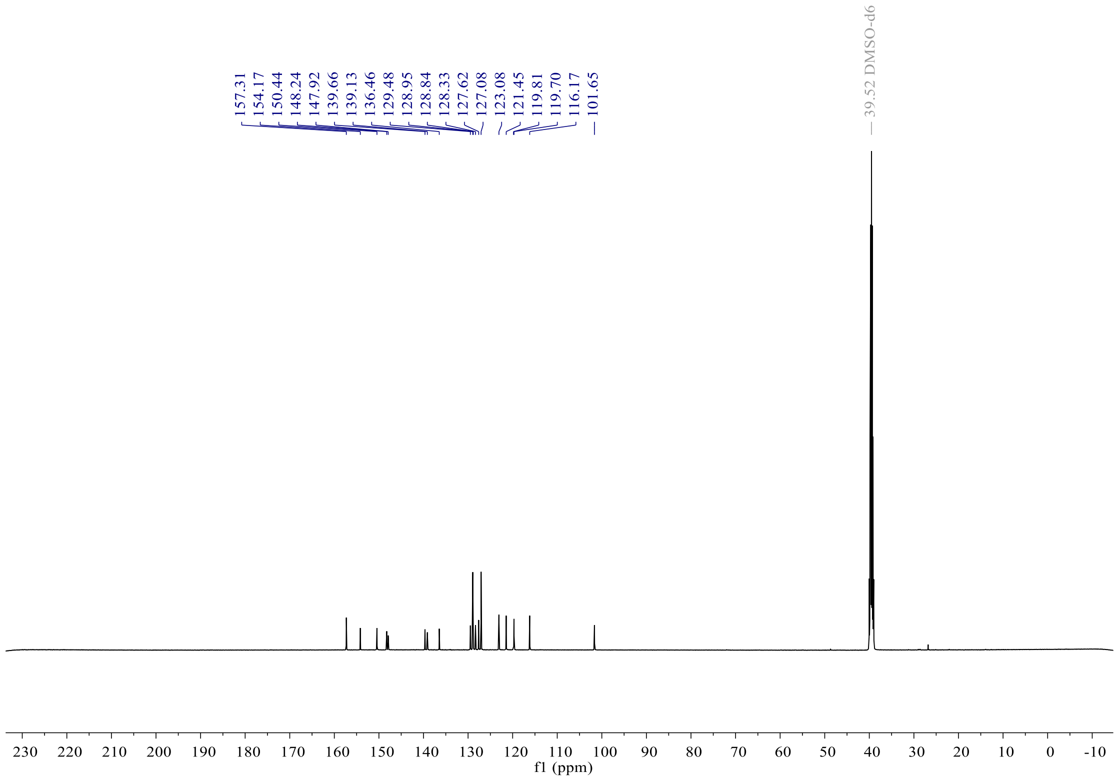

Analysis Report

Graph

**Sample Name**	ftt-10708-066r
**Application Name**	Admin App (Administrator)
**Method Name**	User1
**Configuration Name**	Configuration 1
**Version**	48
**Data Instrument Name**	Detector
**Data Channel Name**	156 Channel 1
**Notes**	
**Injection Number**	9

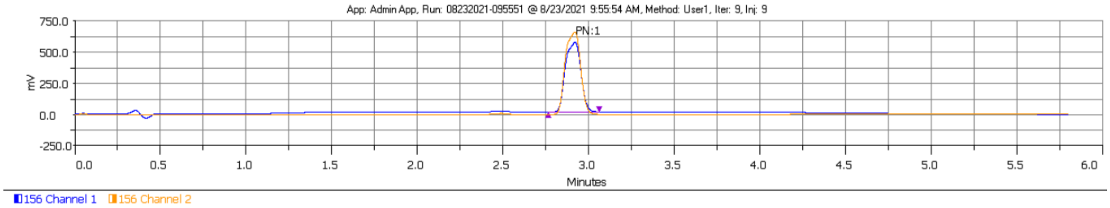

Sample Table

Injection Number	Sample Name	Sample Location	Peak Name	Retention Time (min)	Area (uVmin x100)	Area %	Height (mV)	Plate Number	
9	ftt-10708-066r	Sample Zone->6	1	2.921	5804610	100	557.827	4427.725	

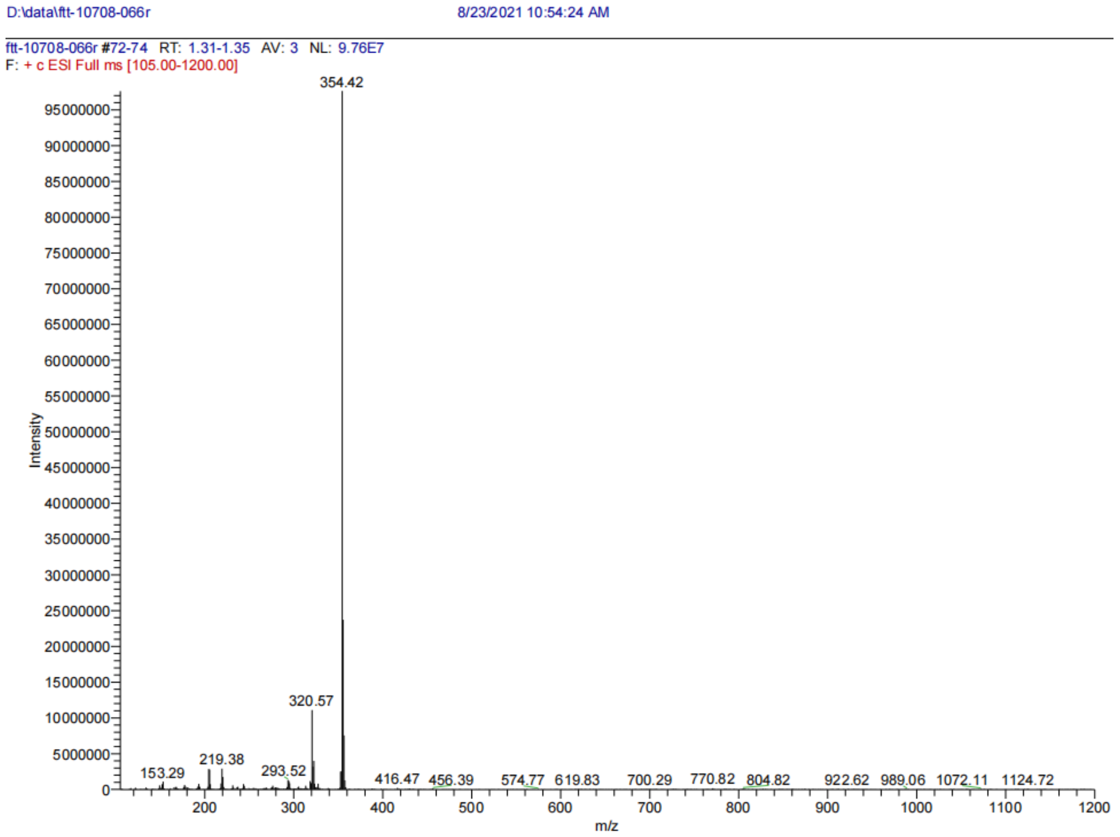

1.19 Compound 19

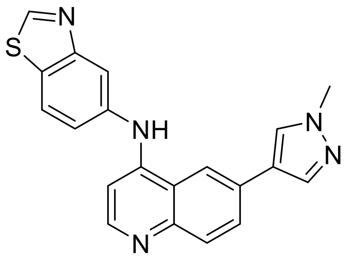

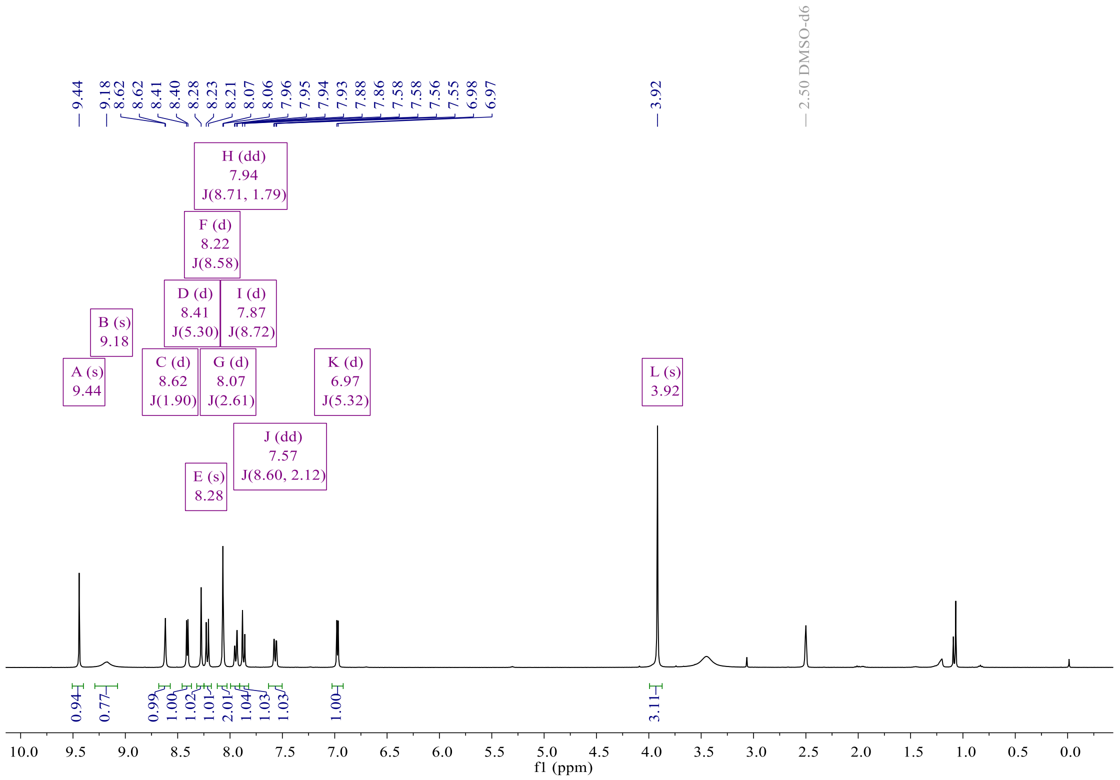

9.44
9.18
8.62
8.62
8.41
8.40
8.28
8.23
8.21
8.07
8.06
7.96
7.95
7.94
7.93
7.88
7.86
7.58
7.58
7.56
7.55
6.98
6.97
3.92
2.50 DMSO-d6
H (dd) 7.94 J(8.71, 1.79)
F (d) 8.22 J(8.58)
D (d) 8.41 J(5.30)
I (d) 7.87 J(8.72)
B (s) 9.18
A (s) 9.44
C (d) 8.62 J(1.90)
G (d) 8.07 J(2.61)
K (d) 6.97 J(5.32)
L (s) 3.92
J (dd) 7.57 J(8.60, 2.12)
E (s) 8.28
0.94
0.77
0.99
1.00
1.02
1.01
2.01
1.04
1.03
1.03
1.00
3.11
10.0 9.5 9.0 8.5 8.0 7.5 7.0 6.5 6.0 5.5 5.0 4.5 4.0 3.5 3.0 2.5 2.0 1.5 1.0 0.5 0.0
f1 (ppm)

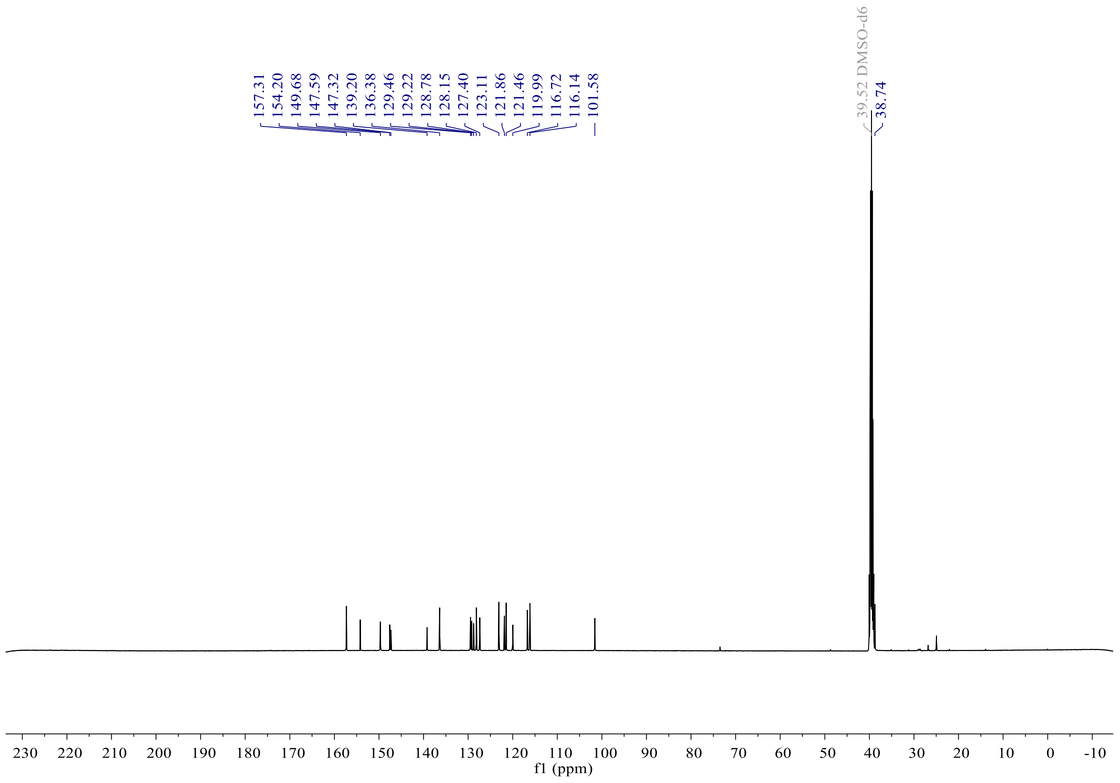

157.31
154.20
149.68
147.59
147.32
139.20
136.38
129.46
129.22
128.78
128.15
127.40
123.11
121.86
121.46
119.99
116.72
116.14
101.58
39.52 DMSO-d6
38.74
230 220 210 200 190 180 170 160 150 140 130 120 110 100 90 80 70 60 50 40 30 20 10 0 -10
f1 (ppm)

Analysis Report

Graph

**Sample Name**	ftt-10708-077
**Application Name**	Admin App (Administrator)
**Method Name**	User2
**Configuration Name**	Configuration 1
**Version**	42
**Data Instrument Name**	Detector
**Data Channel Name**	156 Channel 1
**Notes**	
**Injection Number**	6

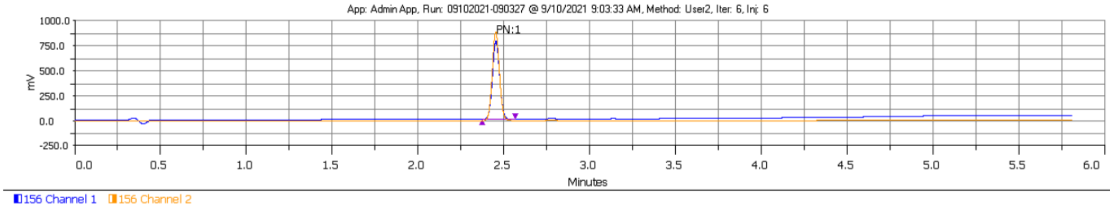

Sample Table

Injection Number	Sample Name	Sample Location	Peak Name	Retention Time (min)	Area (uVmin x100)	Area %	Height (mV)	Plate Number	
6	ftt-10708-077	Sample Zone->6	1	2.457	3820622.0833	100	780.366	16019.396	

D:\data\ftt-10708-077 9/10/2021 1:12:32 PM

ftt-10708-077 #79-81 RT: 1.33-1.36 AV: 3 NL: 1.33E9
F: + c ESI Full ms [105.00-1200.00]

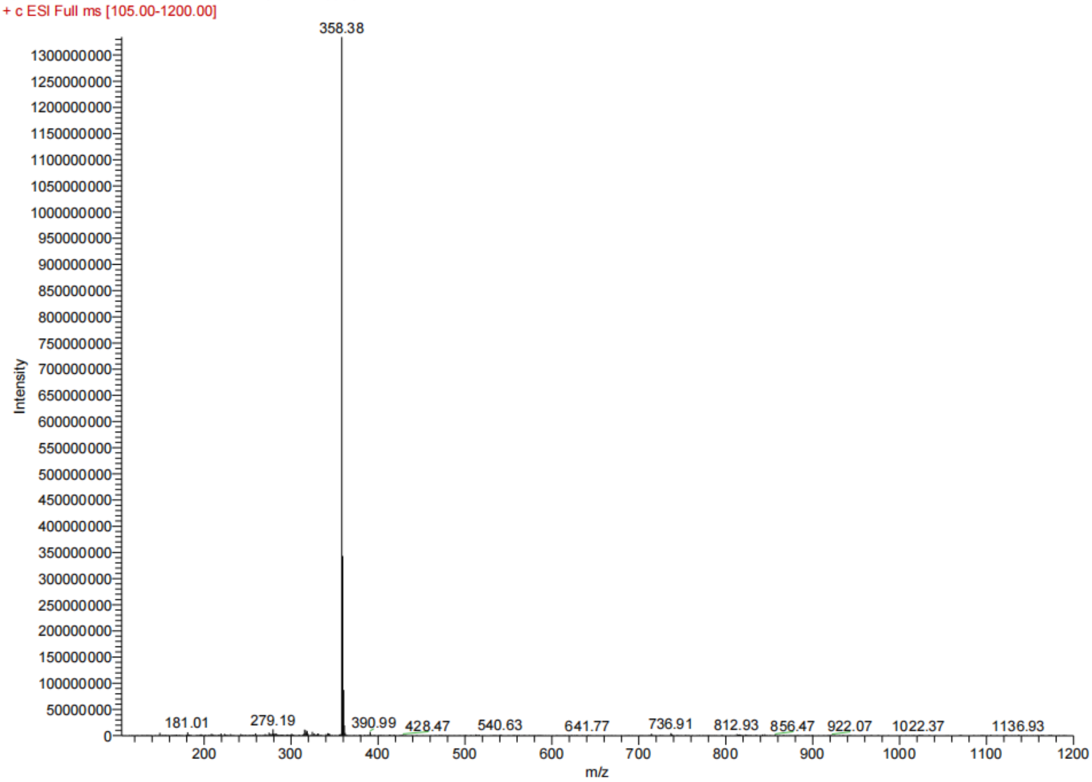

1.20 Compound 20

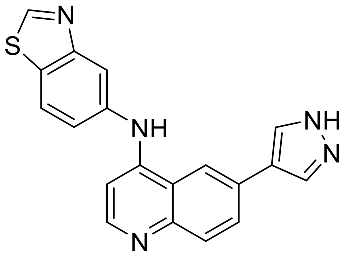

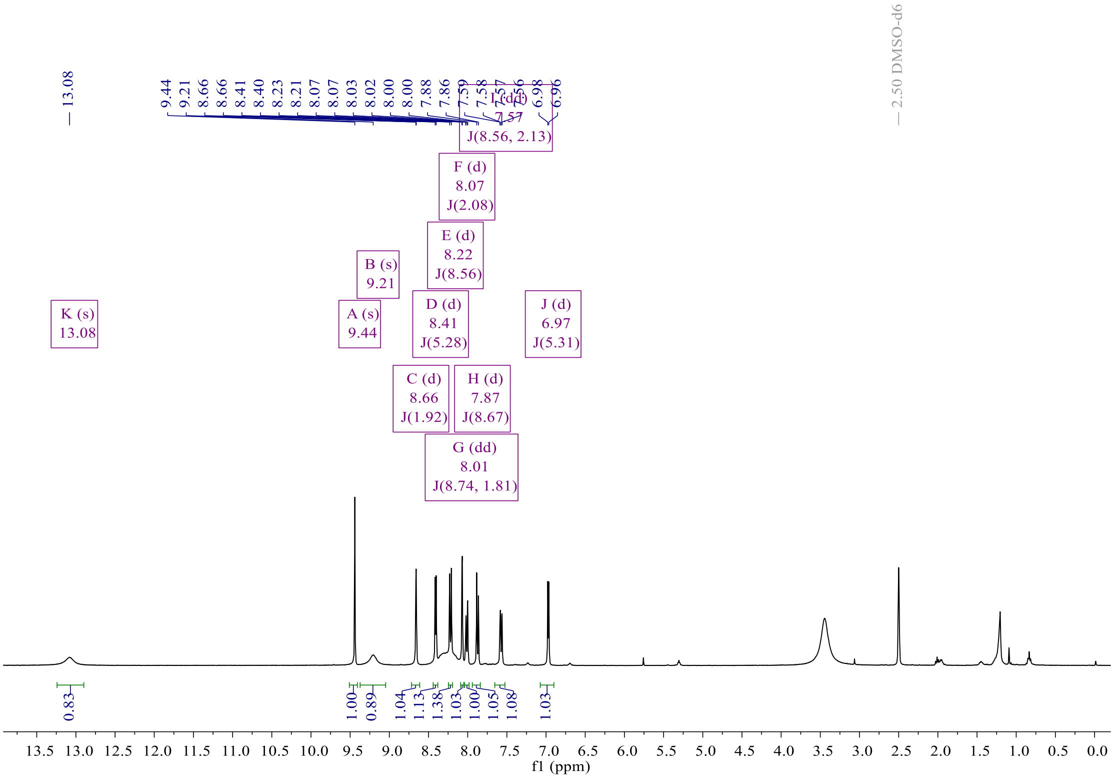

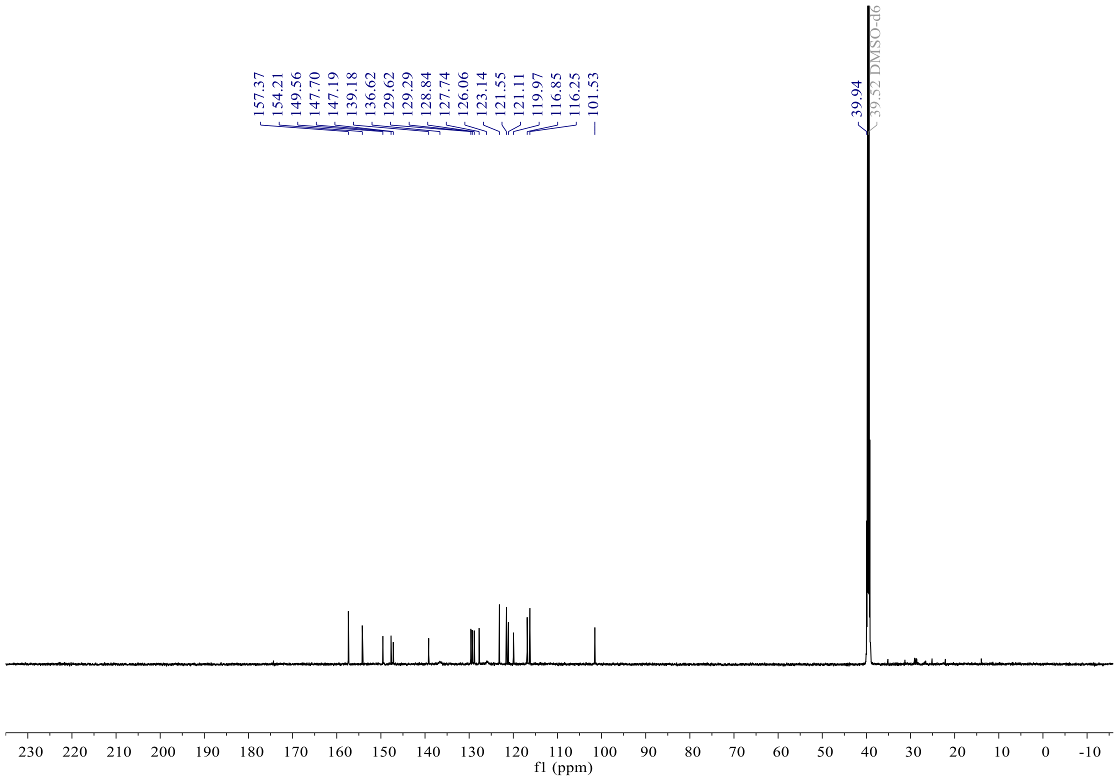

Analysis Report

Graph

**Sample Name**	ftt-10708-078
**Application Name**	Admin App (Administrator)
**Method Name**	User1
**Configuration Name**	Configuration 1
**Version**	48
**Data Instrument Name**	Detector
**Data Channel Name**	156 Channel 1
**Notes**	
**Injection Number**	7

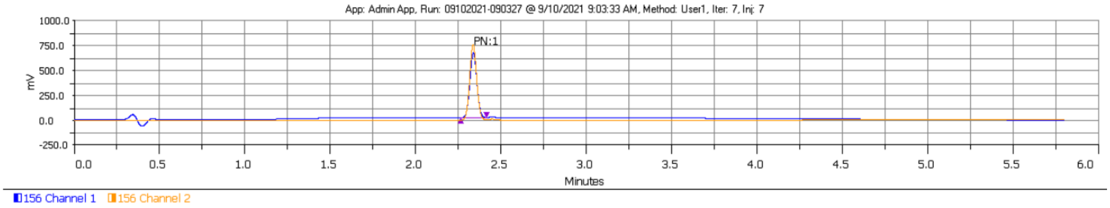

Sample Table

Injection Number	Sample Name	Sample Location	Peak Name	Retention Time (min)	Area (uVmin x100)	Area %	Height (mV)	Plate Number	
7	ftt-10708-078	Sample Zone->7	1	2.341	3147752.9167	100	653.478	14910.714	

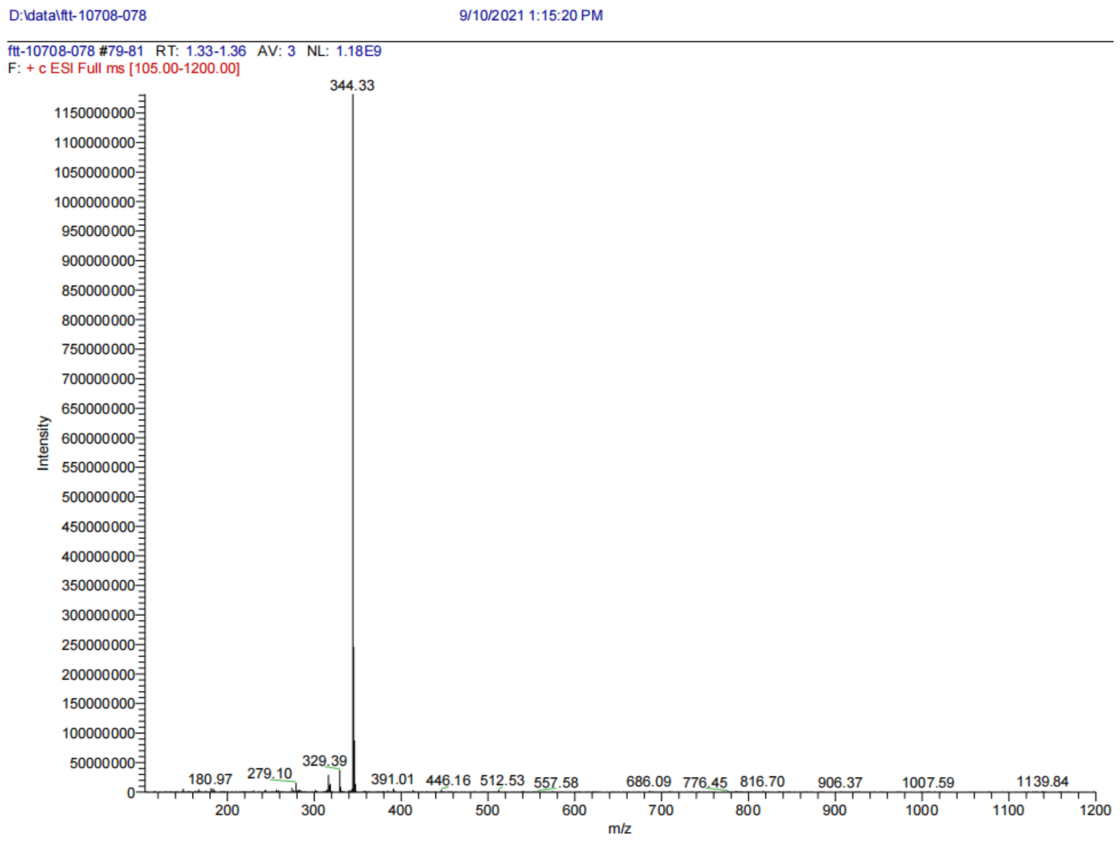

1.21 Compound 21

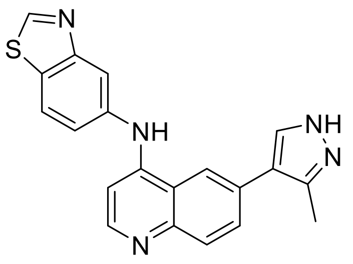

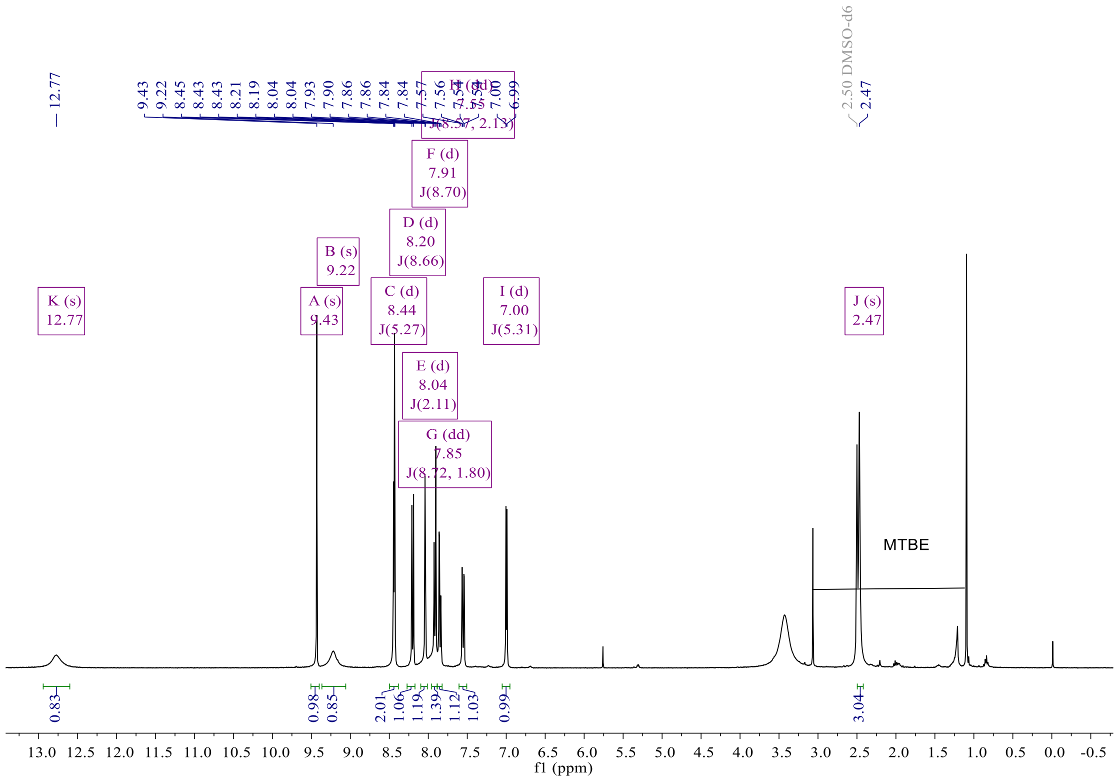

12.77
9.43
9.22
8.45
8.43
8.43
8.21
8.19
8.04
8.04
7.93
7.90
7.86
7.86
7.84
7.84
7.57
7.56
7.54
7.55
7.00
6.99
2.50 DMSO-d6
2.47
K (s) 12.77
A (s) 9.43
B (s) 9.22
C (d) 8.44 J(5.27)
D (d) 8.20 J(8.66)
E (d) 8.04 J(2.11)
F (d) 7.91 J(8.70)
G (dd) 7.85 J(8.72, 1.80)
I (d) 7.00 J(5.31)
J (s) 2.47
MTBE
0.83
0.98
0.85
2.01
1.06
1.19
1.39
1.12
1.03
0.99
3.04
f1 (ppm)

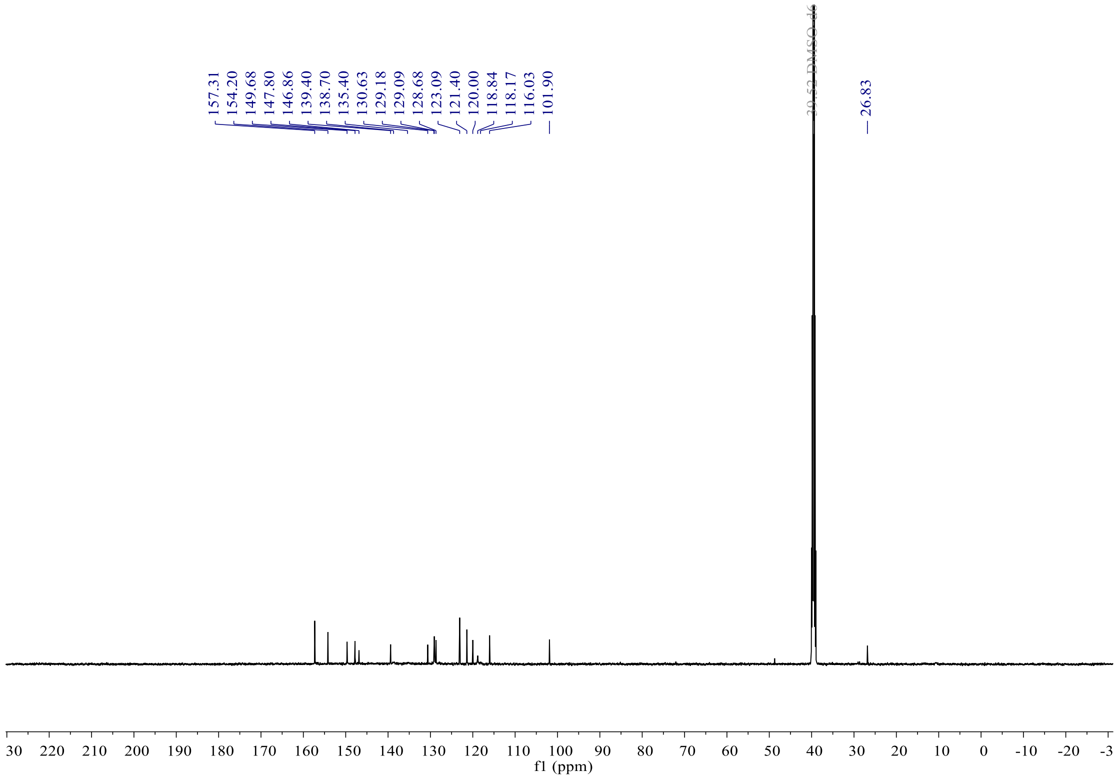

157.31
154.20
149.68
147.80
146.86
139.40
138.70
135.40
130.63
129.18
129.09
128.68
123.09
121.40
120.00
118.84
118.17
116.03
101.90
26.83
f1 (ppm)

Analysis Report

Graph

**Sample Name**	ftt-10708-080
**Application Name**	Admin App (Administrator)
**Method Name**	User2
**Configuration Name**	Configuration 1
**Version**	42
**Data Instrument Name**	Detector
**Data Channel Name**	156 Channel 1
**Notes**	
**Injection Number**	8

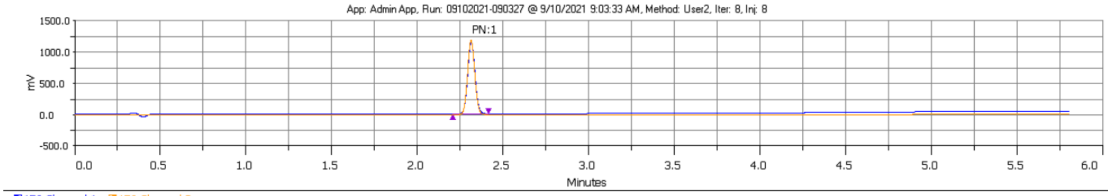

Sample Table

Injection Number	Sample Name	Sample Location	Peak Name	Retention Time (min)	Area (uVmin x100)	Area %	Height (mV)	Plate Number	
8	ftt-10708-080	Sample Zone->8	1	2.318	5730272.9167	100	1159.946	13963.261	

D:\data\ftt-10708-080 9/10/2021 1:18:08 PM

ftt-10708-080 #79-81 RT: 1.33-1.36 AV: 3 NL: 2.01E9
F: + c ESI Full ms [105.00-1200.00]

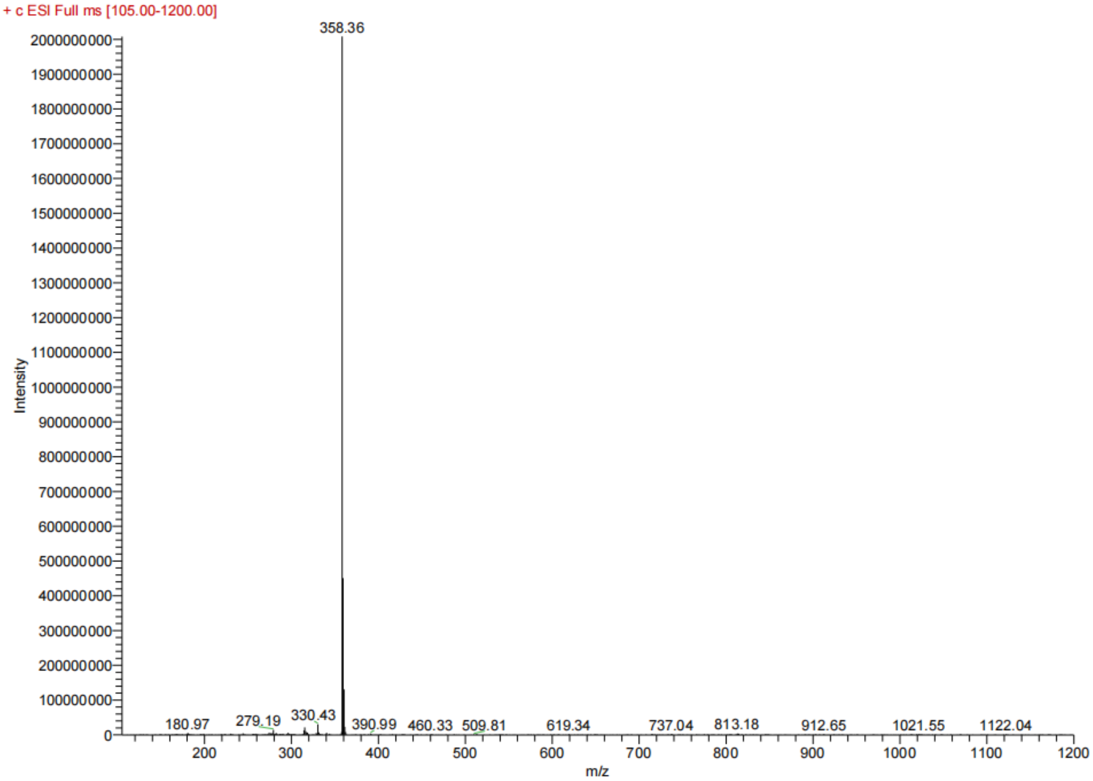

1.22 Compound 22

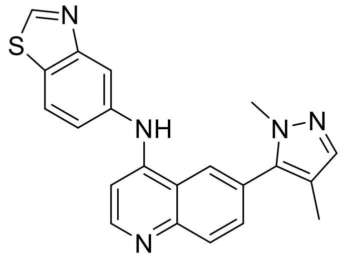

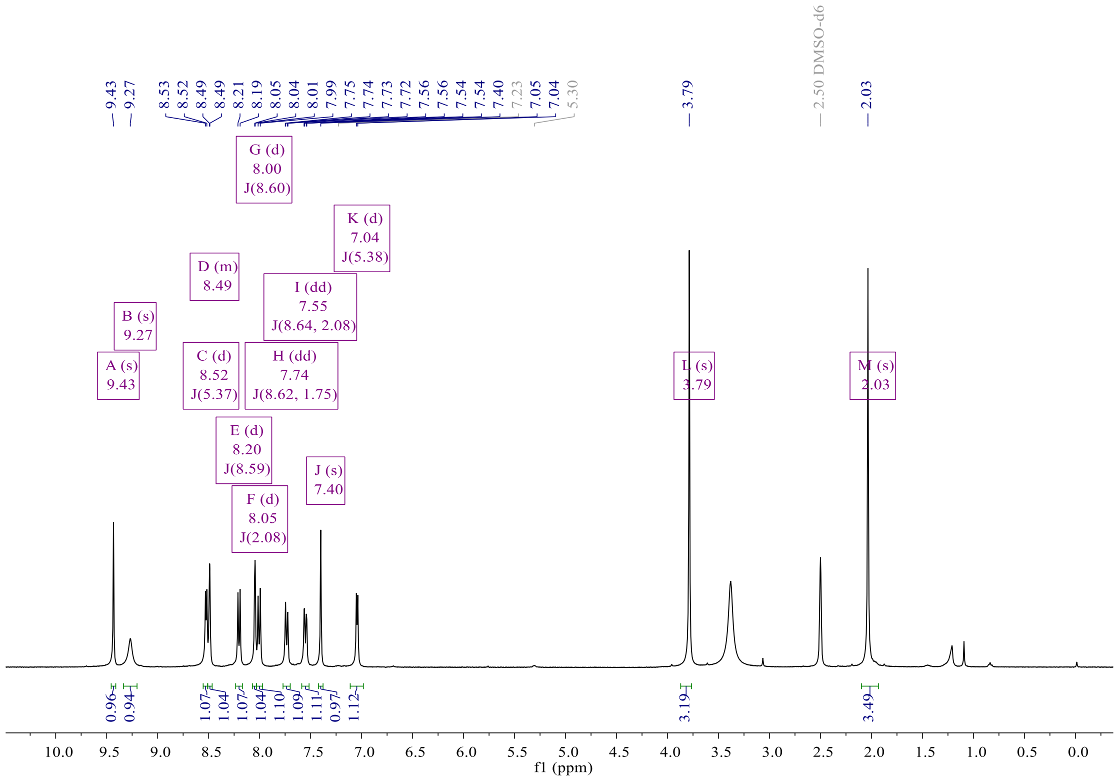

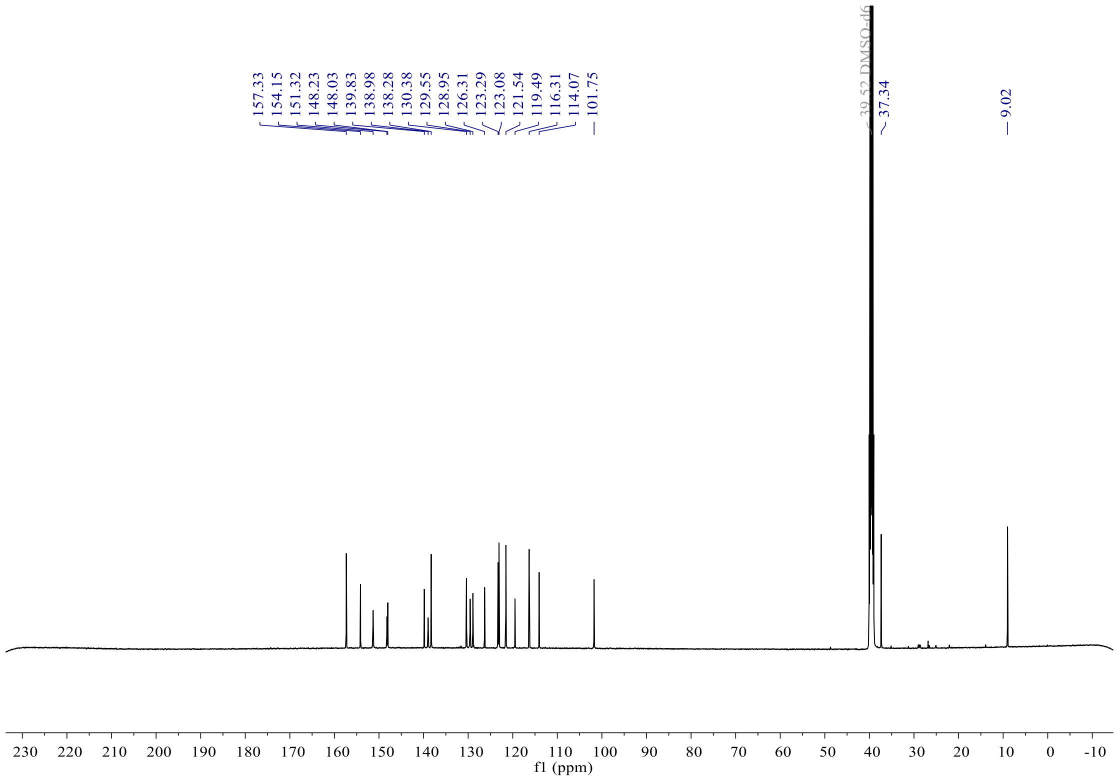

Analysis Report

Graph

**Sample Name**	ftt-10708-081
**Application Name**	Admin App (Administrator)
**Method Name**	User1
**Configuration Name**	Configuration 1
**Version**	48
**Data Instrument Name**	Detector
**Data Channel Name**	156 Channel 1
**Notes**	
**Injection Number**	9

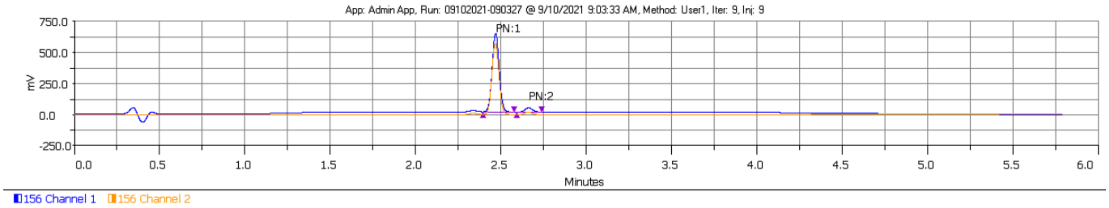

Sample Table

Injection Number	Sample Name	Sample Location	Peak Name	Retention Time (min)	Area (uVmin x100)	Area %	Height (mV)	Plate Number	
9	ftt-10708-081	Sample Zone->9	1	2.472	3053789.1667	95.112	630.796	16524.099	
9	ftt-10708-081	Sample Zone->9	2	2.666	156950	4.888	32.495	19153.392	

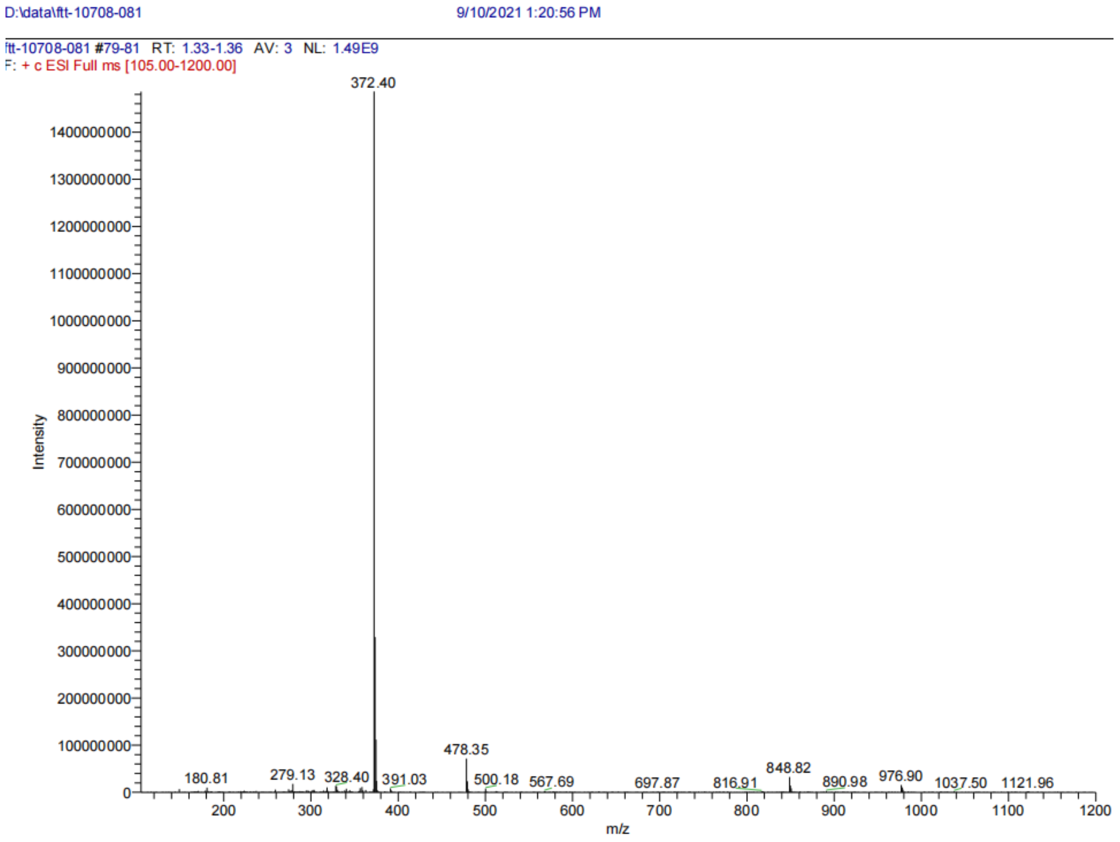

1.23 Compound 23

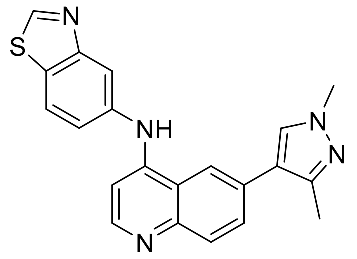

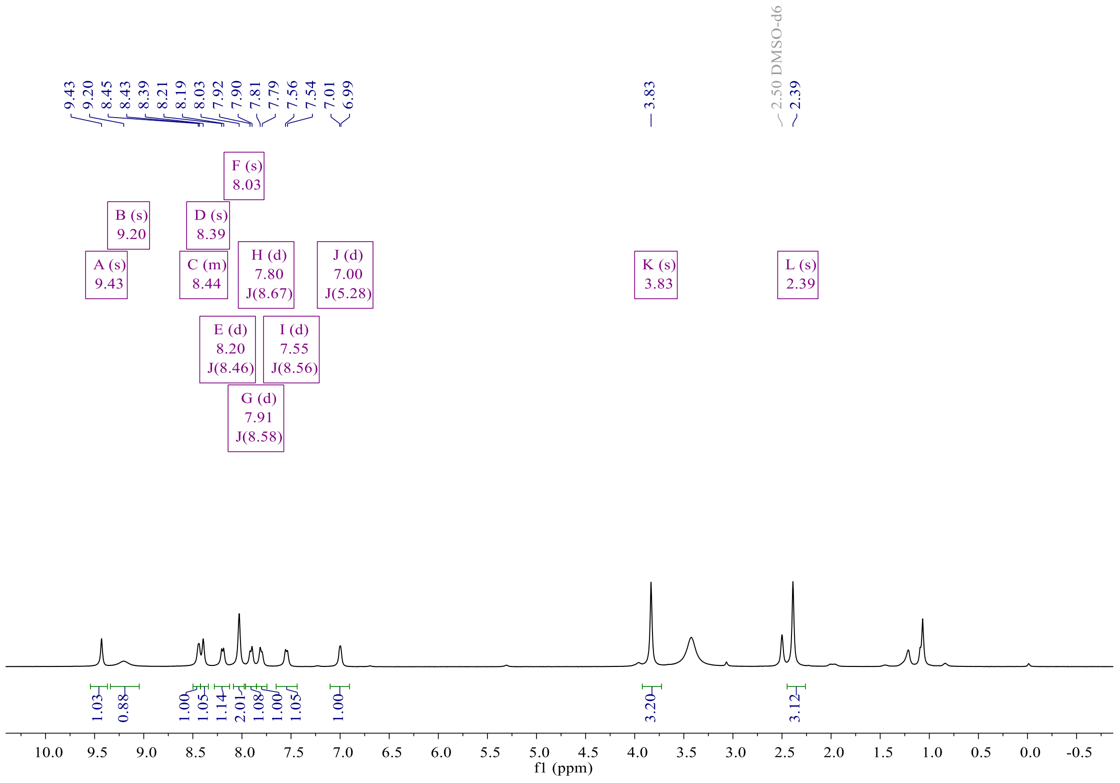
9.43
9.20
8.45
8.43
8.39
8.21
8.19
8.03
7.92
7.90
7.81
7.79
7.56
7.54
7.01
6.99
3.83
2.50 DMSO-d6
2.39
A (s) 9.43
B (s) 9.20
C (m) 8.44
D (s) 8.39
E (d) 8.20 J(8.46)
F (s) 8.03
G (d) 7.91 J(8.58)
H (d) 7.80 J(8.67)
I (d) 7.55 J(8.56)
J (d) 7.00 J(5.28)
K (s) 3.83
L (s) 2.39
1.03
0.88
1.00
1.05
1.14
2.01
1.08
1.00
1.05
1.00
3.20
3.12
10.0 9.5 9.0 8.5 8.0 7.5 7.0 6.5 6.0 5.5 5.0 4.5 4.0 3.5 3.0 2.5 2.0 1.5 1.0 0.5 0.0 -0.5
f1 (ppm)

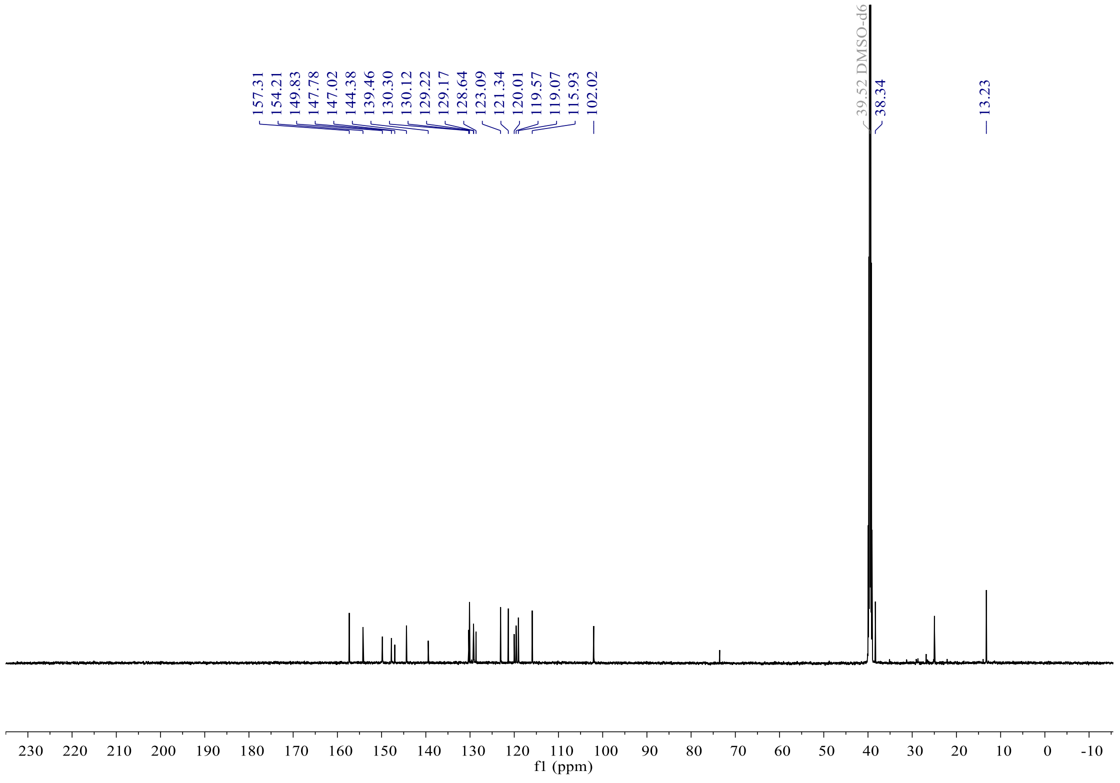
157.31
154.21
149.83
147.78
147.02
144.38
139.46
130.30
130.12
129.22
129.17
128.64
123.09
121.34
120.01
119.57
119.07
115.93
102.02
39.52 DMSO-d6
38.34
13.23
230 220 210 200 190 180 170 160 150 140 130 120 110 100 90 80 70 60 50 40 30 20 10 0 -10
f1 (ppm)

Analysis Report

Graph

**Sample Name**	ftt-10708-082
**Application Name**	Admin App (Administrator)
**Method Name**	User2
**Configuration Name**	Configuration 1
**Version**	42
**Data Instrument Name**	Detector
**Data Channel Name**	156 Channel 1
**Notes**	
**Injection Number**	10

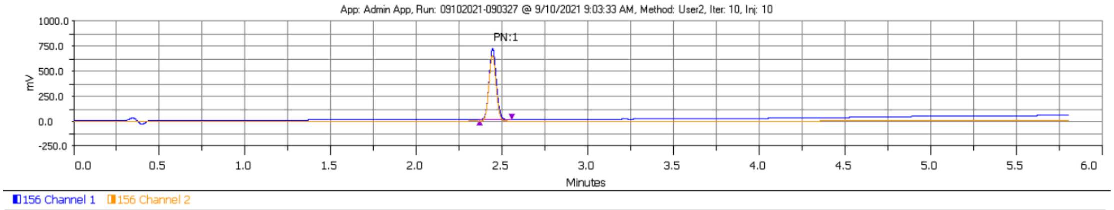

Sample Table

Injection Number	Sample Name	Sample Location	Peak Name	Retention Time (min)	Area (uVmin x100)	Area %	Height (mV)	Plate Number
10	ftt-10708-082	Sample Zone->10	1	2.447	3481133.3333	100	711.988	15931.891

D:\data\ftt-10708-082 9/10/2021 1:23:44 PM

ftt-10708-082 #79-81 RT: 1.33-1.36 AV: 3 NL: 1.18E9
F: + c ESI Full ms [105.00-1200.00]

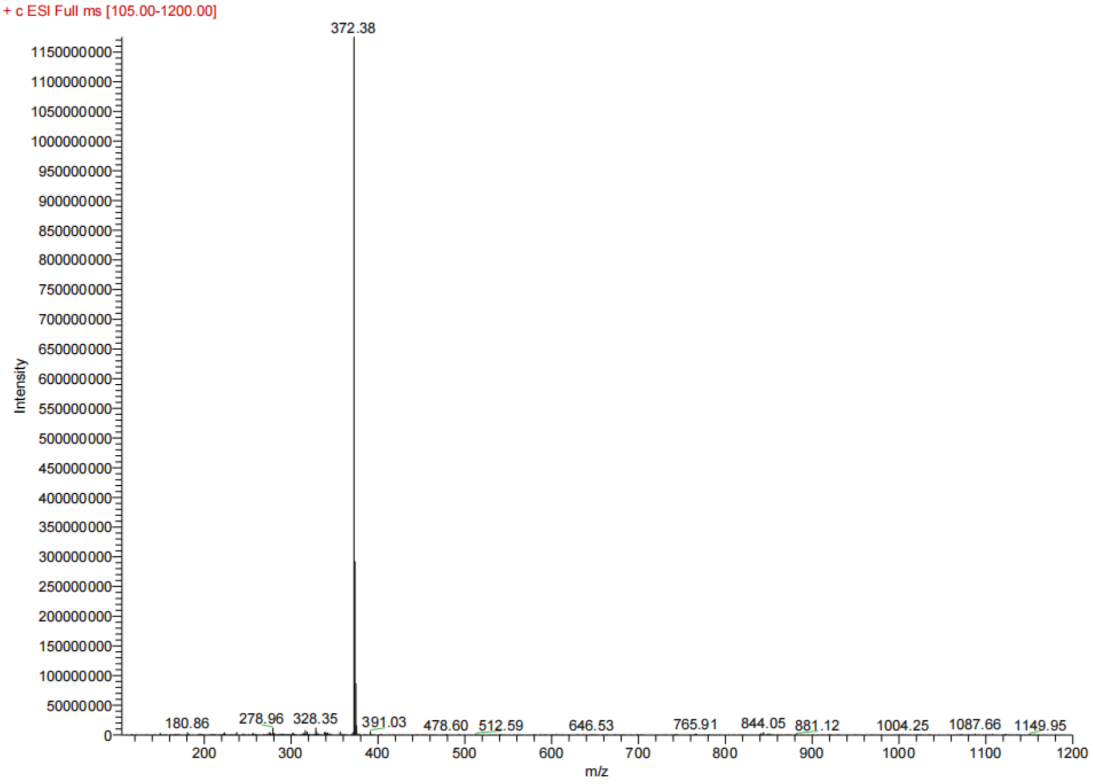

1.24 Compound 24

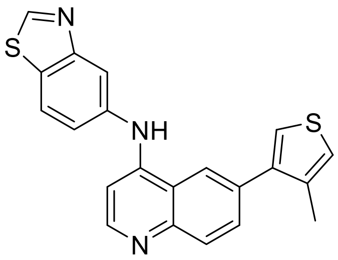

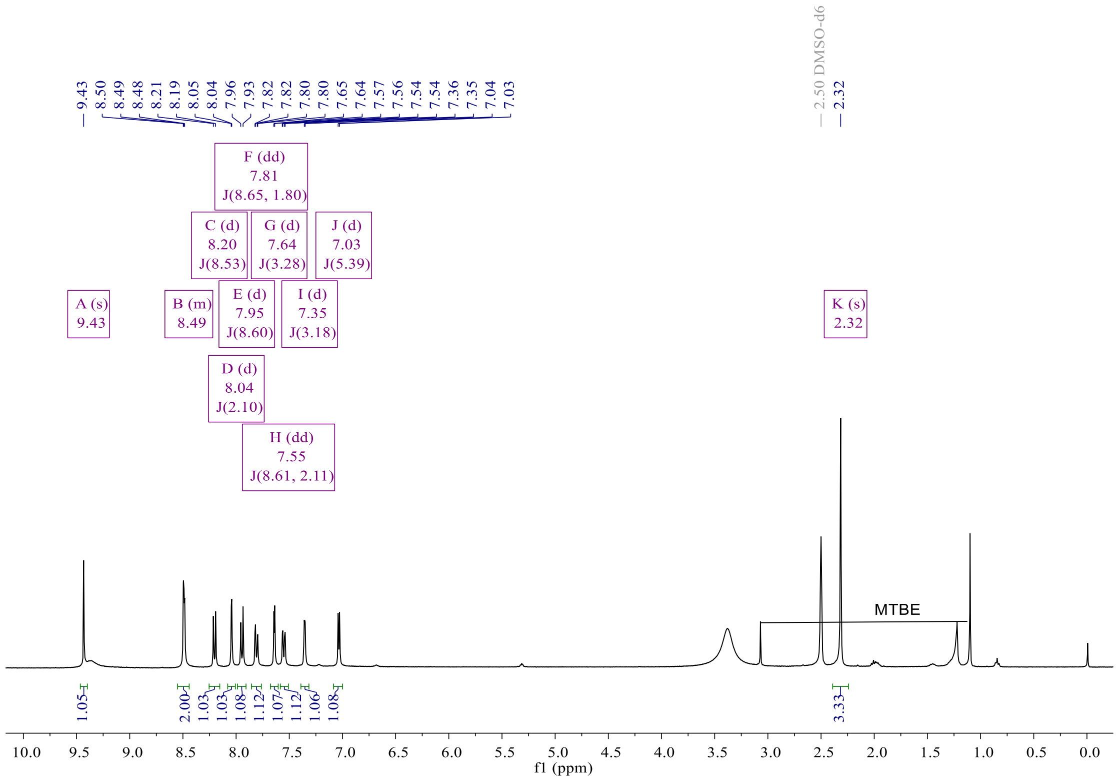
9.43
8.50
8.49
8.48
8.21
8.19
8.05
8.04
7.96
7.93
7.82
7.82
7.80
7.80
7.65
7.64
7.57
7.56
7.54
7.54
7.36
7.35
7.04
7.03
2.50 DMSO-d6
2.32
F (dd) 7.81 J(8.65, 1.80)
C (d) 8.20 J(8.53)
G (d) 7.64 J(3.28)
J (d) 7.03 J(5.39)
A (s) 9.43
B (m) 8.49
E (d) 7.95 J(8.60)
I (d) 7.35 J(3.18)
K (s) 2.32
D (d) 8.04 J(2.10)
H (dd) 7.55 J(8.61, 2.11)
MTBE
1.05
2.00
1.03
1.03
1.08
1.12
1.07
1.12
1.06
1.08
3.33
10.0 9.5 9.0 8.5 8.0 7.5 7.0 6.5 6.0 5.5 5.0 4.5 4.0 3.5 3.0 2.5 2.0 1.5 1.0 0.5 0.0
f1 (ppm)

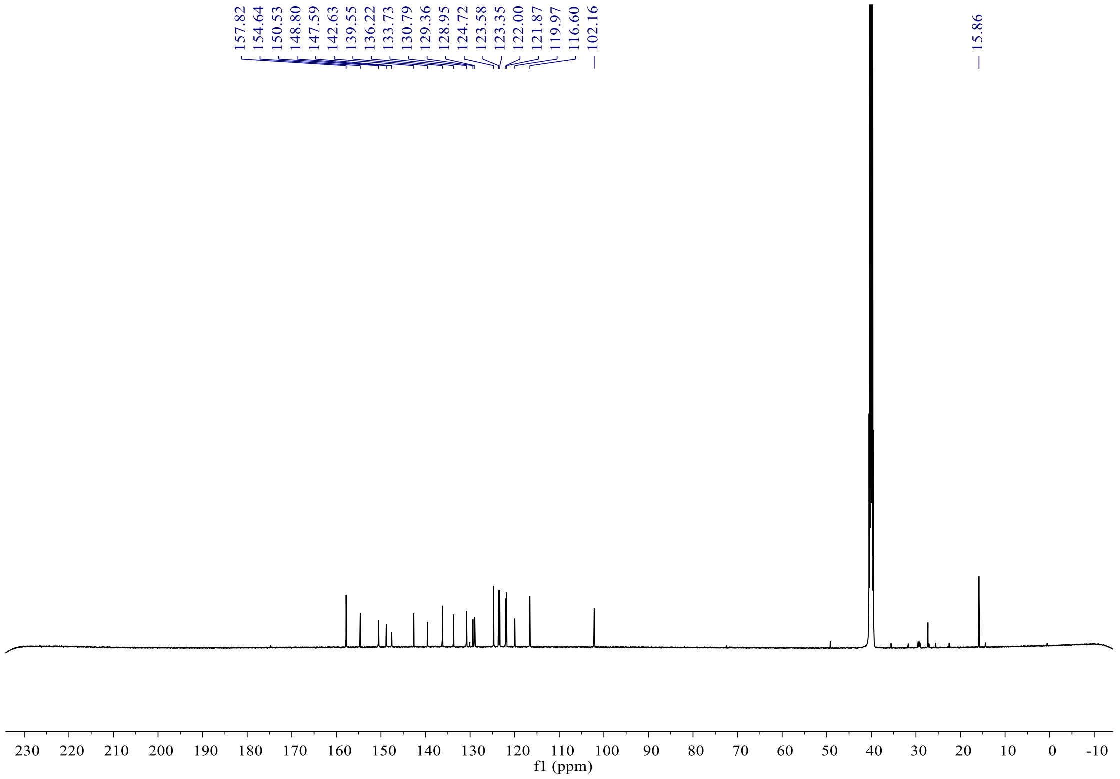
157.82
154.64
150.53
148.80
147.59
142.63
139.55
136.22
133.73
130.79
129.36
128.95
124.72
123.58
123.35
122.00
121.87
119.97
116.60
102.16
15.86
230 220 210 200 190 180 170 160 150 140 130 120 110 100 90 80 70 60 50 40 30 20 10 0 -10
f1 (ppm)

Analysis Report

Graph

**Sample Name**	ftt-10708-083
**Application Name**	Admin App (Administrator)
**Method Name**	User1
**Configuration Name**	Configuration 1
**Version**	48
**Data Instrument Name**	Detector
**Data Channel Name**	156 Channel 1
**Notes**	
**Injection Number**	5

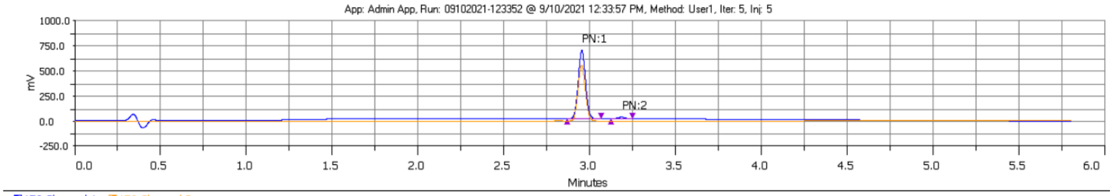

Sample Table

Injection Number	Sample Name	Sample Location	Peak Name	Retention Time (min)	Area (uVmin x100)	Area %	Height (mV)	Plate Number	
5	ftt-10708-083	Sample Zone->11	1	2.958	3412229.1667	97.334	683.587	22323.319	
5	ftt-10708-083	Sample Zone->11	2	3.188	93447.9167	2.666	18.739	25446.567	

D:\data\ftt-10708-083 9/10/2021 1:27:13 PM

ftt-10708-083 #77-79 RT: 1.30-1.33 AV: 3 NL: 1.93E9
F: + c ESI Full ms [105.00-1200.00]

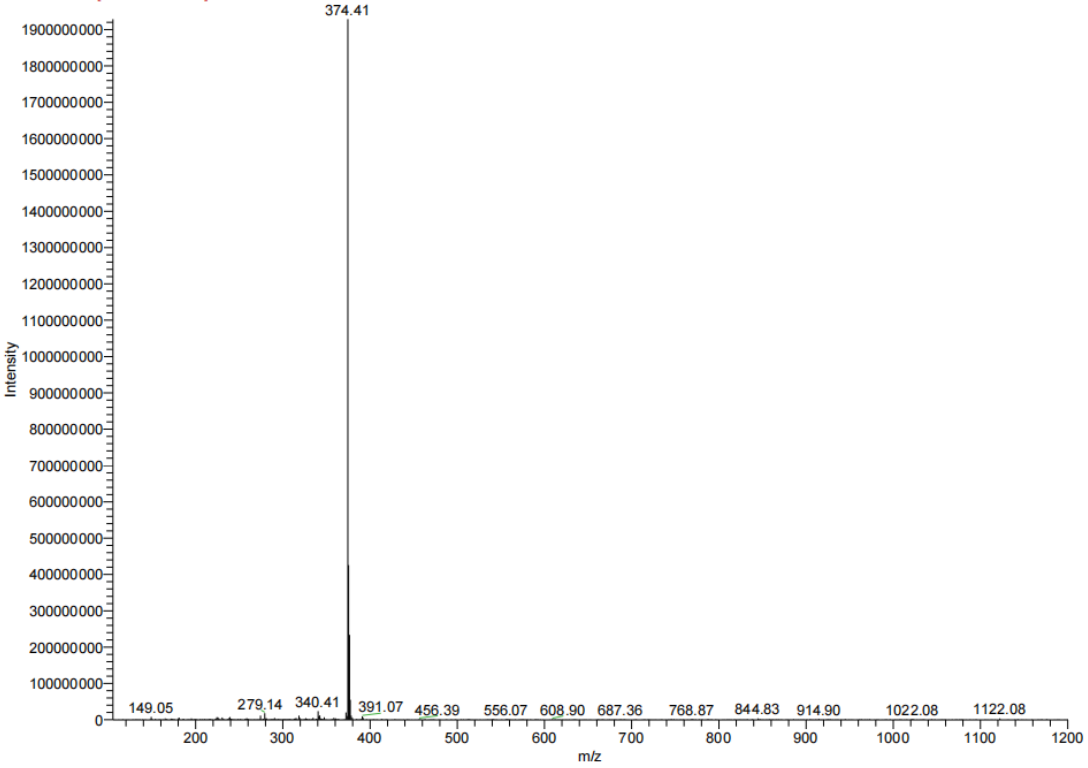

1.25 Compound 25

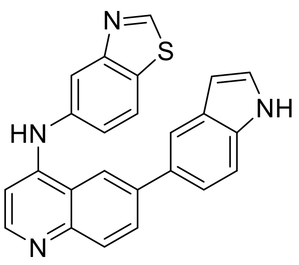

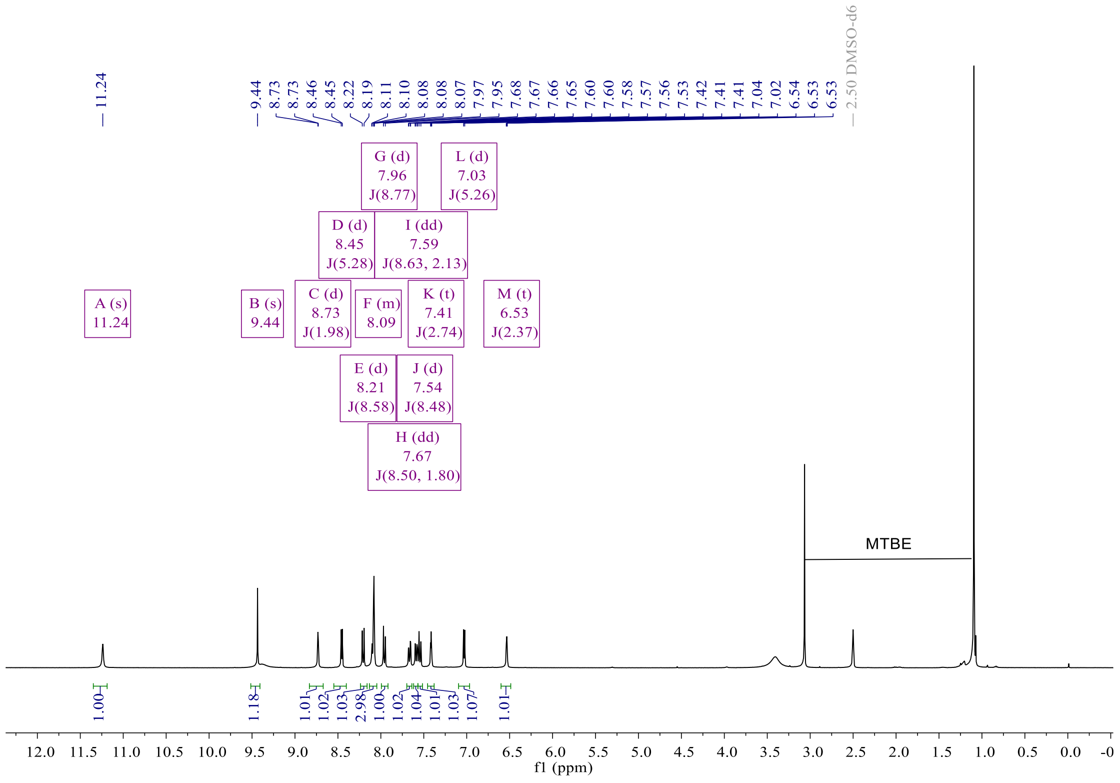

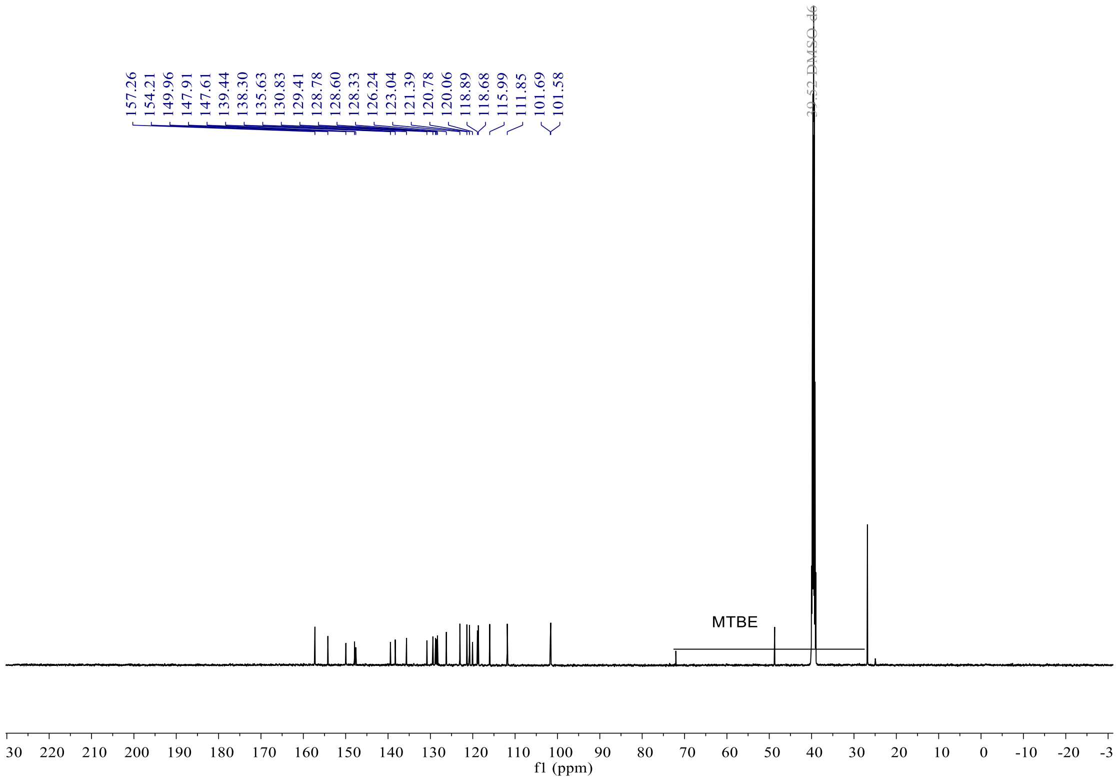

Analysis Report

Graph

**Sample Name**	ftt-10708-068
**Application Name**	Admin App (Administrator)
**Method Name**	User1
**Configuration Name**	Configuration 1
**Version**	48
**Data Instrument Name**	Detector
**Data Channel Name**	156 Channel 1
**Notes**	
**Injection Number**	9

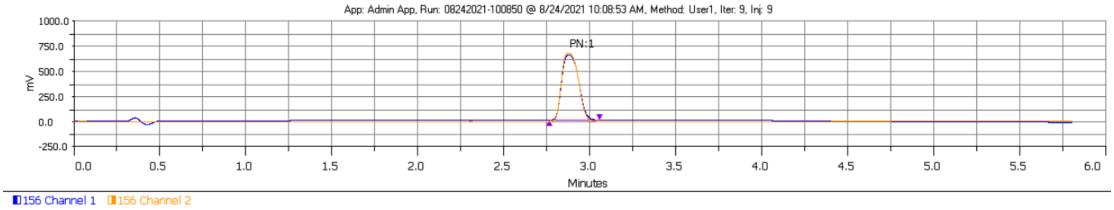

Sample Table

Injection Number	Sample Name	Sample Location	Peak Name	Retention Time (min)	Area (uVmin x100)	Area %	Height (mV)	Plate Number	
9	ftt-10708-068	Sample Zone->20	1	2.884	7265128.3333	100	645.629	3750.531	

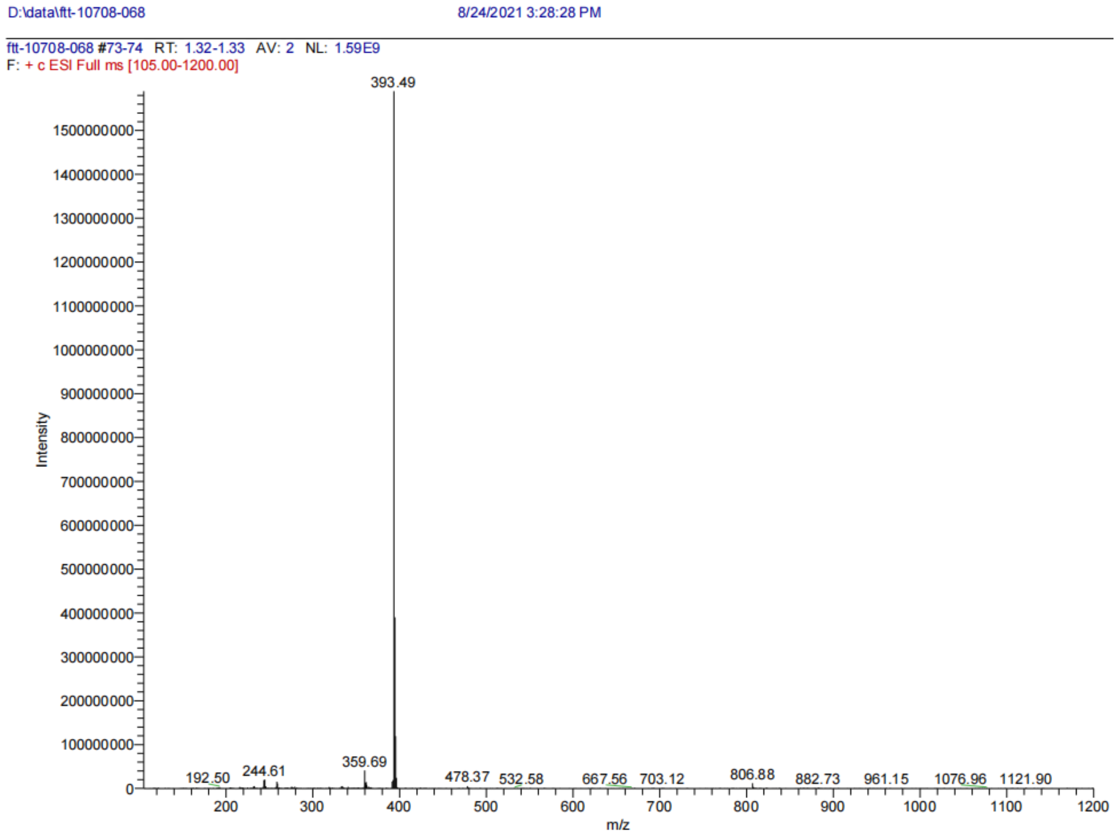

1.26 Compound 26

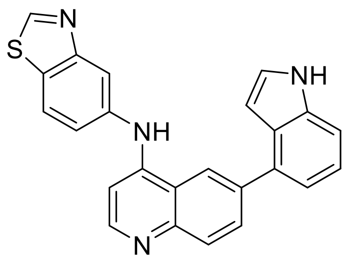

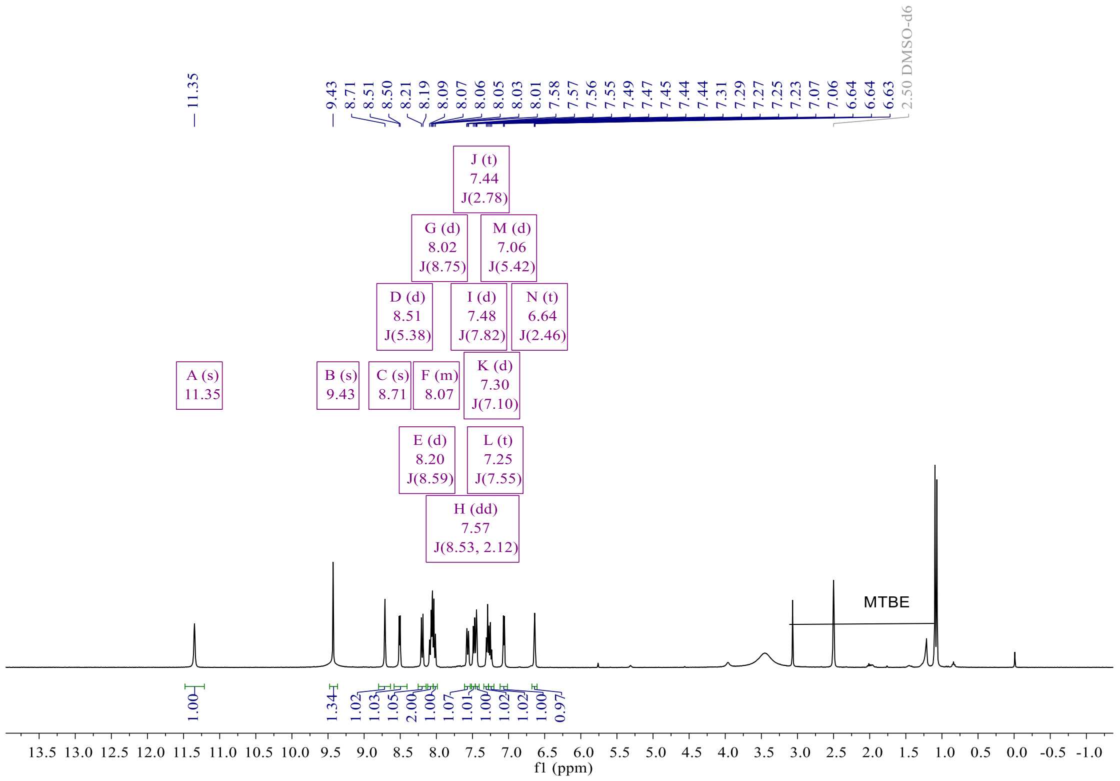

11.35
9.43
8.71
8.51
8.50
8.21
8.19
8.09
8.07
8.06
8.05
8.03
8.01
7.58
7.57
7.56
7.55
7.49
7.47
7.45
7.44
7.44
7.31
7.29
7.27
7.25
7.23
7.07
7.06
6.64
6.64
6.63
2.50 DMSO-d6
J (t) 7.44 J(2.78)
G (d) 8.02 J(8.75)
M (d) 7.06 J(5.42)
D (d) 8.51 J(5.38)
I (d) 7.48 J(7.82)
N (t) 6.64 J(2.46)
A (s) 11.35
B (s) 9.43
C (s) 8.71
F (m) 8.07
K (d) 7.30 J(7.10)
E (d) 8.20 J(8.59)
L (t) 7.25 J(7.55)
H (dd) 7.57 J(8.53, 2.12)
MTBE
1.00
1.34
1.02
1.03
1.05
2.00
1.00
1.07
1.01
1.00
1.02
1.02
1.00
0.97
13.5 13.0 12.5 12.0 11.5 11.0 10.5 10.0 9.5 9.0 8.5 8.0 7.5 7.0 6.5 6.0 5.5 5.0 4.5 4.0 3.5 3.0 2.5 2.0 1.5 1.0 0.5 0.0 -0.5 -1.0
f1 (ppm)

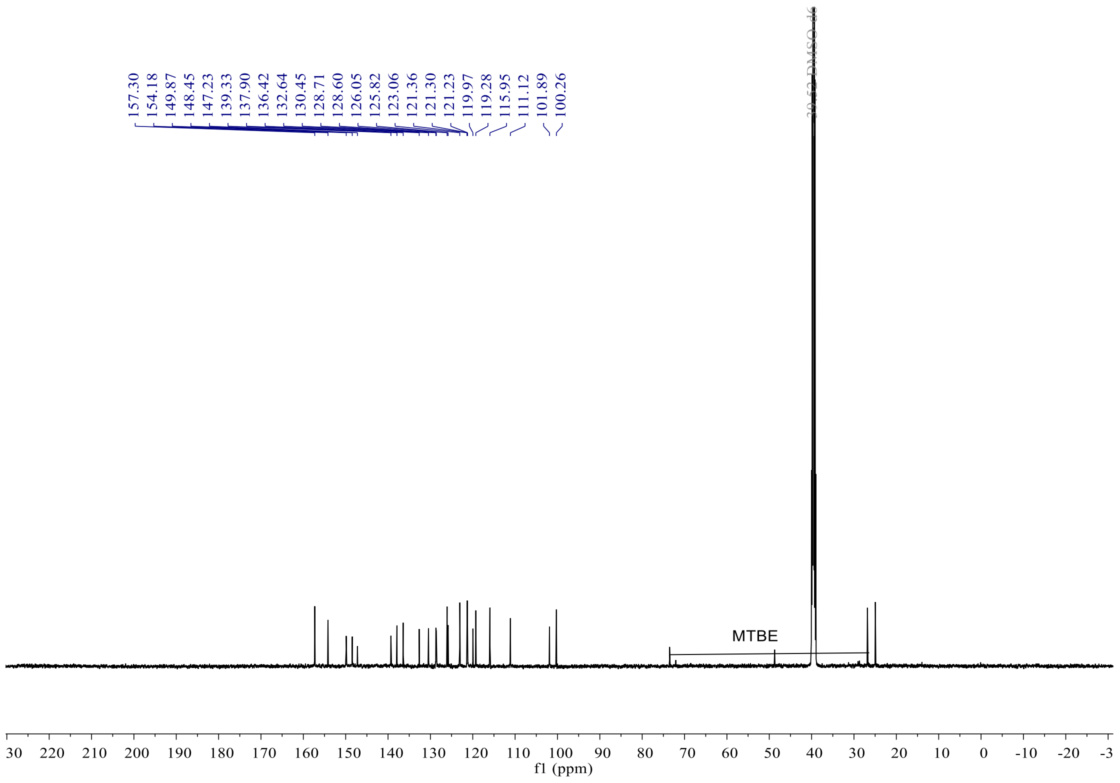

157.30
154.18
149.87
148.45
147.23
139.33
137.90
136.42
132.64
130.45
128.71
128.60
126.05
125.82
123.06
121.36
121.30
121.23
119.97
119.28
115.95
111.12
101.89
100.26
MTBE
30 220 210 200 190 180 170 160 150 140 130 120 110 100 90 80 70 60 50 40 30 20 10 0 -10 -20 -3
f1 (ppm)

Analysis Report

Graph

**Sample Name**	ftt-10708-069r2
**Application Name**	Admin App (Administrator)
**Method Name**	User2
**Configuration Name**	Configuration 1
**Version**	42
**Data Instrument Name**	Detector
**Data Channel Name**	156 Channel 1
**Notes**	
**Injection Number**	2

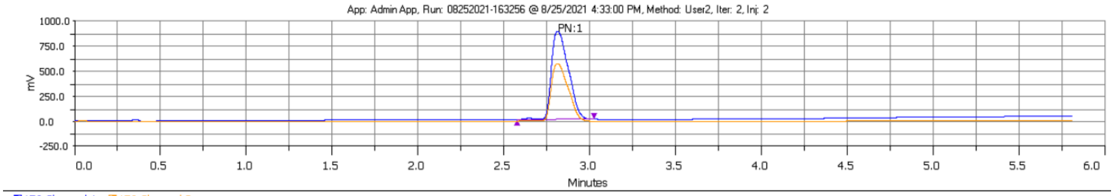

Sample Table

Injection Number	Sample Name	Sample Location	Peak Name	Retention Time (min)	Area (uVmin x100)	Area %	Height (mV)	Plate Number	
2	ftt-10708-069r2	Sample Zone->41	1	2.819	10171446.6667	100	880.935	3463.003	

D:\data\ftt-10708-069 8/24/2021 3:31:16 PM

ftt-10708-069 #73-74 RT: 1.32-1.33 AV: 2 NL: 7.46E8
F: + c ESI Full ms [105.00-1200.00]

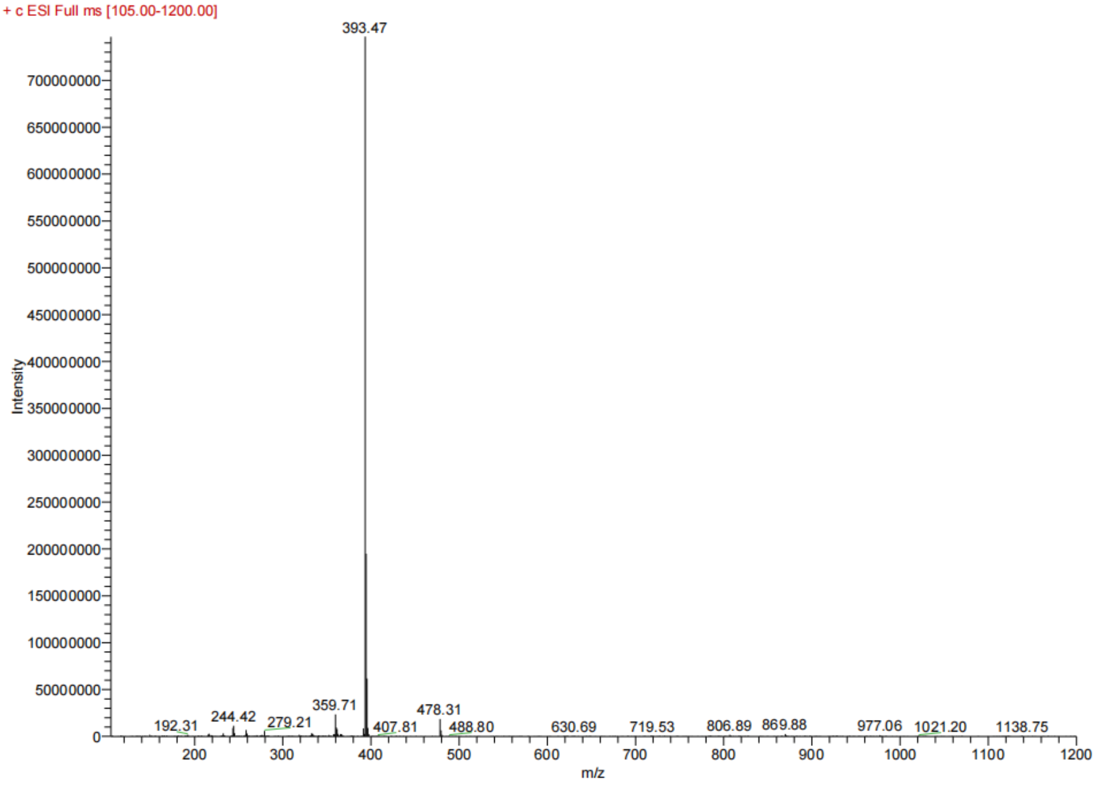

1.27 Compound 27

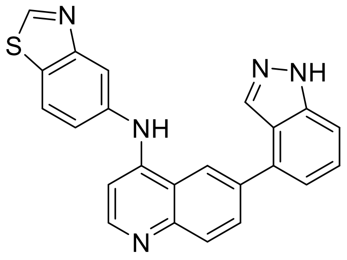

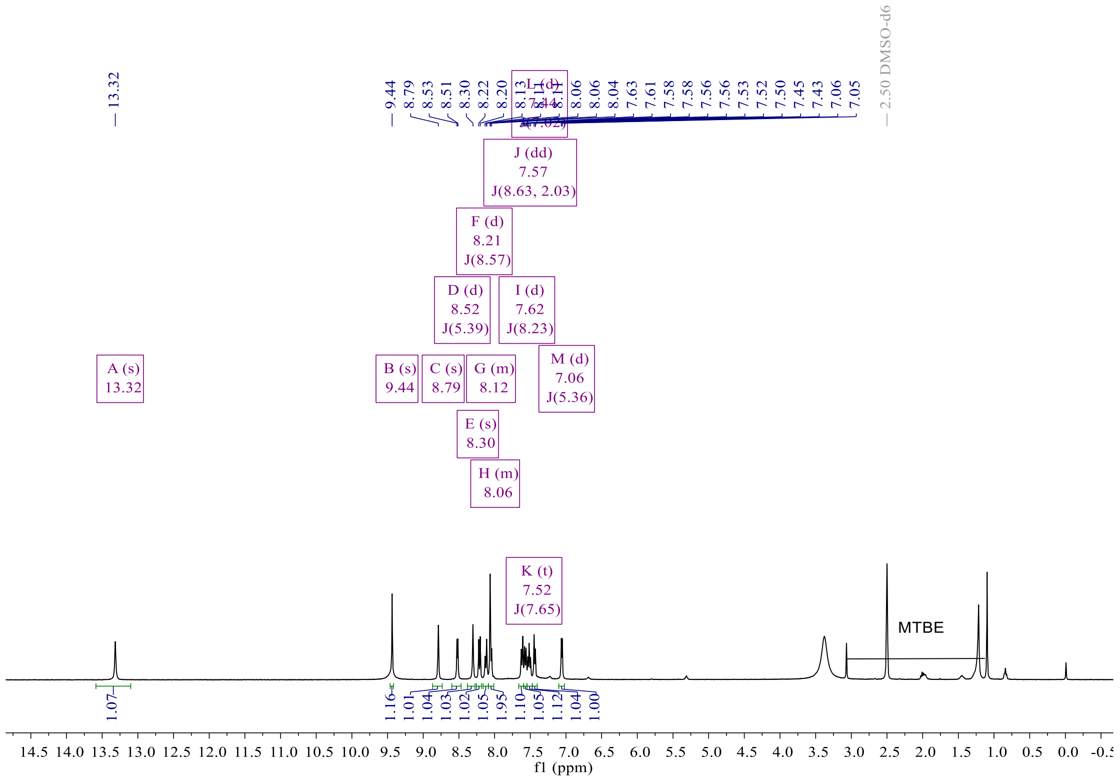

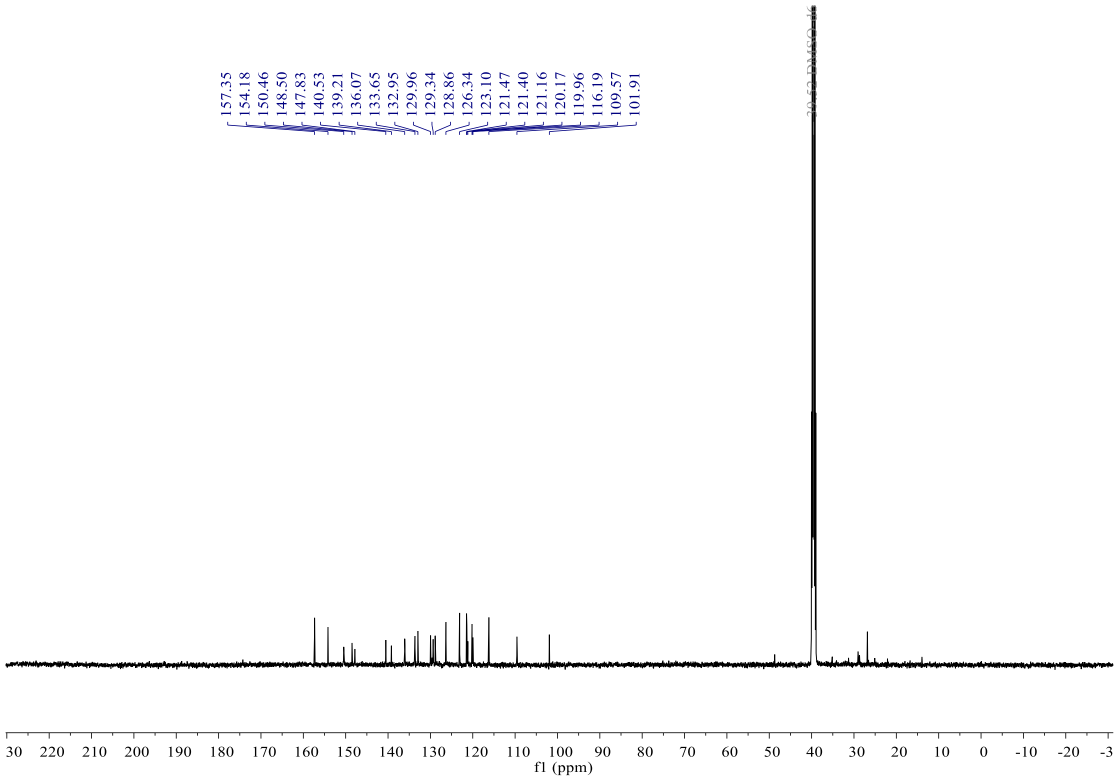

Analysis Report

Graph

**Sample Name**	ftt-10708-070
**Application Name**	Admin App (Administrator)
**Method Name**	User1
**Configuration Name**	Configuration 1
**Version**	48
**Data Instrument Name**	Detector
**Data Channel Name**	156 Channel 1
**Notes**	
**Injection Number**	3

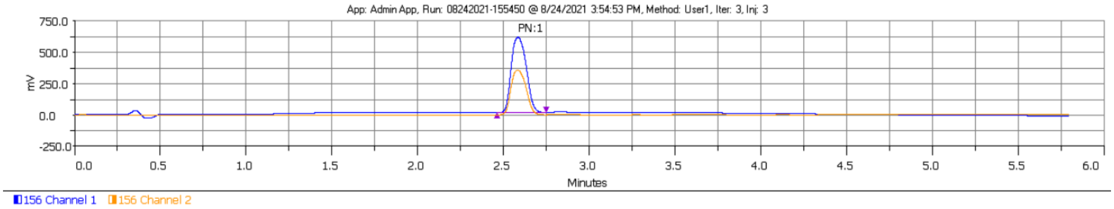

Sample Table

Injection Number	Sample Name	Sample Location	Peak Name	Retention Time (min)	Area (uVmin x100)	Area %	Height (mV)	Plate Number	
3	ftt-10708-070	Sample Zone->26	1	2.588	6126150.4167	100	601.324	3686.23	

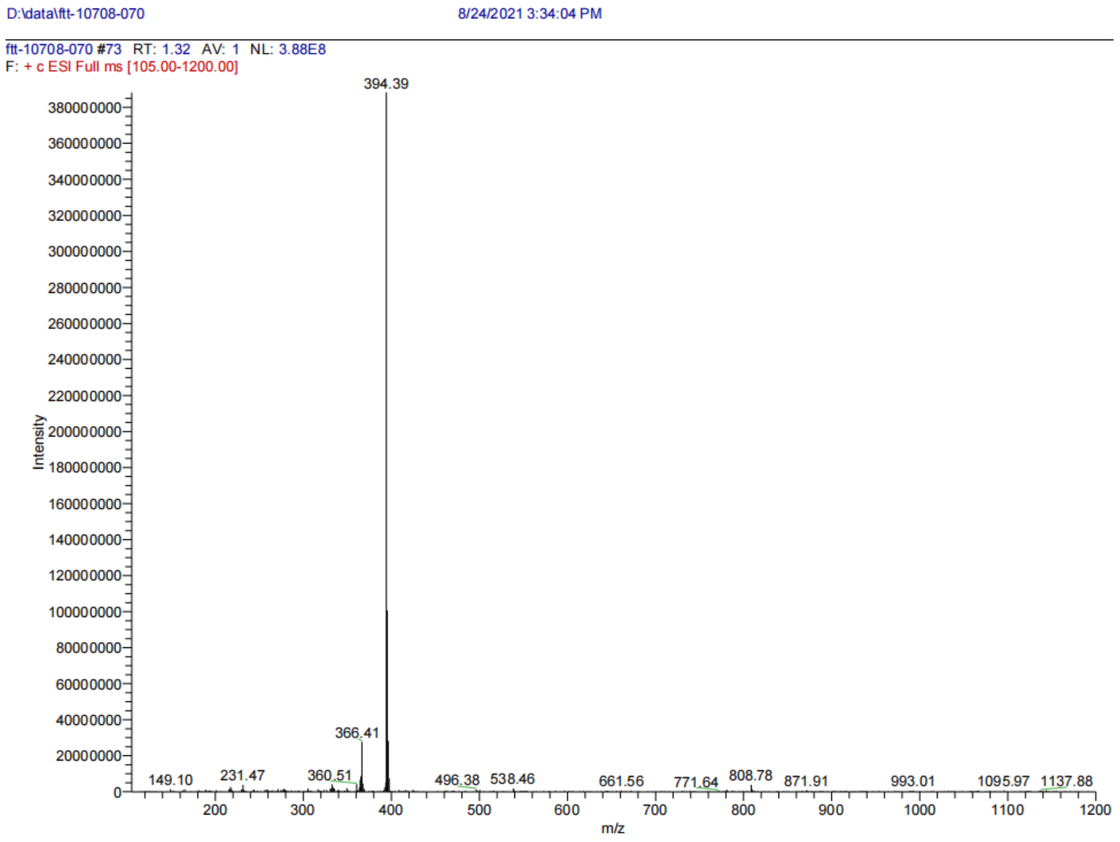

1.28 Compound 28

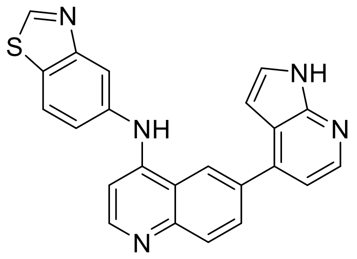

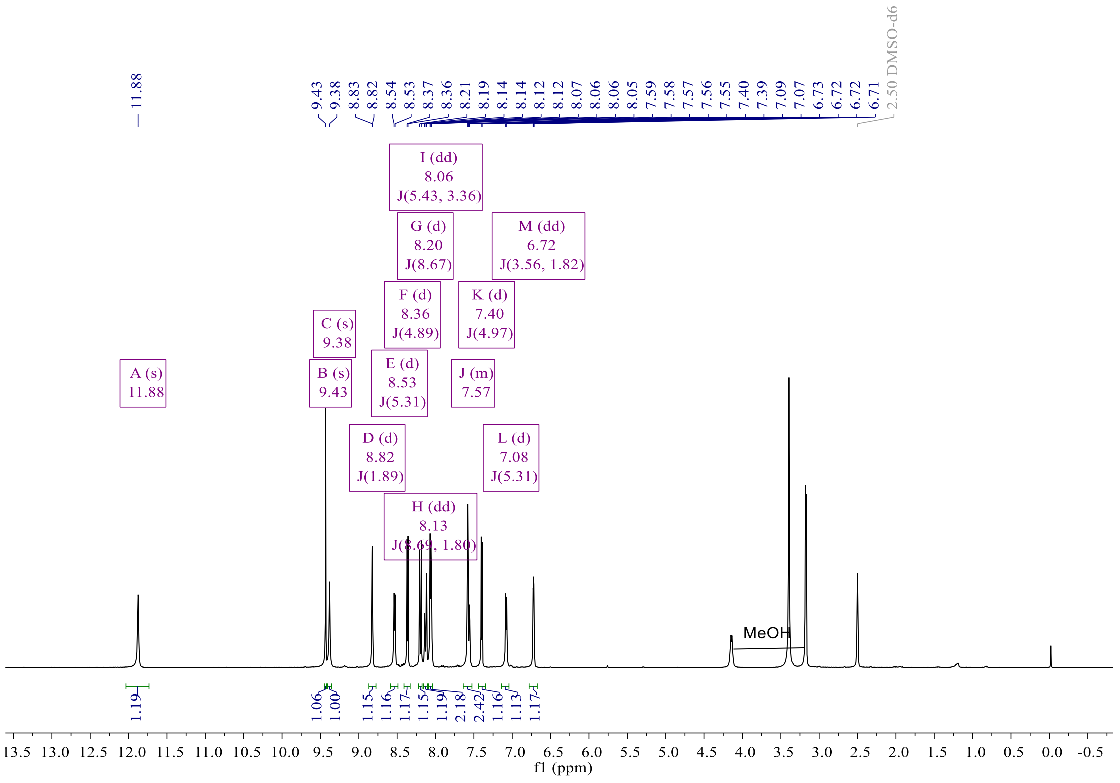

A (s) 11.88
B (s) 9.43
C (s) 9.38
D (d) 8.82 J(1.89)
E (d) 8.53 J(5.31)
F (d) 8.36 J(4.89)
G (d) 8.20 J(8.67)
H (dd) 8.13 J(8.69, 1.80)
I (dd) 8.06 J(5.43, 3.36)
J (m) 7.57
K (d) 7.40 J(4.97)
L (d) 7.08 J(5.31)
M (dd) 6.72 J(3.56, 1.82)
MeOH
2.50 DMSO-d6
f1 (ppm)

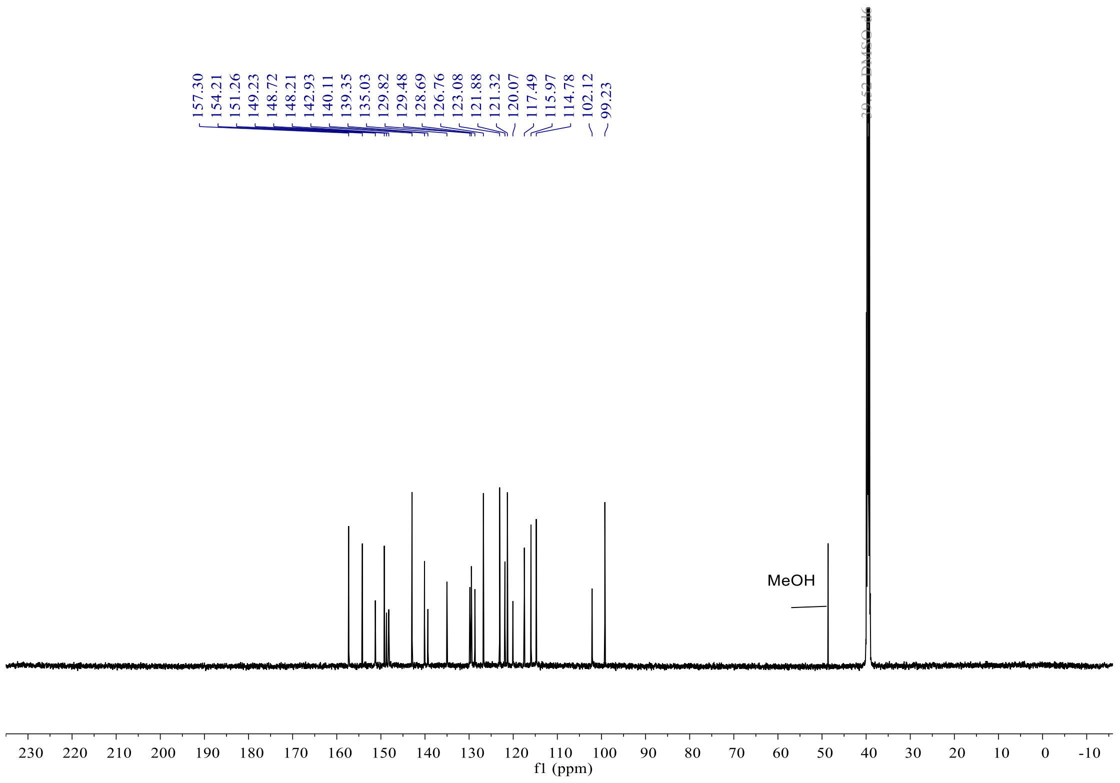

157.30
154.21
151.26
149.23
148.72
148.21
142.93
140.11
139.35
135.03
129.82
129.48
128.69
126.76
123.08
121.88
121.32
120.07
117.49
115.97
114.78
102.12
99.23
MeOH
f1 (ppm)

Analysis Report

Graph

**Sample Name**	ftt-10708-071
**Application Name**	Admin App (Administrator)
**Method Name**	User1
**Configuration Name**	Configuration 1
**Version**	48
**Data Instrument Name**	Detector
**Data Channel Name**	156 Channel 1
**Notes**	
**Injection Number**	6

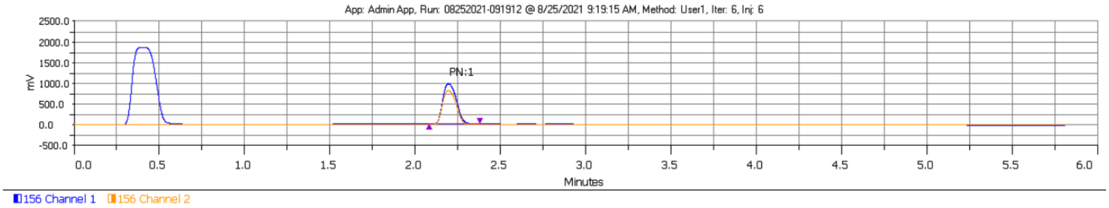

Sample Table

Injection Number	Sample Name	Sample Location	Peak Name	Retention Time (min)	Area (uVmin x100)	Area %	Height (mV)	Plate Number	
6	ftt-10708-071	Sample Zone->28	1	2.2	9082900.8333	100	972.24	3229.498	

D:\RawData\...\20210826\ESIL202107866 08/26/21 15:42:26 C3-FTT-10708-071
Thermo Fisher FINNIGAN LTQ\ESI-LR\BY HuangQiongPing
ESIL202107866 #42 RT: 0.15 AV: 1 SB: 35 0.01-0.12 NL: 2.04E6
T: ITMS + c ESI Full ms [50.00-2000.00]

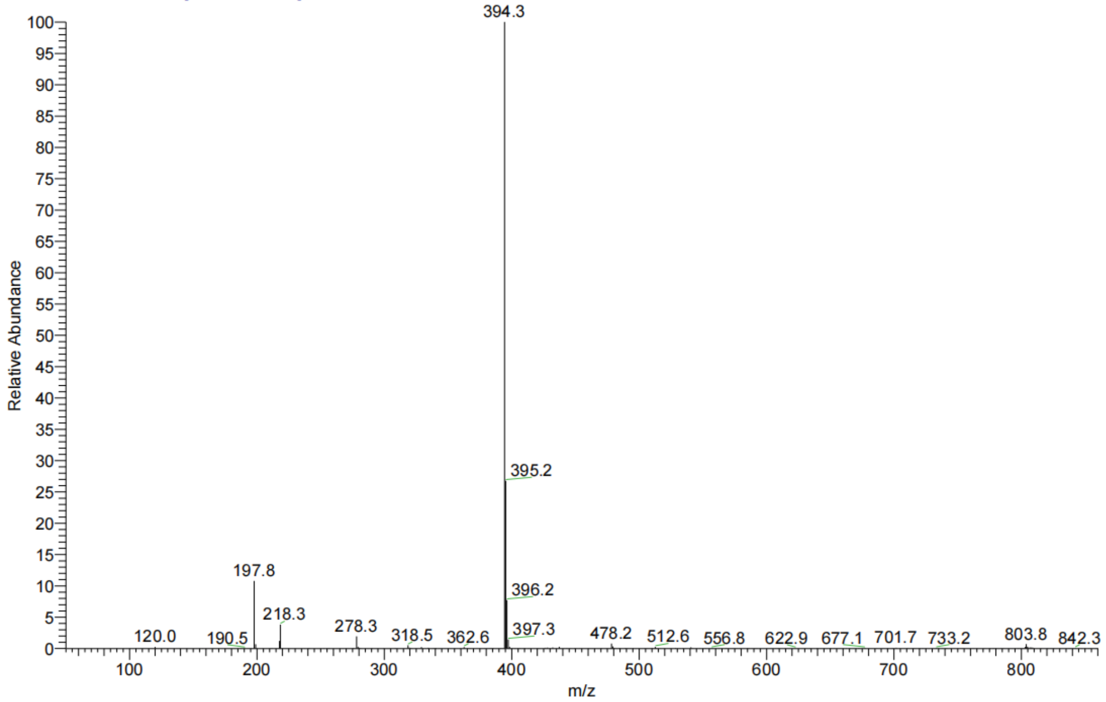

1.29 Compound 29

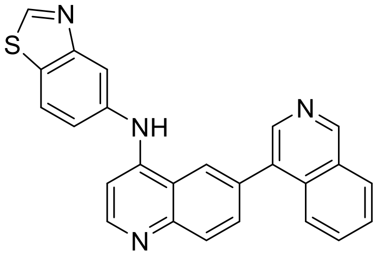

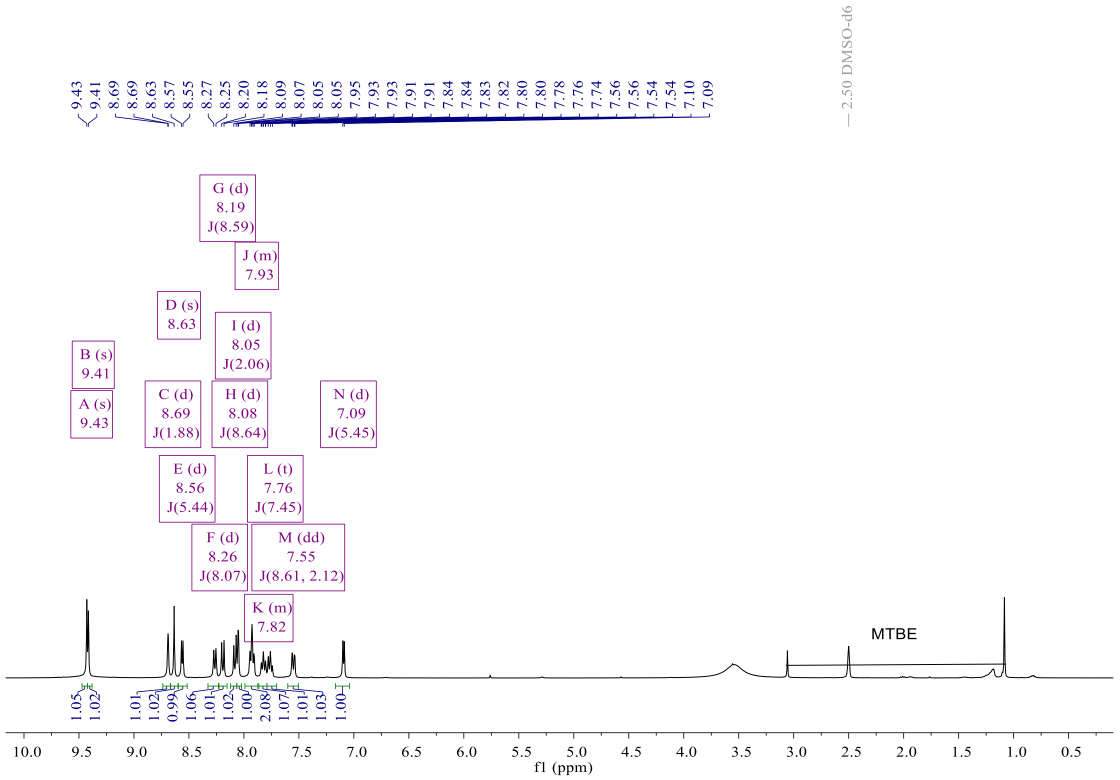

9.43 9.41 8.69 8.69 8.63 8.57 8.55 8.27 8.25 8.20 8.18 8.09 8.07 8.05 8.05 7.95 7.93 7.93 7.91 7.91 7.84 7.84 7.83 7.82 7.80 7.80 7.78 7.76 7.74 7.56 7.56 7.54 7.54 7.10 7.09
2.50 DMSO-d6
G (d) 8.19 J(8.59)
J (m) 7.93
D (s) 8.63
I (d) 8.05 J(2.06)
B (s) 9.41
A (s) 9.43
C (d) 8.69 J(1.88)
H (d) 8.08 J(8.64)
N (d) 7.09 J(5.45)
E (d) 8.56 J(5.44)
L (t) 7.76 J(7.45)
F (d) 8.26 J(8.07)
M (dd) 7.55 J(8.61, 2.12)
K (m) 7.82
MTBE
1.05 1.02 1.01 1.02 0.99 1.06 1.01 1.02 1.00 2.08 1.07 1.01 1.03 1.00
10.0 9.5 9.0 8.5 8.0 7.5 7.0 6.5 6.0 5.5 5.0 4.5 4.0 3.5 3.0 2.5 2.0 1.5 1.0 0.5
f1 (ppm)

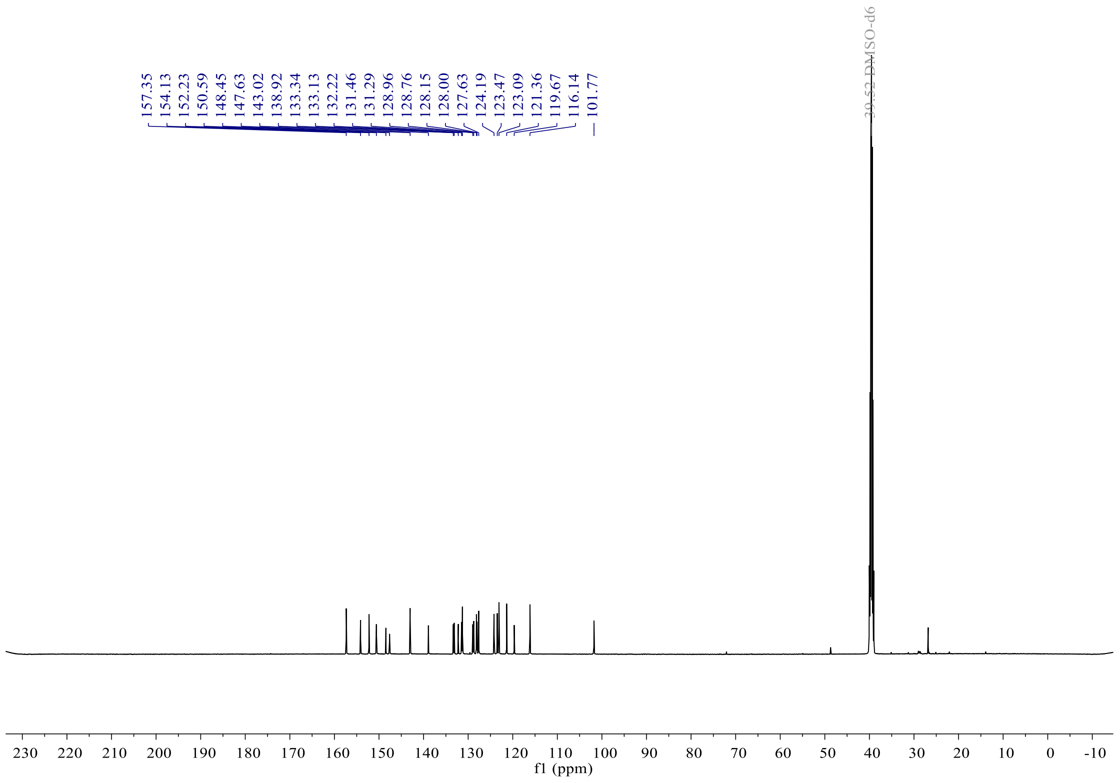

157.35 154.13 152.23 150.59 148.45 147.63 143.02 138.92 133.34 133.13 132.22 131.46 131.29 128.96 128.76 128.15 128.00 127.63 124.19 123.47 123.09 121.36 119.67 116.14 101.77
39.52 DMSO-d6
230 220 210 200 190 180 170 160 150 140 130 120 110 100 90 80 70 60 50 40 30 20 10 0 -10
f1 (ppm)

Analysis Report

Graph

**Sample Name**	ftt-10708-072
**Application Name**	Admin App (Administrator)
**Method Name**	User1
**Configuration Name**	Configuration 1
**Version**	48
**Data Instrument Name**	Detector
**Data Channel Name**	156 Channel 1
**Notes**	
**Injection Number**	3

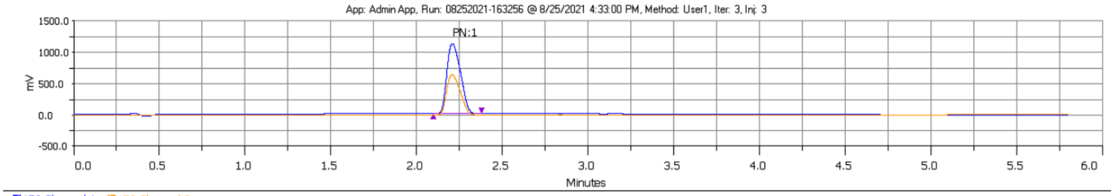

Sample Table

Injection Number	Sample Name	Sample Location	Peak Name	Retention Time (min)	Area (uVmin x100)	Area %	Height (mV)	Plate Number	
3	ftt-10708-072	Sample Zone->42	1	2.213	10320388.3333	100	1121.386	3277.126	

D:\RawData\...\20210826\ESIL202107868 08/26/21 15:46:16 C3-FTT-10708-073
Thermo Fisher FINNIGAN LTQ\ESI-LR\BY HuangQiongPing
ESIL202107868 #160-163 RT: 0.53-0.53 AV: 4 SB: 35 0.01-0.12 NL: 5.97E6
T: ITMS + c ESI Full ms [50.00-2000.00]

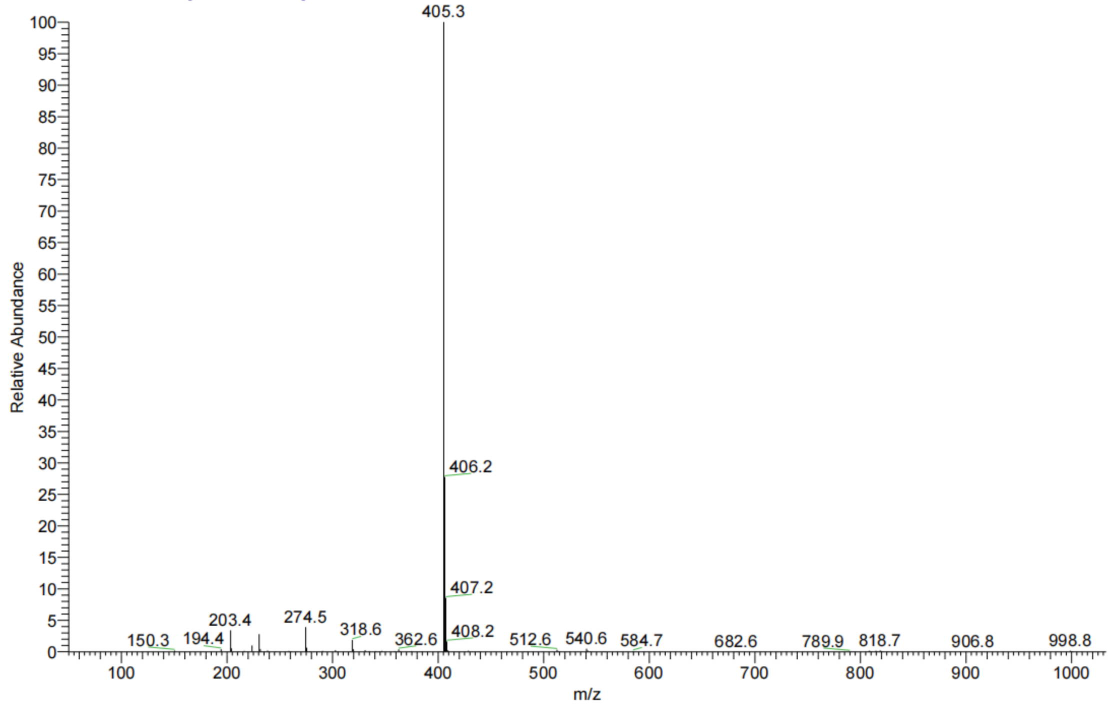

1.30 Compound 30

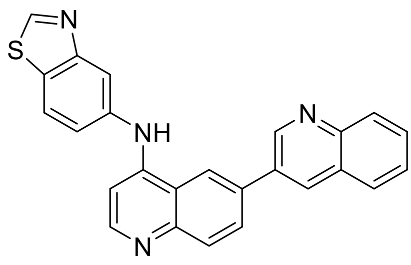

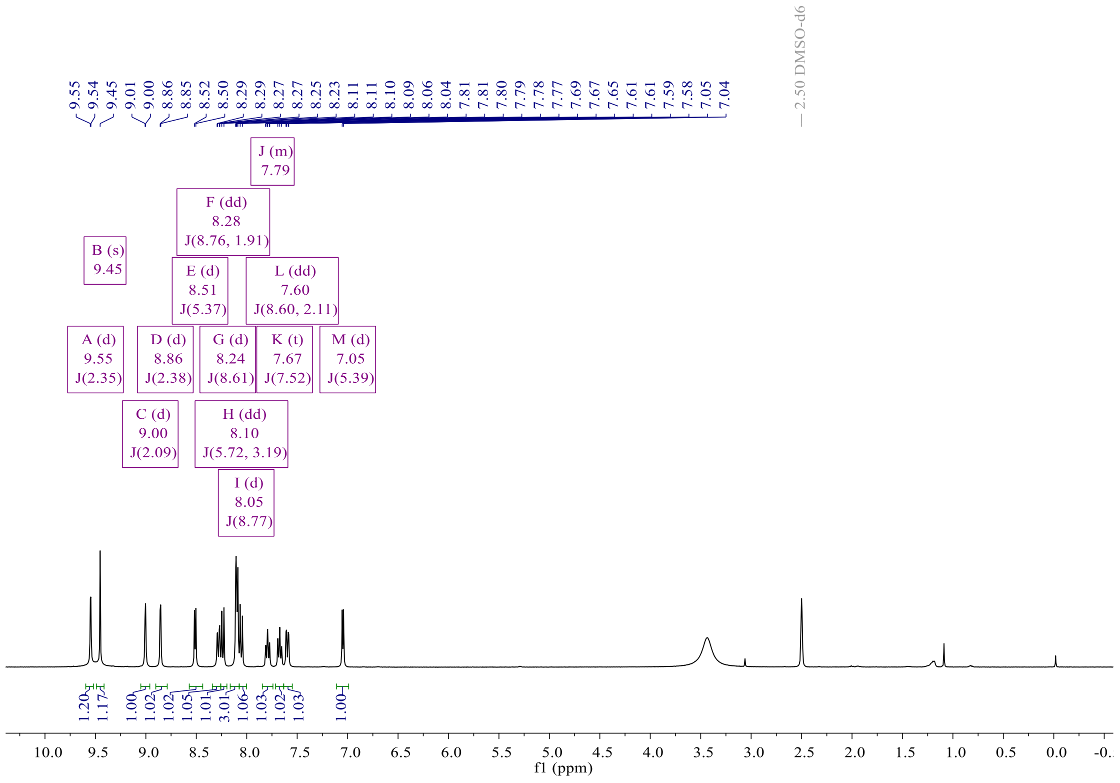
9.55
9.54
9.45
9.01
9.00
8.86
8.85
8.52
8.50
8.29
8.29
8.27
8.27
8.25
8.23
8.11
8.11
8.10
8.09
8.06
8.04
7.81
7.81
7.80
7.79
7.78
7.77
7.69
7.67
7.65
7.61
7.61
7.59
7.58
7.05
7.04
2.50 DMSO-d6
J (m) 7.79
F (dd) 8.28 J(8.76, 1.91)
B (s) 9.45
E (d) 8.51 J(5.37)
L (dd) 7.60 J(8.60, 2.11)
A (d) 9.55 J(2.35)
D (d) 8.86 J(2.38)
G (d) 8.24 J(8.61)
K (t) 7.67 J(7.52)
M (d) 7.05 J(5.39)
C (d) 9.00 J(2.09)
H (dd) 8.10 J(5.72, 3.19)
I (d) 8.05 J(8.77)
1.20
1.17
1.00
1.02
1.02
1.05
1.01
3.01
1.06
1.03
1.02
1.03
1.00
f1 (ppm)

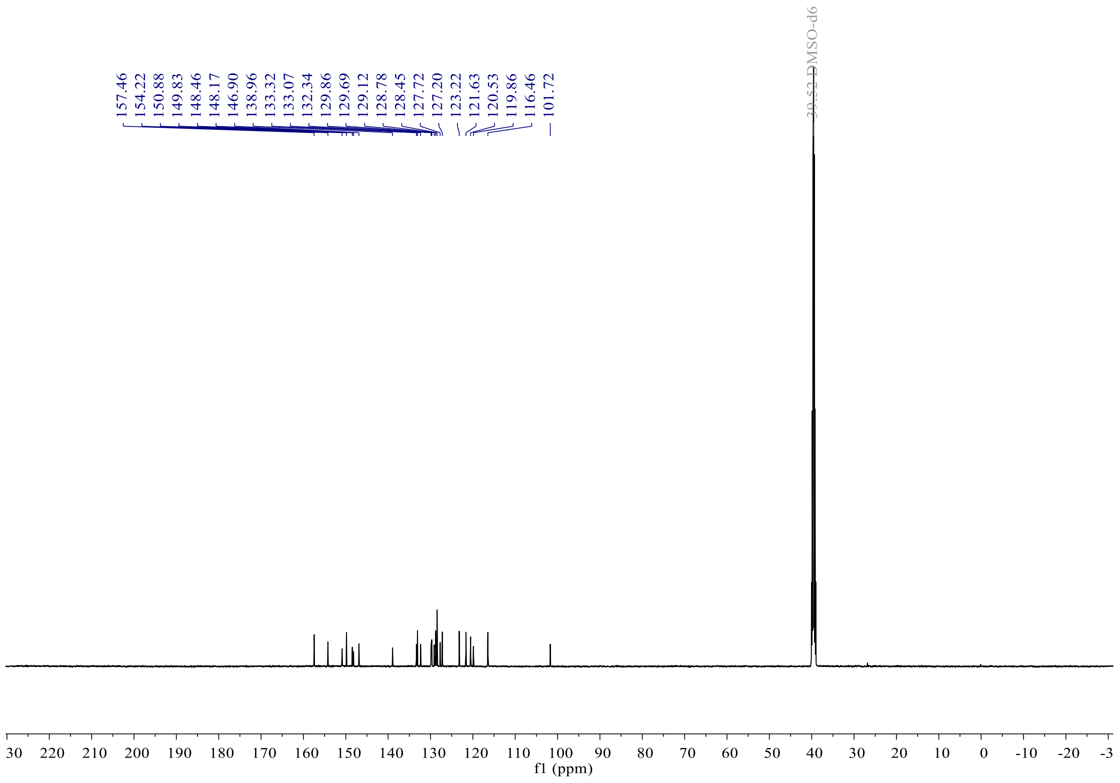
157.46
154.22
150.88
149.83
148.46
148.17
146.90
138.96
133.32
133.07
132.34
129.86
129.69
129.12
128.78
128.45
127.72
127.20
123.22
121.63
120.53
119.86
116.46
101.72
39.52 DMSO-d6
f1 (ppm)

Analysis Report

Graph

**Sample Name**	ftt-10708-073
**Application Name**	Admin App (Administrator)
**Method Name**	User2
**Configuration Name**	Configuration 1
**Version**	42
**Data Instrument Name**	Detector
**Data Channel Name**	156 Channel 1
**Notes**	
**Injection Number**	4

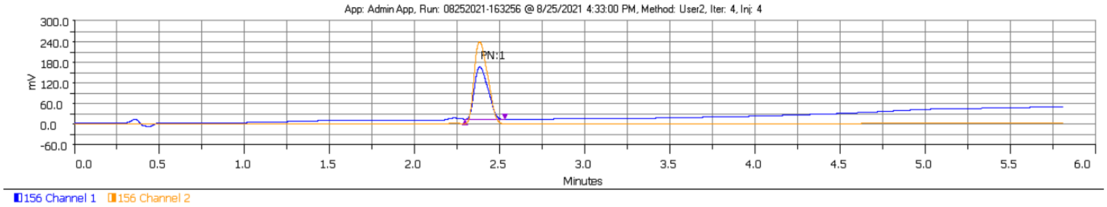

Sample Table

Injection Number	Sample Name	Sample Location	Peak Name	Retention Time (min)	Area (uVmin x100)	Area %	Height (mV)	Plate Number	
4	ftt-10708-073	Sample Zone->43	1	2.387	1405900.4167	100	151.987	3774.812	

D:\RawData\...\20210826\ESIL202107868 08/26/21 15:46:16 C3-FTT-10708-073
Thermo Fisher FINNIGAN LTQ\ESI-LR\BY HuangQiongPing
ESIL202107868 #160-163 RT: 0.53-0.53 AV: 4 SB: 35 0.01-0.12 NL: 5.97E6
T: ITMS + c ESI Full ms [50.00-2000.00]

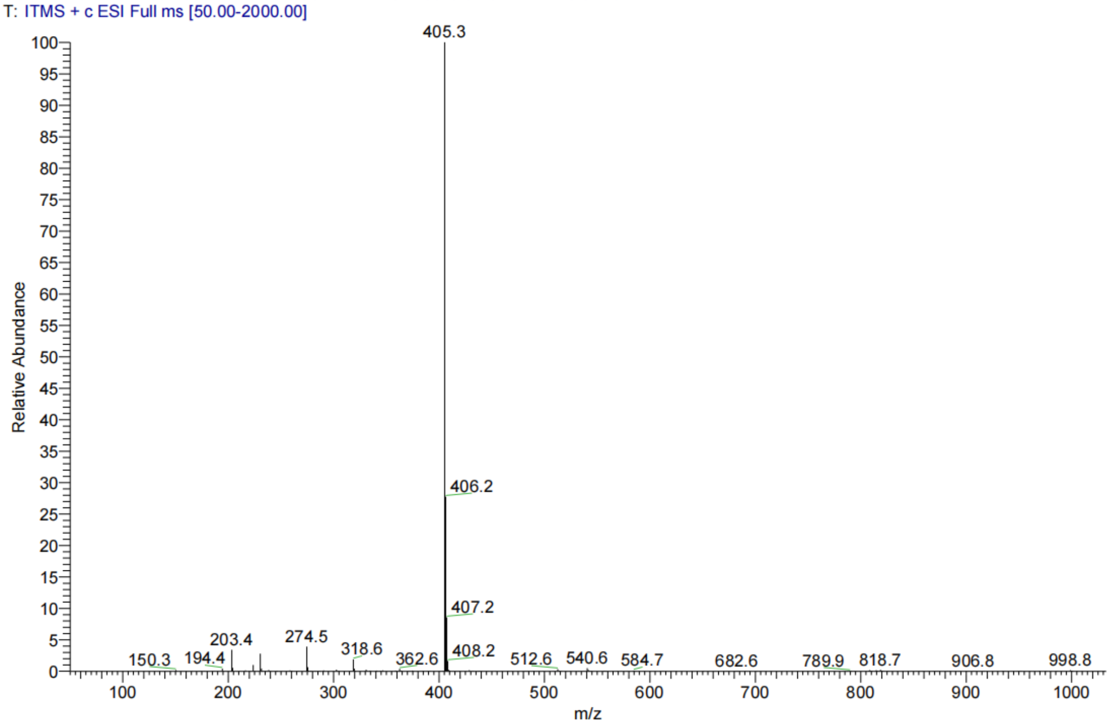

1.31 Compound 31

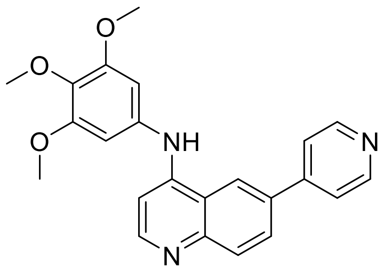

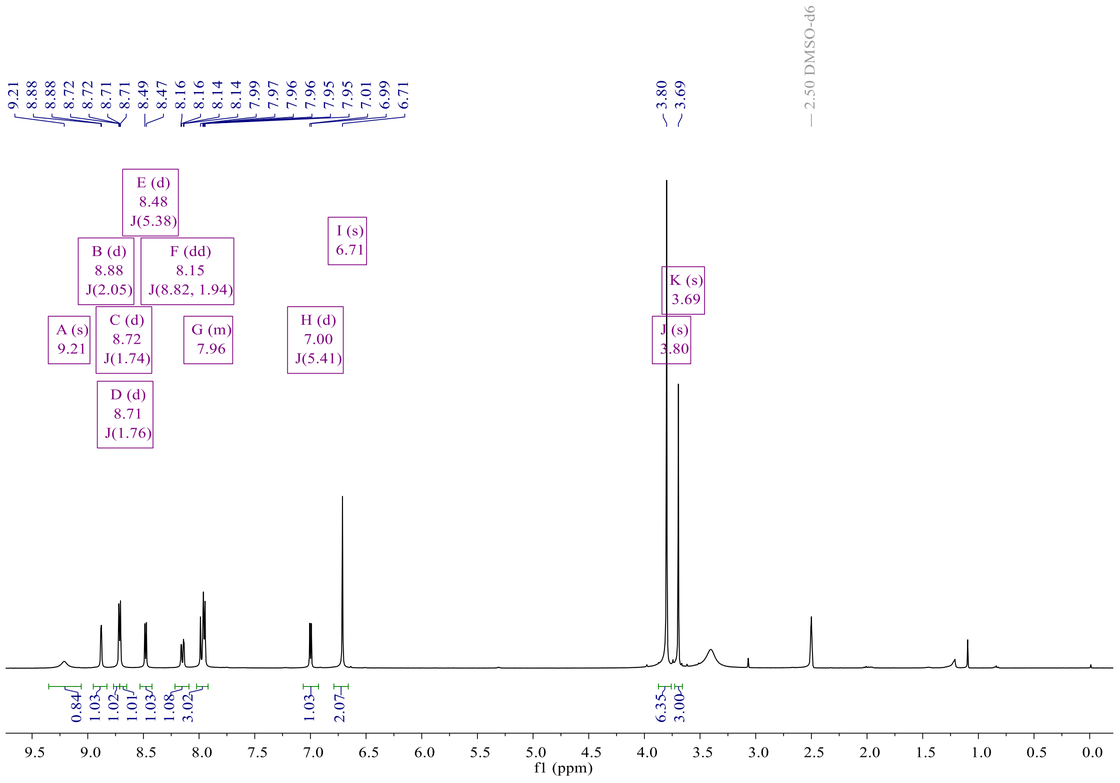

9.21
8.88
8.88
8.72
8.72
8.71
8.71
8.49
8.47
8.16
8.16
8.14
8.14
7.99
7.97
7.96
7.96
7.95
7.95
7.01
6.99
6.71
3.80
3.69
2.50 DMSO-d6
E (d) 8.48 J(5.38)
B (d) 8.88 J(2.05)
F (dd) 8.15 J(8.82, 1.94)
I (s) 6.71
K (s) 3.69
A (s) 9.21
C (d) 8.72 J(1.74)
G (m) 7.96
H (d) 7.00 J(5.41)
J (s) 3.80
D (d) 8.71 J(1.76)
0.84
1.03
1.02
1.01
1.03
1.08
3.02
1.03
2.07
6.35
3.00
9.5 9.0 8.5 8.0 7.5 7.0 6.5 6.0 5.5 5.0 4.5 4.0 3.5 3.0 2.5 2.0 1.5 1.0 0.5 0.0
f1 (ppm)

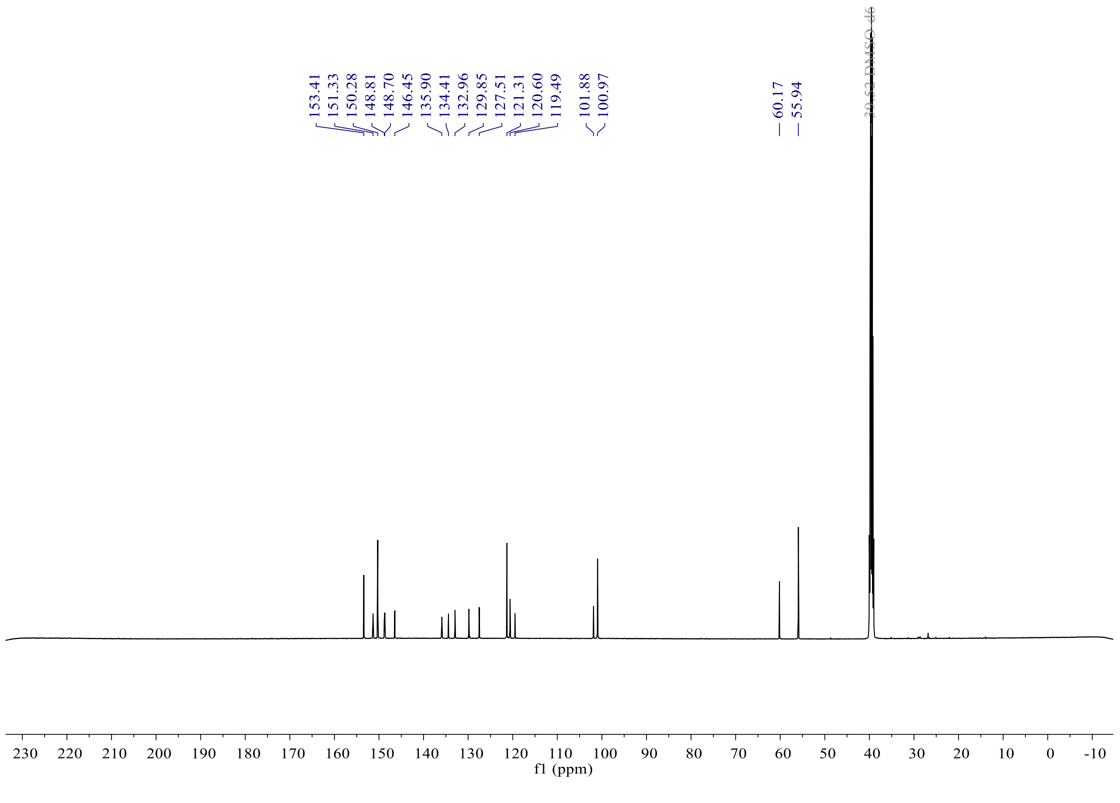

153.41
151.33
150.28
148.81
148.70
146.45
135.90
134.41
132.96
129.85
127.51
121.31
120.60
119.49
101.88
100.97
60.17
55.94
230 220 210 200 190 180 170 160 150 140 130 120 110 100 90 80 70 60 50 40 30 20 10 0 -10
f1 (ppm)

Analysis Report

Graph

**Sample Name**	ftt-10708-116
**Application Name**	Admin App (Administrator)
**Method Name**	User2
**Configuration Name**	Configuration 1
**Version**	42
**Data Instrument Name**	Detector
**Data Channel Name**	156 Channel 1
**Notes**	
**Injection Number**	10

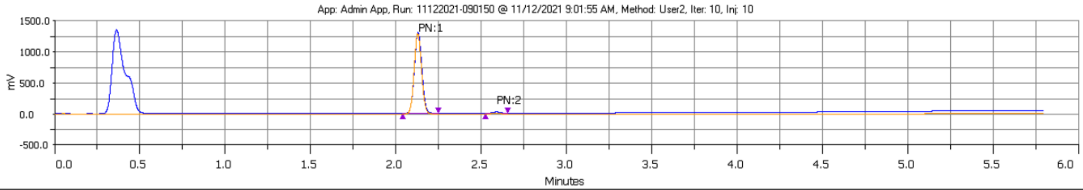

Sample Table

Injection Number	Sample Name	Sample Location	Peak Name	Retention Time (min)	Area (uVmin x100)	Area %	Height (mV)	Plate Number
10	ftt-10708-116	Sample Zone->35	1	2.132	6662266.6667	98.565	1305.441	11021.491
10	ftt-10708-116	Sample Zone->35	2	2.593	97002.0833	1.435	19.735	17373.498

D:\data\ftt-10708-116 11/12/2021 4:13:01 PM

ftt-10708-116 #76-78 RT: 1.30-1.33 AV: 3 NL: 2.59E9
F: + c ESI Full ms [105.00-1200.00]

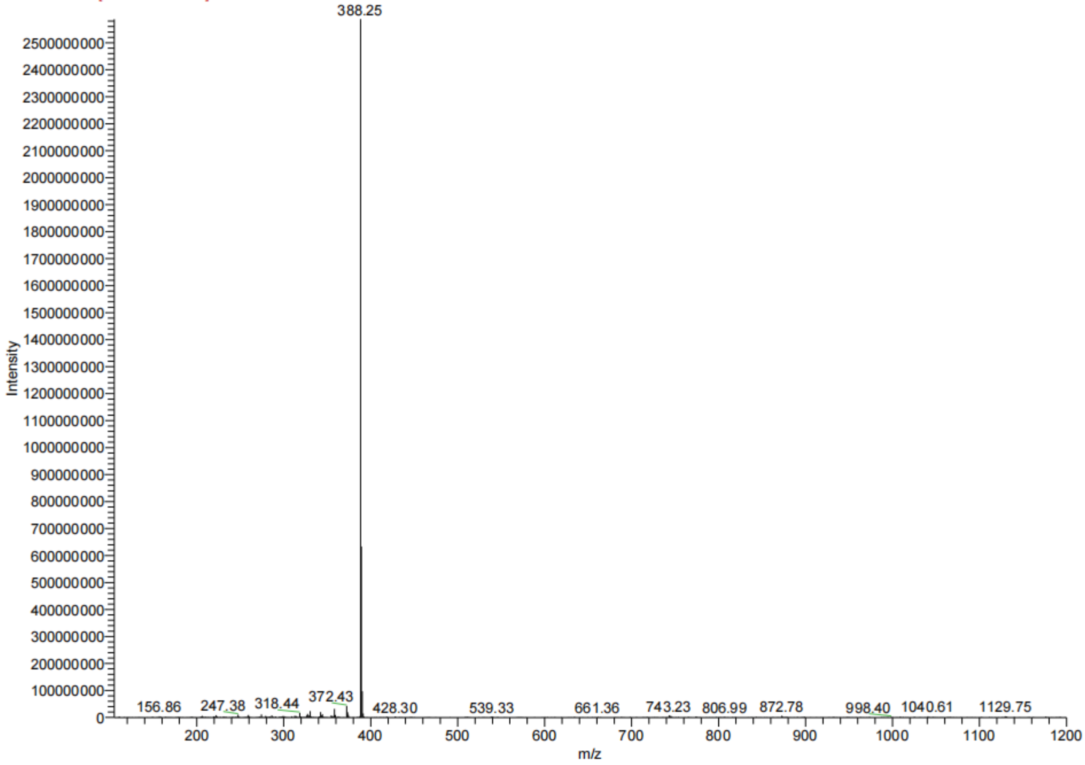

1.32 Compound 32

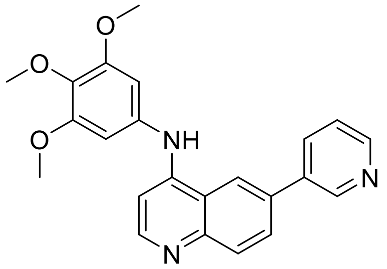

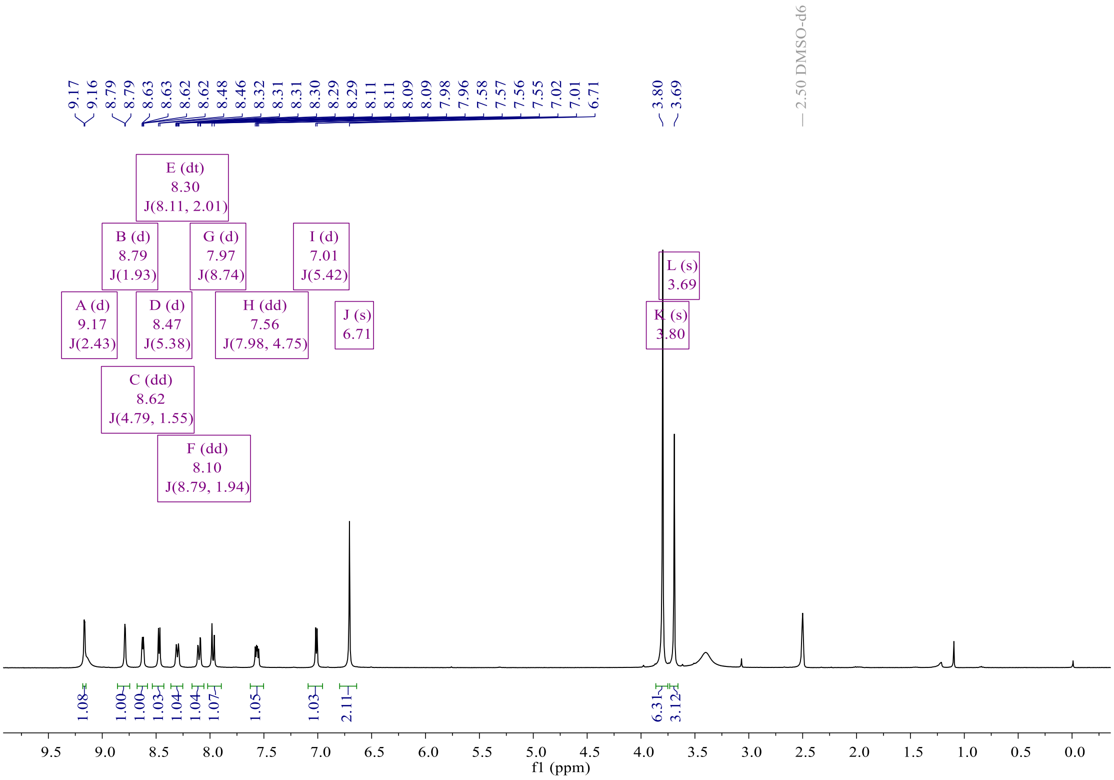

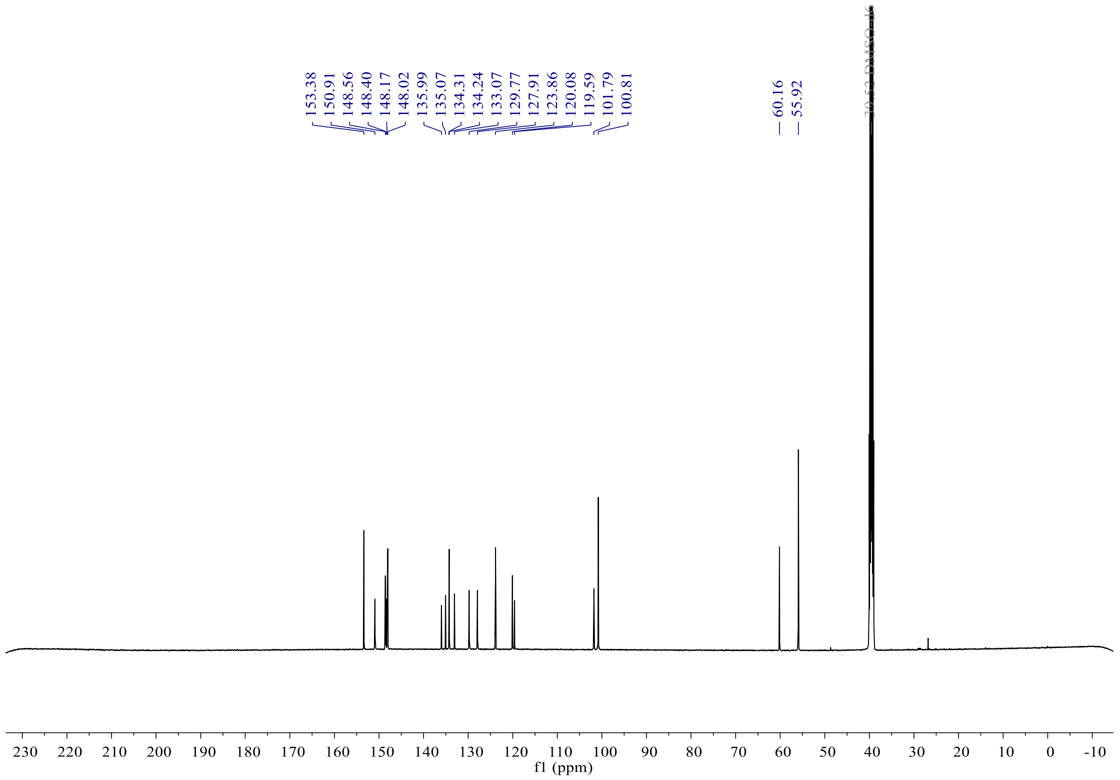

Analysis Report

Graph

**Sample Name**	ftt-10708-117
**Application Name**	Admin App (Administrator)
**Method Name**	User1
**Configuration Name**	Configuration 1
**Version**	48
**Data Instrument Name**	Detector
**Data Channel Name**	156 Channel 1
**Notes**	
**Injection Number**	11

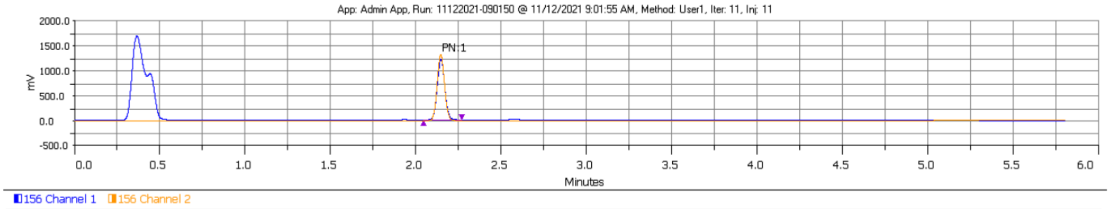

Sample Table

Injection Number	Sample Name	Sample Location	Peak Name	Retention Time (min)	Area (uVmin x100)	Area %	Height (mV)	Plate Number	
11	ftt-10708-117	Sample Zone->36	1	2.15	6170749.1667	100	1211.402	11283.315	

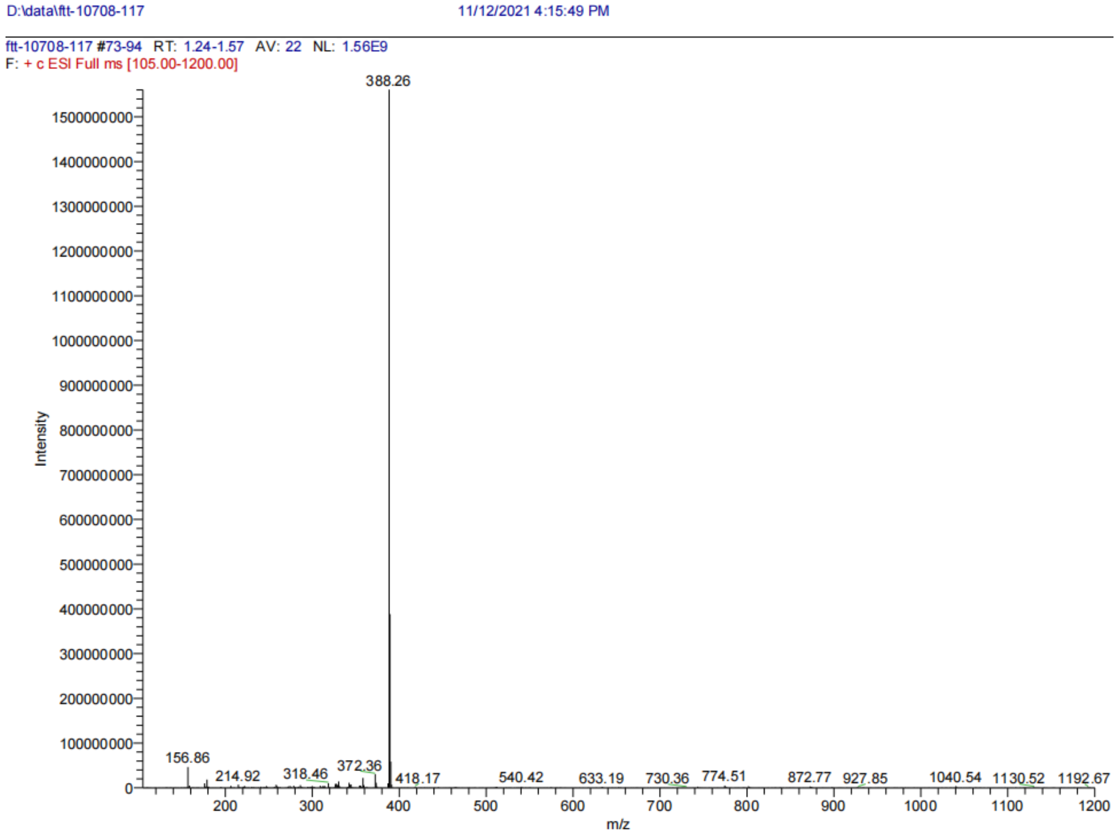

1.33 Compound 33

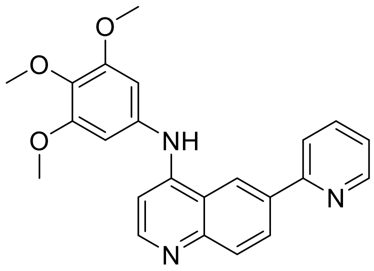

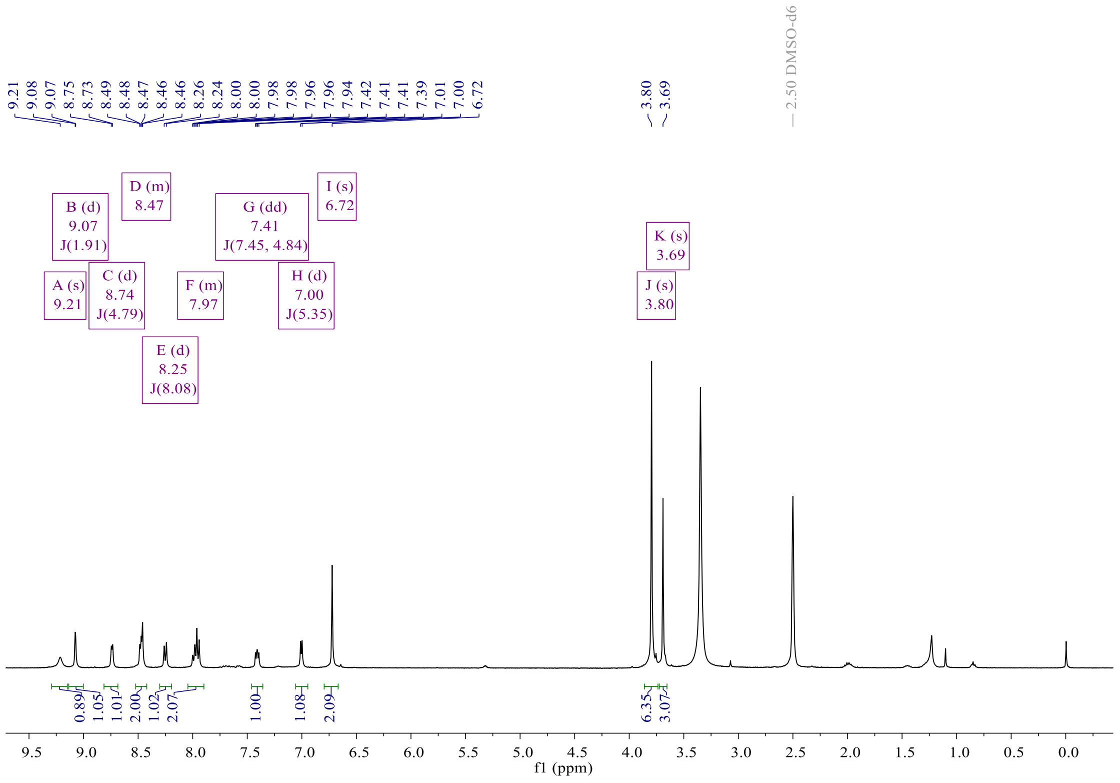

9.21 9.08 9.07 8.75 8.73 8.49 8.48 8.47 8.46 8.46 8.26 8.24 8.00 8.00 7.98 7.98 7.96 7.94 7.42 7.41 7.41 7.39 7.01 7.00 6.72
3.80 3.69
2.50 DMSO-d6
A (s) 9.21
B (d) 9.07 J(1.91)
C (d) 8.74 J(4.79)
D (m) 8.47
E (d) 8.25 J(8.08)
F (m) 7.97
G (dd) 7.41 J(7.45, 4.84)
H (d) 7.00 J(5.35)
I (s) 6.72
J (s) 3.80
K (s) 3.69
0.89 1.05 1.01 2.00 1.02 2.07 1.00 1.08 2.09 6.35 3.07
9.5 9.0 8.5 8.0 7.5 7.0 6.5 6.0 5.5 5.0 4.5 4.0 3.5 3.0 2.5 2.0 1.5 1.0 0.5 0.0
f1 (ppm)

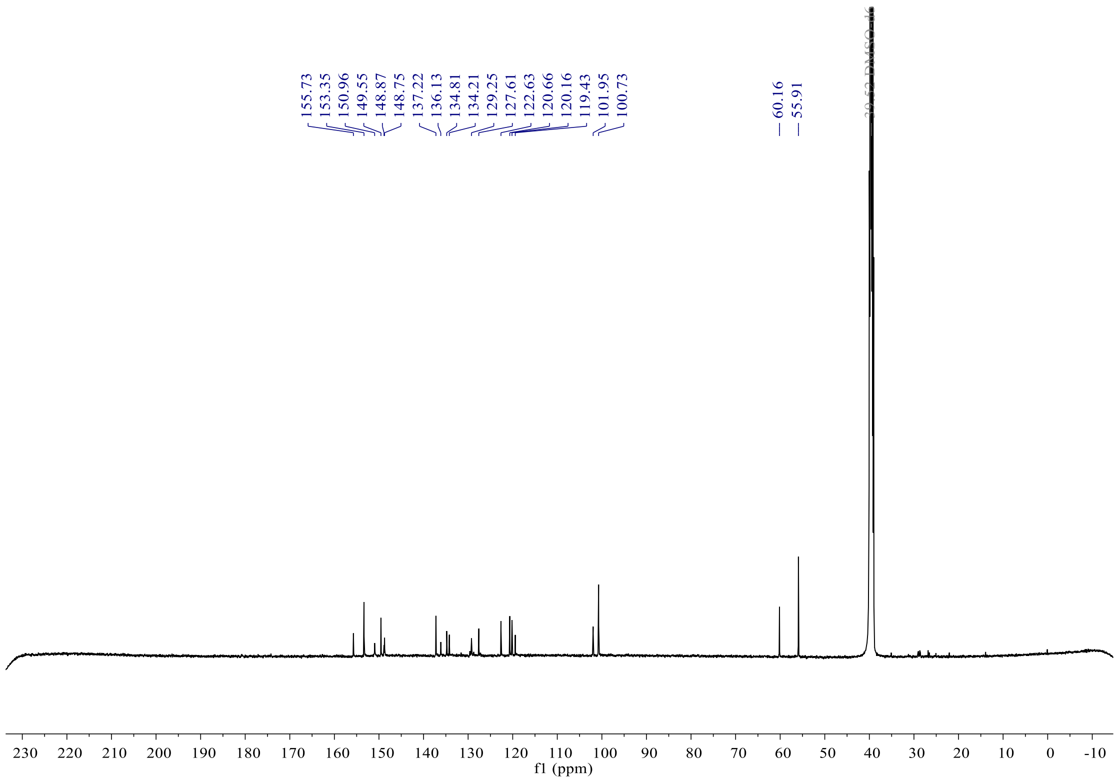

155.73 153.35 150.96 149.55 148.87 148.75 137.22 136.13 134.81 134.21 129.25 127.61 122.63 120.66 120.16 119.43 101.95 100.73
60.16 55.91
230 220 210 200 190 180 170 160 150 140 130 120 110 100 90 80 70 60 50 40 30 20 10 0 -10
f1 (ppm)

Analysis Report

Graph

**Sample Name**	ftt-10708-118
**Application Name**	Admin App (Administrator)
**Method Name**	User2
**Configuration Name**	Configuration 1
**Version**	42
**Data Instrument Name**	Detector
**Data Channel Name**	156 Channel 1
**Notes**	
**Injection Number**	12

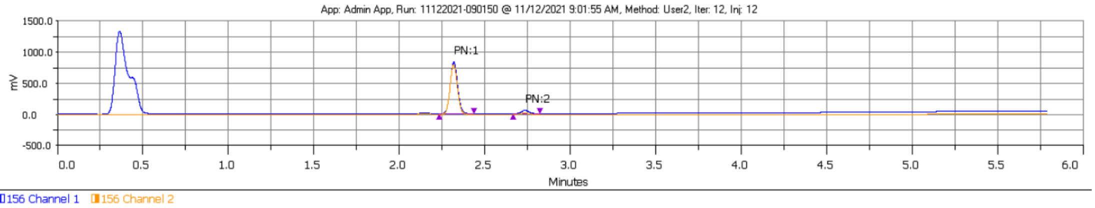

Sample Table

Injection Number	Sample Name	Sample Location	Peak Name	Retention Time (min)	Area (uVmin x100)	Area %	Height (mV)	Plate Number	
12	ftt-10708-118	Sample Zone->38	1	2.321	4168574.5833	94.106	829.939	13631.441	
12	ftt-10708-118	Sample Zone->38	2	2.738	261076.6667	5.894	51.825	18718.551	

D:\data\ftt-10708-118 11/12/2021 4:18:37 PM

ftt-10708-118 #73-93 RT: 1.24-1.56 AV: 21 NL: 8.03E8
F: + c ESI Full ms [105.00-1200.00]

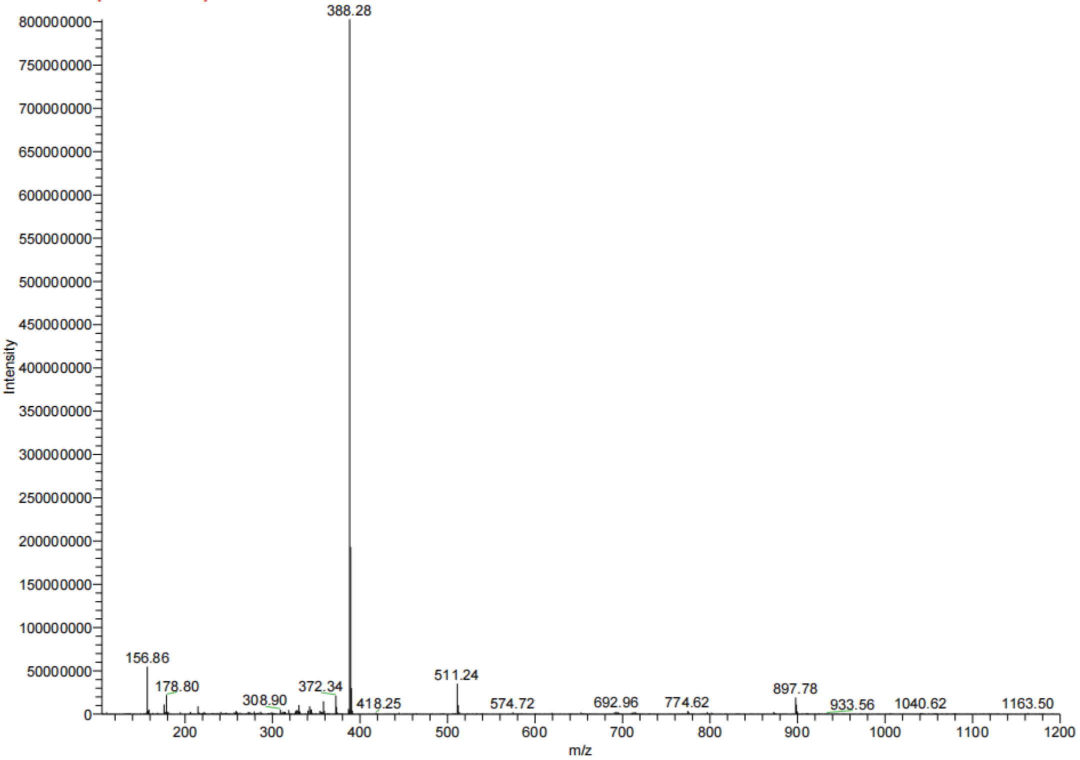

1.34 Compound 34

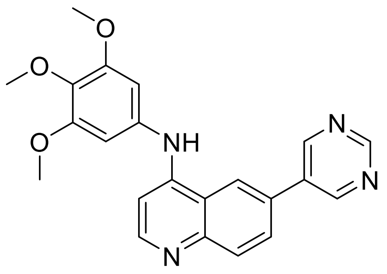

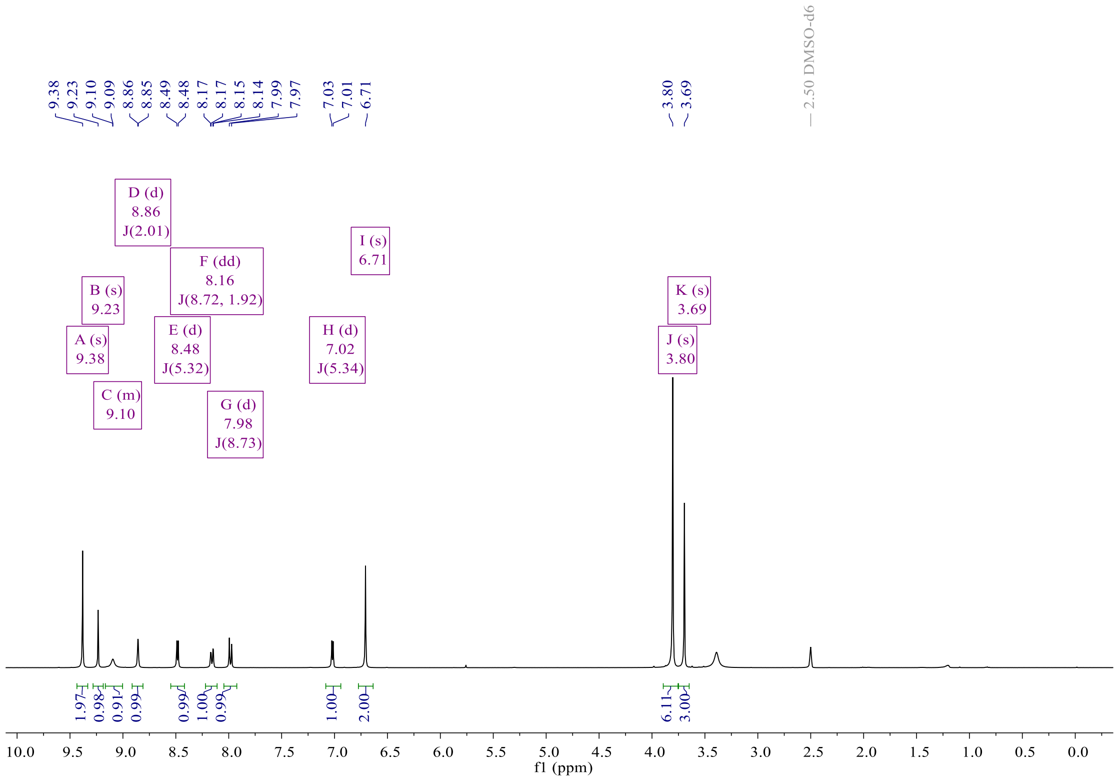

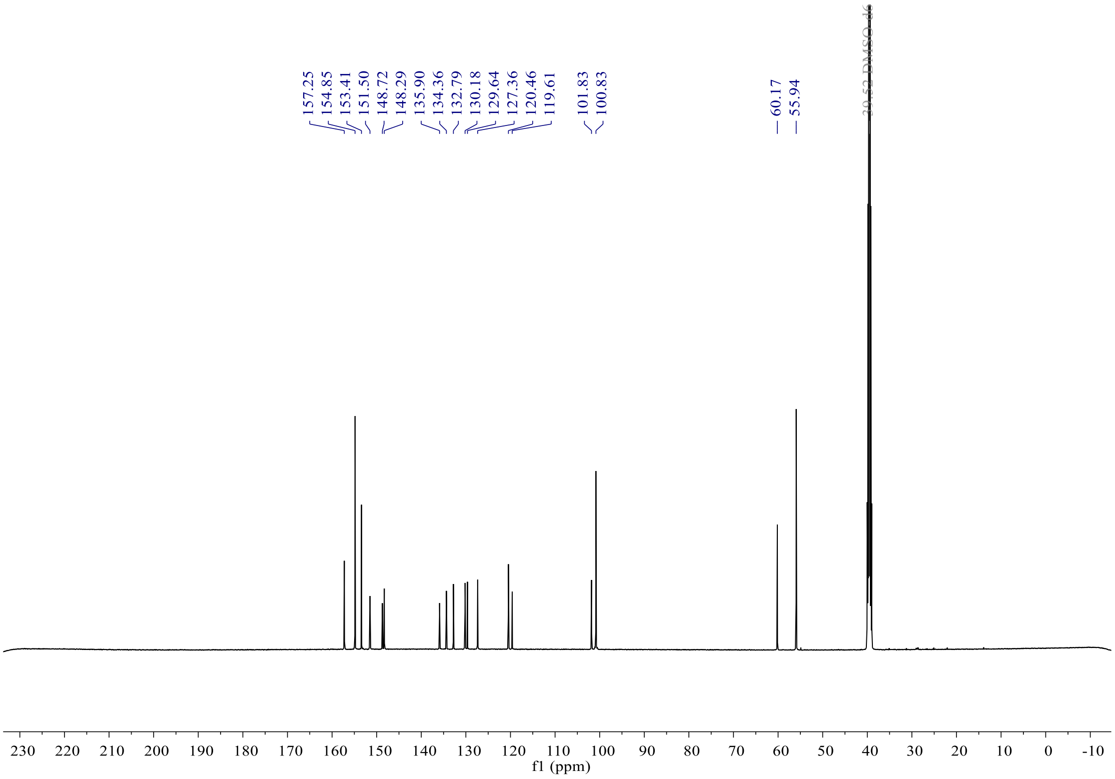

Analysis Report

Graph

Sample Name ftt-10708-119
Application Name Admin App (Administrator)
Method Name User1
Configuration Name Configuration 1
Version 48
Data Instrument Name Detector
Data Channel Name 156 Channel 1
Notes
Injection Number 13

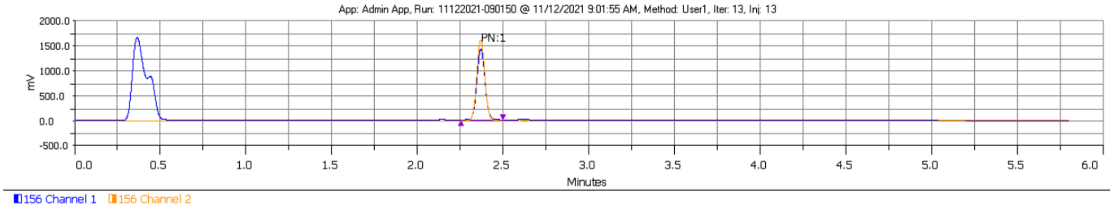

Sample Table

Injection Number	Sample Name	Sample Location	Peak Name	Retention Time (min)	Area (uVmin x100)	Area %	Height (mV)	Plate Number	
13	ftt-10708-119	Sample Zone->41	1	2.373	7815020	100	1420.246	11732.718	

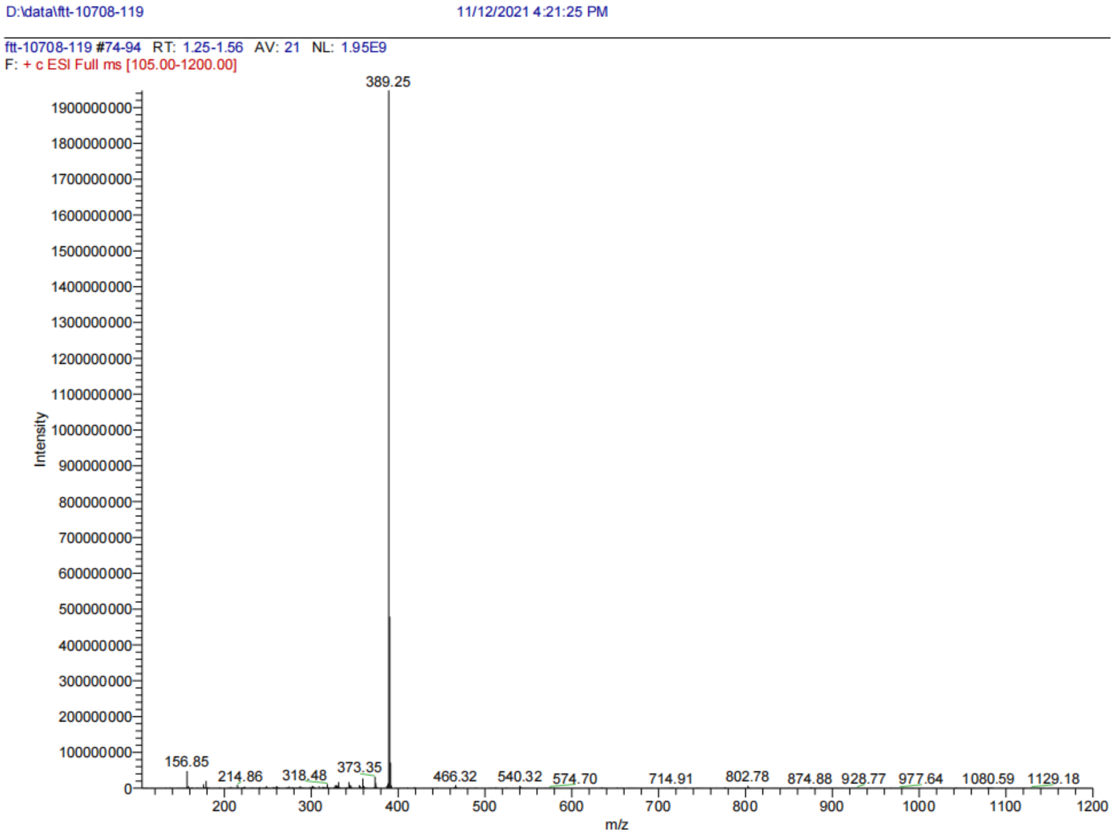

1.35 Compound 35

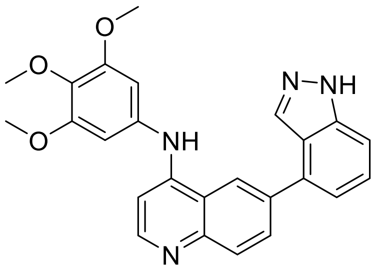

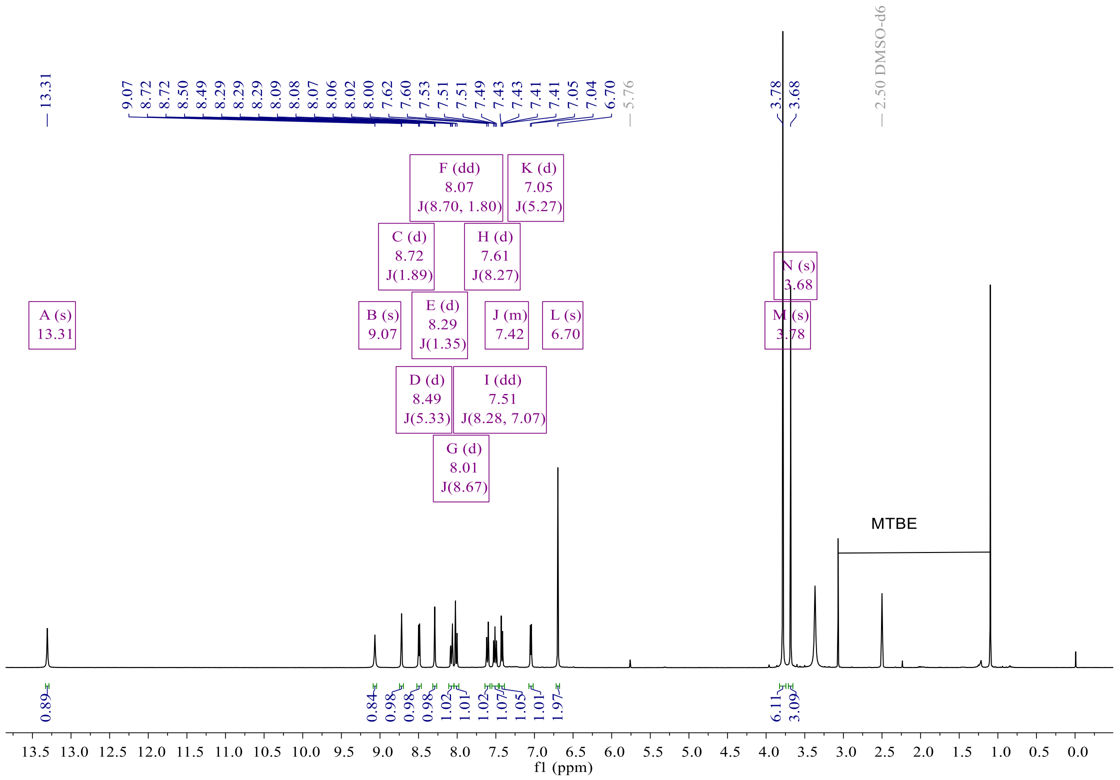

13.31
9.07 8.72 8.72 8.50 8.49 8.29 8.29 8.29 8.09 8.08 8.07 8.06 8.02 8.00 7.62 7.60 7.53 7.51 7.51 7.49 7.43 7.43 7.41 7.41 7.05 7.04 6.70
5.76
3.78 3.68
2.50 DMSO-d6
A (s) 13.31
B (s) 9.07
C (d) 8.72 J(1.89)
D (d) 8.49 J(5.33)
E (d) 8.29 J(1.35)
F (dd) 8.07 J(8.70, 1.80)
G (d) 8.01 J(8.67)
H (d) 7.61 J(8.27)
I (dd) 7.51 J(8.28, 7.07)
J (m) 7.42
K (d) 7.05 J(5.27)
L (s) 6.70
M (s) 3.78
N (s) 3.68
MTBE
0.89 0.84 0.98 0.98 0.98 1.02 1.01 1.02 1.07 1.05 1.01 1.97 6.11 3.09
13.5 13.0 12.5 12.0 11.5 11.0 10.5 10.0 9.5 9.0 8.5 8.0 7.5 7.0 6.5 6.0 5.5 5.0 4.5 4.0 3.5 3.0 2.5 2.0 1.5 1.0 0.5 0.0
f1 (ppm)

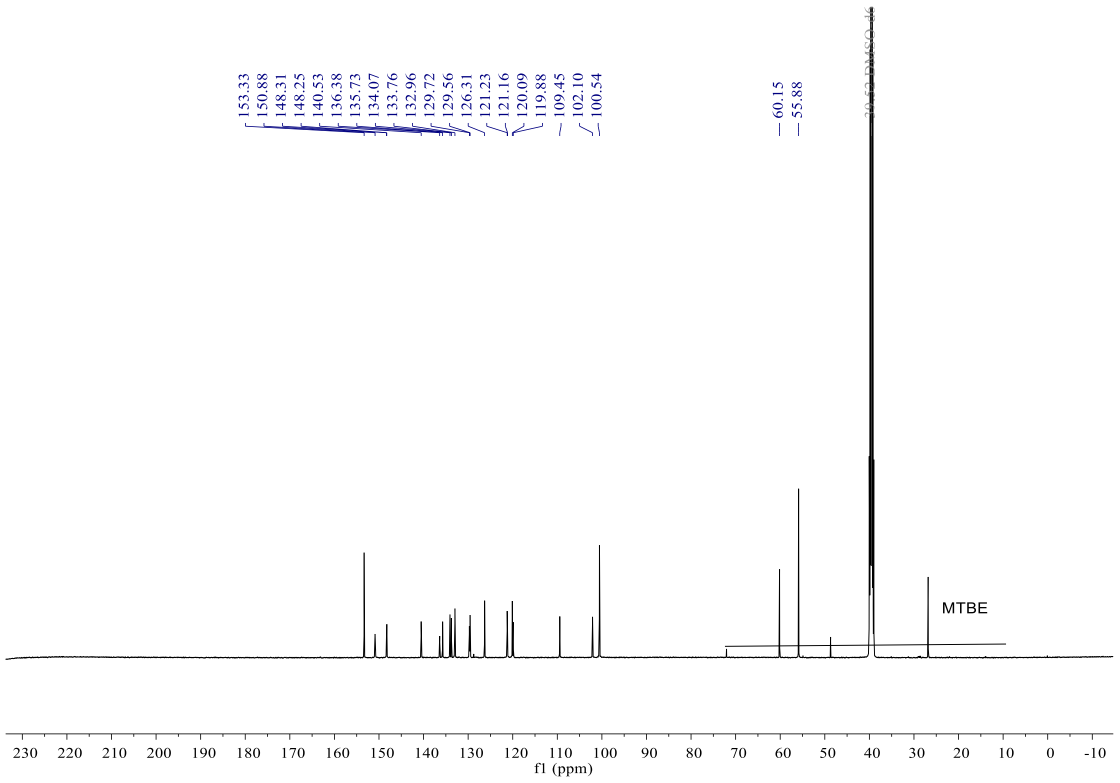

153.33 150.88 148.31 148.25 140.53 136.38 135.73 134.07 133.76 132.96 129.72 129.56 126.31 121.23 121.16 120.09 119.88 109.45 102.10 100.54
60.15 55.88
39.52 DMSO-d6
MTBE
230 220 210 200 190 180 170 160 150 140 130 120 110 100 90 80 70 60 50 40 30 20 10 0 -10
f1 (ppm)

Analysis Report

Graph

**Sample Name**	ftt-10708-120
**Application Name**	Admin App (Administrator)
**Method Name**	User2
**Configuration Name**	Configuration 1
**Version**	42
**Data Instrument Name**	Detector
**Data Channel Name**	156 Channel 1
**Notes**	
**Injection Number**	14

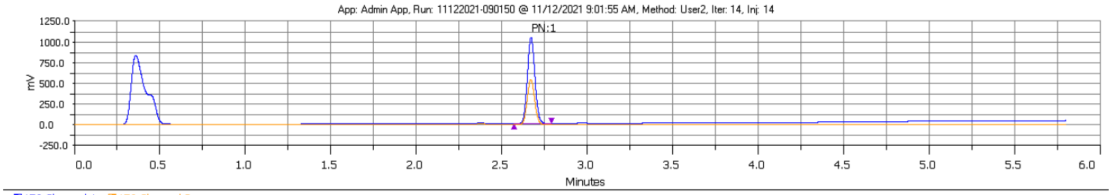

Sample Table

Injection Number	Sample Name	Sample Location	Peak Name	Retention Time (min)	Area (uVmin x100)	Area %	Height (mV)	Plate Number	
14	ftt-10708-120	Sample Zone->42	1	2.674	5311350	100	1040.63	17505.521	

D:\data\ftt-10708-120 11/12/2021 4:24:13 PM

ftt-10708-120 #74-94 RT: 1.25-1.57 AV: 21 NL: 5.87E8
F: + c ESI Full ms [105.00-1200.00]

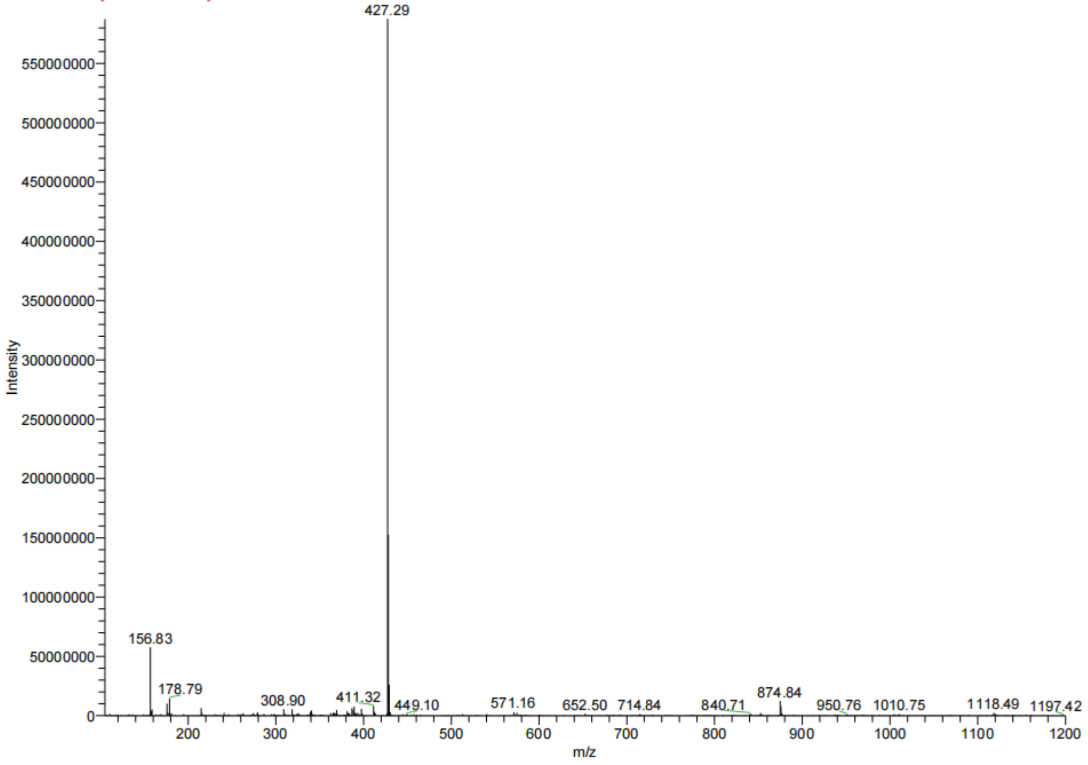

1.36 Compound 36

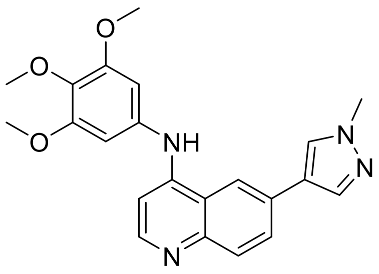

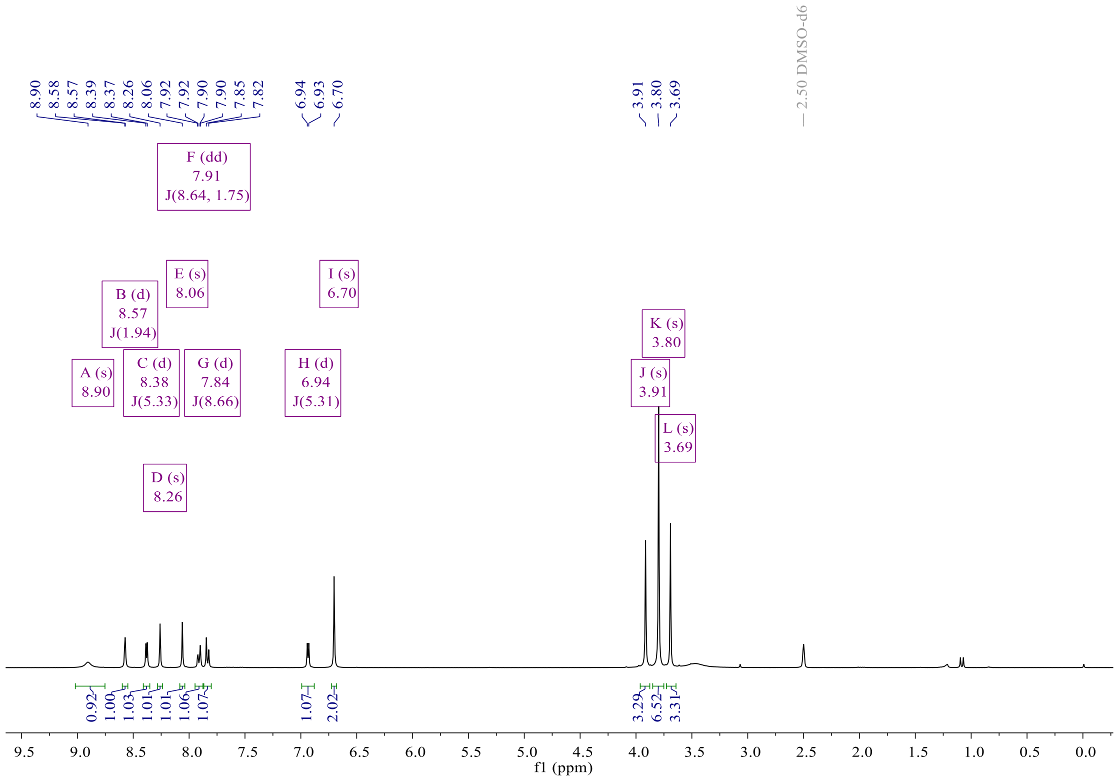

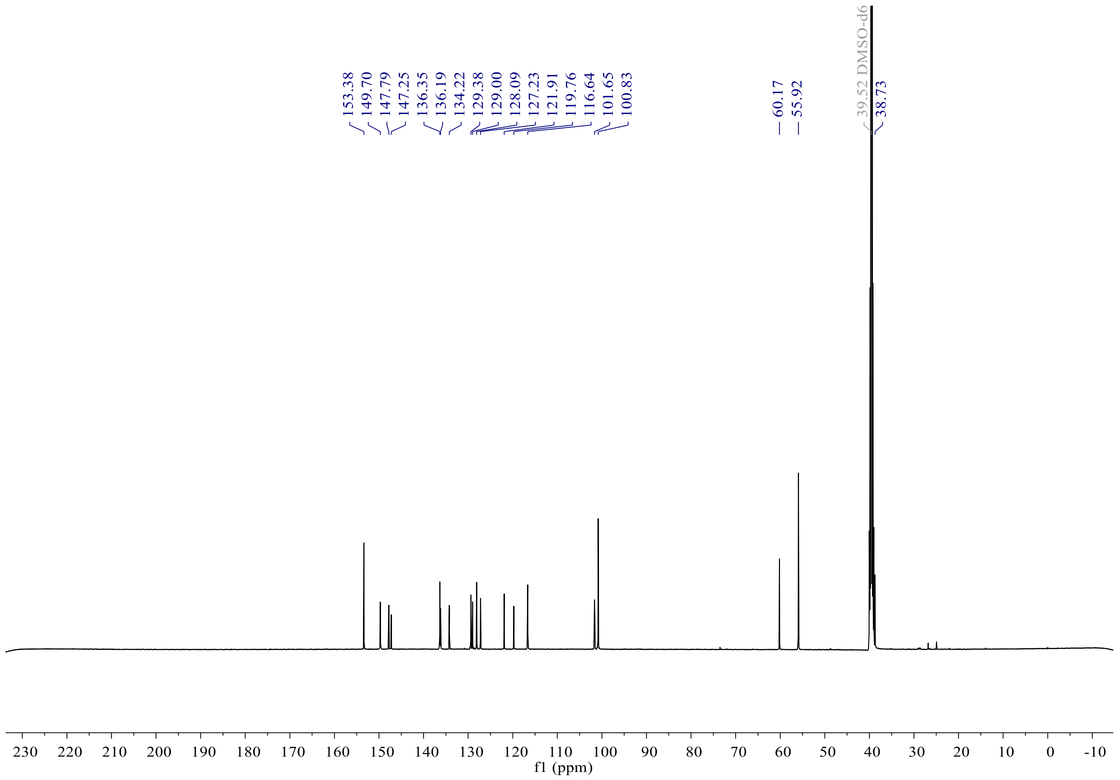

Analysis Report

Graph

Sample Name ftt-10708-121
Application Name Admin App (Administrator)
Method Name User1
Configuration Name Configuration 1
Version 48
Data Instrument Name Detector
Data Channel Name 156 Channel 1
Notes
Injection Number 15

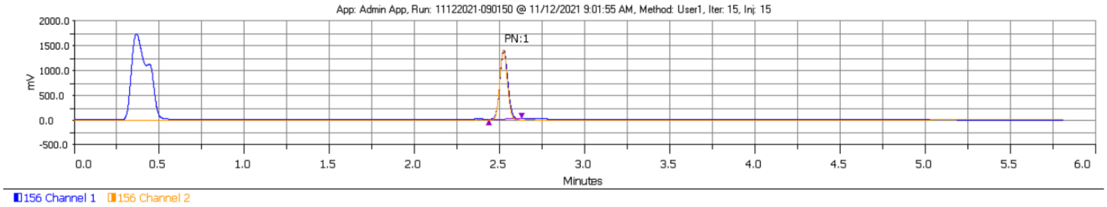

Sample Table

Injection Number	Sample Name	Sample Location	Peak Name	Retention Time (min)	Area (uVmin x100)	Area %	Height (mV)	Plate Number	
15	ftt-10708-121	Sample Zone->43	1	2.527	7584344.1667	100	1393.653	3555.211	

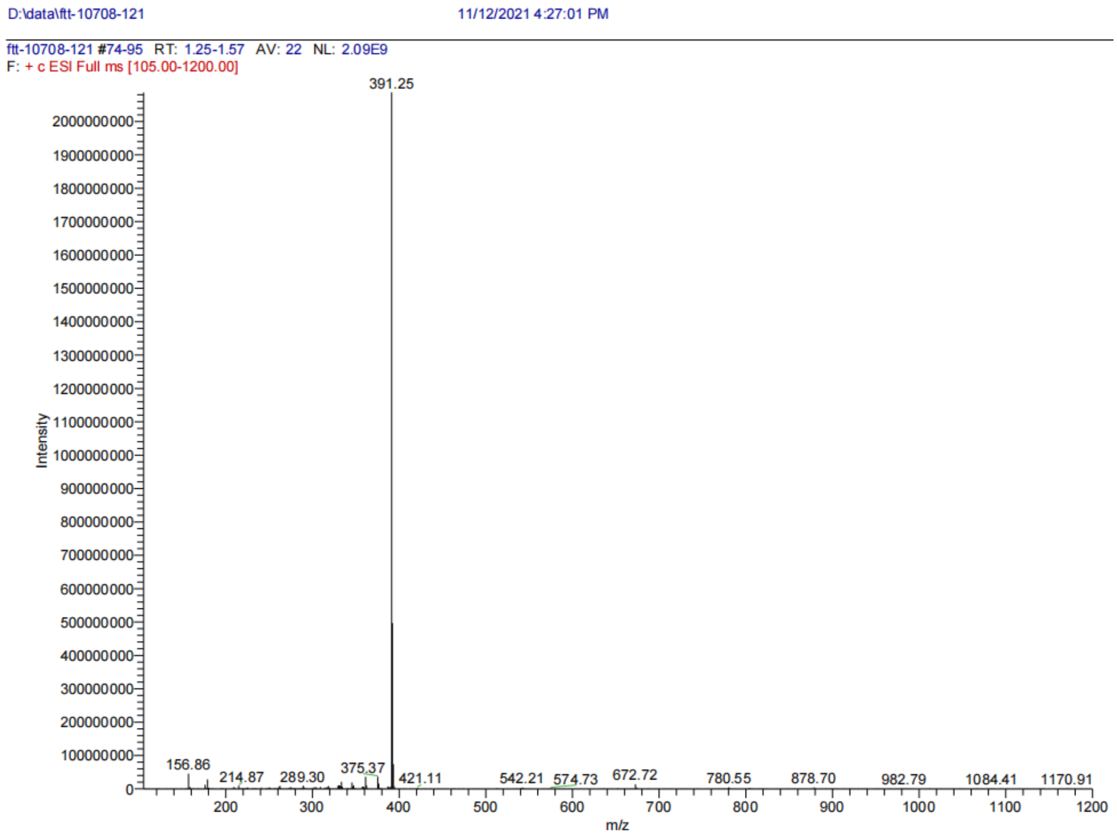

1.37 Compound 37

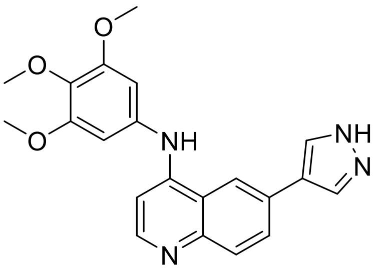

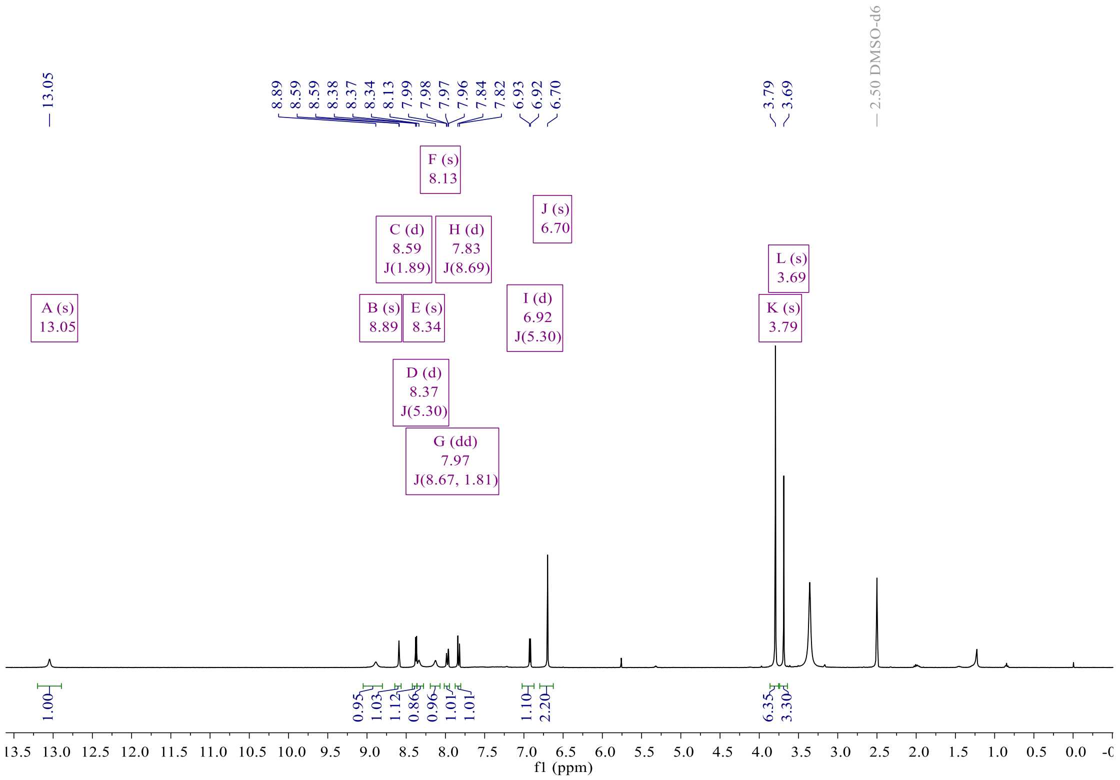
13.05
8.89
8.59
8.59
8.38
8.37
8.34
8.13
7.99
7.98
7.97
7.96
7.84
7.82
6.93
6.92
6.70
3.79
3.69
2.50 DMSO-d6
F (s) 8.13
J (s) 6.70
C (d) 8.59 J(1.89)
H (d) 7.83 J(8.69)
L (s) 3.69
A (s) 13.05
B (s) 8.89
E (s) 8.34
I (d) 6.92 J(5.30)
K (s) 3.79
D (d) 8.37 J(5.30)
G (dd) 7.97 J(8.67, 1.81)
1.00
0.95
1.03
1.12
0.86
0.96
1.01
1.01
1.10
2.20
6.35
3.30
f1 (ppm)

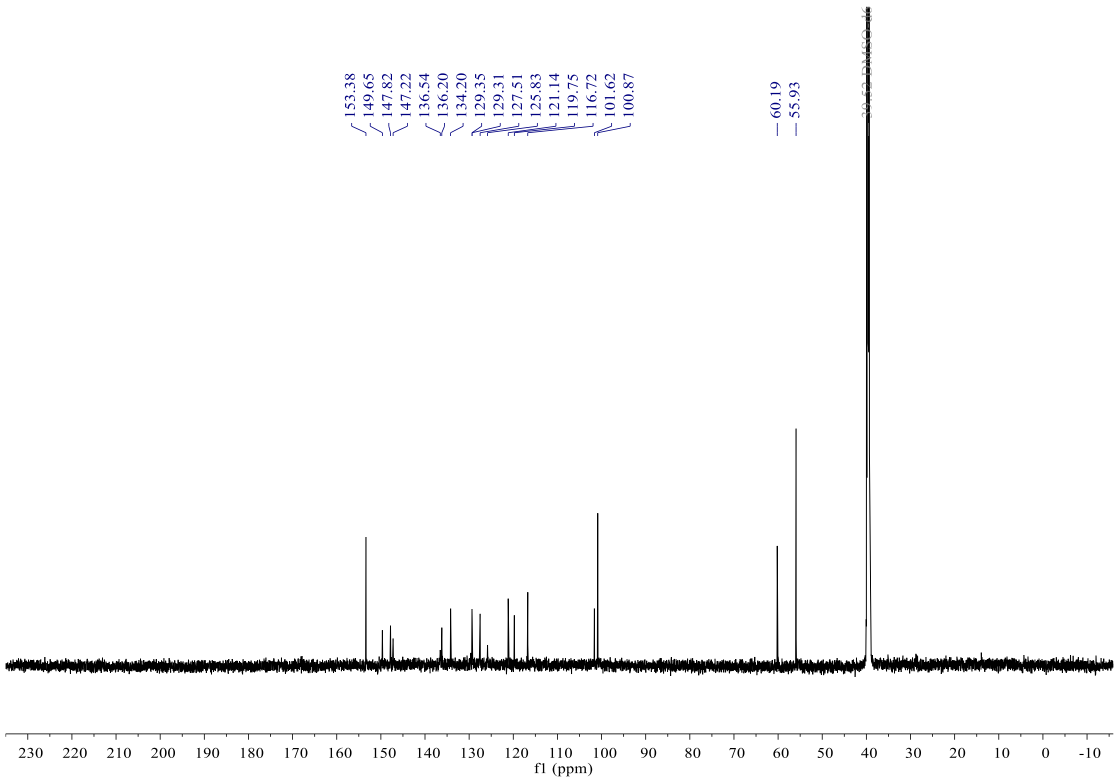
153.38
149.65
147.82
147.22
136.54
136.20
134.20
129.35
129.31
127.51
125.83
121.14
119.75
116.72
101.62
100.87
60.19
55.93
f1 (ppm)

Analysis Report

Graph

**Sample Name**	ftt-10708-122
**Application Name**	Admin App (Administrator)
**Method Name**	User2
**Configuration Name**	Configuration 1
**Version**	42
**Data Instrument Name**	Detector
**Data Channel Name**	156 Channel 1
**Notes**	
**Injection Number**	16

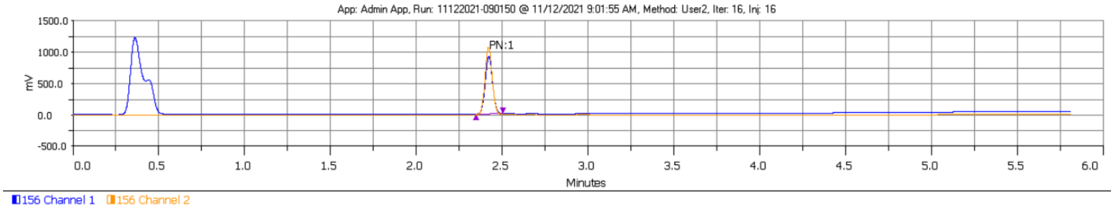

Sample Table

Injection Number	Sample Name	Sample Location	Peak Name	Retention Time (min)	Area (uVmin x100)	Area %	Height (mV)	Plate Number	
16	ftt-10708-122	Sample Zone->44	1	2.425	4563092.5	100	917.57	15046.442	

D:\data\ftt-10708-122 11/12/2021 4:29:49 PM

ftt-10708-122 #75-94 RT: 1.25-1.56 AV: 20 NL: 8.30E8
F: + c ESI Full ms [105.00-1200.00]

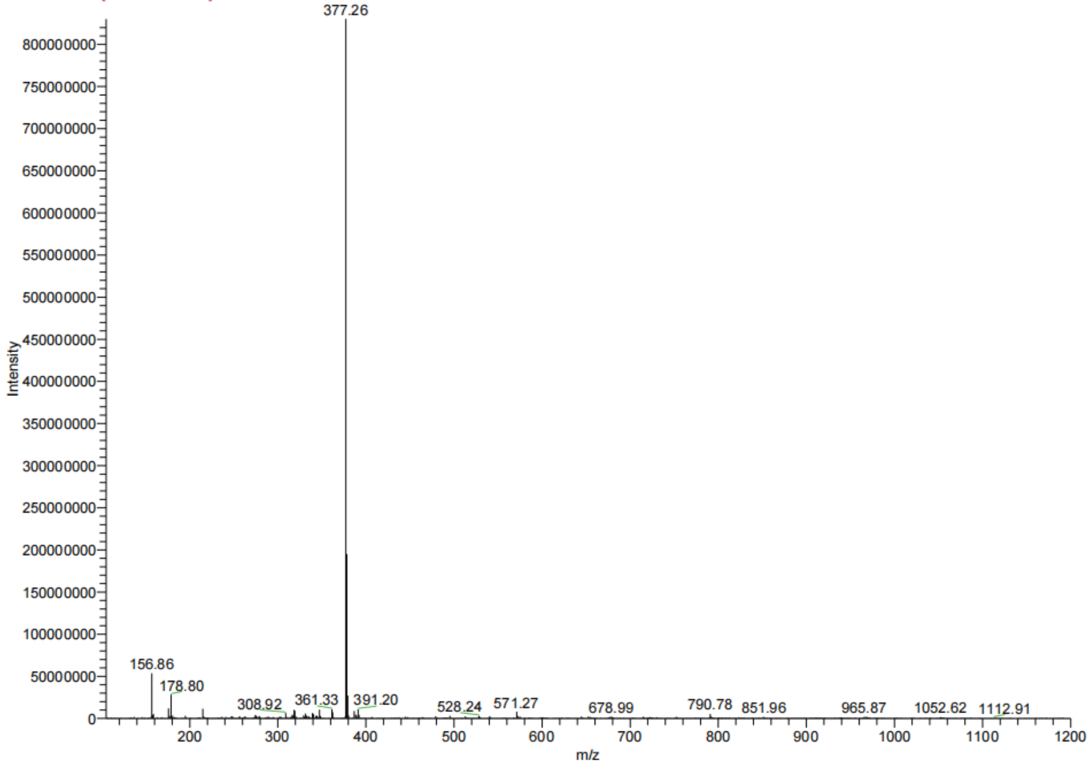

1.38 Compound 38

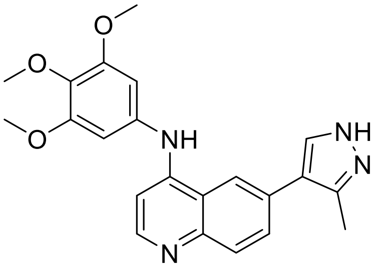

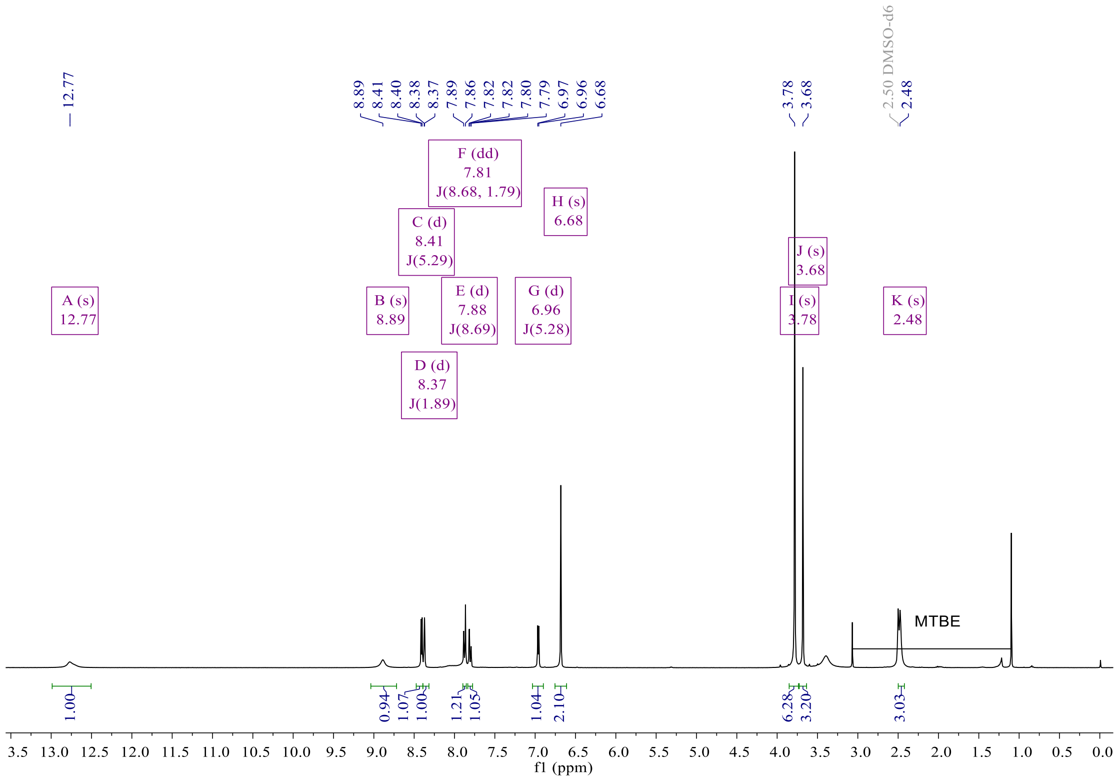

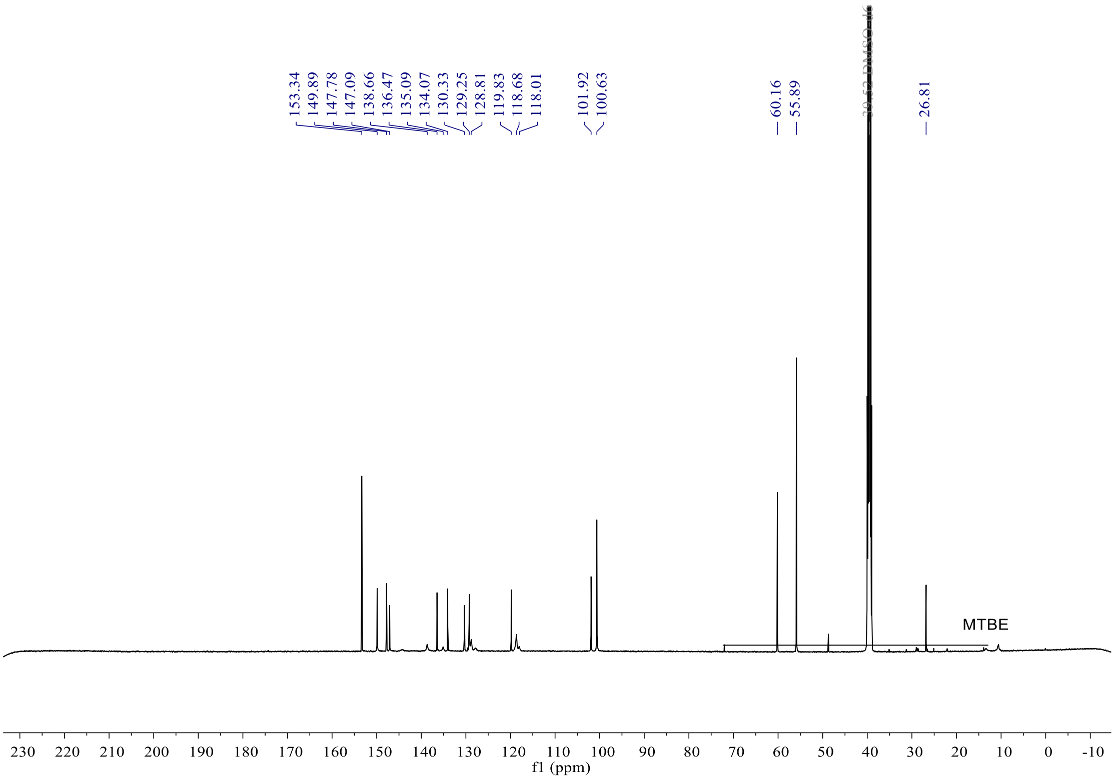

Analysis Report

Graph

**Sample Name**	ftt-10708-123
**Application Name**	Admin App (Administrator)
**Method Name**	User1
**Configuration Name**	Configuration 1
**Version**	48
**Data Instrument Name**	Detector
**Data Channel Name**	156 Channel 1
**Notes**	
**Injection Number**	17

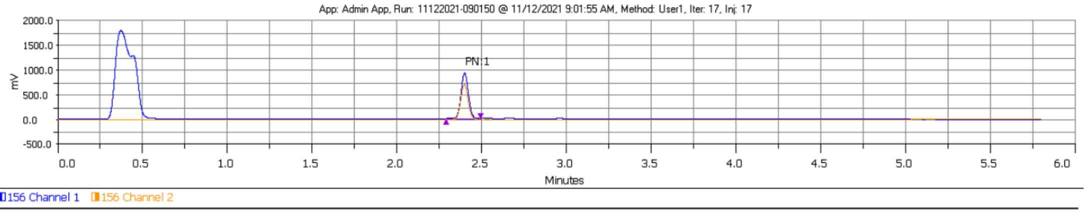

Sample Table

Injection Number	Sample Name	Sample Location	Peak Name	Retention Time (min)	Area (uVmin x100)	Area %	Height (mV)	Plate Number	
17	ftt-10708-123	Sample Zone->45	1	2.402	4615982.5	100	923.782	14717.136	

D:\data\ftt-10708-123 11/12/2021 4:32:37 PM

ftt-10708-123 #74-94 RT: 1.25-1.57 AV: 21 NL: 6.50E8
F: + c ESI Full ms [105.00-1200.00]

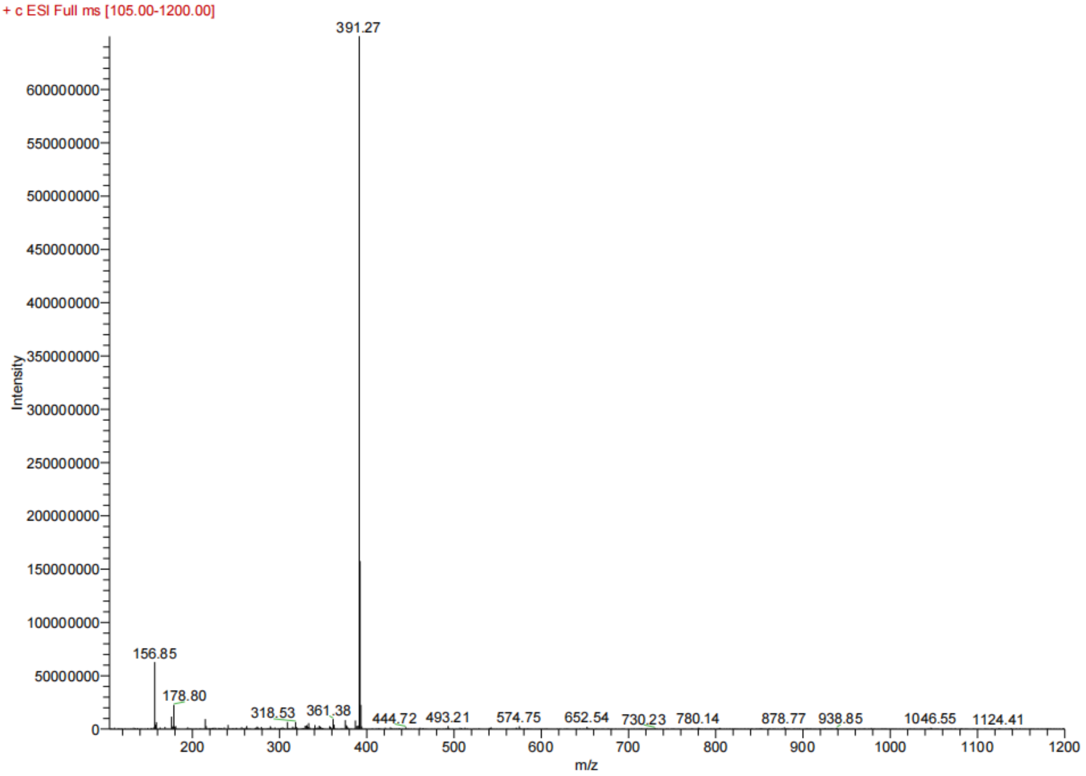

2. Kinase selectivity of compound 14

Table 1. Kinase selectivity of compound **14**

Kinase	14 @ 1 μM	Kinase	14 @ 1 μM
Abl(h)	9	Lyn(h)	3
ALK(h)	77	MAPKAP-K2(h)	95
AMPKα1(h)	103	MEK1(h)	105
ASK1(h)	87	MLK1(h)	89
Aurora-A(h)	95	Mnk2(h)	68
BTK(h)	4	MSK2(h)	107
BTK(R28H)(h)	63	MST1(h)	105
CaMKI(h)	97	mTOR(h)	106
CDK1/cyclinB(h)	90	NEK2(h)	88
CDK2/cyclinA(h)	78	p70S6K(h)	95
CDK6/cyclinD3(h)	100	PAK2(h)	100
CDK7/cyclinH/MAT1(h)	83	PDGFRβ(h)	64
CDK9/cyclin T1(h)	41	Pim-1(h)	78
CHK1(h)	100	PKA(h)	117
CK1γ1(h)	93	PKBα(h)	80
CK2α2(h)	104	PKCα(h)	94
c-RAF(h)	65	PKCθ(h)	74
DRAK1(h)	110	PKG1α(h)	64
eEF-2K(h)	102	Plk3(h)	86
EGFR(h)	104	PRAK(h)	123
EphA5(h)	96	RIPK1(h)	112
EphB4(h)	96	RIPK2(h)	3
Fyn(h)	0	ROCK-I(h)	70
GSK3β(h)	75	Rse(h)	121
IGF-1R(h)	109	Rsk1(h)	98
IKKα(h)	104	SAPK2a(h)	54
IKKε(h)	106	SRPK1(h)	111
IRAK1(h)	91	Syk(h)	94
IRAK4(h)	107	TAK1(h)	90
Itk(h)	78	TBK1(h)	97
JAK1(h)	105	TYK2(h)	108
JAK2(h)	93	PI3 Kinase (p110b/p85a)(h)	88
JAK3(h)	100	PI3 Kinase (p120g)(h)	79
KDR(h)	11	PI3 Kinase (p110d/p85a)(h)	54
LOK(h)	44	PI3 Kinase (p110a/p85a)(h)	81

3. ClogP and LipE

Table 2. ClogP and LipE

No.	ClogP	LipE	No.	ClogP	LipE
**1**	4.04	2.57	**20**	4.98	3.04
**2**	4.16	3.10	**21**	4.43	4.01
**3**	4.93	-	**22**	4.28	3.86
**4**	3.53	3.50	**23**	4.85	2.92
**5**	4.72	2.78	**24**	5.01	2.37
**6**	4.34	3.63	**25**	3.92	3.87
**7**	3.60	-	**26**	3.09	5.00
**8**	3.14	3.68	**27**	3.40	4.99
**9**	3.14	3.24	**28**	4.26	3.46
**10**	3.95	3.97	**29**	4.21	3.81
**11**	3.87	3.21	**30**	4.91	2.80
**12**	4.16	3.49	**31**	4.22	3.87
**13**	4.11	2.82	**32**	4.32	4.30
**14**	3.89	4.40	**33**	4.65	3.26
**15**	3.95	4.27	**34**	3.38	4.58
**16**	4.33	4.11	**35**	4.91	3.24
**17**	4.82	2.80	**36**	4.47	3.67
**18**	3.02	5.81	**37**	3.61	4.22
**19**	4.87	3.08	**38**	3.92	3.79
